# Transcription facilitates sister chromatid cohesion on chromosomal arms

**DOI:** 10.1093/nar/gkw252

**Published:** 2016-04-15

**Authors:** Shweta Bhardwaj, Margarita Schlackow, Miroslava Rabajdova, Monika Gullerova

**Affiliations:** Sir William Dunn School of Pathology, South Parks Road, Oxford OX1 3RE, UK

## Abstract

Cohesin is a multi-subunit protein complex essential for sister chromatid cohesion, gene expression and DNA damage repair. Although structurally well studied, the underlying determinant of cohesion establishment on chromosomal arms remains enigmatic. Here, we show two populations of functionally distinct cohesin on chromosomal arms using a combination of genomics and single-locus specific DNA-FISH analysis. Chromatin bound cohesin at the loading sites co-localizes with Pds5 and Eso1 resulting in stable cohesion. In contrast, cohesin independent of its loader is unable to maintain cohesion and associates with chromatin in a dynamic manner. Cohesive sites coincide with highly expressed genes and transcription inhibition leads to destabilization of cohesin on chromatin. Furthermore, induction of transcription results in *de novo* recruitment of cohesive cohesin. Our data suggest that transcription facilitates cohesin loading onto chromosomal arms and is a key determinant of cohesive sites in fission yeast.

## INTRODUCTION

Cohesin is a conserved multi-subunit protein complex that plays an essential role in sister chromatid cohesion and proper chromosome segregation. Cohesin is also involved in other fundamental processes such as gene expression regulation and DNA damage repair. The core cohesin complex consists of structural maintenance of chromosomes (SMC) proteins, Psm3 and Psm1, and the kleisin subunit Rad21 (Table 1) (1,2). SMC proteins are characterized by a globular hinge domain surrounded by two α-helices that fold back onto themselves at the hinge, thereby bringing the N- and C-termini together to form an ABC-type nucleotide binding domain (NBD) (3). The SMC proteins form V-shaped Psm3-Psm1 heterodimers that interact with the N- and C-terminal domains of Rad21, thus forming the tripartite cohesin ring (4). The ring is further stabilized by the essential subunit Psc3 which is recruited by Rad21 (5). Experimental evidence strongly suggests that cohesion is maintained via topological entrapment of sister chromatids by the cohesin ring (6,7).

**Table 1.** Nomenclature of the cohesin subunits in various organisms

Mammals	*D. melanogaster*	*S. cerevisiae*	*S. pombe*	Function
SMC1A	Smc1	Smc1	Psm1	Core cohesin (mitosis)
SMC1B				Core cohesin (meiosis)
SMC3	Smc3	Smc3	Psm3	Core cohesin
RAD21	Rad21/Vtd	Mcd1/Scc1	Rad21	Core cohesin (mitosis)
REC8	C(2)M	Rec8	Rec8	Core cohesin (meiosis)
SA1/STAG1	SA (Stromalin)	Scc3	Psc3	Core cohesin (mitosis)
SA2/STAG2	SA2 (Stromalin-2)			
SA3/STAG3				Core cohesin (meiosis)

The association of cohesin with chromatin is dependent on a loading complex, which consists of two essential subunits, Mis4 and Ssl3 in fission yeast (8,9). It has been speculated that the loading complex stimulates the ATPase activity of cohesin (10–13) and creates a DNA ‘entry gate’ via the transient opening of the Psm3-Psm1 hinge interface in yeast (14,15). In budding yeast, the activity of a loading complex is counteracted by the ‘anti-establishment’ activity of Wpl1 that destabilizes the Smc3-Scc1 interface (16) and forms a DNA ‘exit gate’ (17). In fission yeast, chromatin bound Psm3 is acetylated by the Eso1 acetyltransferase. Acetylated Wpl1 resistant cohesin exhibits increased dwelling times on chromatin and is believed to be the topologically bound cohesin that entraps sister chromatids until mitosis (Table 2) (18,19).

**Table 2.** Nomenclature of the regulatory proteins involved in the cohesion cycle

Mammals	*D. melanogaster*	*S. cerevisiae*	*S. pombe*	Function
NIPBL/SCC2	Nipped-B	Scc2	Mis4	Cohesin loading
MAU2/SCC4	Mau2	Scc4	Ssl3	Cohesin loading
ESCO1	Eco/Deco	Eco1/Ctf7	Eso1	Cohesion establishment
ESCO2	San			
PDS5A	Pds5	Pds5	Pds5	Cohesion maintenance
PDS5B/APRIN				
WAPL/WAPAL	Wapl	Rad61/Wpl1	Wpl1	Cohesion maintenance
SORONIN/CDCA5	Dmt (Dalmatian)	–	–	Cohesion maintenance
HDAC8	–	Hos1	–	Cohesin deacetylase
Shugosin1	Sse1	Esp1		Protection of centromeric cohesion
Separase	Sse1	Esp1	Separase	Cohesin removal
Polo like Kinase 1 (PLK1)	Polo	Cdc5	Plk1	Cohesin removal

Surprisingly, the underlying determinant of cohesin loading sites remains unclear. Considerable differences have been documented between different eukaryotes. In *Xenopus*, cohesin is recruited to chromatin via Dbf4/Drf1 Dependent Kinase (DDK), a component of the pre-replicative complex (20,21). In contrast, in *S. pombe*, cohesin enrichment at centromeric and peri-centromeric sites is attributed to heterochromatin protein Swi6 (22,23), whereas in *S. cerevisiae* the kinetochore proteins play a critical role in the recruitment of the cohesin complex (24,25). While centromeres and peri-centromeric regions constitute the strongest cohesin binding sites in all eukaryotes, cohesin also binds to ‘Cohesin Associated Regions’ or CARs on chromosomal arms (26–30). In metazoans, cohesin overlaps with the mediator complex, CTCF (CCCTC-binding factor) and tissue-specific transcription factors (27,31–32). In budding yeast, cohesin is actively moved by ongoing transcription away from its loading sites and accumulates between convergent genes (26,28). Interestingly, in *S. pombe* cohesin appears to be a combination of both: it associates with its loader at sites of strong transcriptional activity (29), while a sub-set re-locates and accumulates between convergent genes (33).

Despite such diversity, cohesin exhibits an ordered and highly reproducible chromatin association pattern, suggesting the presence of a hitherto uncharacterized determinant. We have successfully applied a combination of single-locus DNA fluorescent *in situ* hybridization assay and bioinformatics analysis of genome wide ChIP-chip data of cohesin proteins (29) to present a functional analysis of cohesin dynamics across the fission yeast genome. We identified that transcription mediates cohesion establishment on chromosomal arms at the sites of cohesin loading.

## MATERIALS AND METHODS

### Cell culture

Most experiments were performed in *S. pombe* cycling cells (∼80% G2), unless indicated. All strains were cultured in rich or minimal medium supplemented with essential amino acids. Wild type (972), *mis4-367ts* and *rad21-K1ts* cultures were synchronized in G1 by nitrogen starvation in EMM (minus NH_4_Cl) for 16 h. Subsequently, cells were resuspended in rich medium at an OD_600_ = 0.2 and shifted to 37°C, for a total of 8 h. Aliquots were taken at 1 h intervals for FACS. For heat shock experiments, log phase cultures were shifted from 32 to 42°C for 30 min and processed for FISH or ChIP-qPCR. *Cdc25-22* or *cdc10-129* strains were grown at 25°C, followed by a shift to 37°C to synchronize the cells in G2 or G1 respectively. All strains used in this study are listed in Supplementary Table S3.

Mammalian cell line HEK293T was cultured in DMEM supplemented with 10% FBS + antibiotics. For α-amanitin treatment, cells were grown to 70% confluency and treated with α-amanitin at 2 μg/ml for 36 h (recommended incubation time is 12–48 h) (34) and processed for ChIP-qPCR or western blotting.

### Chromatin immunoprecipitation (ChIP)

ChIP was performed using exponential cultures (OD_600_ < 0.5) according to previously published protocols. Cells were crosslinked with 3% para-formaldehyde for 30 min; glycine quenched and lysed in buffer I (50 mM HEPES (pH 7.5), 140 mM NaCl, 1 mM EDTA (pH 7.5), 1% Triton X-100, 0.1% sodium deoxycholate, protease inhibitiors) using the MagnaLyzer. Whole cell lysates were sonicated with a diagenode Bioruptor at high intensity for 15 min, with 30 s ON/OFF intervals. DNA was visualized on an agarose gel showing fragments of 200–500 bp. 100 μg of sonicated, precleared chromatin was incubated per antibody at 4°C overnight. In experiments with RNase treatment, mix of RNase A/T was added 30 min prior to incubation with antibodies. Crosslinked immuno-complexes were captured with Protein A agarose beads (Millipore) and washed once each with Buffer I, Buffer II (50 mM HEPES (pH 7.5), 500 mM NaCl, 1 mM EDTA (pH 7.5),1% Triton-X-100, 0.1% sodium deoxycholate), Buffer III (10 mM Tris-Cl (pH 8.0), 250 mM LiCl, 1 mM EDTA (pH 7.5), 0.5% NP-40, 0.5% sodium deoxycholate) and TE (10 mM Tris–Cl and 1 mM EDTA). Immunoprecipitated DNA was eluted from the beads in TE + 1% SDS. Samples were reverse crosslinked with 3 μg/ml of RNaseA (Roche) at 65°C overnight, treated with 20 μg Proteinase K (Roche) at 45°C for 2 h and purified using Qiagen PCR purification columns. DNA was eluted twice with 35 μl MilliQ water. 2 μl of each sample was used for qPCR (SensiMix, Bioline). In experiments with chemical treatment, cells were treated with 1,10-phenananthroline (300 μg/ml in ethanol, 30 min) and ChIP-qPCR analysis was performed as above. Antibodies were obtained from Abcam; Anti-GFP (ab290), RNAPII (ab817) and histone H3 (ab1791). All primer pairs used for ChIP analysis are listed in Supplementary Table S5.

### FACS

To determine cell cycle progression the DNA content was measured by propidium iodide staining of ethanol-fixed cells. 1 ml aliquots were pelleted and fixed in cold 70% ethanol. Pellets were resuspended in 500 μl of 50 mM sodium citrate and treated with 1 mg/ml RNaseA, 2 h at 37°C. Pellets were washed with 50 mM sodium citrate, resuspended in 50 mM sodium citrate + propidium iodide (8 μg/ml) and incubated at 4°C for 2 h (to overnight). Subsequently, pellets were washed with 50 mM sodium citrate and DNA content analyzed using the CellQuest pro software for the FACS Calibur machine.

### Data analysis

ChIP-chip data (GEO accession number GEO:GSE13517) were reanalyzed. All data for Pk9 tagged Rad21, Psc3, Pds5, Mis4, Ssl3 and untagged control were normalized to control whole cell extract (WCE) fraction values. The original data generated on the Affymetrix S_pombea520106F platform was adjusted to the current *S. pombe* annotation (EF2 annotation from the iGenomes database; this annotation is equivalent to the current ASM294v2 annotation). In particular, we observed a shift of 80,031 bp in the genome annotation on chromosome 2 since 2004 and two regions of 1000 ‘N's were replaced by 100 ‘N's. Data was plotted in MATLAB.

Swi6 data was obtained from the first biological repeat performed previously (35) (GEO:GSE3186, sample GSM71577). Data were generated with the NCI Pombe 44K v1.0 platform from 2005 and readjusted to the current *S. pombe* annotation similarly to above.

### Peak-caller

The MATLAB peakfinder algorithm was employed to log_2_ normalized signal data (http://www.mathworks.co.uk/matlabcentral/fileexchange/25500-peakfinder). The algorithm employs a minimal threshold and a cut-off value, by a factor of which the peak must be higher than the average surrounding data. Based on data noise and distribution this threshold was set to 0.3 for Rad21, 0.4 for Swi6, 0.5 for Mis4 and Sfc6, and 0.7 for all other datasets. The cut-off was set to 0.2 for all datasets. Peaks were extended to both sides until log2 reached 0 (corresponding to the value where WCE normalization data becomes higher than the specific signal). The identified peaks were further subjected to a custom written Perl code. The criteria employed were a minimal peak-width of 1000 bp and an average signal throughout the peak. According to data distribution the average signal value throughout a peak was chosen as 0.2 for Rad21, 0.3 for Mis4, Sfc6 and Swi6, and 0.4 for all other datasets. The average peak signal was computed as the average of the log_2_ values of each probe within the peak.

### Gene expression analysis

Gene expression in reads per kilobase per million (RPKM) values were computed as the average from previously published RNA-Seq data (36) generated from two biological replicates. The RPKM value of the most highly expressed overlapping gene was assigned to the peak. This was applied to Rad21 peaks, which were split into groups: overlapping with a called Mis4 peak and not overlapping with a called Mis4 peak. Likewise, for called Ssl3 peaks. RPKM values, which were overlapping a called peak in Mis4 or Ssl3 data, were extracted to compute gene expression values for each of these four sets. Note, the peak at 4,494,276 was not taken into consideration for the above analysis as it does not overlap any genes and may be part of the adjacent peak (not considered as such due to a possible data generation artifact, where surrounding probes give 0 signal).

### Genome-wide ChIP analysis for *S. cerevisiae* and *H. sapiens*

*S. cerevisiae* cohesin and cohesin loader ChIP-Seq data was obtained from a previous study (11). Data were mapped to the *S. cerevisiae* genome SacCer3 using Bowtie with the following parameters: -m 1 -k 1 -v 3 –C (-m: permissible maximal number of alignments found for any read; -k: permissible maximal number of alignments to be reported for any read; -v: permissible number of mismatches end to end of any read; -C: color space alignment). ChIP-Seq data for mammalian cohesin subunit (SMC1) and RNAPII in HB2 cells were extracted from (37). ChIP Seq data was mapped to hg19 with Bowtie using the options -m 1 -k 1 –n 1 -S (-m: permissible maximal number of alignments found for any read; -k: permissible maximal number of alignments to be reported for any read; -n: permissible number of mismatches in seed region of any read; -S: report alignments in SAM output).

### Gene expression analysis for *S. cerevisiae* and *H. sapiens*

SCC1 peaks were considered in a RNAPII transcribed region, if they overlapped with any genes from the *Saccharomyces* Genome Database (SGD). These were obtained from the UCSC table browser. RNAPIII regions are defined as tRNA loci obtained from the SGD.

*S. cerevisiae* gene expression values in RPKM were re-analyzed from previously published study (38) and averaged over two biological repeats.

RNA-Seq from HB2 cells was mapped to hg19 using Tophat2 with the following parameters: -G genes_hg19.gtf -g 1 -p 8 –segment-length 15 –no-coverage-search, where genes_hg19.gtf is the gtf file containing all the human genes downloaded from UCSC. The mapped Bam file was further processed to determine RPKM values with Cufflinks2, which were used as gene expression values for human genes.

Called SCC2 and SMC1 peaks were extended at each end by 200nt for overlap analysis, as this is the estimated fragment size (39).

### Meta analysis

Peaks for *S. cerevisiae* data were called using MACS with default parameters. Hyper-ChIPable region in *S. cerevisiae* had been annotated previously (40).

Peaks for human SMC1, NIPBL and RNAPII were called with MACS2 using the –broad parameter (default parameters for SMC1 and NIPBL, –broad-cutoff = 0.01 for RNAPII). Further RNAPII peaks were pooled into one if they were less than 10 kb apart.

### Genomic distances

Genomic distance of Rad21 peaks at Mis4+/Rad21+ and Rad21+ sites to nearest Pds5 peaks were computed as the distance between annotated margins of the respective peaks. Any overlap was taken as distance 0.

### Distance profiles

Distances of called Sfc6 and Pds5 peaks from called cohesive and non-cohesive peaks were plotted as histograms. Data was binned into 5000 bp bins and normalized to the size of dataset under consideration (cohesive or non-cohesive peaks).

### Boxplots

Boxplots were created using R. The median is presented as a line, upper and lower quartiles (q3 and q1 respectively) are presented as a box. The whiskers are given by q3 + 1.5(q3 – q1) (upper) or q1 – 1.5(q3 – q1) (lower). The default of 1.5 corresponds to approximately ±2.7σ and 99.3 coverage if the data are normally distributed. Outliers are plotted if their value is higher than the upper whisker or smaller than the lower. The plotted whiskers extend to the adjacent value, which is the most extreme data value that is not an outlier.

### Statistical analyses: comparisons of two sets of peaks

To identify whether cohesive peaks localize in significantly higher expressed regions than non-cohesive peaks the Wilcoxon Rank Sum test was applied to compute the one-sided *P*-value. The Wilcoxon Rank Sum test was also applied to the absolute values of peak distances to Pds5 peaks. *P*-values were further verified by a permutation test.

### Fluorescent *in situ* hybridization

Wild type (or *ts* mutants) cells were grown overnight to mid-log phase (OD_600_ = 0.5). The following day, cells were re-inoculated into fresh medium and incubated for an hour until the culture had an OD_600_ = 0.30–0.35. Nearly 3 × 10^7^ cells were resuspended in 1.2 M sorbitol and gently swirled for 5–10 min. Cells were fixed in freshly prepared 3% para-formaldehye at 30°C for 30 min and quenched with 104 mM glycine. Subsequently, cells were harvested at 3000 rpm, 5 min and washed twice with PEM buffer (100 mM PIPES, 1 mM EGTA, 1 mM MgSO_4_). Cells were transferred to 1.5 ml sterile screw cap tubes, washed with PEMS (PEM + 1.2 M sorbitol), spheroblasted with 100 U of Zymolyase 100T in PEMS at 37°C for 1 h and pelleted twice at 2000 rpm for 1 min by turning the caps for each spin to collect maximum cells. Cytoplasm was permeabilized in PEMS + 1% Triton-X-100 for exactly 6 min. Pellets were washed three times with PEM, resuspended in PEMBAL (PEMS + 1% BSA, 0.1% NaN_3_, 100 mM lysine monohydrochloride) containing 1 mg/ml RNaseA and incubated at 37°C for 2–4 h.

Hybridization was performed in solution. RNaseA treated cells were resuspended once in each of 200 μl 2× SSC, 2× SSC + 10% formamide, 2× SSC + 20% formamide and 2× SSC + 40% formamide, 15 min at room temperature. Finally, pellets were resuspended in 100 μl hybridization buffer (10% dextran sulphate, 5× Denhardt's, 50% formamide, 0.5 mg/ml salmon sperm DNA, 2× SSC) and labeled FISH probe was added at a final concentration of 1 ng/μl. The hybridization mix (cells + probe) was denatured at 75°C for 5 min, followed by 2 min on ice and finally, incubated at 40°C overnight in a thermomixer at 700 rpm. Next day, cells were washed three times with 2× SSC for 15 min at 700 rpm, 37°C and DNA stained DAPI. Excess DAPI was removed by a wash in PBS only and cells were resuspended in 30–50 μl PBS. For microscopy, ∼7 μl cells were dropped on poly-l-lysine coated slides and covered with 22 × 22 mm coverslips. Excess liquid was removed by blotting and slides were sealed with nail varnish. When imaging was not performed immediately, slides were stored at 4°C in the dark.

Image acquisition was performed using SoftWorx program (Applied Precision) on the DeltaVision microscope (Applied Precision). Cells were visualized with a 100 × 1.35 NA objective lens. For each experiment, z-sections were taken at 200 nm intervals. Background light was corrected by the built-in deconvolution algorithm of the SoftWorx program. Final images represent maximum intensity projections of z-stacks obtained using the ImageJ/Fiji software (NIH).

FISH probes were prepared using the FISH-Tag Kit (Molecular Probes, Invitrogen) with either Alexa-488 or Alexa-555 fluorophores. All FISH probes (except the centromeres) spanned ∼10 kb. The centromeric probes targeted the *dg* region in the outer most centromeric repeats and were ∼2.2 kb. Overlapping PCR fragments (∼2.5 kb) were amplified using Phusion DNA polymerase and pooled in equimolar quantities. All primer pairs used for the probes preparation are listed in Supplementary Table S4. The labeling reaction was performed according to the manufacturers’ instructions. The strategy involved modifying the template DNA by incorporating aminolyated nucleotides, which then acted as a bait to couple the reactive dye.

## RESULTS

### Cohesive and non-cohesive loci on chromosomal arms

**Figure 1.**
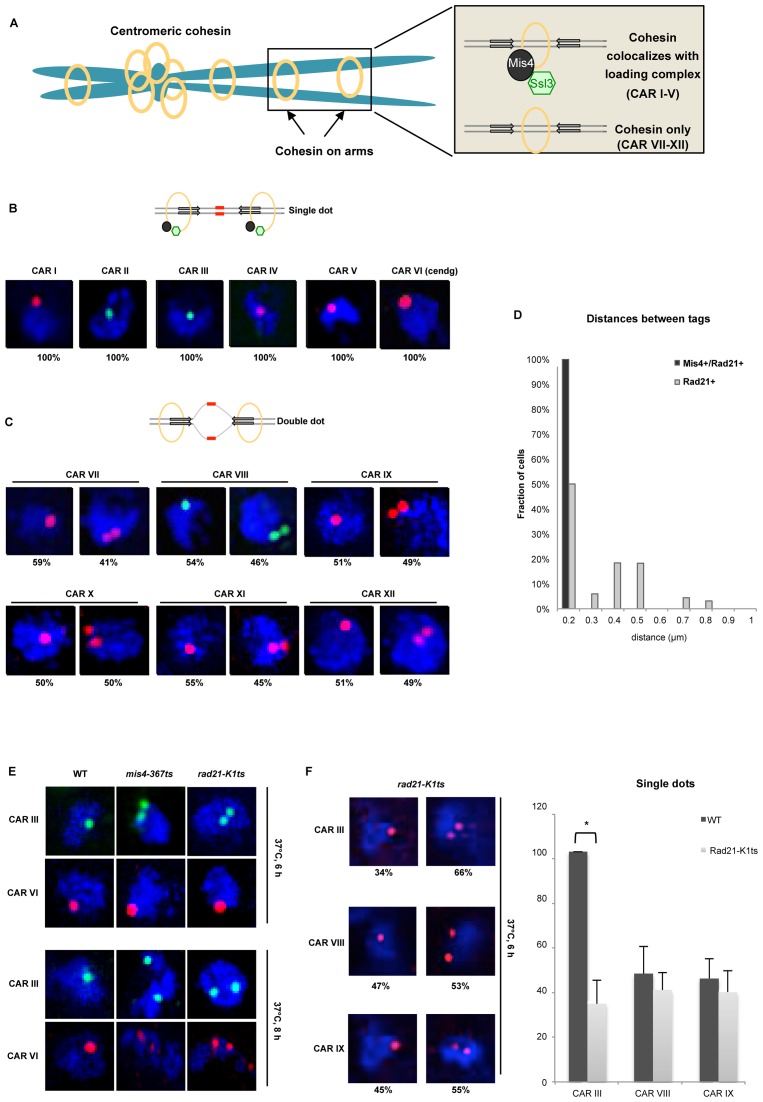
Cohesive and non-cohesive subsets of cohesin on chromosome arms in *S. pombe*. (**A**) An illustration of sister chromatids (blue) held together by the ring shaped cohesin complex (yellow) at centromeres and across the arm regions. The inset depicts cohesin enrichment at intergenic regions between convergent genes (grey arrows) on chromosome arms in *S. pombe*, wherein cohesin localizes with (top panel) or without (bottom panel) its loader Mis4-Ssl3 (black-green). (**B**) Top: schematic showing cohesed sister chromatids at Mis4+/Rad21+ sites visualized by DNA-FISH (red probes). Bottom: Single locus specific DNA FISH showing single dots at CARs I to VI (Mis4+/Rad21+ sites) in G2 cells (selected by septation index) (*n* = 200, three biological repeats). Red (Alexa-555) or green (Alexa-488) probes are shown inside DAPI stained nucleus (blue). (**C**) Top: schematic showing non-cohesed sister chromatids at Rad21+ sites visualized by DNA-FISH (increased separation between red probes). Bottom: Similar amount (±50%) of single-double dots at CAR VI to XII in G2 cells (selected by septation index) (*n* = 200, three biological repeats). Red (Alexa-555) or green (Alexa-488) probes are shown inside DAPI stained nucleus (blue). (**D**) Inter-chromatid distance measurement between sister chromatids at Mis4+/Rad21+ and Rad21+ regions. Z-stacks were converted into 2D images and distances between two spots measured using line scan tool of the ImageJ software. (E) DNA-FISH analysis in wild type (WT), *mis4-367ts* and *rad21-K1ts* strains after Mis4 and Rad21 inactivation by shift to 37°C. Top: cohesion at the arms, CAR III (green dots) was destabilized but cohesion at centromeres, CAR VI (red dots) was unaffected after 6 h at 37°C. Bottom: defective arm (CAR III) and centromeric cohesion (CAR VI) after 8 h at 37°C. Arm and centromeric cohesion was unaffected in WT throughout (*n* = 100 cells). (*F*) DNA-FISH analysis in *rad21-K1ts* strains after Rad21 inactivation by shift to 37°C. CARs III, VIII and IX were analyzed. Cells showing single or double dots were counted and plotted in a bar graph. * *P* < 0.05, two-tailed, paired Student's *t*-test, Error bars represent SD, *n* = 3.

This study builds on previously published ChIP-chip data that provide binding profiles of cohesion proteins across chromosomes 2 and 3 in *S. pombe* (29). Cohesin co-localizes with its loading complex Mis4-Ssl3 (Figure 1A, inset top panel), but surprisingly, there are also cohesin peaks detected away from its loading sites (Figure 1A, inset bottom panel). It has been shown previously that cohesin can be pushed by ongoing transcription across a gene (26,33). However it remained unclear how cohesin could be re-located from its loading sites across several kilobases and various transcription units. Furthermore, recent studies showed that cohesin could bind to DNA *in vitro* through spontaneous topological but inefficient interaction. Cohesin loader Mis4 stimulates ATPase activity of cohesin, resulting in its efficient binding to DNA (12).

We re-analyzed the distribution of cohesin subunits Rad21 and Psc3; cohesin loader Mis4-Ssl3 and cohesin maintenance protein Pds5 from ChIP-chip data (29). We used our own peak-calling algorithm to match the current *S. pombe* annotation, to avoid data smoothing and to maintain consistency between datasets. We identified 283 peaks for Rad21 and 150 peaks for Mis4 on chromosome 2 (all peaks shown in Supplementary File 1), which include >90% Rad21 and >82% Mis4 of previously described peaks (see Supplementary File 2 for comparison in log_2_ and linear scale).

In agreement with previous analysis, only 33% of Rad21 peaks overlap with Mis4. We selected six Rad21 positive regions, CAR I to VI that co-localize with Mis4-Ssl3 (Mis4+/Rad21+) and a further six that do not co-localize with Mis4-Ssl3, CAR VII to XII (Rad21+, Supplementary Tables S1 and S2). CAR VI derives from centromeric degenerate repeats (cendg) and is used as a positive control. Detailed profiles of Rad21 (green), Mis4 (red) and a no tag control (blue) across all selected CARs can be seen in Supplementary Figure S1.

We employed DNA FISH to test cohesion at selected CARs. A 10 kb long unique probe was fluorescently labeled and hybridized to a single CAR resulting in single (Figure 1B, schematic) or double dots (Figure 1C, schematic). Single dot represents cohesion, whilst double dots are a result of locally separated sister chromatids. We observed 100% of cells exhibiting single dots at Mis4+/Rad21+ loci in G2 cells (selected by septation index = 0) (Figure 1B, CARs I–VI and Supplementary Figure S2A) indicating stable cohesion between chromatids at these sites. In contrast, Rad21+ loci showed ∼50% of cells with single or double dots (Figure 1C, CAR VII–XII and Supplementary Figure S2B) indicating reduced cohesion at these loci. Furthermore, sister chromatids at Mis4+/Rad21+ sites were <0.2 μm apart, while the inter-chromatid distance at Rad21+ loci increased from >0.2 to 0.9 μm (Figure 1D).

To confirm that FISH signals do indeed represent functional cohesion, we employed temperature sensitive *(ts)* Mis4 and Rad21 mutants. First, wild type (WT), *mis4-367ts* (9) and *rad21-K1ts* (41) strains were synchronized in G1 by nitrogen starvation, transferred to rich medium and simultaneously shifted to 37°C to inactivate Mis4 and Rad21 proteins. Cell cycle progression for WT and *rad21-K1ts* was monitored by FACS analysis. Nitrogen starved cells exhibit a ∼2 h lag period before re-entering the cell cycle (42), likewise WT moved from 1C to 2C DNA content between 3–5 h at 37°C (Supplementary Figure S3A), whereas *rad21-K1ts* completed replication between 4–5 h. Subsequently, DNA content in *rad21-K1ts* became heterogeneous, predominantly between 6–8 h at 37°C (41). While WT maintained punctate nuclei throughout, *mis4-367ts* and *rad21-K1ts* exhibited DNA fragmentation at 6–8 h, indicating loss of cohesion due to the inactivation of Mis4 and Rad21 (Supplementary Figure S3B).

Next, we analyzed cohesion on chromosomal arms (CAR III) and centromeres (CAR VI) after 4, 6 and 8 h at 37°C. Cohesion was intact in WT throughout this time course (Figure 1E and Supplementary Figure S3C). In contrast, cohesion on the arms (CAR III) was destabilized in *mis4-367ts* and *rad21-K1ts* mutants after 6 h at restrictive temperature (Figure 1E, top panel). Centromeric cohesion was eventually destabilized after 8 h of Rad21 or Mis4 inactivation (Figure 1E, bottom panel). These observations confirm that our FISH assay is biologically functional and specific for physiological cohesion of sister chromatids.

FISH analysis of Rad21+ loci resulted in single dots in ∼50% of cells. To test whether single dots at CARs VII-XII are cohesin dependent, we performed FISH experiment in *rad21-K1* cells at restrictive temperature. We employed probes specific for one Mis4+/Rad21+ (CARIII) and two Rad21+ (CARs VIII and IX) loci (Figure 1F). We do confirm the loss of cohesion at CARIII in 66% of cells (34% remain with single dot after Rad21 inactivation). The loss of cohesion from 100% to 34% is significant (*P* < 10^−3^). Surprisingly, we see no significant loss of cohesion at Rad21+ CARs after Rad21 inactivation. For CARs VIII and IX, the number of cells with a single dot dropped from 47% to 40% and 45% to 39%, respectively. These changes are not statistically significant (*P* = 0.269 for CAR VIII and *P* = 0.344 for CAR IX) (Figure 1F). Therefore, we conclude that detected single dots at Rad21+ sites are most likely a result of random chromosome breathing or just general close proximity of sister chromatids.

Overall, our DNA FISH analysis demonstrates that Mis4+/Rad21+ loci are associated with stable cohesion, while Rad21+ loci display cohesin's dynamic association with chromatin.

### Mis4+/Rad21+ sites associate with Pds5

**Figure 2.**
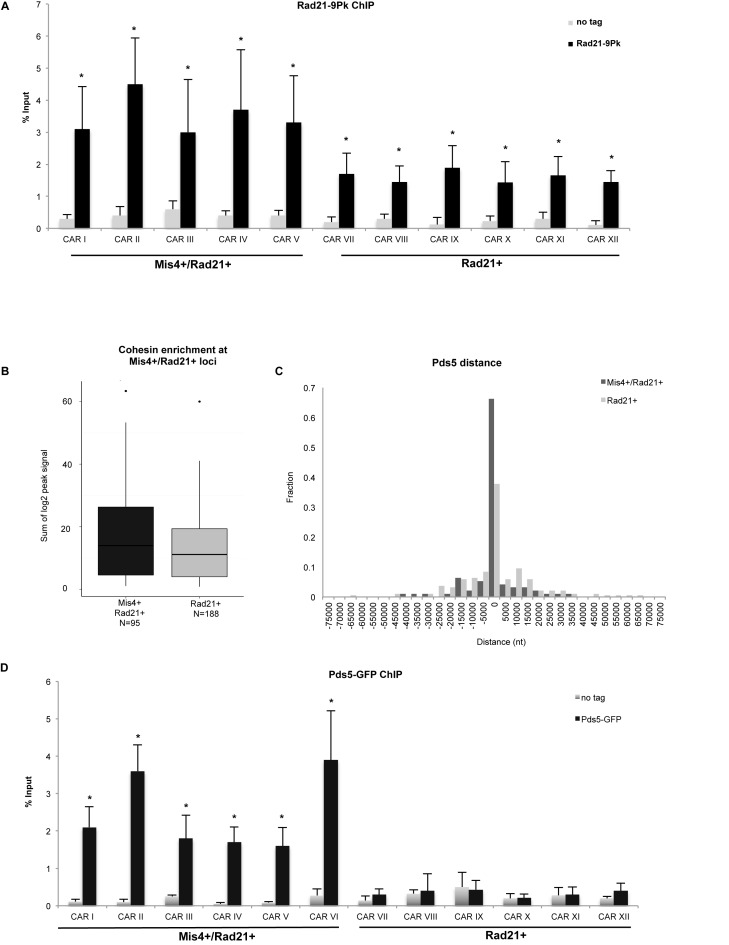
Cohesin and Pds5 co-localize at Mis4+/Rad21+ loci. (**A**) ChIP-qPCR analysis showing Rad21-Pk9 at selected CARs. No tag strain was used to assess the background levels. * *P* < 0.05, two-tailed, paired student's t-test for Rad21-Pk9 compared to background levels. Error bars represent SD, *n* = 3. (**B**) Cohesin enrichment at Mis4+/Rad21+ versus Rad21+ sites. Boxplots comparing sum of log2 Rad21 ChIP signal at Mis4+/Rad21+ versus Rad21+. *P* < 0.05, one-sided Wilcoxon Rank Sum test. (**C**) Pds5 enrichment at Mis4+/Rad21+ versus Rad21+ sites. Bar graph shows distances between Pds5 peak margins at Mis4+/Rad21+ and Rad21+ sites in 5000 nt bins. An overlap between intervals spanned by a Mis4+/Rad21+ and Pds5 peak gives a distance of 0. (**D**) ChIP-qPCR analysis showing Pds5-GFP at selected CARs. No tag strain was used to assess the background levels. *P < 0.05, two-tailed, paired Student's t*-*test for Pds5-GFP compared to background levels. Error bars represent SD, *n* = 3.

Although cohesin levels across the *S. pombe* genome seem to be similar, our DNA FISH analysis shows functional differences between Mis4+/Rad21+ and Rad21+ cohesin sub-sets. Therefore, we performed ChIP-qPCR analysis of Rad21-9Pk at all CARs and we detect significant (*P* < 0.05) enrichment of Rad21 over the background (no tag control) at all tested CARs (Figure 2A). Interestingly, genome wide analysis shows significant cohesin enrichment (*P* < 0.05) at Mis4+/Rad21+ sites in comparison to Rad21+ only (Figure 2B), suggesting that functional differences between Mis4+/Rad21+ and Rad21+ sites may reflect stoichiometric variation in cohesin levels along the chromosomal arms.

Pds5 is a cohesin associated factor essential for cohesion maintenance and *de novo* Psm3 acetylation (43–45). To test whether the observed differential cohesion at Mis4+/Rad21+ loci could arise due to Pds5 enrichment, we employed analysis of Pds5 ChIP-chip peaks and show that it significantly overlaps with Mis4+/Rad21+ loci (*P* < 10^−5^, Supplementary Figure S4A). Similarly, metagene analysis confirms that Pds5 peaks are closer to Mis4+/Rad21+ loci than to Rad21+ loci (Figure 2C). Next, we employed ChIP-qPCR to test the Pds5 enrichment at selected CARs. We observed significant (*P* < 0.05) Pds5 enrichment at Mis4+/Rad21+ CARs. Rad21+ loci showed Pds5 close to background levels (Figure 2D).

These data suggest that cohesin levels are generally higher at its loading sites, where they overlap with Pds5.

### Mis4+/Rad21+ sites associate with acetyltransferase Eso1

**Figure 3.**
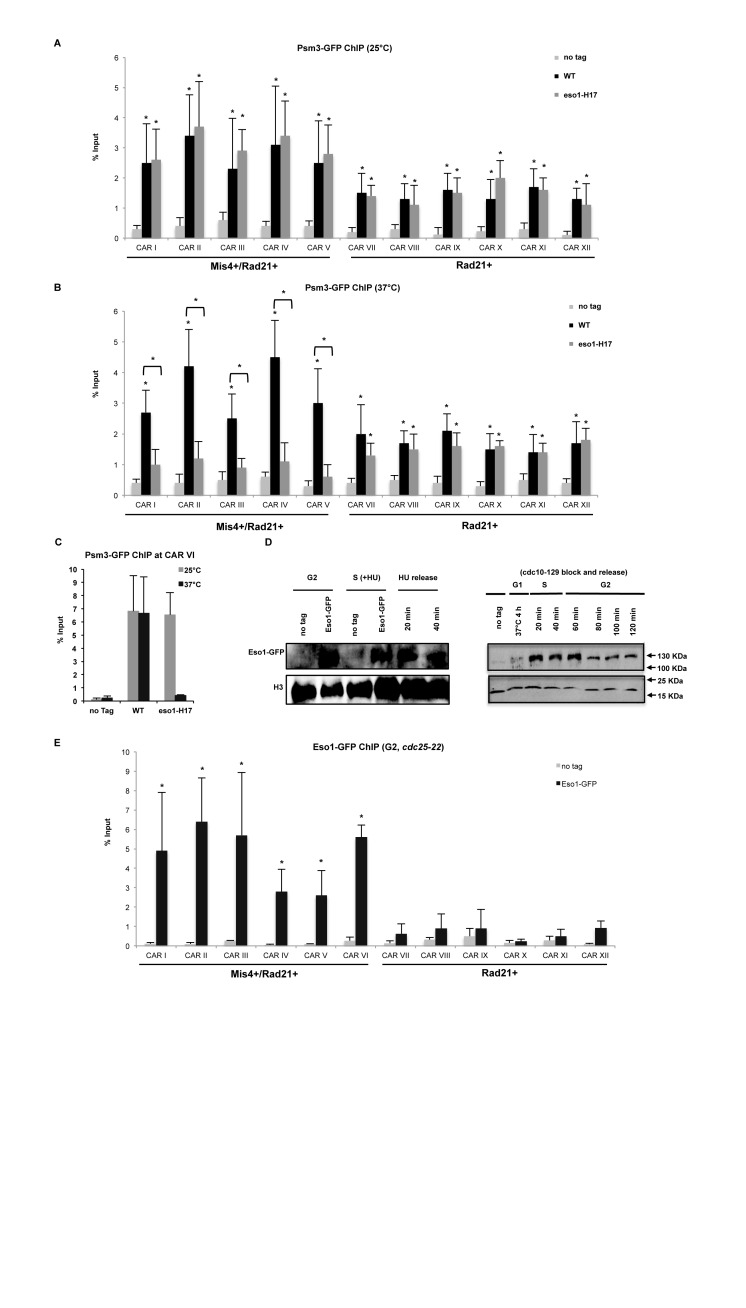
Cohesin at Mis4+/Rad21+ loci is stabilized by Eso1. (**A**) ChIP-qPCR analysis showing Psm3-GFP enrichment at selected CARs in WT and *eso1-H17* cells at 25°C. No tag strain was used to assess the background levels. *P < 0.05, two-tailed, paired Student's *t*-test for Psm3-GFP compared to background levels. Error bars represent SD, *n* = 3. (**B**) ChIP-qPCR analysis showing Psm3-GFP enrichment at selected CARs in WT and *eso1-H17* cells at 37°C. No tag strain was used to assess the background levels. *P < 0.05, two-tailed, paired Student's *t*-test for Psm3-GFP compared to background levels. Error bars represent SD, *n* = 3. (**C**) ChIP-qPCR analysis showing Psm3-GFP enrichment at centromeric *dg* repeat (CAR VI) in WT and *eso1-H17* cells at 25 and 37°C. Error bars represent SD, *n* = 3. (**D**) Left panel: western blot showing Eso1-GFP protein levels in G2/S phase cells. Whole cell extracts were prepared from cycling cells (G2), cells arrested in early S-phase with Hydroxyurea (HU) and subsequently released into G2 at indicated time points. No tag is a control for anti-GFP antibody specificity and Histone H3 is used as a loading control. Right panel: western blot showing Eso1-GFP protein levels in G1 synchronized cells through to G2 at respective time points. H3 is used as a control. (**E**) ChIP-qPCR analysis showing Eso1-GFP enrichment at selected CARs in G2 synchronized cells (*cdc25-22*, 37°C, 4 h). No tag strain was used to assess the background levels. * *P* < 0.05, two-tailed, paired Student's *t*-test for Eso1-GFP compared to background levels. Error bars represent SD, *n* = 3.

Psm3 acetylation by Eso1 is a hallmark of cohesion establishment. To test whether Eso1 stabilizes cohesin association with chromatin at CARs I-VI, we performed ChIP-qPCR to detect Psm3 levels at selected CARs in WT and *eso1-H17* mutant. Cells were synchronized in early S phase (HU 15mM, 2 h) at 25°C followed by a shift to 37°C for 2 h to inactivate Eso1. Cell cycle progression was monitored by FACS analysis (Supplementary Figure S3D). Cohesin enrichment at all tested CARs was observed in WT and *eso1-H17* cells at the permissive temperature (Figure 3A and C). However, Psm3-GFP levels were significantly (*P* < 0.05) decreased only at Mis4+/Rad21+ loci at the restrictive temperature (Figure 3B and C).

Interestingly, levels of the Eso1 orthologue in *S. cerevisiae*, Eco1, are regulated by proteasome degradation after S phase (46). To test whether Eso1 is similarly regulated in fission yeast, we compared Eso1 protein levels in G2 and S-phases. FACS analysis indicated S phase arrest after hydroxyurea treatment (Supplementary Figure S3E, 15 mM 2 h at 32°C) followed by progression to G2. Surprisingly, Eso1-GFP protein levels were similar between G2 and S phases (Figure 3D, left panel). We extended this analysis to G1 arrested cells using the *cdc10-129* mutant at 37°C, followed by release to S and G2 at 25°C. Cell cycle progression was monitored by FACS analysis (Supplementary Figure S3G). We do detect Eso1 protein levels throughout the cell cycle, although its levels are slightly decreased in G2 (Figure 3D, right panel).

Furthermore, we employed ChIP-qPCR and observed significant enrichment of Eso1 in G2 synchronized cells (*cdc25-22*, Supplementary Figure S3F) at CARs I–VI, but not at CARs VII–XII (Figure 3E).

These data together with FISH results suggest that Mis4+/Rad21+ loci are associated with Eso1 and Pds5 and represent cohesive sites on chromosomal arms.

### Mis4+/Rad21+ sites overlap with highly expressed RNAPII genes

**Figure 4.**
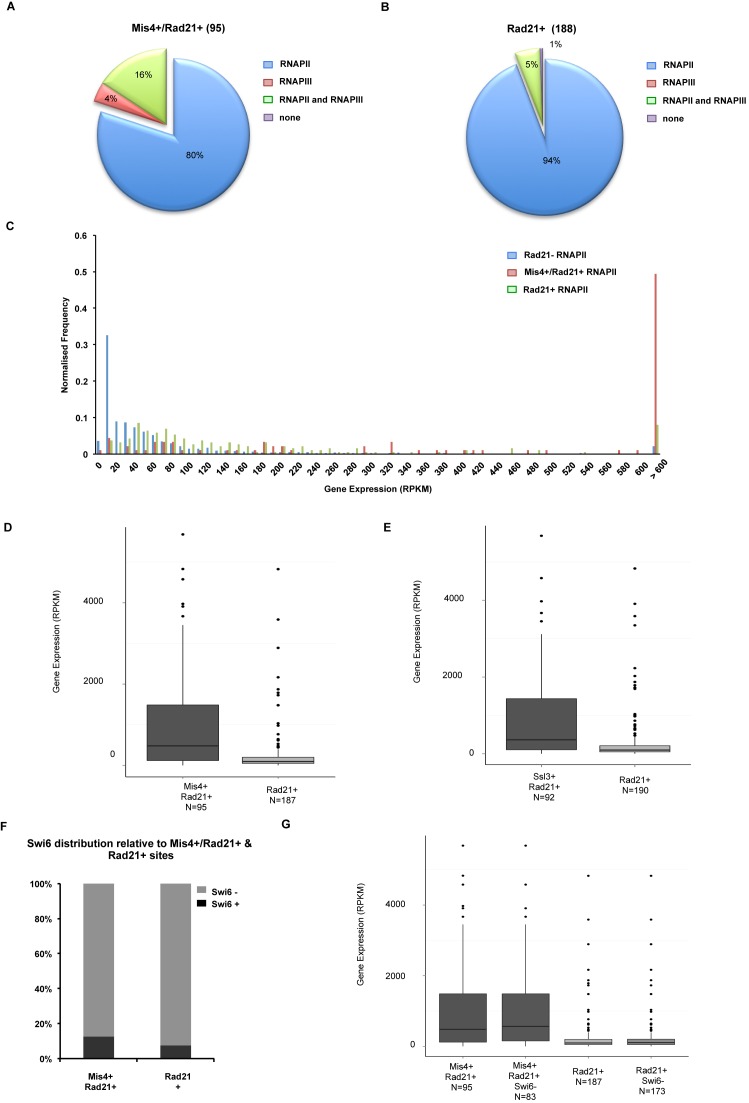
Cohesive sites overlap highly transcribed RNAPII regions. (**A**) Pie-chart showing overlap between Mis4+/Rad21+ loci and RNAPII genes, with a mere 4% overlap with only RNAPIII genes. (**B**) Pie-chart showing overlap between Rad21+ loci and RNAPII and RNAPIII genes. (**C**) Histogram showing the distribution of RNAPII genes versus gene expression in non-cohesin associated genes (blue bars, 1412 genes), cohesive (red bars, 91 genes) and non-cohesive loci (green bars, 187 genes). (**D**) Boxplots showing expression levels (Reads per Kilobase per Million; RPKM) of genes that overlap with Mis4+/Rad21+ and Rad21+ sites. *P* < 10^−9^, Wilcoxon Rank Sum test. (**E**) Boxplots showing expression level (reads per kilobase per million; RPKM) of genes that overlap with Ssl3+/Rad21+ and Rad21+ sites. *P* < 10^−7^, Wilcoxon Rank Sum test. (**F**) Graph showing Swi6 occupancy at Mis4+/Rad21+ and Rad21+ loci. (**G**) Boxplots showing Mis4+/Rad21+ sites overlap with highly expressed genes in comparison to Rad21+ sites, including or excluding Swi6 loci. Changes in gene expression of Mis4+/Rad21+/Swi6− loci compared to Mis4+/Rad21+/Swi6+ loci and Rad21+/Swi6− loci compared to Rad21+/Swi6+ loci are not significant. Changes in gene expression of Mis4+/Rad21+/Swi6− loci compared to Rad21+/Swi6− loci and Mis4+/Rad21+/Swi6+ loci compared to Rad21+/Swi6+ loci are significant (*P* < 10^−9^, Wilcoxon Rank Sum test).

Previous analysis demonstrates that Mis4-Ssl3 binding sites on chromosome 2 overlap with highly transcribed RNAPII and RNAPIII genes (29). To delineate this further, we extracted RNAPIII loci from PomBase based on tRNA and 5S rRNA search and assumed all other genes to be RNAPII transcribed. Subsequently, we overlapped Mis4+/Rad21+ and Rad21+ peaks with RNAPIII and RNAPII genes. Interestingly, ∼80% Mis4+/Rad21+ peaks overlap RNAPII genes (Figure 4A), while 4% overlap RNAPIII genes and additional 16% overlap both. Similarly, ∼94% Rad21+ peaks overlap RNAPII genes, with only 1% present at RNAPIII loci (Figure 4B). The slight preference (3%) of Mis4+/Rad21+ versus Rad21+ sites for RNAPIII genes is supported by their proximity to RNAP III transcription factor Sfc6 (Supplementary Figure S4B and C). It should be noted that RNAPI transcribed ribosomal RNA genes were excluded, as all rRNA loci in *S. pombe* are located on chromosome III.

Next, we examined the expression of all RNAPII genes (47) and combined it with Rad21-/RNAPII, Mis4+/Rad21+/RNAPII and Rad21+/RNAPII sites. There is a clear association of Mis4+/Rad21+ sites with highly expressed genes (Reads per kilobase per million, RPKM>600), while the majority of all other RNAPII sites are aggregated at lowly expressed genes (Figure 4C). Despite >90% overlap of Mis4+/Rad21+and Rad21+ peaks with RNAPII, only Mis4+/Rad21+ sites show a significant overlap with highly expressed RNAPII genes (median RPKM > 350, *P* < 10^−7^, Figure 4D, Mis4+/Rad21+ and Figure 4E, Ssl3+/Rad21+).

Recent reports demonstrate the propensity of misleading ChIP signals observed at highly expressed, open chromatin hyper-ChIPable regions (40). However, it should be noted that in that study, formaldehyde cross-linking was performed for 60 min, while routine ChIP protocols (as used in our study) cross-link for 30 min. Naturally, higher cross-linking times could generate artifacts. However, to eliminate false ChIP signals, we analyzed the distribution of heterochromatin binding protein Swi6 (48) and observed that ∼85% of Mis4+/Rad21+ and ∼90% of Rad21+ sites lack Swi6, in line with overlap with highly expressed genes and not heterochromatin regions (Figure 4F). Furthermore, both Mis4+/Rad21+ and Rad21+ sites exhibit significant differences in gene expression, with or without inclusion of Swi6 peaks (Figure 4G).

These data confirm that the association of Mis4+/Rad21+ sites with highly transcribed genes is a biologically valid effect. The correlation between cohesin binding sites and RNAPII regions suggests a possible interplay between RNAPII and cohesin loading/cohesion establishment.

### Transcription inhibition reduces chromatin bound Mis4 and cohesin on chromosomal arms

**Figure 5.**
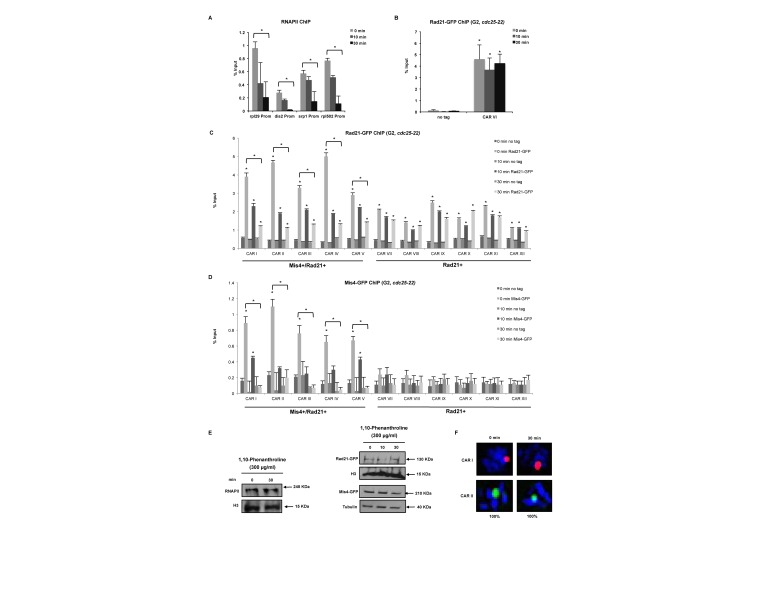
Transcription inhibition reduces chromatin association of cohesin proteins. (**A**) ChIP-qPCR analysis showing RNAPII occupancy after 1,10-phenanthroline treatment (300 μg/ml, 10 and 30 min) at selected promoters. *P < 0.05, two-tailed, paired Student's *t*-test between 0 and 30 min. Error bars represent SD, *n* = 3. (**B**) ChIP-qPCR analysis showing Rad21-GFP enrichment at CAR VI after 1,10-phenanthroline treatment (300 μg/ml, 10 and 30 min) in G2 synchronized cells (*cdc25-22*, 37°C 4 h). No tag strain was used to assess the background levels. *P < 0.05, two-tailed, paired Student's *t*-test for Rad21-GFP compared to background levels. Error bars represent SD, *n* = 3. (**C**) ChIP-qPCR analysis as in (**B**) at selected CARs. (**D**) ChIP-qPCR analysis as in (**C**) showing Mis4-GFP enrichment at selected CARs (**E**) Western blot showing RNAPII, Rad21-GFP, Mis4-GFP, H3 and tubulin protein levels after transcription inhibition using 1,10-phenanthroline (300 μg/ml, 30 min). Total RNAPII was probed with 8WG16 (ab817), Rad21 and Mis4 with anti-GFP (ab290) and histone H3 with anti-H3 (ab1791) antibody. (**F**) DNA-FISH analysis of CARs I and II after 1,10-phenanthroline treatment (300 μg/ml, 30 min).

To test whether association of Mis4+/Rad21+ loci with highly transcribed genes might have a biological relevance; we employed G2 synchronized cells treated with the transcription inhibitor 1,10-phenanthroline. Transcription inhibition was confirmed by a reduction in RNAPII levels at four tested RNAPII promoters after 10 and 30 min of treatment with 1,10-phenanthroline (Figure 5A). Next, we assessed cohesin levels at centromeres. Rad21 signals remained unchanged after transcription inhibition (CAR VI, Figure 5B), presumably because cohesion establishment at centromeres occurs via the RNAi dependent heterochromatin pathway (49). Furthermore, Rad21 levels were significantly (*P* < 0.05) reduced after 1,10-phenanthroline treatment at CARs I-V but not at CARs VII–XII (Figure 5C). We also performed Mis4 ChIP-qPCR and we detect significant (*P* < 0.05) Mis4 levels only at Mis4+/Rad21+ loci. Transcription inhibition resulted in reduced levels of Mis4 at CARs I–V, similar to Rad21 (Figure 5D). To test whether decreased levels of Rad21 and Mis4 at Mis4+/Rad21+ loci are not a result of reduced protein levels after transcription inhibition, we performed western blot analysis and show that Rad21 and Mis4 protein levels remained unchanged after treatment with 1,10-phenanthroline (Figure 5E).

Finally, we performed FISH experiment in 1,10-phenantroline treated cells, wherein we do not observe any separation of sister chromatids (Figure 5F). This was an expected result, as cohesion established during S-phase would persist throughout G2 phase. Decreased levels of cohesin after transcription inhibition would most probably have no effect on already established cohesion.

### Transcription induction facilitates cohesion establishment at heat shock genes.

**Figure 6.**
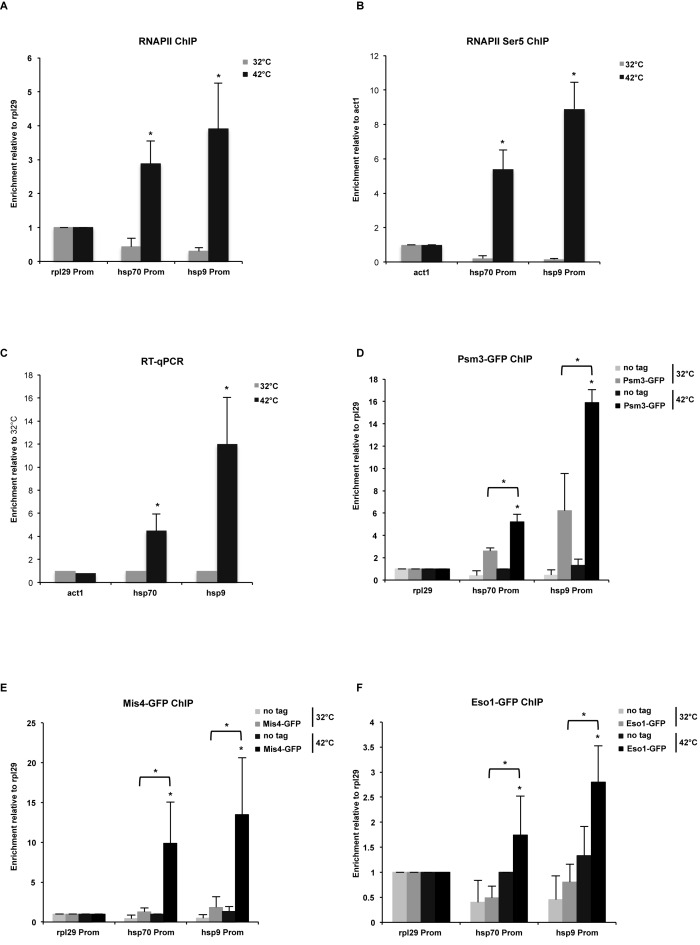
Transcription induction mediates cohesin proteins recruitment to chromatin. (**A**) ChIP-qPCR analysis showing RNAPII enrichment at selected promoters after heat shock at 42°C, 30 min. Values are normalized to *rpl29*. Error bars represent SD, *n* = 3. *P<0.05, one-tailed, paired Student's *t*-test comparing 42 to 32°C values. (**B**) ChIP-qPCR analysis showing enrichment of RNAPII phosphorylated at Ser5 at selected promoters after heat shock at 42°C, 30 min. Values are normalized to *act1*. Error bars represent SD, n = 3. * *P*<0.05, two-tailed, paired student's t-test comparing 42°C to 32°C values. (**C**) RT-qPCR showing *act1, hsp70* and *hsp9* mRNA levels after heat shock. Error bars represent SD, *n* = 3. Values are normalized to 32°C signals. *P < 0.05, two-tailed, paired Student's *t*-test comparing 42 to 32°C values. (**D**) ChIP-qPCR analysis showing Psm3-GFP enrichment at *hsp70* and *hsp9* promoters after shifting from 32 to 42°C, 30 min. Values are normalized to *rpl29*. Error bars represent SD, *n* = 3. *P < 0.05, one-tailed, paired Student's *t*-test comparing 42 to 32°C values and GFP signal to no tag control. (**E**) ChIP-qPCR analysis showing Mis4-GFP enrichment at *hsp70* and *hsp9* promoters after shifting from 32 to 42°C, 30 min. Values are normalized to *rpl29*. Error bars represent SD, *n* = 3. *P < 0.05, one-tailed, paired Student's *t*-test comparing 42 to 32°C values and GFP signal to no tag control. (**F**) ChIP-qPCR analysis showing Eso1-GFP enrichment at *hsp70* and *hsp9* promoters after shifting from 32 to 42°C, 30 min. Values are normalized to *rpl29*. Error bars represent SD, *n* = 3. *P < 0.05, one-tailed, paired Student's *t*-test comparing 42 to 32°C values and GFP signal to no tag control.

To further validate the role of RNAPII in cohesion establishment, we employed the heat shock response *(hsp)* gene loci and assessed chromatin occupancy of Mis4 and Psm3 at *hsp* promoters. Transcription was rapidly induced at *hsp70* and *hsp9* genes, as we detect increased RNAPII levels at these promoters after heat shock (Figure 6A). To test that RNAPII is not only pausing at *hsp* promoters but is actively engaged in transcription, we performed ChIP-qPCR and show increased levels of RNAPII phosphorylated at the Serine 5 residue (Ser5), which is a mark of transcriptional activity (Figure 6B). Furthermore, we confirmed transcription of *hsp* genes by RT-qPCR (Figure 6C). Interestingly, transcription induction resulted in enrichment of Psm3 (Figure 6D), Mis4 (Figure 6E) and Eso1 (Figure 6F) at *hsp70* and *hsp9* promoters after heat shock. To test, whether the recruitment of cohesin proteins to chromatin is RNA dependent, we performed Psm3 and Mis4 ChIP-qPCR with RNAse A/T treatment and observed the same level of their enrichment at *hsp* genes after heat shock (Supplementary Figure S5A and B). These data suggest that increased levels of transcription rather than RNA could mediate recruitment of cohesin proteins to chromatin.

**Figure 7.**
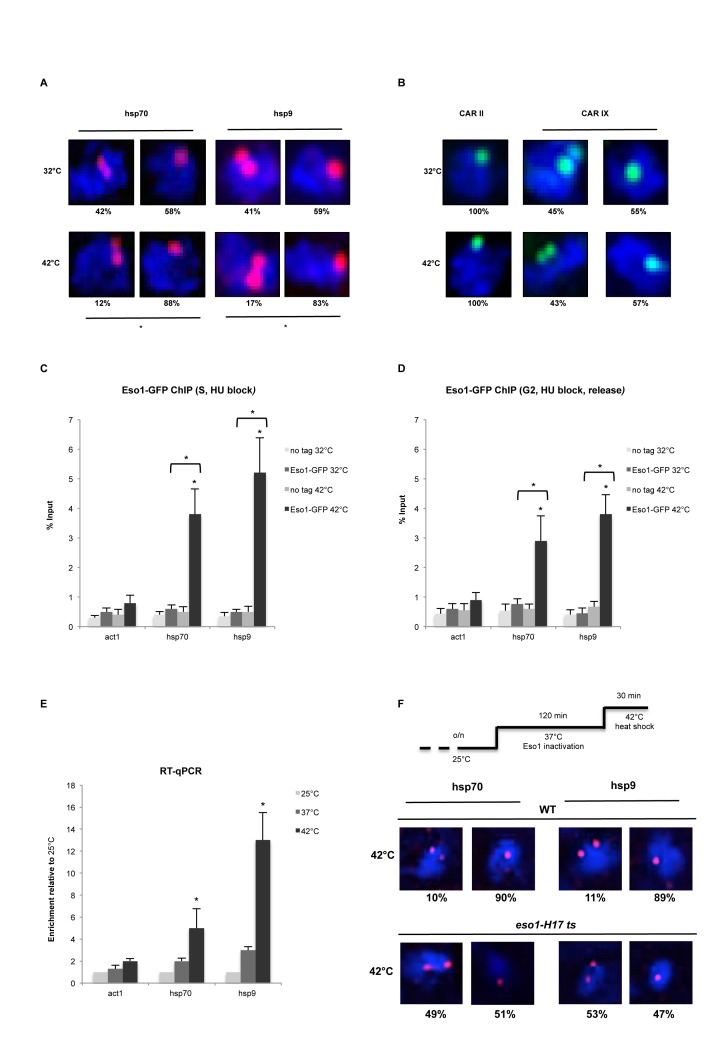
Transcription induction mediates cohesion establishment. (**A**) DNA FISH analysis of *hsp70* and *hsp9* loci. FISH probes (red dots, Alexa-555) are shown inside DAPI stained nucleus (blue). *P < 0.05. two-tailed, paired Student's *t*-test. *n* = 100 cells, two biological repeats. (**B**) DNA FISH analysis of CAR II and CAR IX used as controls for FISH after heat shock induction. 100% cohesion observed at CAR II between 32 and 42°C (left panel) and ±50% cohesion at CAR IX (same as in Figure 1B and C, respectively). *n* = 100 cells, two biological repeats. (**C**) ChIP-qPCR analysis showing Eso1-GFP enrichment at *hsp70* and *hsp9* loci in S synchronized cells (Hydroxyurea) after heat shock. No tag strain was used to assess the background levels. Error bars represent SD, *n* = 3. *P < 0.05, two-tailed, paired Student's *t*-test comparing 42 to 32°C values and GFP signal to no tag control. (**D**) ChIP-qPCR analysis showing Eso1-GFP enrichment at *hsp70* and *hsp9* loci in G2 synchronized cells (Hydroxyurea block and release) after heat shock. No tag strain was used to assess the background levels. Error bars represent SD, *n* = 3. *P < 0.05, two-tailed, paired Student's *t*-test comparing 42 to 32°C values and GFP signal to no tag control. (**E**) RT-qPCR showing *act1, hsp70* and *hsp9* mRNA levels at 25, 37 and 42°C. Error bars represent SD, *n* = 3. *P < 0.05, two-tailed, paired Student's *t*-test comparing 42 to 25°C values. (**F**) DNA FISH analysis of *hsp70* and *hsp9* loci in WT and *eso1-H17* cells. Cells were grown o/n at 25°C. Eso1 was inactivated at 37°C for 2 h, followed by heat shock at 42°C.

Next, we employed DNA FISH analysis to test whether *de novo* recruitment of cohesin, Mis4 and Eso1 in G2 is sufficient to establish stable cohesion. The *hsp* loci are normally Rad21+ and at 32°C display single dots in ∼50% of cells (Figure 7A). Surprisingly, heat shock induction led to significant (*P*<0.05) increase in cells with *hsp70* (58–88%) and *hsp9* (59–83%) single dots, indicating stable cohesion at these loci (Figure 7A). As a control, we analyzed cohesion at Mis4+/Rad21+ and Rad21+ sites (Figure 7B, CARs II and IX), which resulted in cells with 100% and ∼50% cohesion, respectively and were unaffected by heat shock conditions.

We showed that Eso1 is recruited to *hsp* genes after heat shock (Figure 6F). In normal conditions cohesion is established during replication in S phase, when Eso1 protein levels are also highest. We employed Eso1 ChIP-qPCR in S or G2 phase synchronized cells prior to heat shock. Eso1 enrichment at *hsp* genes was moderately higher in S phase than in G2 phase, correlating with its protein levels (Figures 3D and 7C and D).

Finally, we tested whether *de novo* established cohesion at *hsp* genes is dependent on Eso1. We employed WT and *eso1-H17* cells at restrictive temperature followed by a heat shock. RT-qPCR analysis showed only a mild effect of 37°C temperature, which is necessary for Eso1 inactivation, on expression of the heat shock genes (Figure 7E). FISH performed on WT cells confirmed a significant (*P* < 0.05) increase in the number of cells with single dots at *hsp70* (90% of cells) and *hsp9* (89% of cells) after heat shock (Figure 7F). In contrast, inactivation of Eso1 resulted in no *de novo* cohesion at *hsp* loci after heat shock.

These results suggest that increased transcription can lead to functional cohesion establishment, which is Eso1 dependent.

### RNAPII transcription affects chromatin association of cohesion proteins in human cells.

To generalize our results, we cross-compared existing ChIP-Seq data for cohesin subunit SMC1 and RNAPII from human cells (39). ChIP-Seq data was extracted and analyzed as described previously (39), except the reads were mapped against Human_UCSC_GRCh37/hg19 assembly. Interestingly, SMC1 was present within ±1 kb of RNAPII (Supplementary Figure S6A) suggesting that cohesin is in close physical proximity to RNAPII. Furthermore, SMC1 overlapping with cohesin loader NIPBL (human orthologue of Mis4) also associates with highly transcribed genes in human cells (Supplementary Figure S6B).

To further substantiate the interplay between cohesin and RNAPII, we assessed chromatin occupancy of cohesin subunits RAD21 and SMC3 in human HEK293T cells after inhibition of RNAPII by α-amanitin (2 ug/ml, 36 h) (34). We observed a >95% drop in RNAPII occupancy at the *GAPDH* transcription start site (Supplementary Figure S6C, left graph). This coincided with a significant reduction in RAD21 (Supplementary Figure S6C, middle graph, up to 75%) and SMC3 (Supplementary Figure S6C, right graph, up to 82%). Similarly, a decrease in RNAPII at c-*MYC* TSS (Supplementary Figure S6D, left graph) matched reduced RAD21 (Supplementary Figure S6D, middle graph) and SMC3 signals at the TSS (Supplementary Figure S6D, right graph). While RAD21 dropped at the CTCF+/cohesin+ site upstream (up probe) of c*-MYC* TSS, SMC3 levels were unperturbed, suggesting that RNAPII mediates chromosomal association of cohesin at highly transcribed genes. Secondary effects due to α-amanitin were ruled out as only RNAPII was degraded (Supplementary Figure S6F), while RAD21, SMC3 and tubulin were unaffected (Supplementary Figure S6F).

Next, we assessed cohesin occupancy after transcription induction in human cells, focussing on the well-characterized *ERBB2* gene that is actively transcribed in breast cancer derived ZR-75-1 cells, but silent in MCF-7 (50). Coincident with RNAPII enrichment (Supplementary Figure S6E, left graph), RAD21 (Supplementary Figure S6E, middle graph) and SMC3 (Supplementary Figure S6E, right graph) also showed a significant increase at the *ERBB2* gene promoter in ZR-75-1 cells compared to MCF7 cells. *HPRT* was used as a negative control and protein levels were consistent between ZR-75-1 and MCF-7 cells (Supplementary Figure S6G). These results suggest that RNAPII facilitates cohesin association with chromatin in human cells.

### Cohesin and its loader co-localize with highly expressed RNAPII genes in *S. cerevisiae*

To extend our findings further, we re-analyzed previously published *S. cerevisiae* ChIP-Seq data (11). First, we observed 92.2% overlap between cohesin and RNAPII loci, in sharp contrast to <0.6% overlap with RNAPIII only (Supplementary Figure S7A), suggesting that most cohesin in budding yeast also associates with RNAPII. Next, cohesin peaks that colocalize with cohesin loader proteins (Scc2+/Scc1+ or Scc4+/Scc1+) showed significant overlap with highly expressed RNAPII genes in contrast to Scc1+ peaks alone (Supplementary Figure S7B, C). Likewise, Scc2+/Smc3+ or Scc4+/Smc3+ overlap highly expressed RNAPII genes in comparison to Smc3+ peaks alone (Supplementary Figure S7D, E).

Additionally, ∼80% Scc2+/Scc1+, ∼85% Scc4+/Scc1+ and ∼95% Scc1+ sites (Supplementary Figure S8A) were devoid of the 238 annotated highly expressed, open chromatin hyper-ChIPable regions (40). Similarly, ∼85% Scc2+/Smc3+, ∼85% Scc4+/Smc3+ and ∼95% Smc3+ sites (Supplementary Figure S8B) were also devoid of hyper-ChIPable regions. Furthermore, a comparison of gene expression between Scc2+/Scc1+ versus Scc1+ (Supplementary Figure S8C) and Scc4+/Scc1+ versus Scc1+ (Supplementary Figure S8D) sites, with or without inclusion of hyper-ChIPable regions was similar. Likewise, gene expression differences between Scc2+/Smc3+ versus Smc3+ (Supplementary Figure S8E) and Scc4+/Smc3+ versus Smc3+ (Supplementary Figure S8F) were independent of any hyper-ChIPable artifacts.

Overall our results have identified cohesive loci on chromosomal arms in fission yeast. At these sites cohesin co-localizes with its loading complex, Pds5 and Eso1. Furthermore, we have identified RNAPII transcription as a potential functional determinant for cohesin loading and consequent cohesion establishment.

## DISCUSSION

The multi-subunit cohesin complex is highly conserved across all eukaryotes and executes cohesion, transcription and repair/recombination functions (51). Interestingly, cohesin occupancy along chromosomes is highly variable among different organisms (26–30,52). In metazoans cohesin co-localizes at sites of active transcription and insulator elements. In budding yeast, cohesin accumulates at intergenic regions away from loading sites (26,28). In fission yeast, cohesin is divided into sub-sets, (i) cohesin at its loading sites Mis4+/Rad21+, and (ii) cohesin on its own Rad21+ only (29,33).

We demonstrate in *S. pombe* that chromatin-associated cohesin results in functional cohesion of sister chromatids only at the sites of loading (Figure 1). Recent *in vitro* experiments showed that cohesin exhibits DNA affinity but its loading onto DNA substrates is greatly facilitated by its loader Mis4-Ssl3 (12). Our results show that cohesin association with its loader is indeed required for stable cohesion *in vivo*.

The cohesin loading complex facilitates association of cohesin with DNA during late G1 in *S. cerevisiae* and telophase in vertebrates (8) but cohesion is established only during S phase (53,54), concomitant with Psm3 acetylation. Additionally, in *S. cerevisiae* Eco1 (Eso1 in *S. pombe*) levels are regulated via Cdk1 phosphorylation and proteasome degradation in G2 and M, with maximal expression in S phase (46). We observe significant Eso1 enrichment at cohesive sites (Figure 3E), supported by the presence of Eso1 protein (Figure 3D) in G2 and S phases in *S. pombe*. This suggests that Eso1 could be recruited to cohesive sites in G2, similar to *de novo* Eco1 dependent cohesion establishment during DNA damage in budding yeast (55,56). Interestingly, Mis4 and Ssl3 are HEAT- and TPR-repeat containing proteins respectively, inherently capable of several protein-protein interactions (57). Mis4-Ssl3 might play an yet uncharacterized role in chromatin recruitment of Eso1.

Genome wide analysis shows that cohesive sites are close to Pds5 sites on chromatin (Figure 2B). In budding yeast and *Xenopus*, Pds5 is crucial for cohesin release, partly by recruiting Wpl1 (17,58). In contrast, Pds5 is also essential for *de novo* Psm3 acetylation (43–44,59) and protects cohesin from Hos1 mediated deacetylation during G2 and M phases in yeast (44). We detect significant levels of Pds5 at the cohesin loading sites, exhibiting stable cohesion (Figure 2D).

Next, we show that cohesive sites significantly overlap with highly expressed RNAPII transcribed genes (Figure 4) suggesting a possible interplay between cohesion and transcription. We observed a dramatic reduction in chromatin bound cohesin after transcription inhibition (Figure 5) using 1,10-phenanthroline, a commonly used chemical with phenotype similar to an RNAPII *ts* mutant (60). While much evidence argues for a function of cohesin in the gene expression regulation, here we show that chromosomal association of cohesin and Mis4 depends on RNAPII transcription. Furthermore, a recent study shows direct interaction between RNAPII and cohesin subunit SA1 (homologue of Psc3 in *S. pombe*) in mammalian cells (61). Once stable cohesion is established (S-phase) it persists until anaphase, wherein only a fraction of cohesin is cleaved. However, we observe a reduction in chromatin bound cohesin upon transcription inhibition in G2, suggesting that RNAPII transcription is likely to facilitate cohesin loading throughout the cell cycle.

Next, we show that induction of heat shock transcription leads to recruitment of Psm3, Mis4 and Eso1 at heat shock genes resulting in significant and functional *de novo* cohesion establishment in G2 (Figures 6 and 7). Recent evidence in budding yeast implicates the RSC chromatin-remodeling complex in establishing nucleosome free regions and facilitating chromatin association of Scc2-Scc4 and cohesin (62). Whether RSC complex performs a similar function in *S. pombe* is unknown. A direct interaction between cohesin and RNAPII (61) suggests that chromatin association of cohesin could be facilitated by chromatin remodeling but is mediated via RNAPII.

To summarize, we propose a model (Supplementary Figure S9) for cohesion establishment on chromosomal arms in fission yeast. *De novo* synthesis of Rad21 in G1 (Rad21 is cleaved at anaphase onset) coincides with the reassembly of the cohesin ring. RNAPII is likely to mediate recruitment of cohesin through Mis4-Ssl3 to highly transcribed genes throughout the cell cycle. The open chromatin conformation at active genes provides an ambient platform for replication fork assembly and replication initiation. Consequently, the concurrence between newly replicated sister chromatids and the cohesion machinery promotes establishment of stable cohesion at these sites. In contrast, poorly expressed genes fail to recruit Pds5, Mis4 and Eso1. As a result, cohesin at non-cohesive sites maintains transient association and dissociation kinetics and fails to establish stable cohesion. Perhaps these non-cohesive sites could serve as a platform for cohesion establishment in events of DNA damage or stress response. We propose that RNAPII transcription is a key player for cohesin loading and consequently cohesion establishment on chromosome arms in eukaryotes.

## Supplementary Material

SUPPLEMENTARY DATA
