# Supplementary material for: Transcription facilitates sister chromatid cohesion on chromosomal arms
Source: Nucleic Acids Res. 2016 Apr 15;44(14):6676–92. doi: 10.1093/nar/gkw252 (PMC5001582; doi:10.1093/nar/gkw252)
Supplement: SUPPLEMENTARY DATA [file supp_44_14_6676__index.html]

Transcription facilitates sister chromatid cohesion on chromosomal arms — Transcription facilitates sister chromatid cohesion on chromosomal arms — SUPPLEMENTARY DATA 

# Transcription facilitates sister chromatid cohesion on chromosomal arms

## SUPPLEMENTARY DATA

- SUPPLEMENTARY DATA
- SUPPLEMENTARY DATA
- SUPPLEMENTARY DATA
- SUPPLEMENTARY DATA
- SUPPLEMENTARY DATA
- SUPPLEMENTARY DATA
- SUPPLEMENTARY DATA
- SUPPLEMENTARY DATA
