# Supplementary material for: Transcription facilitates sister chromatid cohesion on chromosomal arms
Source: Nucleic Acids Res. 2016 Apr 15;44(14):6676–92. doi: 10.1093/nar/gkw252 (PMC5001582; doi:10.1093/nar/gkw252)
Supplement: SUPPLEMENTARY DATA [file supp_gkw252_nar-00155-v-2016-File003.doc]

**Supplementary Information**

**Supplementary File 1**: Binding profiles for Rad21, Mis4, Ssl3, Pds5 and Psc3 in *S. pombe* on chromosome 2.

**Supplementary File 2**: Comparison of log2 and linear scale of CARs I-XII.

**Table S1**:

| **CAR** | **Cohesin peak** | **Mis4 peak** |
| --- | --- | --- |
| **I** | **201** | **106** |
| **II** | **214** | **114** |
| **III** | **208** | **110,111** |
| **IV** | **188** | **102** |
| **V** | **44** | **18** |
| **VI** | **cendg** | **cendg** |
| **VII** | **205** | **-** |
| **VIII** | **66** | **-** |
| **IX** | **52** | **-** |
| **X** | **241** | **-** |
| **XI** | **57** | **-** |
| **XII** | **196** |  |

**Table S1:** List of Rad21 and Mis4 peaks used in this study. CARs I-VI represent Mis4+/Rad21+ sites, while CARs VII-XII represent Rad21+ sites only. The numbers for cohesin and Mis4 represent the peak number in the respective datasets. For a full list of peaks please see supplemental information 1.

|  | **position of CAR** | **nearest Mis4+ left** | **nearest Mis4+ right** |
| --- | --- | --- | --- |
| **CAR VII** | **3178376** | **3130000** | **3230000** |
| **CAR VIII** | **1089126** | **1000000** | **1150000** |
| **CAR IX** | **850001** | **800000** | **900000** |
| **CAR X** | **3720876** | **3610000** | **3770000** |
| **CAR XI** | **921751** | **900000** | **100000** |
| **CAR XII** | **3048376** | **2980000** | **3110000** |

**Table S2:** Position of CARs VII-XII in respect to closest Mis4+ peak on each side

|  | **Strain** | **Reference** |
| --- | --- | --- |
| 3385 | h- leu1-32 mis4-GFP-hygMX6 ssl3-Pk9-kanMX6 | Schmidt et al, 2009 |
|  | 972h- | Gullerova and Proudfoot, 2008 |
| 2730 | h+ mis4-367 | Bernard et al., 2006 |
|  | h+ rad21-K1ts-ura4+ leu1-32 ura4D18 ade6-210 | Gullerova and Proudfoot, 2008; Tatebayashi et al., 1998 |
| Y3127 | h- ura4-D18 rad21-3xeGFP-kanMX6 mis4-Pk9-kanMX6 | Schmidt et al., 2009 |
| 3678 | h- ura4 psm3-GFP-natR | Vaur et al., 2012 |
| 3925 | h- ura4 eso1-H17 psm3-GFP-natR | Feytout et al., 2011 |
|  | h- leu1 mis4-GFP-LEU2+ | Gullerova and Proudfoot, 2008 |
| 436 | h+ cdc25-22 | Norbury’s lab |
| 187 | h+ cdc10-129 leu1-32 | Norbury’s lab |
|  | h- eso1-GFP kanR | Gift from Yanagida M (NBRP) |
| 2 | h- ura4-D18 rad21-3eGFP-kanMX6 mis4-Pk9-kanMX6 cdc25-22 | this study |
| 3 | cdc25-22 leu1-32 mis4-GFP-hygMX6 ssl3-Pk9-kanMX6 | this study |
| 1b | eso1-GFP kanR cdc25-22 | this study |
| 1a | eso1-GFP kanR cdc10-129 leu1-32 | this study |
| 405 | h+ cdc25-22 | Javerzat JP |
| 3202 | mis4-367 cdc25-22 | Javerzat JP |
| 3448 | h- cdc25-22 rad21-9PK-KanR | Javerzat JP |
| 363 | h-pds5-GFP:kanMX leu1-32 | Gift from C. Norbury |

**Table S3:** List of strains used in this study.

| **Locus** | **Primer sequence** |
| --- | --- |
| CAR I-F1 | TGGATCAACTTTTCAGCCTTG |
| CAR I-R1 | TATACTGGTCTCACGTTGGA |
| CAR I -F2 | AAGCACCTTACTCTCTCTTAC |
| CAR I -R2 | ATCATAAGCAGGTGCAGCTT |
| CAR I -F3 | GAGATAGCAACCTACTTCAAGA |
| CAR I -R3 | GCTGGTATTAATTAGTGGACATGC |
| CAR I -F4 | ATGATGAAGGCTATACTCCCTTGT |
| CAR I -R4 | TCATTAGGCGTCTTGTGACCTT |
|  |  |
| CAR III -F1 | ATTGTCGCGGTTTCTCAGTT |
| CAR III -R1 | TTGACGGTGTCAAGCGATTA |
| CAR III -F2 | GTGCCTCATCAGCAGTATAT |
| CAR III -R2 | CCATCATGTCTCTTACCATG |
| CAR III -F3 | CGTATTCATGACTGACTAAAGCTGT |
| CAR III -R3 | TAGAATCCCCGGTCTTCACA |
| CAR III -F4 | CAGGCTCTAATAATTGTAAGCGCA |
| CAR III -R4 | TACTTGTTCGTGGTTTAACCCA |
| CAR III -F5 | GGCACTCACCTTTCTAATTCGTA |
| CAR III -R5 | TATGCTGGTAAATGGCCTTTCT |
|  |  |
| CAR IV -F1 | GCCTAAGATTATGATCACCAACGTA |
| CAR IV -R1 | TTCGATGTATGAAGGTTGCC |
| CAR IV -F2 | CTCGTTGTTCCCAATTTGAAC |
| CAR IV -R2 | GCAGTTTCTCAATAGGAGATAGCC |
| CAR IV -F3 | CCTTCGAGTTTGCGTTATAAAC |
| CAR IV -R3 | GAAGAGAAGAAGAGTATGCCGAA |
| CAR IV -F4 | CTGCTTTATTGCCTTCTTCTGTTC |
| CAR IV -R4 | TGGAAGGAGAGATTTCTGCTCT |
|  |  |
| CAR II -F1 | GTGTACATCAATAGTGTTCGTTCCC |
| CAR II -R1 | ATTGGATGATGAGACTCTCCGT |
| CAR II -F2 | CAGCAACATGACCACCATAGATAT |
| CAR II -R2 | AAGAGAAATTAGGGACTCGAGTGTA |
| CAR II -F3 | AGGAAGGTATGCGATCGGATA |
| CAR II -R3 | TGCTAGTTTAATACACAGGCGCT |
| CAR II -F4 | TTATCAGTATTAGATTCCCAGCGC |
| CAR II -R4 | TGGGCTAATGCAACATTCGT |
|  |  |
| CAR V -F1 | AACTTCAATCTAACGCCCATTC |
| CAR V -R1 | GGAACTTTCACGTACTCCTTTTCT |
| CAR V -F2 | GCTTCAGGCGTTACAGTACAA |
| CAR V -R2 | TTGACGTTGGTGTTTATTTGCC |
| CAR V -F3 | CCTACGATTACACCATCAGGATTA |
| CAR V -R3 | TCGAATAAGCTTTCTGCGCTT |
| CAR V -F4 | AGTTGACTGTCTCTCAAAGATGGAT |
| CAR V -R4 | CCGGAGATTTCACATTTAGACCT |
|  |  |
| CAR VII -F1 | TGCGTTTGAAGAAGTAAGGACT |
| CAR VII -R1 | CTCCATCAAAGTTGACAACAC |
| CAR VII -F2 | ACTTGATGTAGTTCTGTGAGGA |
| CAR VII -R2 | AGTCCAGGTGCTTTCATCTAA |
| CAR VII -F3 | GAAATCCCTCCAGGTAGATAT |
| CAR VII -R3 | ATTCAAGCACTGTGTGAACGA |
| CAR VII -F4 | GGCAACTATAGTTGTATTCGGA |
| CAR VII -R4 | CATGTTCAGCAACAGAAGAGTT |
|  |  |
| CAR VIII-F1 | CTGCCAACCTTTTGAGTCAACT |
| CAR VIII-R1 | CGTATAGCTTACTTGACTGCATAGCAT |
| CAR VIII -F2 | CTAGTCCAACTTACCAAATTCGCT |
| CAR VIII -R2 | GCGACCATTGTGAAAACTGA |
| CAR VIII -F3 | GTTTCGTCAGTTTTCACAATGGT |
| CAR VIII -R3 | AGCTTTGAATGCAGCAGACT |
| CAR VIII -F4 | TCACTGCTAGGAACAAGGGATACT |
| CAR VIII -R4 | CATGCAAATCGCTTACCCAT |
|  |  |
| CAR IX -F1 | GTTCAATACAGTCGAACAGTTGCA |
| CAR IX -R1 | CCCCAACATCGTAATTACTATCC |
| CAR IX -F2 | ATGGGAGTCAAGAGCGAAAAC |
| CAR IX -R2 | TTGGCAAATTCGAGCTACCAT |
| CAR IX -F3 | CGCTAATGTCGTAGGATTTCCT |
| CAR IX -R3 | TGGTATGGCTTATTATGCCTCTCT |
| CAR IX -F4 | CAATCCGGCAACATAATGACA |
| CAR IX -R4 | TCGCTATCTGAAAGAGTTTCCCA |
|  |  |
|  |  |
| CAR X -F1 | AAGCGTTTCTATCCAACCAGACT |
| CAR X -R1 | CACGTAAAAGTCGTGTTTTAACGTC |
| CAR X -F2 | GAATTAGAAGCTGAAGTTAGGAAGC |
| CAR X -R2 | CATGACGCTTCATTGTTTTATGC |
| CAR X -F3 | GCAAGCACTGTTCGTTTTTTCT |
| CAR X -R3 | GCCCTGTTATTAACTGAGCGTAGT |
| CAR X -F4 | TCAGTTAATAACAGGGCGCAAT |
| CAR X -R4 | GGTTAAGAAGGTAATCTGATAGCTTCTC |
|  |  |
| CAR XI -F1 | CAAGCATTTGCTCAGCAACA |
| CAR XI -R1 | TTCGAGATAGCTATTTGGCGGT |
| CAR XI -F2 | GGTTCAAGAGCTTTGGTTTAACCT |
| CAR XI -R2 | CCAGACAACAATGGACTCAACTT |
| CAR XI -F3 | CAGTTCATCTTCAGAATATTCGGGA |
| CAR XI -R3 | TCAAGGAGAAGTCGAATCAATAGC |
| CAR XI -F4 | AGACCATCATCACCTTCTGATATAGC |
| CAR XI -R4 | GGAAGCGATTTAATTTCAGGCA |
|  |  |
| CAR XII -F1 | CGATGTTTATGTGCGTGTTGA |
| CAR XII -R1 | TTCTGCAGTAGCCAATCCAATA |
| CAR XII -F2 | TTGACTGGATCCTCTAAGGGTATT |
| CAR XII -R2 | ACCTCCTAATATTGCCCTGACAT |
| CAR XII -F3 | CACCGAGTTTTAACGATGTCA |
| CAR XII -R3 | CGACGTTGTTTTCTTTGCTCA |
| CAR XII -F4 | GGGTAAACAAAGATGGCACCA |
| CAR XII -R4 | CACAATACCTACAATTTGCTTCC |
|  |  |
| CAR VI-F1 | CAAGTCTCTTAATAAGTAAGGAAGTCGC |
| CAR VI-R1 | TTTGTACTTGCTAAGCATGTCCTGT |
| CAR VI-F2 | TCAGGAGTGGAGACCATAATCATT |
| CAR VI-R2 | CGCAGCATATTGGTCGTTTTTA |

Table S4: List of primers used for FISH probes.

| **Locus** | **Primer sequence** |
| --- | --- |
| CAR I F | GCTAGACTGATGATTCGCCCTTATA |
| CAR I R | TTTACCGGCGAGTTAGCAGAGTA |
| CAR II F | ATTCGTGGCAGATTTTGCTCT |
| CAR II R | AAACAAGCGTTTGGCAGTAGC |
| CAR III F | CTACTTCTGTTGCCTAATAGTAAAAACCGT |
| CAR III R | ACTGTCCGTGAATCAATCGTG |
| CAR IV F | CAGGACAATTGGATGCTAAGTGTATT |
| CAR IV R | GGAACCTCTTCATTGCTTAAGCAT |
| CAR V F | TTACTGAGGTTGGAAAGAAGGCAT |
| CAR V R | GGGTCTGCTTATAAAATTCGTGAAC |
| CAR VI F | AAGACTGTTGTTGAGTGCTGTGGA |
| CAR VI R | CCATGCTTTTAGTGCGGTCA |
| CAR VII F | AGGCAGGAATCAGAAGGAGAGTT |
| CAR VII R | CAATCTCACCCACTTCTCCACTT |
| CAR VIII F | GGAAGCCGATATCGGGTTAATATT |
| CAR VIII R | CCATGACAGTTCAGAAACCTCAT |
| CAR IX F | GTTGCTTGCTTAACACTCACGAGTA |
| CAR IX R | CTGGTTCAATATCGATAATATACAAGCAGT |
| CAR X F | GACACTTACAATATGGTGCTCTACGA |
| CAR X R | TGCGGTCAATTGTTTTCATCTAC |
| CAR XI F | GGATACGTTCTATATGGTATGCATACGA |
| CAR XI R | ATCTTCACTCGCTTTTAAAGCCTGT |
| CAR XII F | AACCACCTATTCAAAGAGAGCTGCT |
| CAR XII R | TGTGTTTGCAGTATAAGATCTCTCCTC |
|  |  |
| rpl29Prom-F | GGTATTCGCTTTAGTAAACTACGCT |
| rpl29Prom-R | TGAGCCTTCTTGTTTTGGTTGT |
| dis2Prom-F | GTCTTACTTTTCCTCGGAACACTTTACT |
| dis2Prom-R | TCAAGTCTGCTTTTCCCCACT |
| srp1Prom-F | GTCGTCGGATTTCAATTTAGTGA |
| srp1Prom-R | ATTCATAAGCAAGCTCACGAGCA |
| rpl502Prom-F | ACGACGACGACGATATTTCGTCT |
| rpl502Prom-R | GGCTTATTTATGGCTATTAATCCACCT |
| hsp70_Prom_F | TCCCGCTTGTCCATTTTTCTCT |
| hsp70_Prom_R | TCCTCTTTTAAATTCCCAAGGGA |
| hsp9_Prom_F | CGCTCATTGGAAATAGCTTACGT |
| hsp9_Prom_R | TGCTCATGTCATTATGATCGCA |
|  |  |

Table S5: List of primers used for ChIP.

**Supplementary Figure legends**

**Figure S1: Comparison of Rad21 and Mis4 enrichment at CARs in logarithmic scale (log_2_ data).**

(A) Rad21 (green) enrichment at cohesive sites CAR I-VI from the ChIP-chip data. Likewise Mis4 enrichment (red) is plotted relative to a non-tag control (blue). Grey bars above and below 0 depict genes in sense and antisense orientation, respectively.

(B) Rad21 (green) and Mis4 (red) and non-tag control (blue) signals at non-cohesive sites CAR VII-XII as in (A). dg repeat is marked as a black line in CARVI box.

**Figure S2: Representative images of cohesive and non-cohesive loci**

(A) Representative images showing single dots at CARs IV and CAR VI in G2 phase only.

(C) Representative images showing single and double dots (white arrows) at CARs VII and CAR VIII in G2 phase only.

**Figure S3: Cohesive sites represent topological bound cohesin regulated by Mis4, Rad21 and Eso1.**

(A) Representative FACS analysis from wild type (WT) and *rad21-K1ts* mutant. Cycling cells were synchronized in G1 by nitrogen starvation (EMM-N_2_) released into rich medium and simultaneously shifted to 37°C to inactive Mis4 and Rad21. Cell cycle progression was monitored with FACS at 1 h interval. Both, WT and *rad21-K1ts* completed replication between t = 4-6 h. Subsequently, WT progressed normally but *rad21-K1ts* exhibited defective chromosome segregation.

(B) Representative images of DAPI stained nuclei in wild type, *mis4-367ts* and *rad21-K1ts* strains at 4 h (top panel), 6 h (middle panel) and 8 h (bottom panel) after shift to 37°C. Arrows indicate DNA fragmentation after 6 h and 8 h at 37°C, while WT maintain punctate nuclei throughout.

(C) DNA-FISH analysis in wild type (WT), *mis4-367ts* and *rad21-K1ts* strains. CAR III (green dots, top panel) and CAR VI (red dots, bottom panel) are shown after Mis4 and Rad21 inactivation by shift to 37°C after 4 h. n=50-100 cells

(D) Representative FACS analysis from no tag, Psm3-GFP WT and Psm3-GFP *eso1-H17* mutant strains. Cycling cells (G2) were synchronized in early S phase with hydroxyurea (HU, 15 mM 2 h) at 25°C. Subsequently, HU was washed off and cells released into G2 at 37°C. Cell cycle progression was followed by FACS at indicated time points.

(E) Representative FACS analysis from no tag and Eso1-GFP strains. Cycling cells (mostly G2) were synchronized in early S phase with hydroxyurea (HU, 15 mM 2 h) at 32°C. Subsequently, HU was washed off and cells released into G2 at 32°C. Cell cycle progression was followed by FACS at indicated time points.

(F) Representative FACS analysis from Eso1-GFP *cdc25-22* strain. Cycling cells (mostly G2) were synchronized in G2 phase by temperature shift to 37°C for 4 hours.

(G) Representative FACS analysis from no tag and Eso1-GFP *cdc10-101* strains. Cycling cells (25°C) were synchronized in G1 phase by temperature shift to 37°C for 4 hours followed by release to 25°C.

**Figure S4: Mis4+/Rad21+ and Rad21+ sites on chromosomal arms.**

(A) Boxplot showing Pds5 overlap with Mis4+/Rad21+ and Rad21+ sites versus random DNA regions. A higher fraction of Mis4+/Rad21+ regions overlap with Pds5 binding sites than Rad21+ sites only. Significance was determined by Fisher Exact Test (p<10^-5^). Randomly chosen DNA regions (equal in size and number to Pds5 peaks) were sampled >1000 times. The overlap with Mis4+/Rad21+ sites and Rad21+ sites was determined every time. The one-sided p-value was determined by counting the quantity of overlaps with the random regions as extreme as the observed overlap with Pds5 peaks. Error bars indicate one standard deviation.

(B) Boxplot showing Mis4+/Rad21+ overlap with RNAPIII transcription factor Sfc6. Boxplot of absolute distance between margins of Sfc6 and Rad21 peaks at Mis4+/Rad21+ and Rad21+ sites. Outliers are represented by black dots. The median is plotted as a black line and whiskers extend to the maximal value, which is not an outlier. p<10^-11^, one sided Wilcoxon Rank Sum test.

(C) Histogram of proximity between Sfc6 and Rad21 at Mis4+/Rad21+ and Rad21+ sites. Each bin represents 5 kbp and each midpoint is plotted. Data is normalized to the number of Mis4+/Rad21+ and Rad21+ peaks, respectively. Peaks are centered at 0 and any overlap with an Sfc6 peak is assigned the distance 0. Distances between peak margins and not midpoints are plotted.

**Figure S5: Psm3 and Mis4 at *hsp 70* and *hsp9* promoters.**

(A) ChIP-qPCR analysis showing Psm3-GFP enrichment at *hsp70* and *hsp9* promoters after shift from 32°C to 42°C, 30 min. ChIP samples were treated with RNaseA/T mix prior immunoprecipitation. Values are normalized to 32°C. Error bars represent SD, n=3. Data were extracted and plotted from three biological repeats.

(B) ChIP-qPCR analysis showing Mis4-GFP enrichment at *hsp70* and *hsp9* promoters after shift from 32°C to 42°C, 30 min. ChIP samples were treated with RNaseA/T mix prior immunoprecipitation. Values are normalized to 32°C. Error bars represent SD, n=3. Data were extracted and plotted from three biological repeats.

**Figure S6: Chromatin association of cohesin in human cells is mediated via RNAPII.**

(A) Metagene analysis of cohesin and RNAPII ChIP-Seq data showing cohesin distribution relative to RNAPII. Blue line represents number of cohesin peaks present at the corresponding distance (bp, x axis) from the RNAPII peak summit. Most cohesin is enriched within +/- 600 bp of RNAPII.

(B) Boxplot comparing expression (RPKM) of genes that overlap SMC1 and SMC1/NIPBL sites. p<0.05, Wilcoxon Rank Sum test.

(C) Transcription inhibition affects cohesin enrichment at *GAPDH*. Schematic representation of the *GAPDH* gene showing probes used for ChIP-qPCR. RNAPII signal (left graph) at the transcription start site (TSS) was reduced to background levels as at intron 2 (in2) after treatment with α-amanitin (2 μg/ml, 36 h). Likewise RAD21 (middle graph) and SMC3 levels (right graph) were also reduced after α-amanitin treatment. Error bars=SEM, n=3. * p<0.05, one-tailed paired student’s t-test.

(D) Reduction in RNAPII (left graph), RAD21 (middle graph) and SMC3 (right graph) levels at the TSS of c-*MYC* but not at the CTCF+/cohesin+/RNAPII- “up” region, after α-amanitin treatment as in (C). Error bars=SEM, n=3. * p<0.05, one-tailed paired student’s t-test.

(E) Cohesin enrichment coincides with RNAPII transcription at *ERBB2* promoter. *ERBB2* is highly expressed in ZR-75-1 cell line relative to non-expressing MCF-7 cell line (compare RNAPII levels, left graph). High transcription is followed by RAD21 (middle graph) and SMC3 enrichment, while *HPRT* is the negative control. Error bars=SD, n=3. * p<0.05, one-tail paired student’s t-test.

(F) Western blot showing reduction in total RNAPII (N20X antibody) after α-amanitin treatment (2 μg/ml, 36 h). In contrast, RAD21 (ab992), SMC3 (Millipore) and tubulin (ab6160) levels were unaffected.

(G) Western blot showing similar RAD21 and SMC3 levels between ZR-75-1 and MCF-7 cell lines. Tubulin serves as a loading control.

**Figure S7: Cohesin overlaps highly transcribed RNAPII loci in *S. cerevisiae***

(A) Pie chart showing ~92% overlap between Scc1 and RNAPII peaks in budding yeast.

(B) Scc2+/Scc1+ peaks overlap highly transcribed genes in comparison to Scc1+ alone. Boxplots compare expression level (RPKM) of genes that overlap Scc2+/Scc1+ and Scc1+ sites. p <10^-23^, one-sided Wilcoxon signed rank test.

(C) Scc4+/Scc1+ peaks overlap highly transcribed genes in comparison to Scc1+ alone. p <10^-16^, one-sided Wilcoxon signed rank test.

(D) Scc2+/Smc3+ peaks overlap highly transcribed genes in comparison to Smc3+ alone. p <10^-16^, one-sided Wilcoxon signed rank test.

(E) Scc4+/Smc3+ peaks overlap highly transcribed genes in comparison to Smc3+ alone. p <10^-10^, one-sided Wilcoxon signed rank test.

**Figure S8: Cohesin association on the arms is independent of ChIP artifacts at open chromatin hyper-ChIPable regions in *S. cerevisiae*.**

(A) Bar graph showing ~80% Scc2+/Scc1+ (left graph), ~85% Scc4+/Scc1+ (right graph) and ~95% Scc1+ (both graphs) peaks are devoid of the 238 hyper-ChIPable regions.

(B) Bar graph showing ~80% Scc2+/Smc3+ (left graph), ~85% Scc4+/Smc3+ (right graph) and ~95% Smc3+ (both graphs) peaks are devoid of the 238 hyper-ChIPable regions.

(C) Scc2+/Scc1+ sites overlap highly expressed genes in comparison to Scc1+ sites, with or without inclusion of the 238 annotated hyper-ChIPable regions. p <10^-19^, one-sided Wilcoxon signed rank test.

(D) Scc4+/Scc1+ sites overlap highly transcribed genes in comparison to Scc1+sites. p <10^-14^, one-sided Wilcoxon signed rank test.

(E) Scc2+/Smc3+ peaks overlap highly transcribed genes in comparison to Smc3+ alone. p <10^-14^, one-sided Wilcoxon signed rank test.

(F) Scc4+/Smc3+ peaks overlap highly transcribed genes in comparison to Smc3+ alone. p <10^-8^, one-sided Wilcoxon signed rank test.

**Figure S9: Model for cohesion establishment at cohesive and non-cohesive sites.**

During G1, RNAPII recruits cohesin to actively transcribed gene loci. RNAPII or Mis4 recruit Eso1 to the Mis4+/Rad21+ sites. Continuing in S phase, Eso1 acetylates cohesin, thereby resulting in cohesin stabilization and establishment of stable sister chromatid cohesion. In contrast, cohesion association with chromatin is dynamic and unstable at Rad21+ loci.
