# Supplementary material for: Transcription facilitates sister chromatid cohesion on chromosomal arms
Source: Nucleic Acids Res. 2016 Apr 15;44(14):6676–92. doi: 10.1093/nar/gkw252 (PMC5001582; doi:10.1093/nar/gkw252)

Supplementary Figure 1

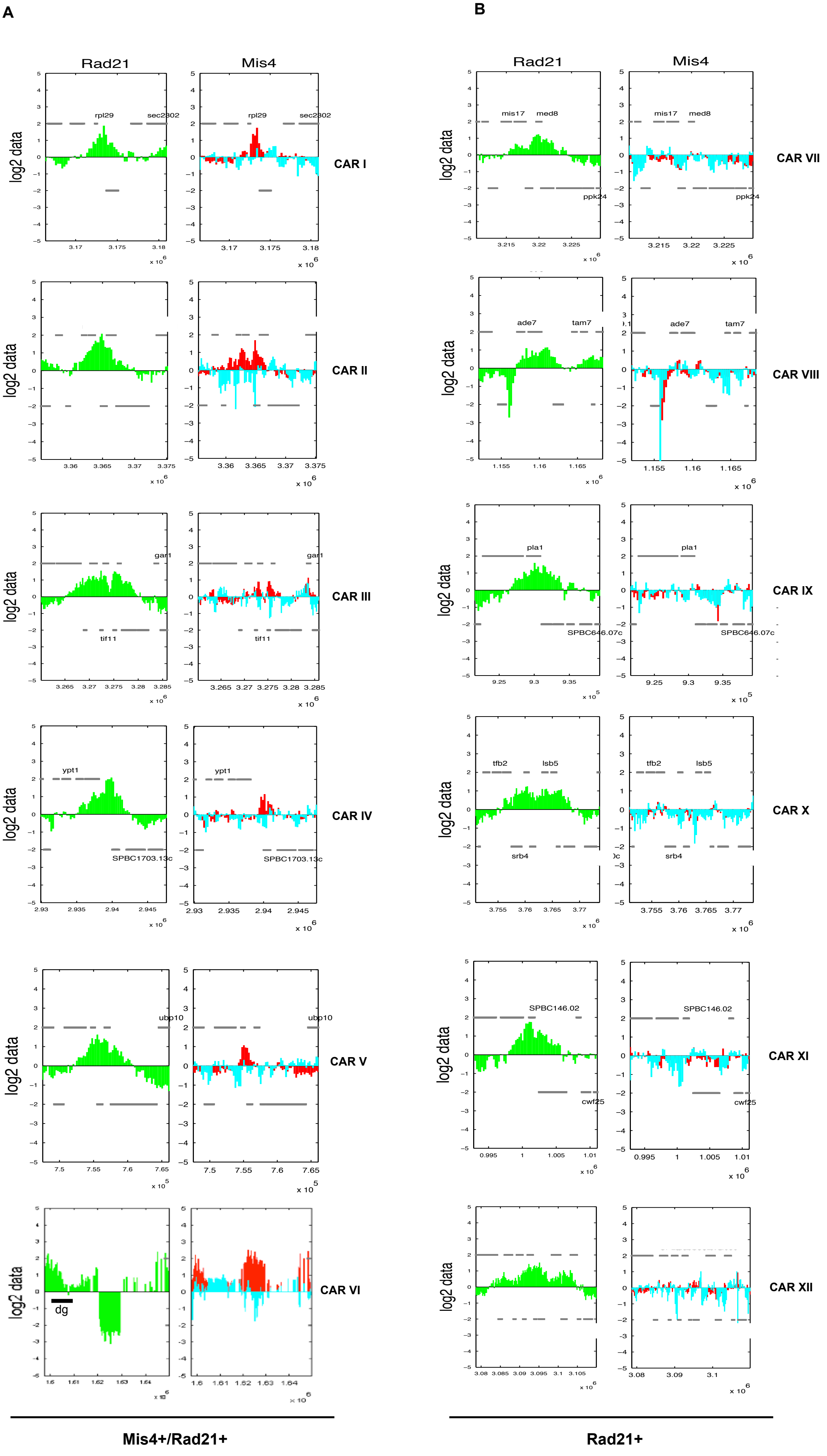

Mis4+/Rad21+

A

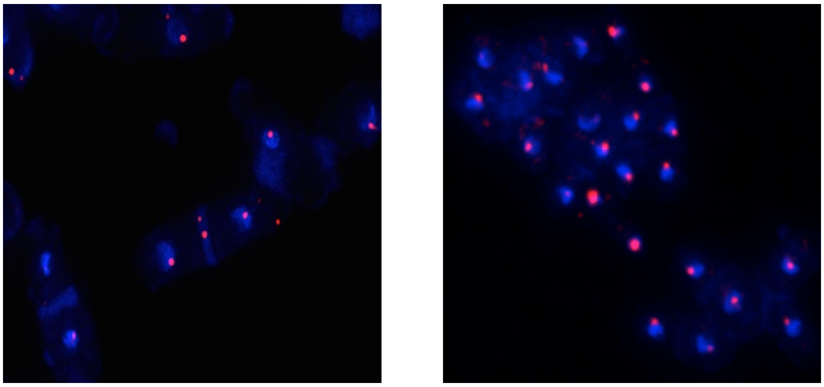

CAR IV CAR VI

Rad21+

B

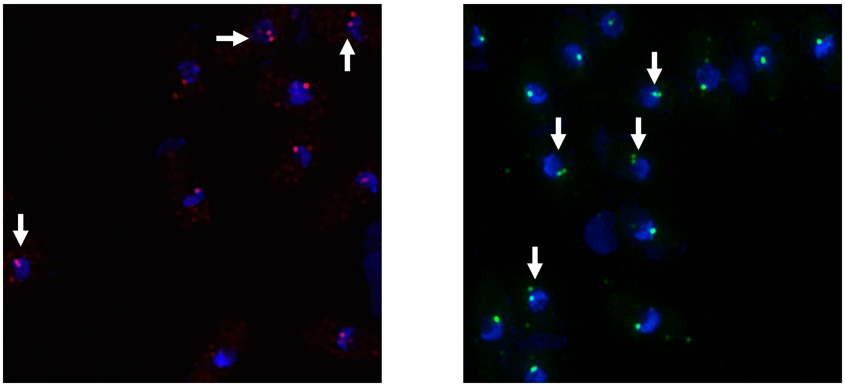

CAR VII CAR VIII

Supplementary Figure 3

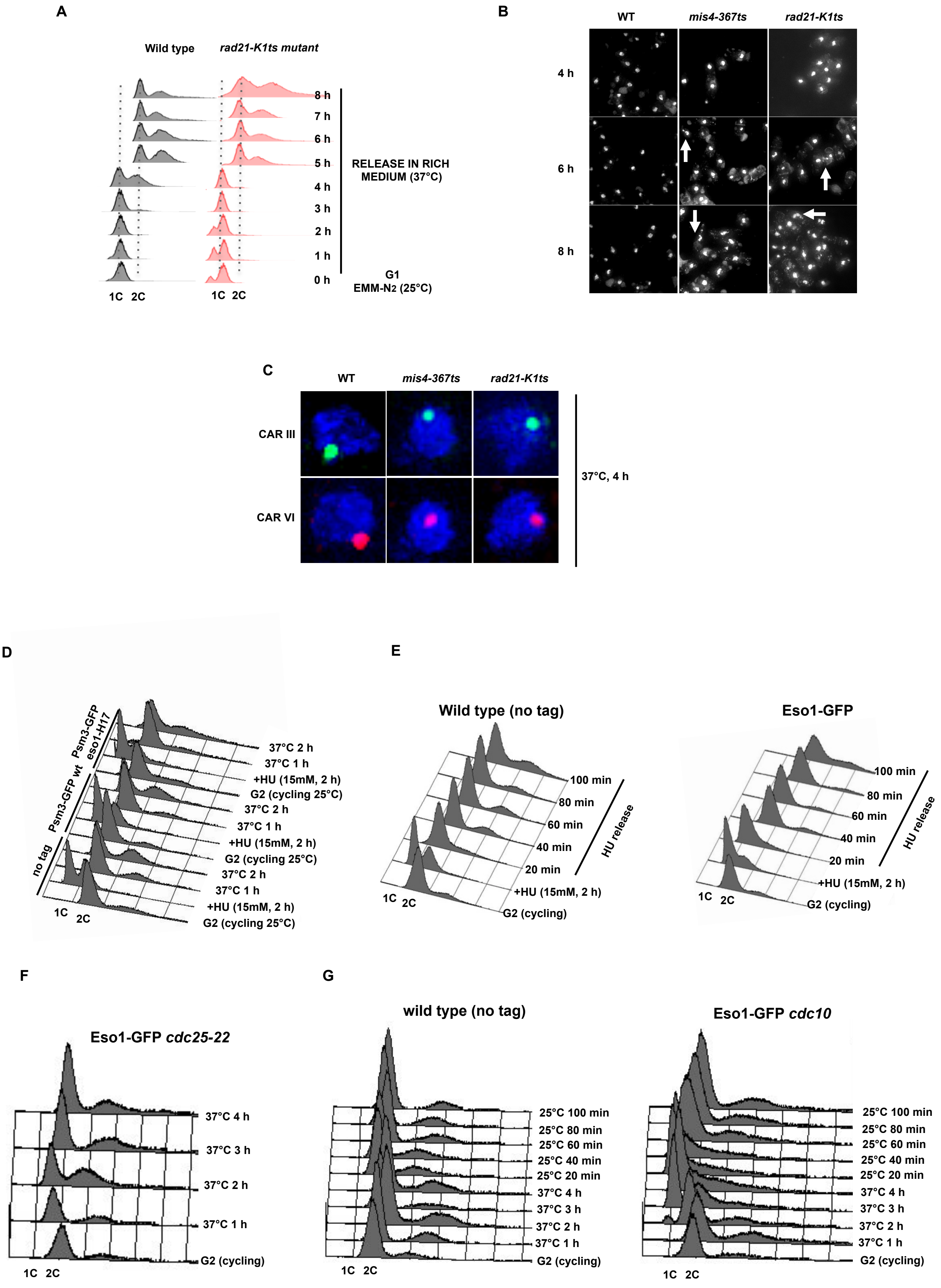

A

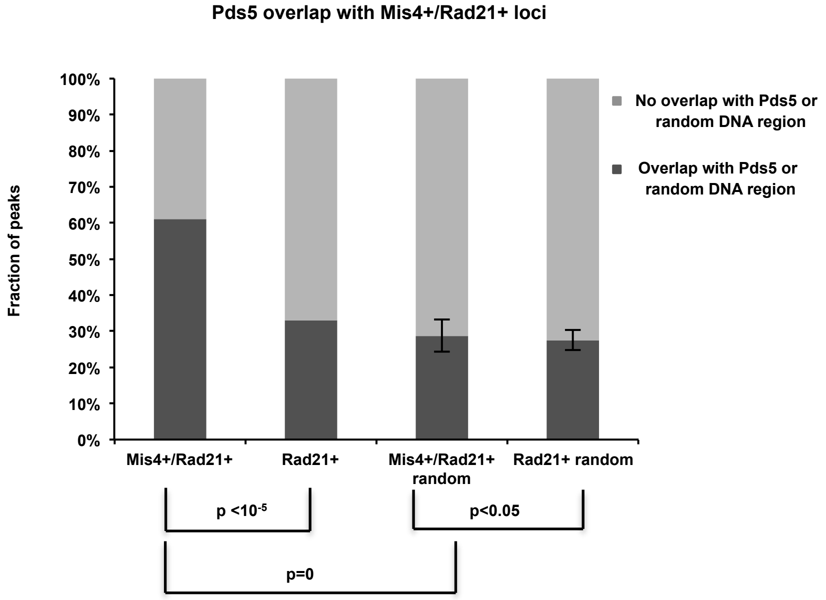
Pds5 overlap with Mis4+/Rad21+ loci
Fraction of peaks
100%
90%
80%
70%
60%
50%
40%
30%
20%
10%
0%
No overlap with Pds5 or random DNA region
Overlap with Pds5 or random DNA region
Mis4+/Rad21+
Rad21+
Mis4+/Rad21+ random
Rad21+ random
p <10-5
p<0.05
p=0

B

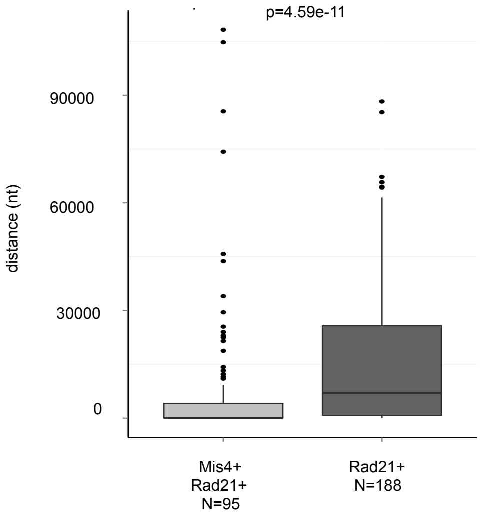
p=4.59e-11
distance (nt)
90000
60000
30000
0
Mis4+ Rad21+ N=95
Rad21+ N=188

C

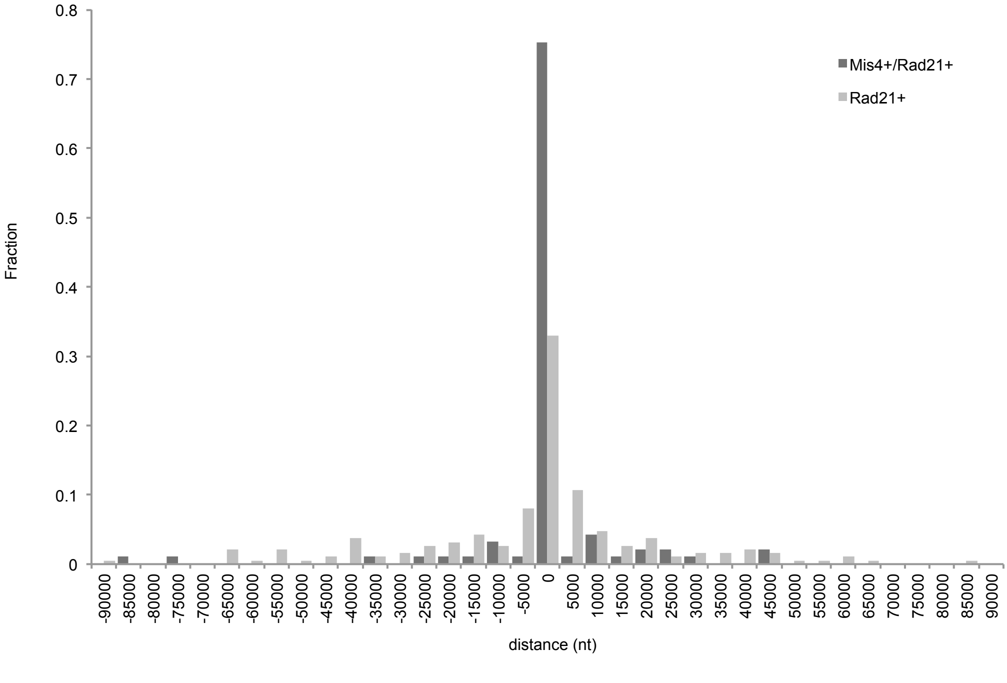
Fraction
0.8
0.7
0.6
0.5
0.4
0.3
0.2
0.1
0
Mis4+/Rad21+
Rad21+
distance (nt)
-90000 -85000 -80000 -75000 -70000 -65000 -60000 -55000 -50000 -45000 -40000 -35000 -30000 -25000 -20000 -15000 -10000 -5000 0 5000 10000 15000 20000 25000 30000 35000 40000 45000 50000 55000 60000 65000 70000 75000 80000 85000 90000

Supplementary Figure 5

A

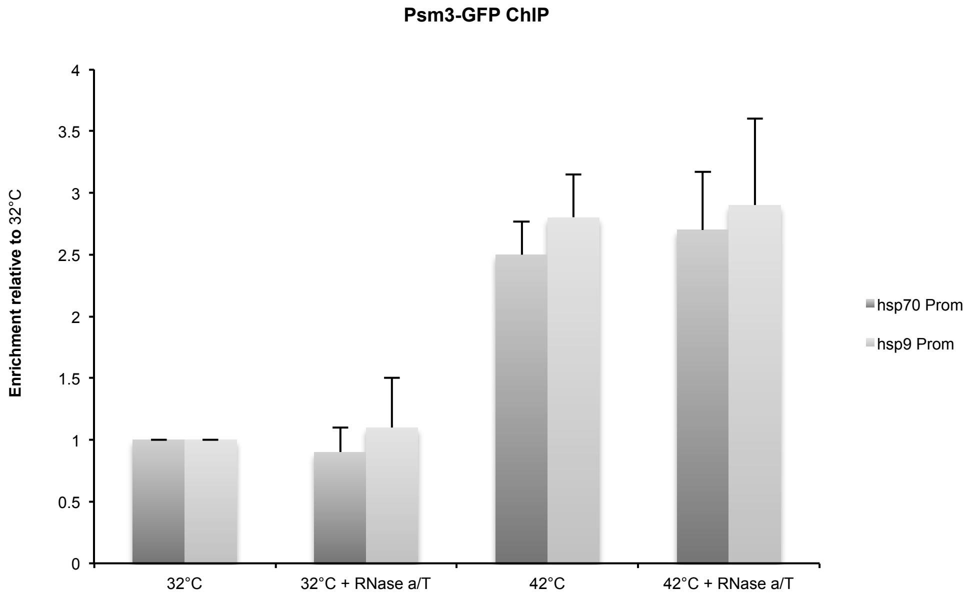

B

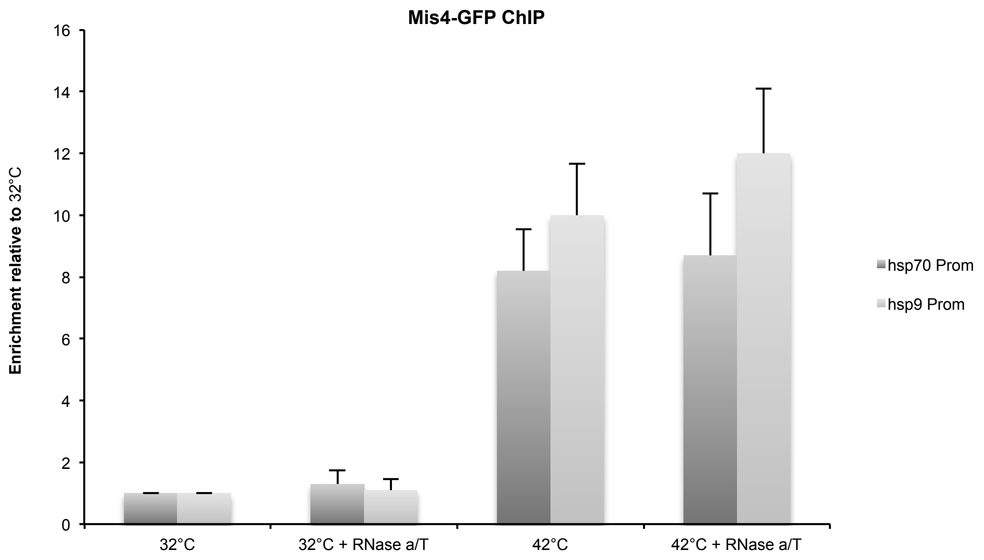

Supplementary Figure 6

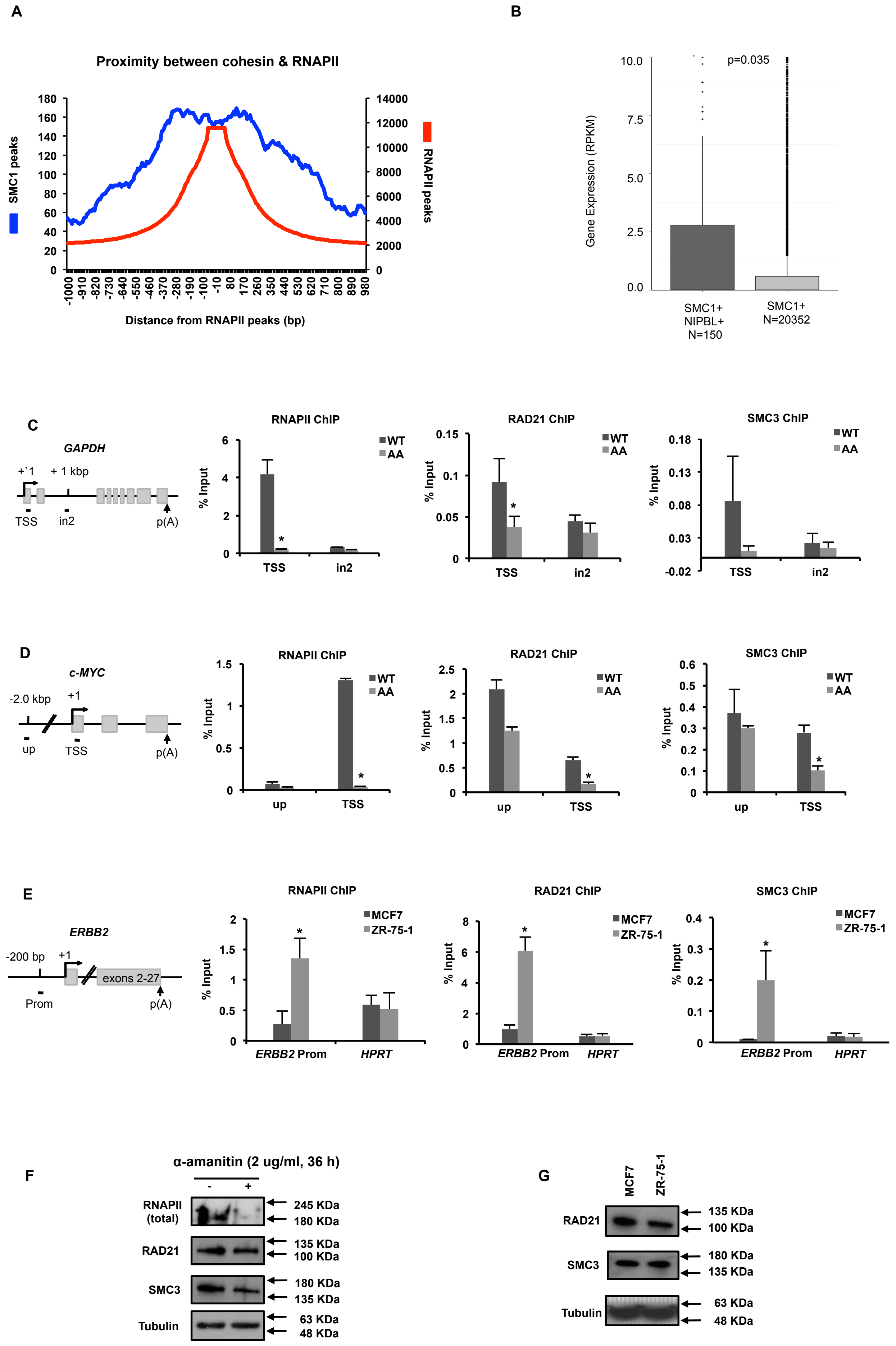

Supplementary Figure 7

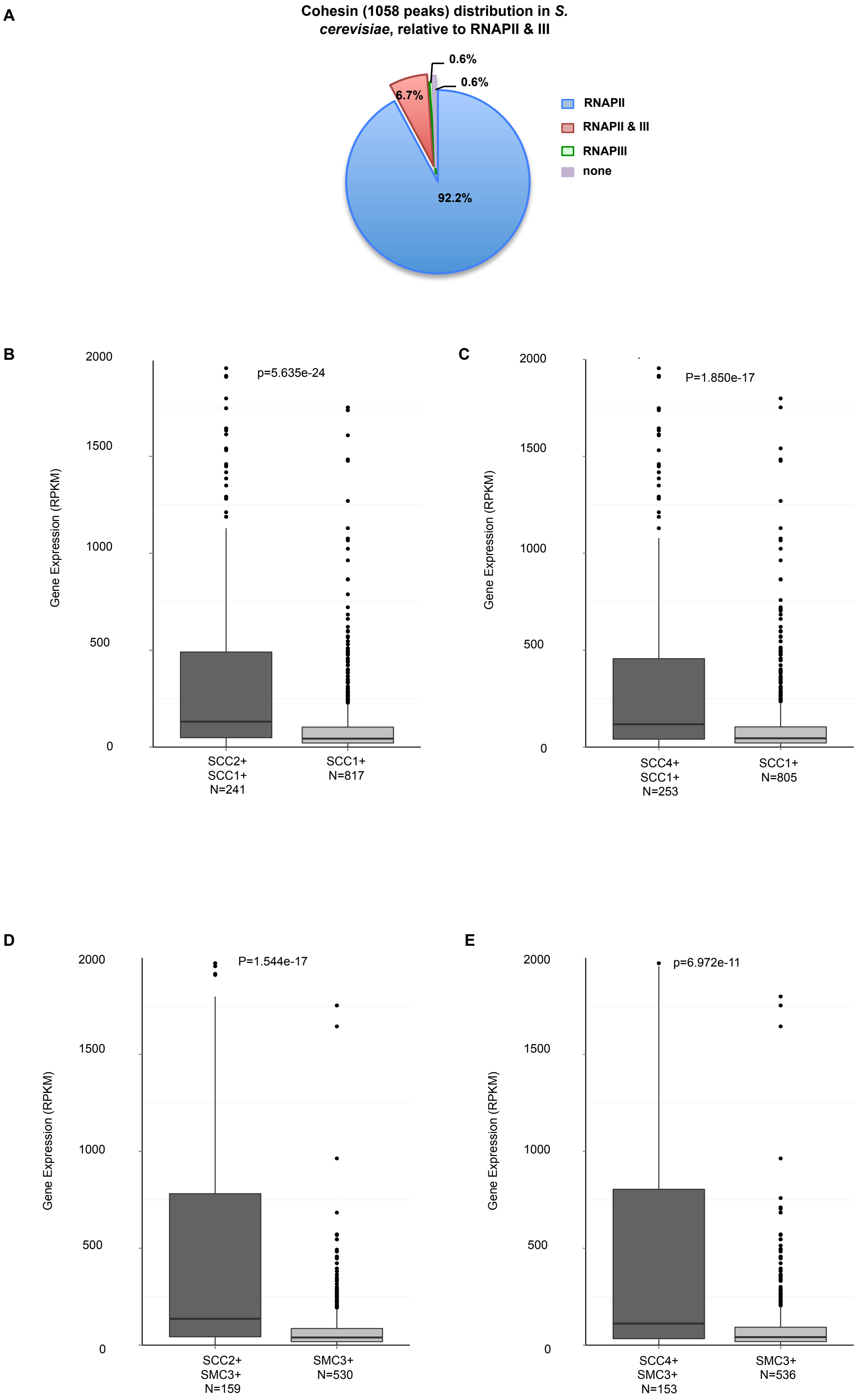

Supplementary Figure 8

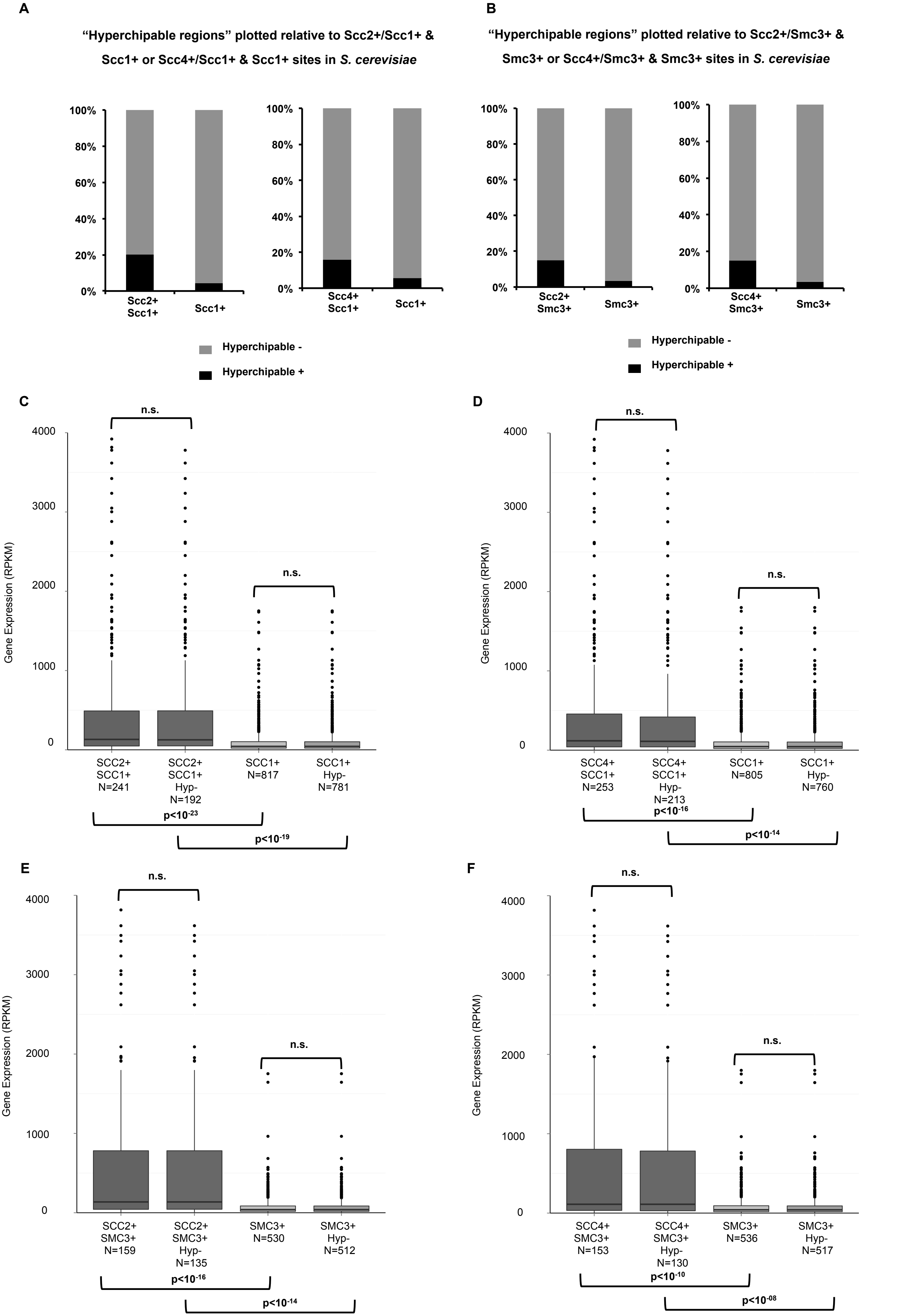

Supplementary Figure 9

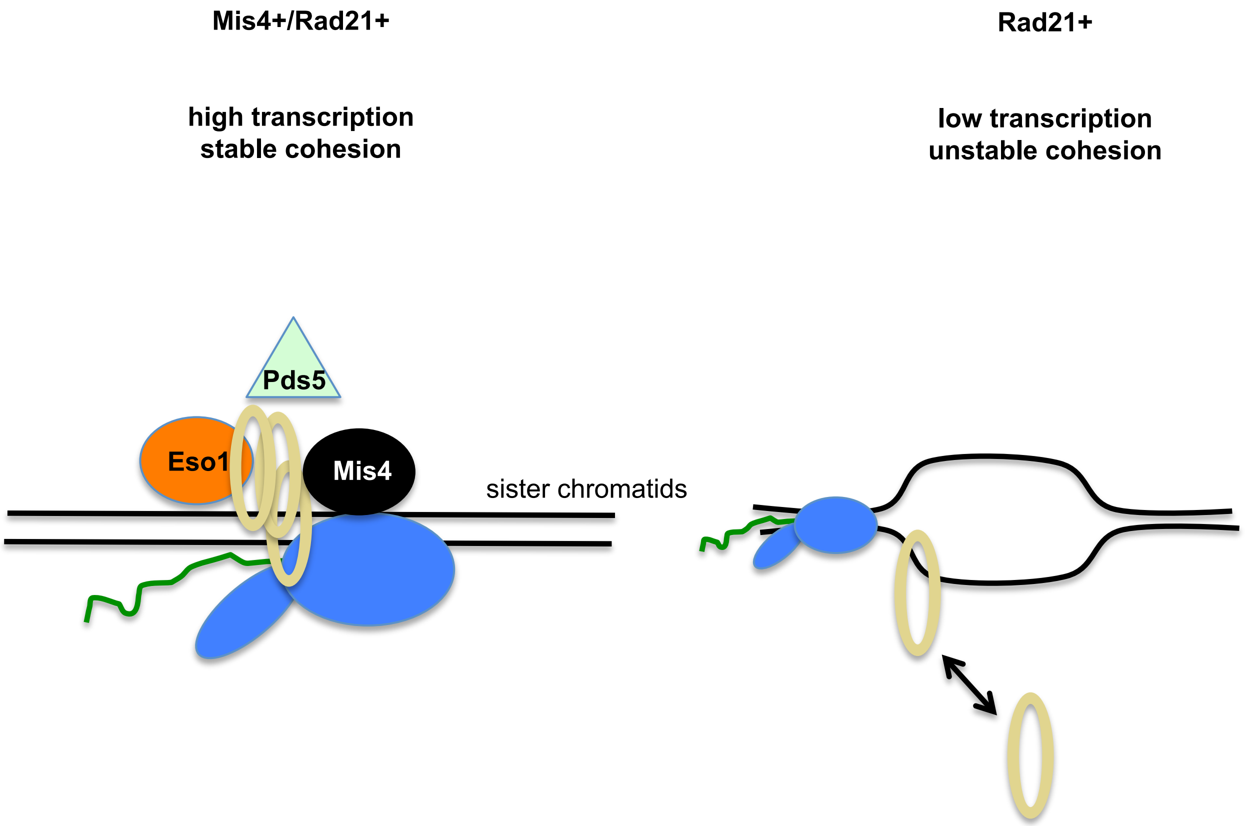

Supplement: SUPPLEMENTARY DATA [file supp_gkw252_nar-00155-v-2016-File004.pdf]
