# Supplementary figures and images for: Transcription facilitates sister chromatid cohesion on chromosomal arms
Source: Nucleic Acids Res. 2016 Apr 15;44(14):6676–92. doi: 10.1093/nar/gkw252 (PMC5001582; doi:10.1093/nar/gkw252)

CAR1

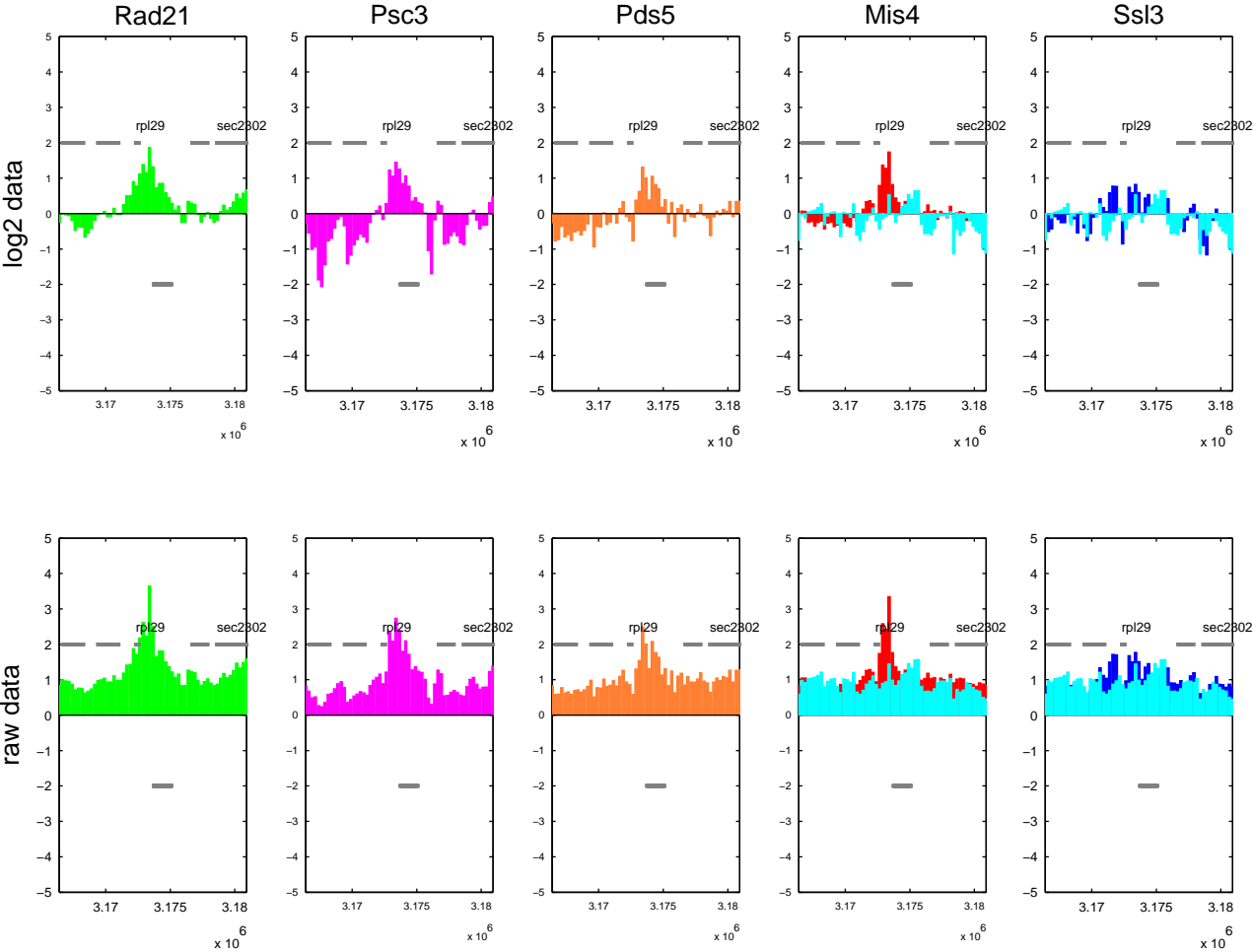

CAR2

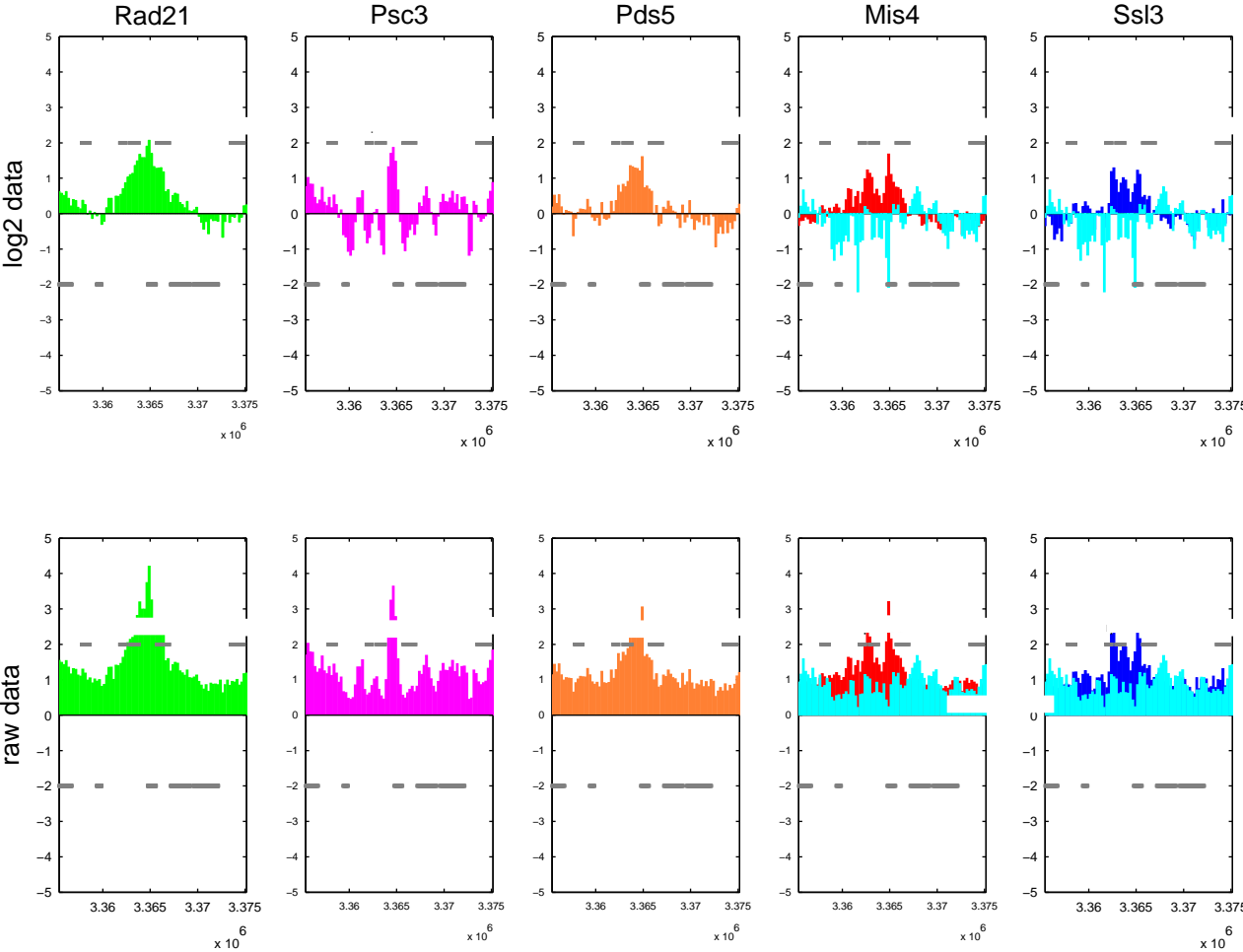

CAR3

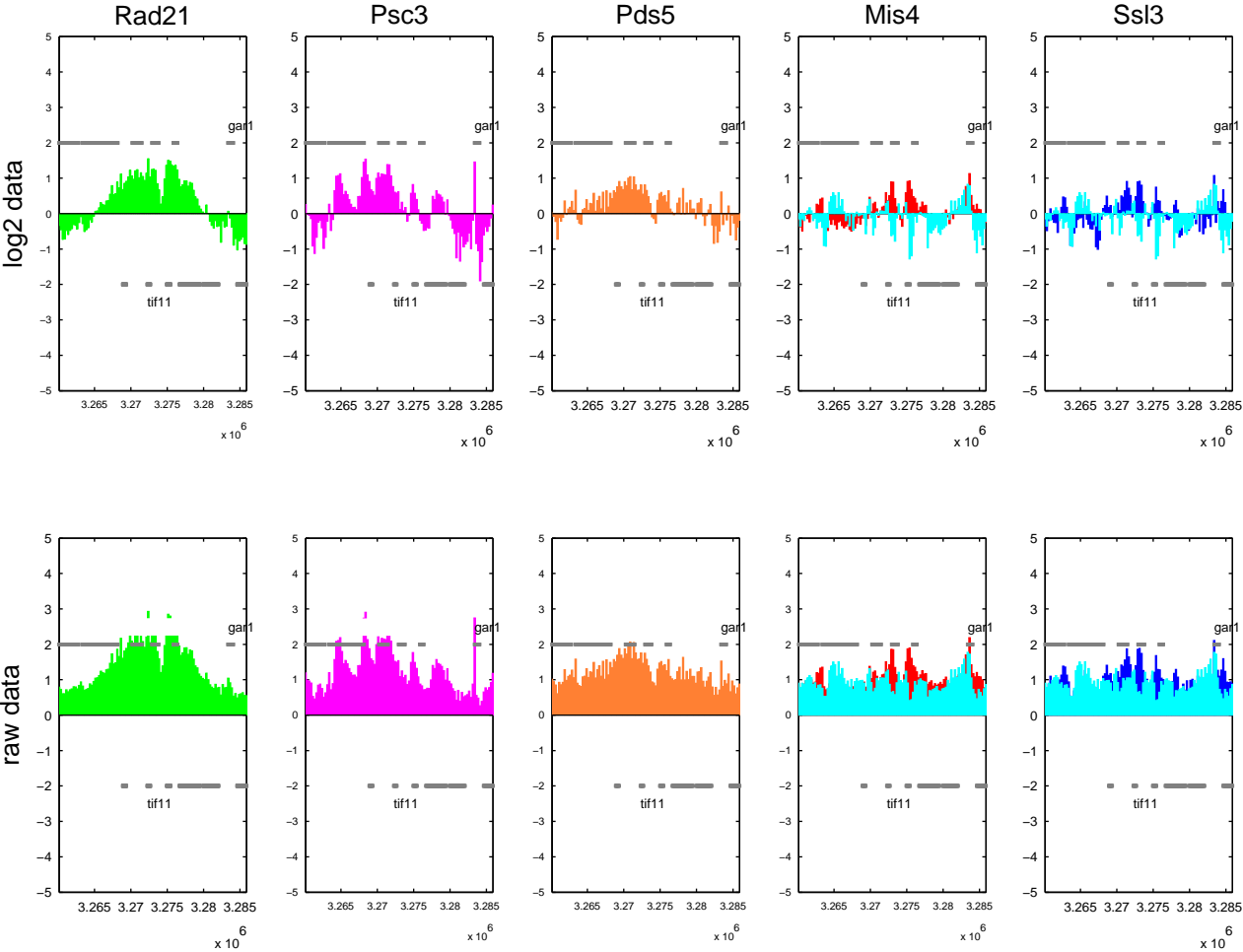

CAR4

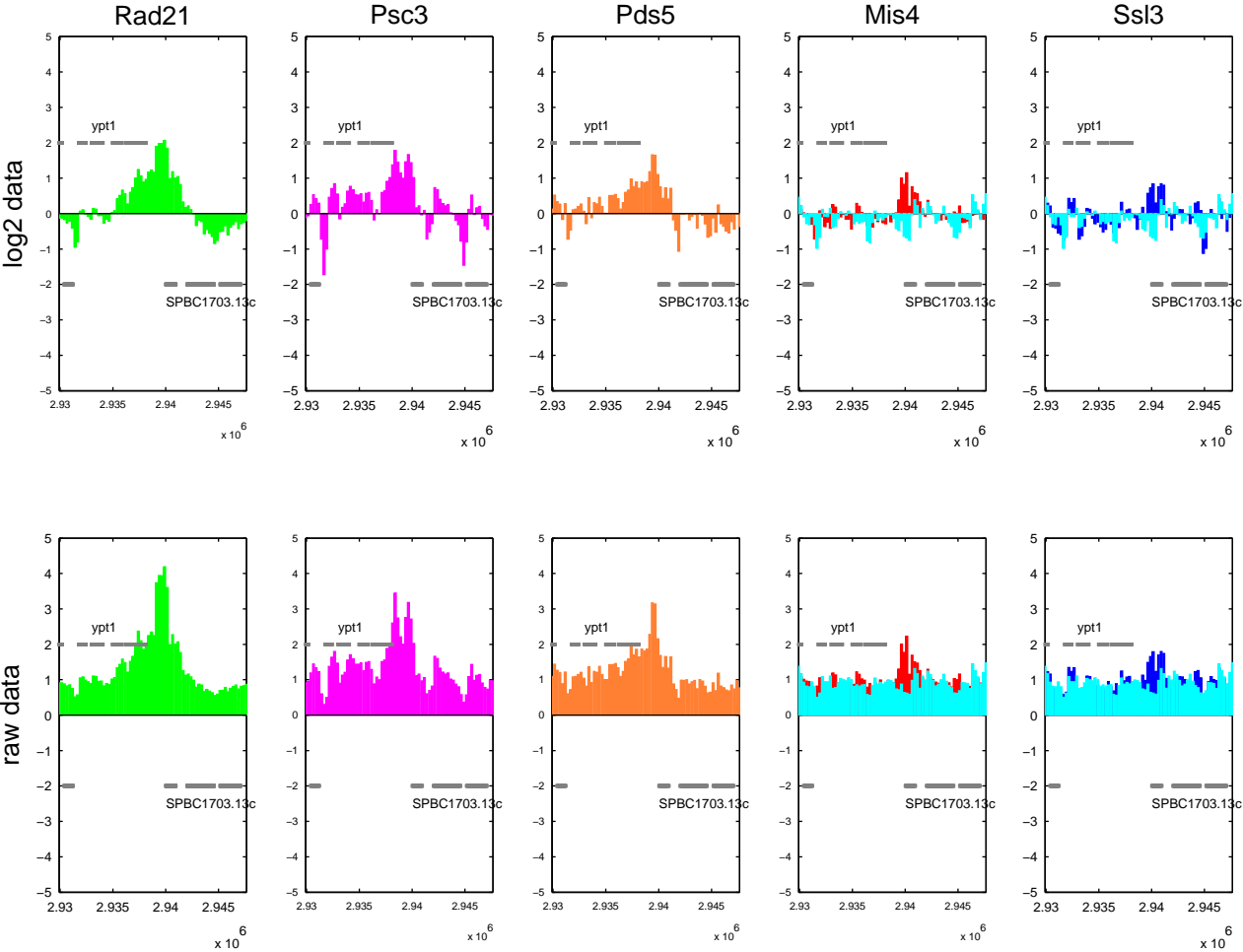

CAR5

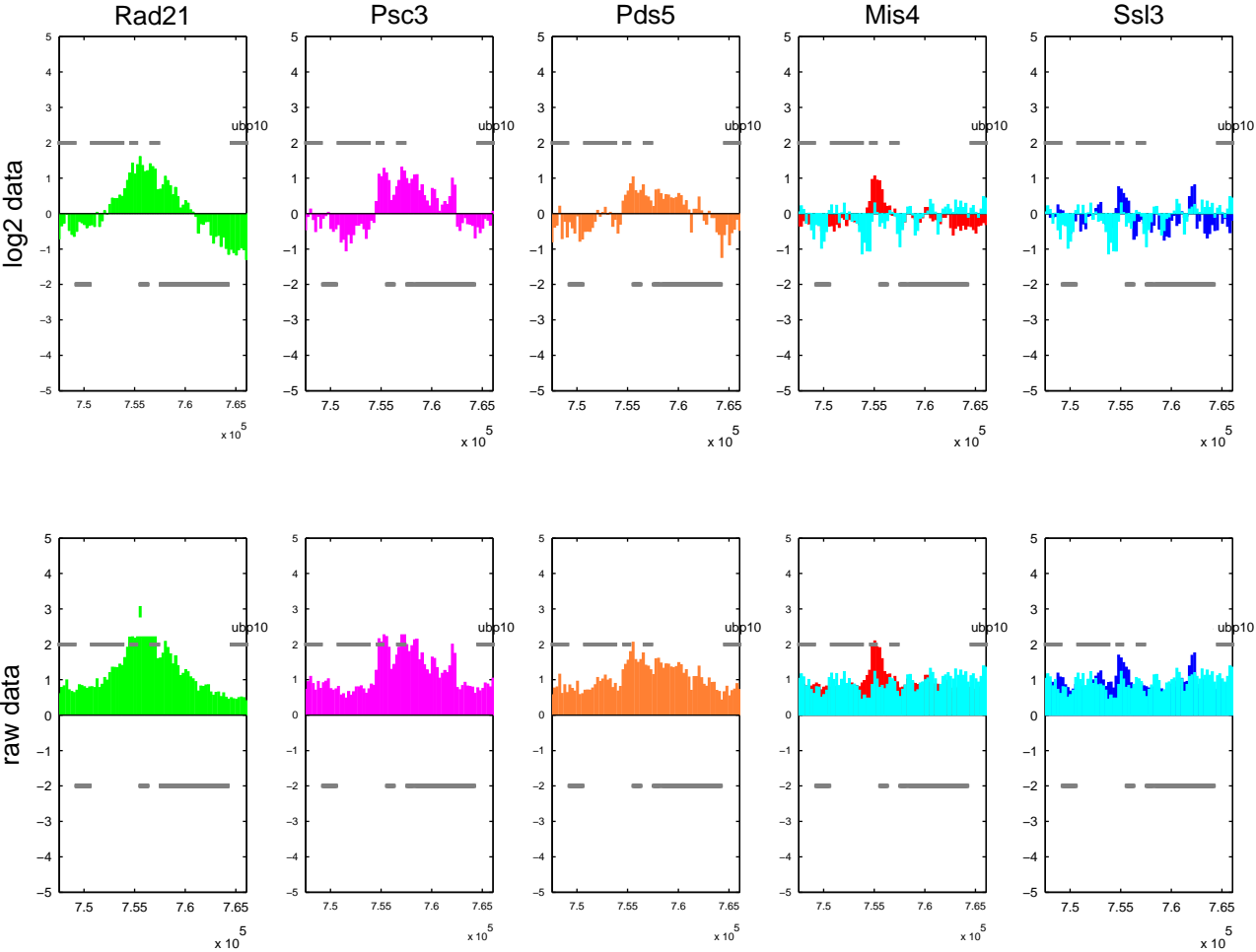

CAR6

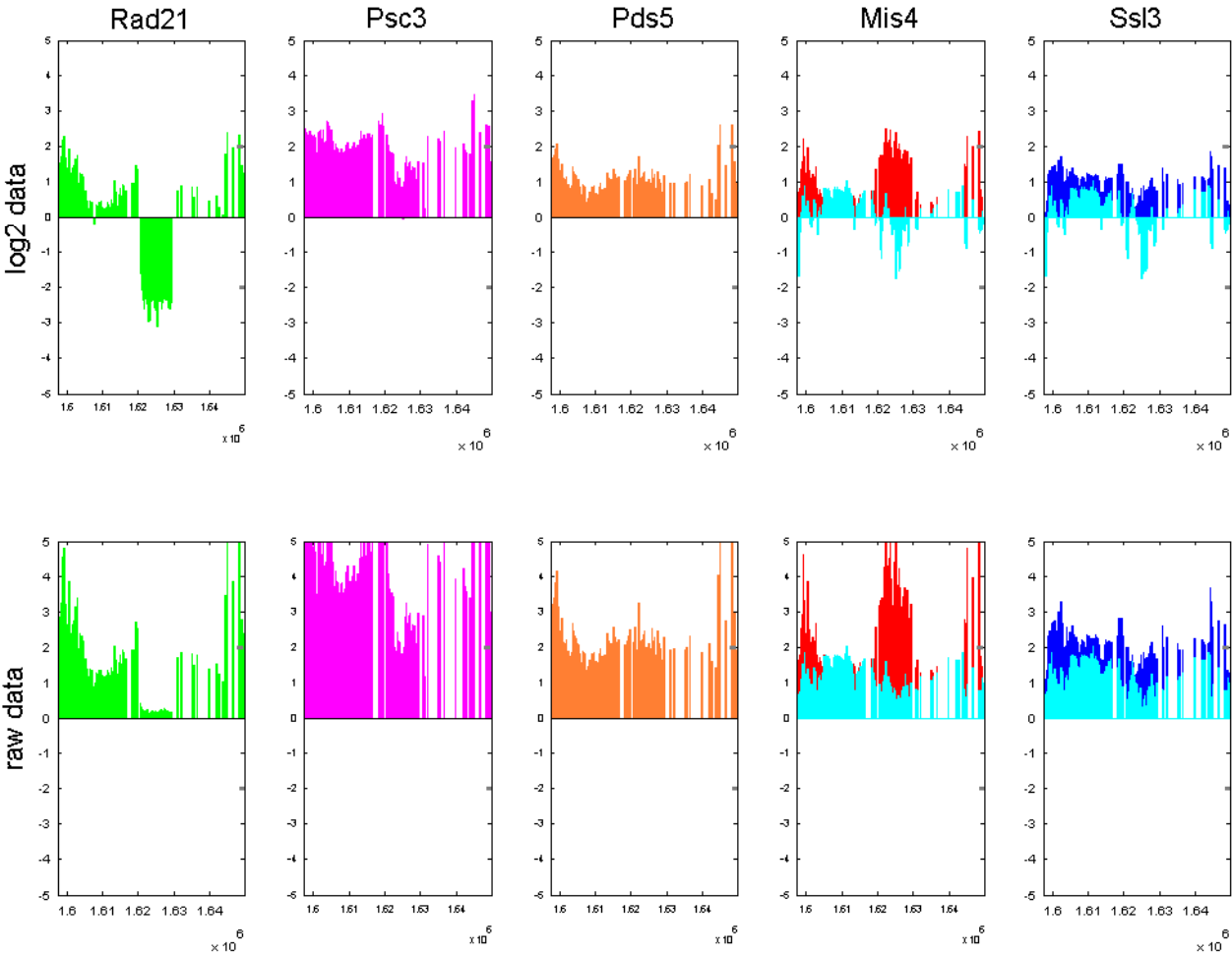

CAR7

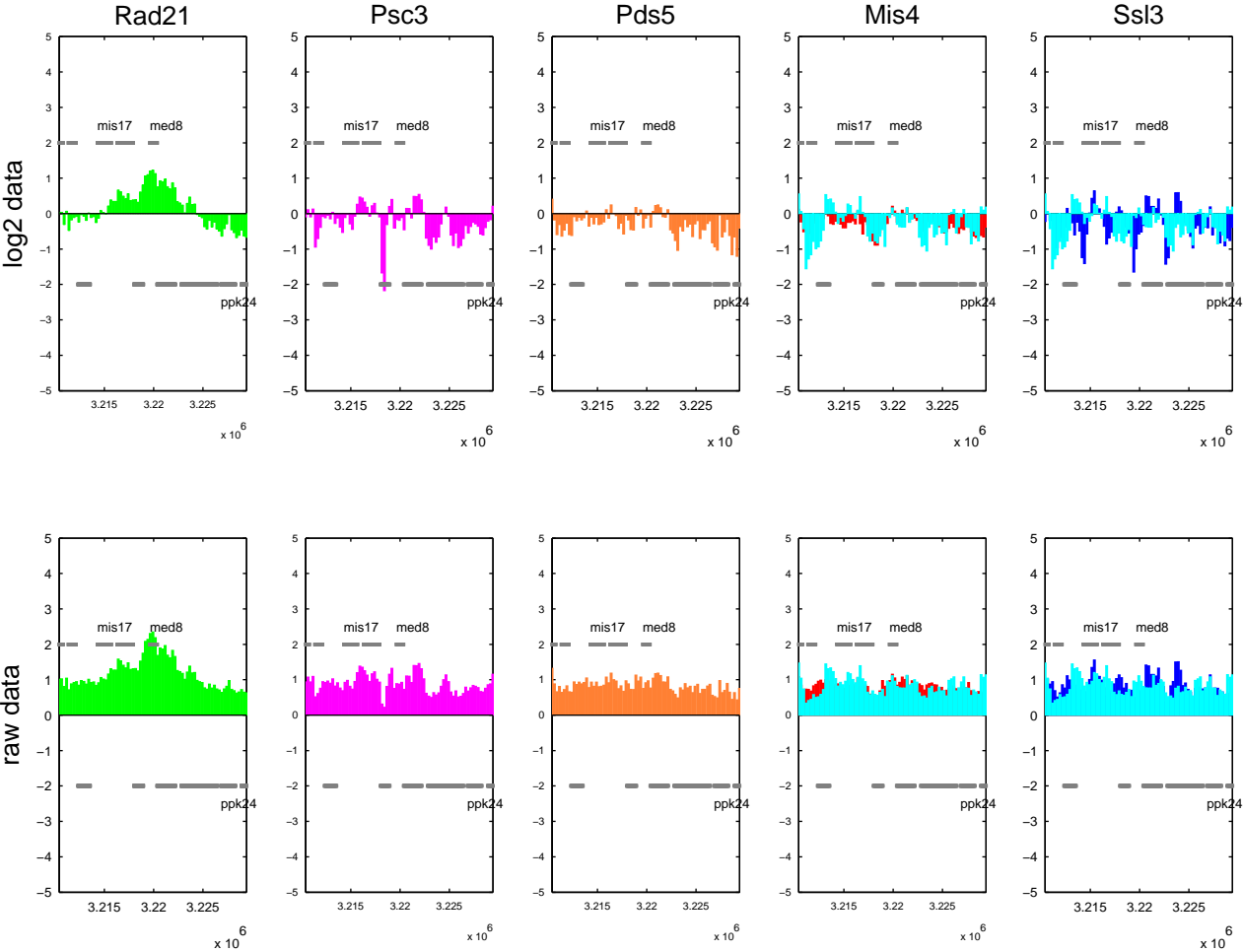

CAR8

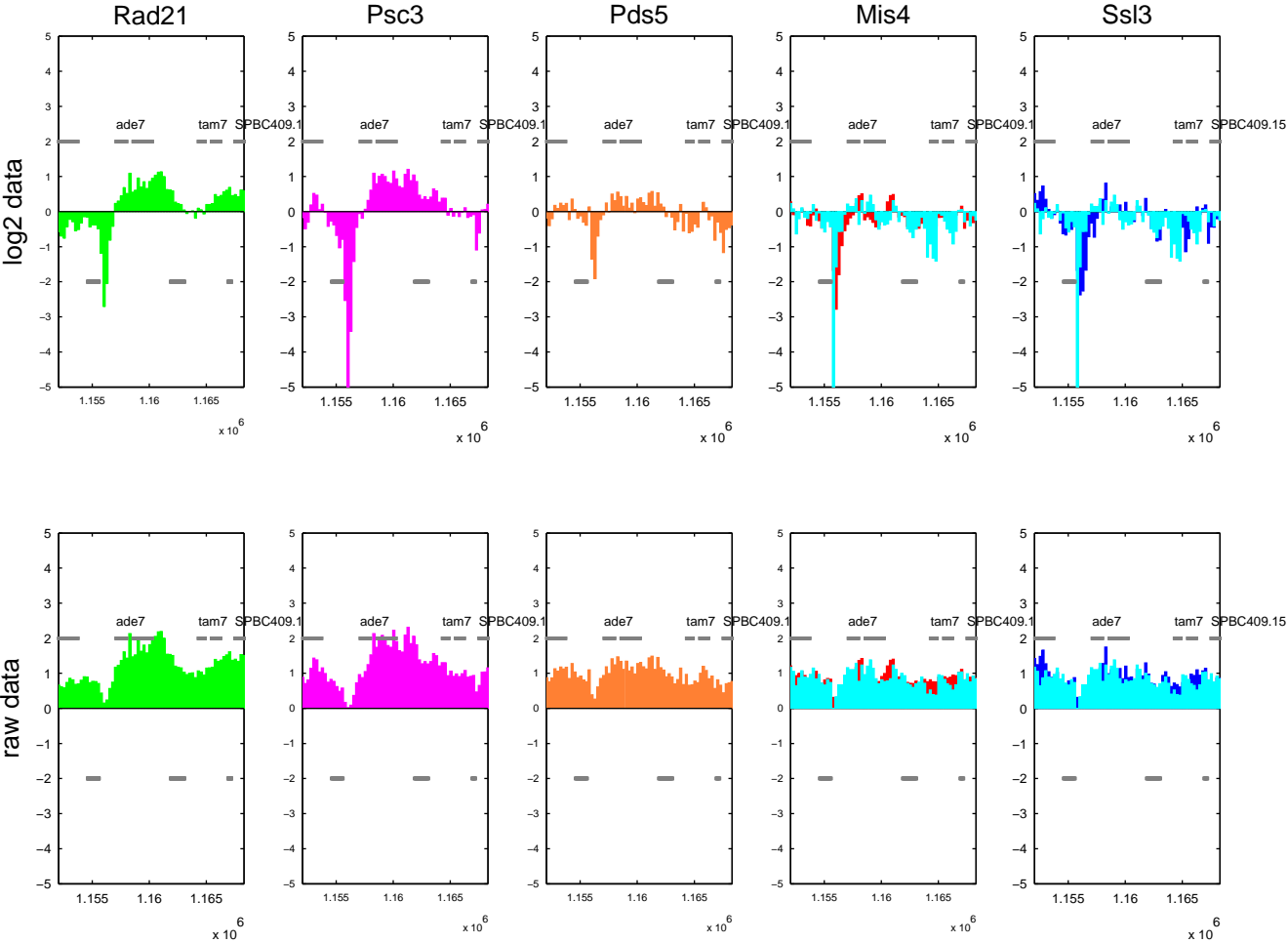

CAR9

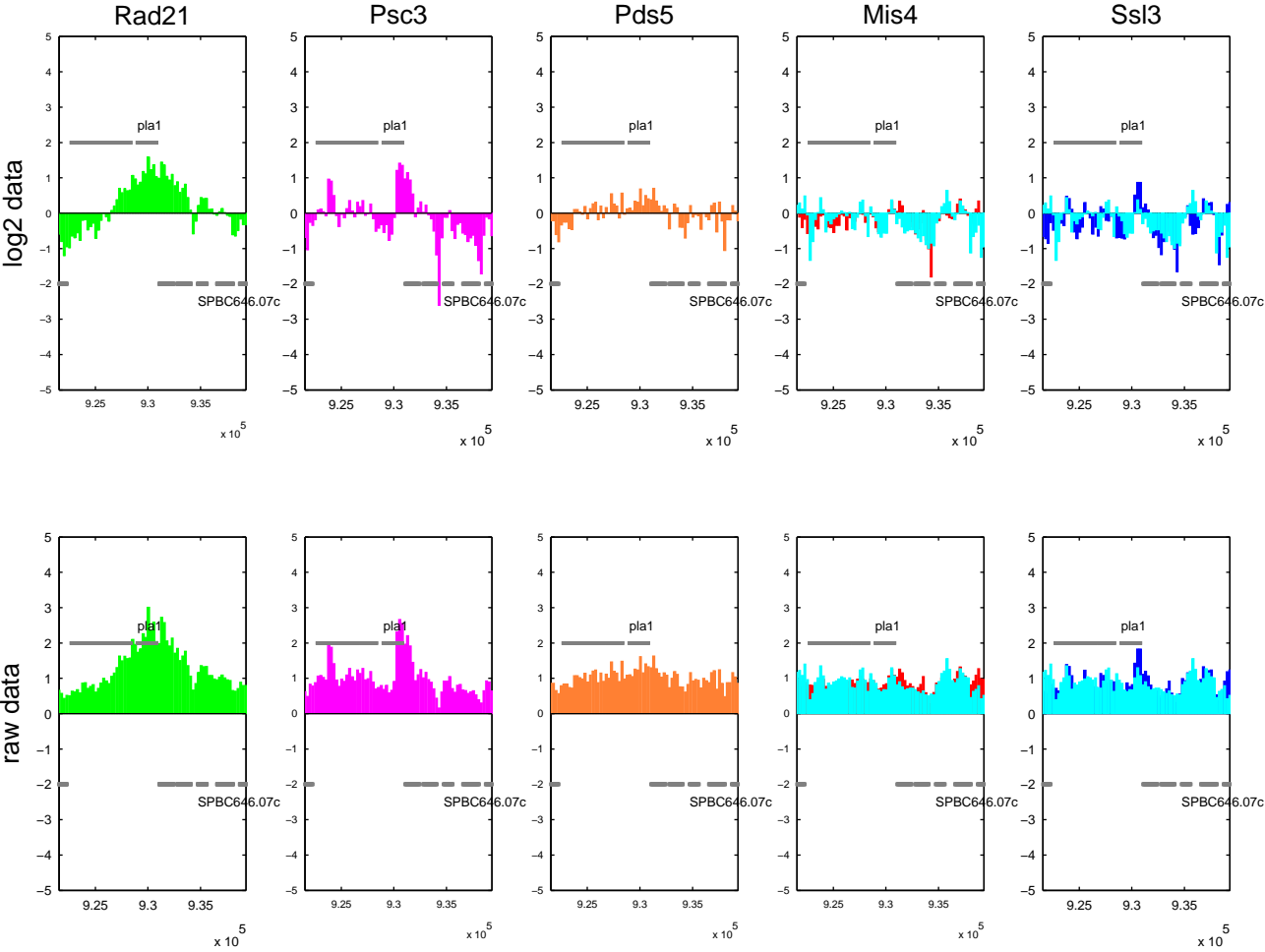

# CAR10

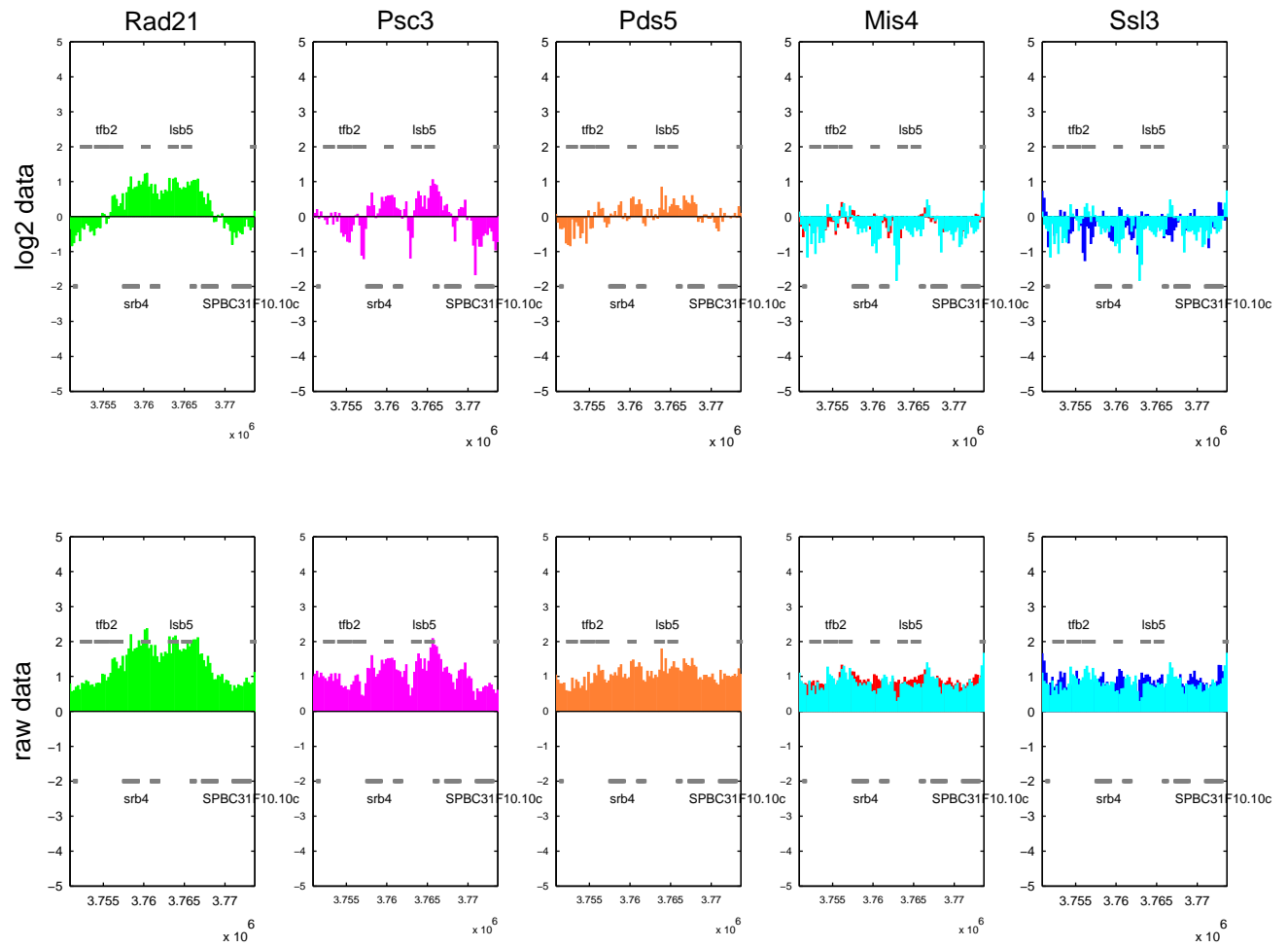

# CAR11

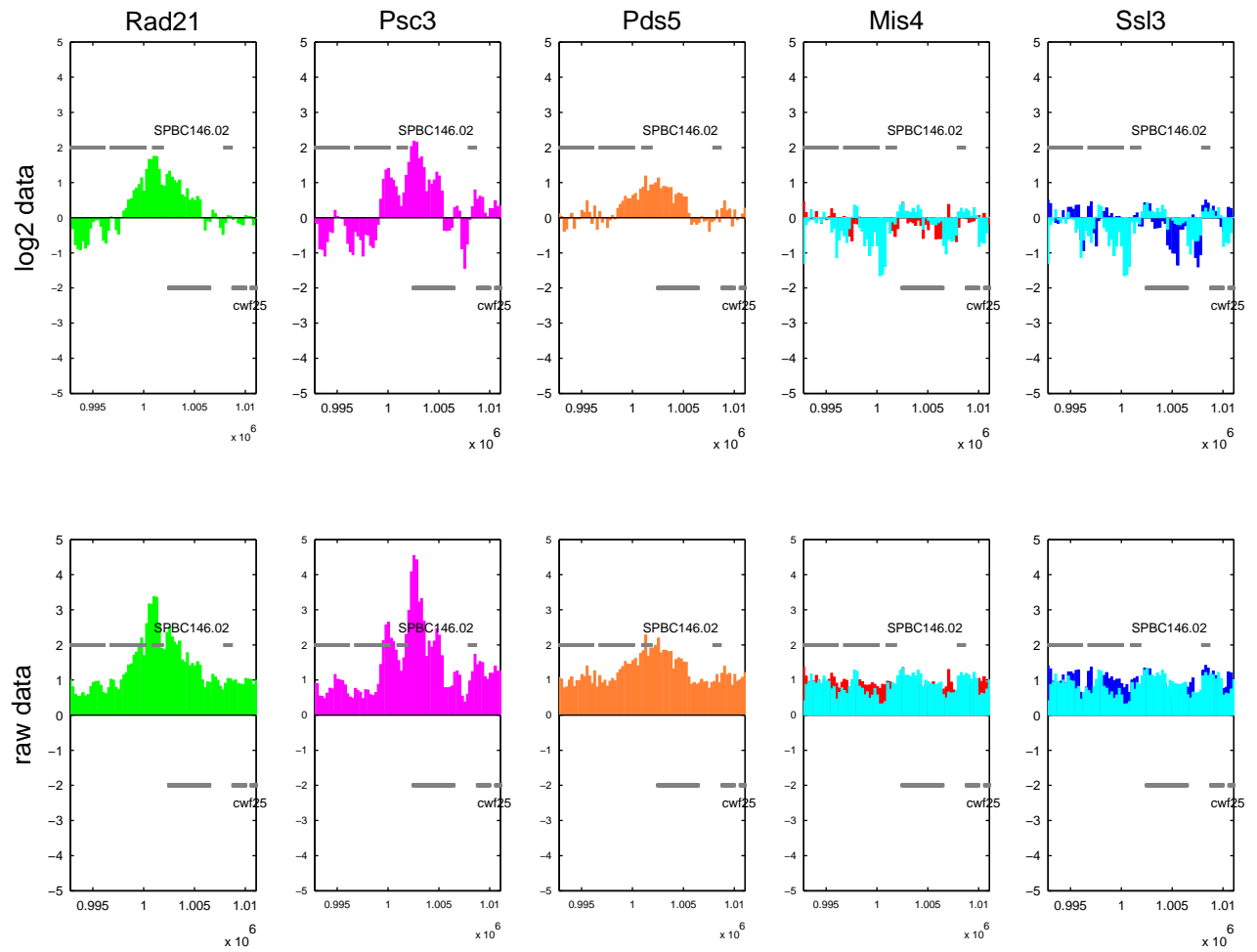

# CAR12

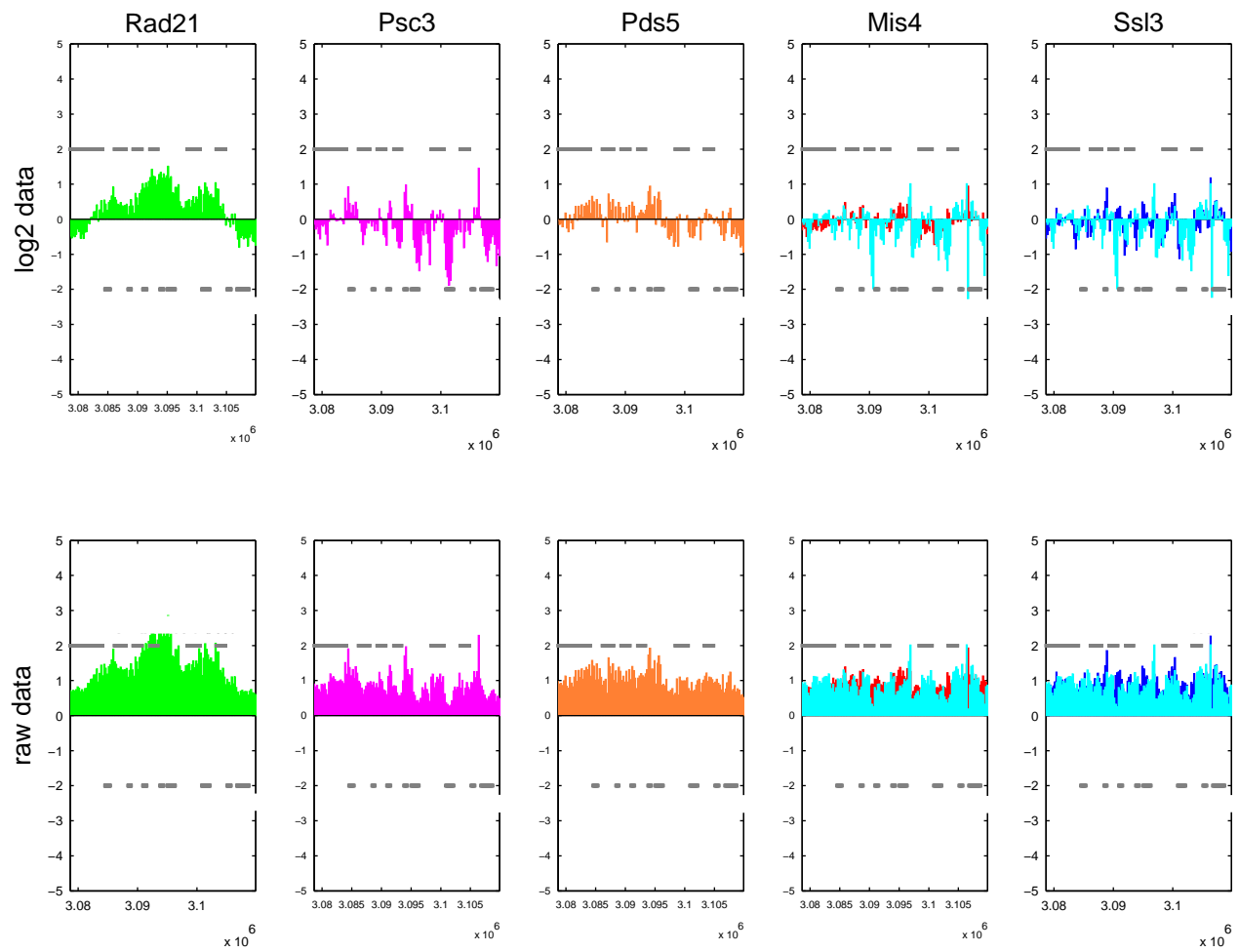

Supplement: SUPPLEMENTARY DATA [file supp_gkw252_nar-00155-v-2016-File005.pdf]

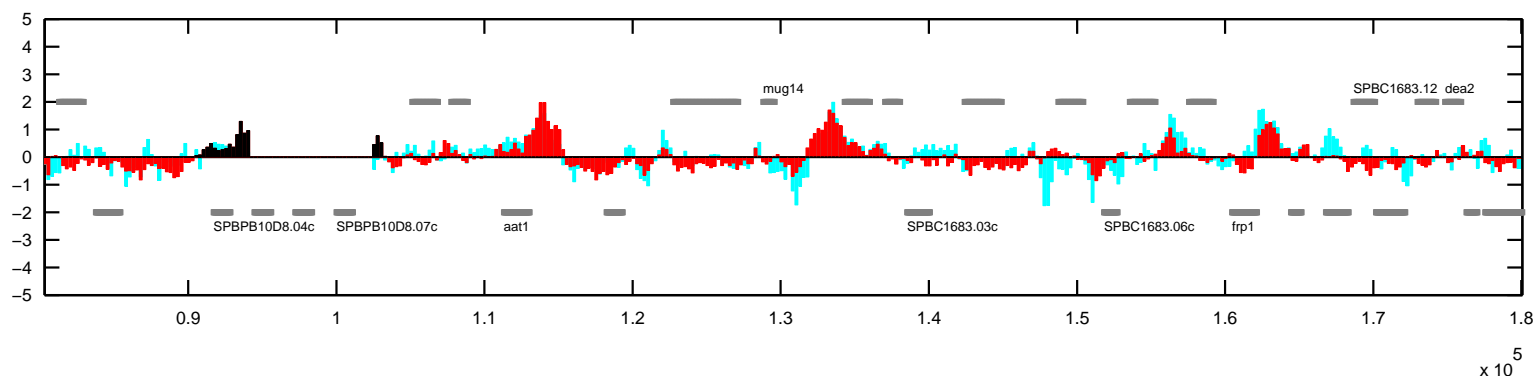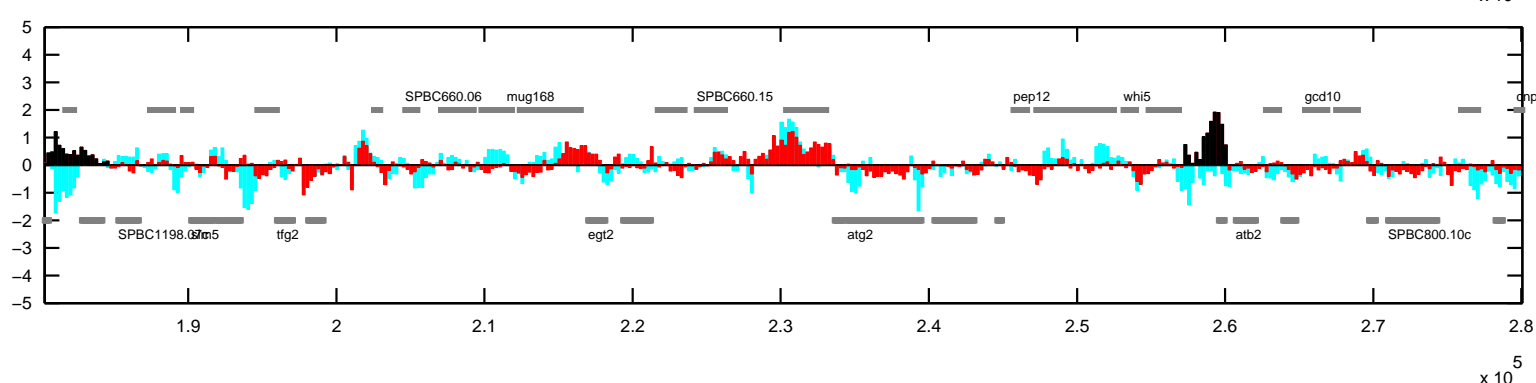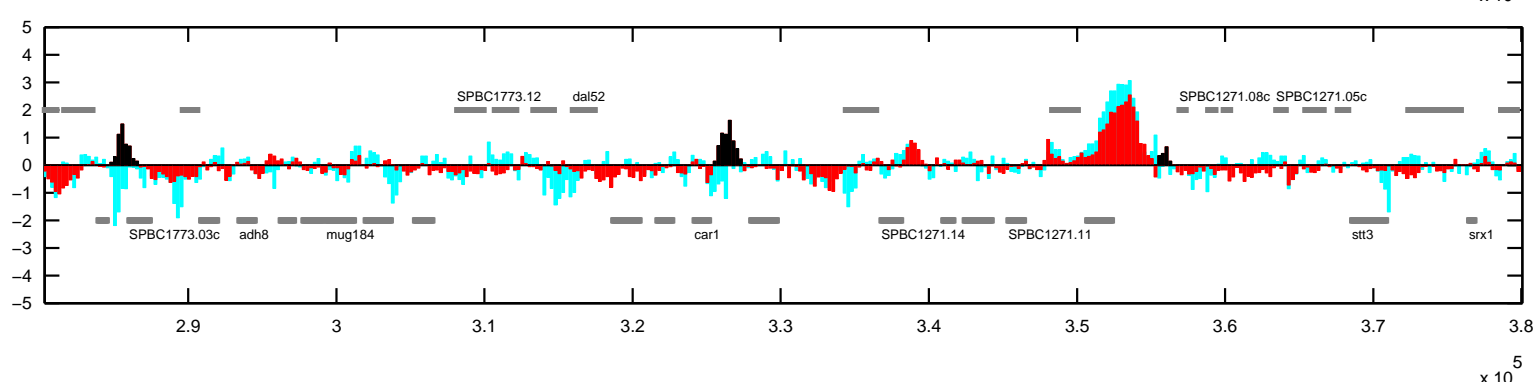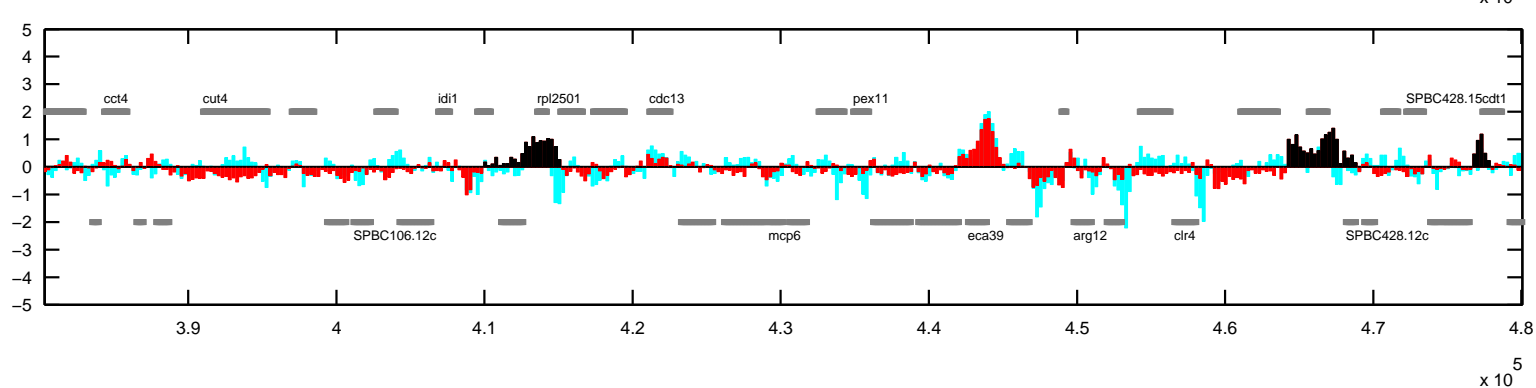

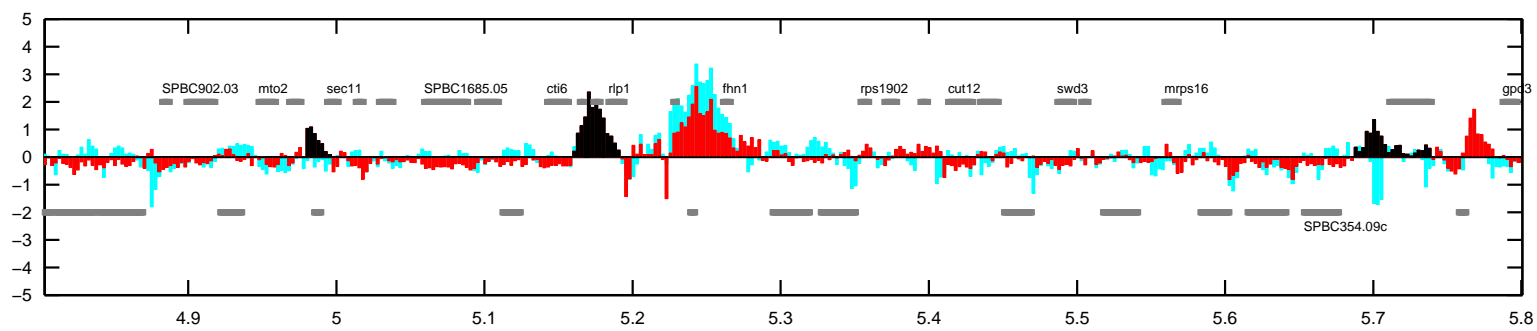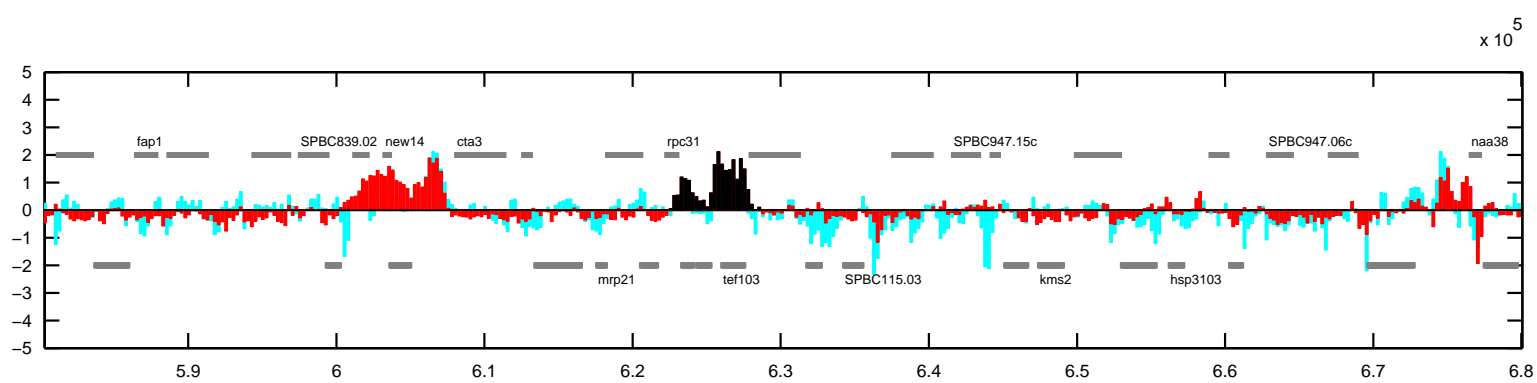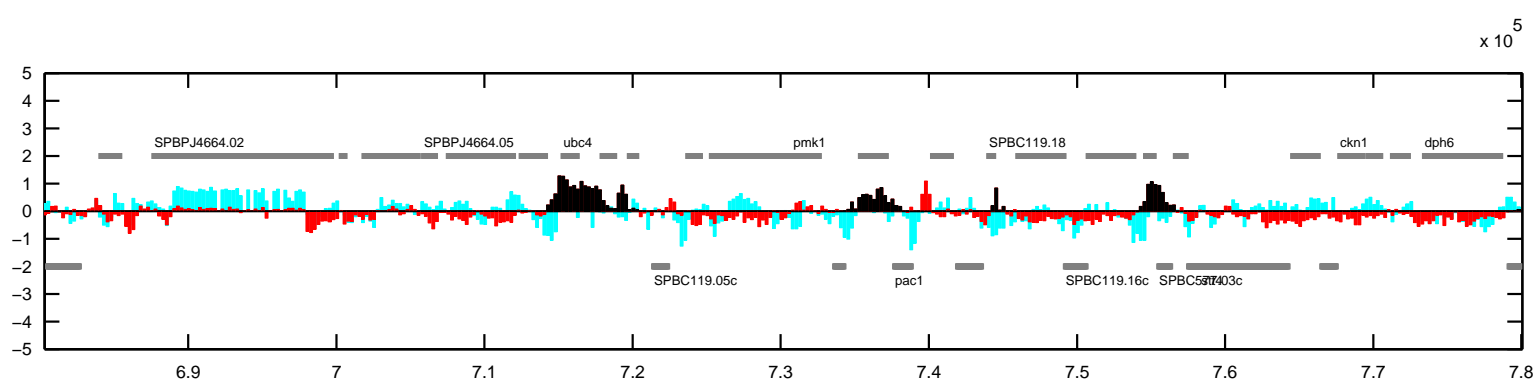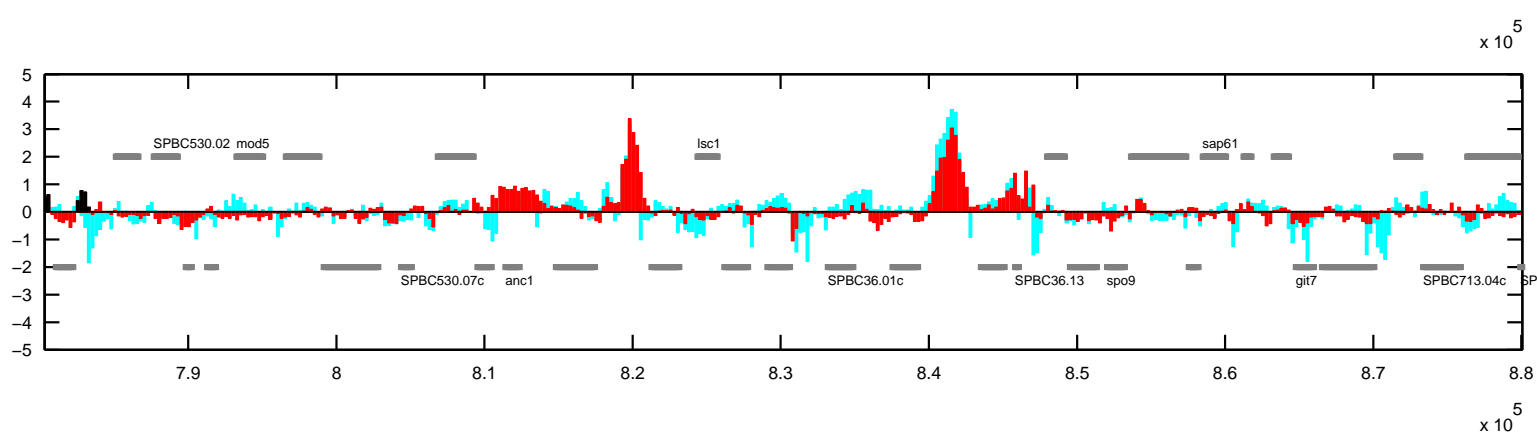

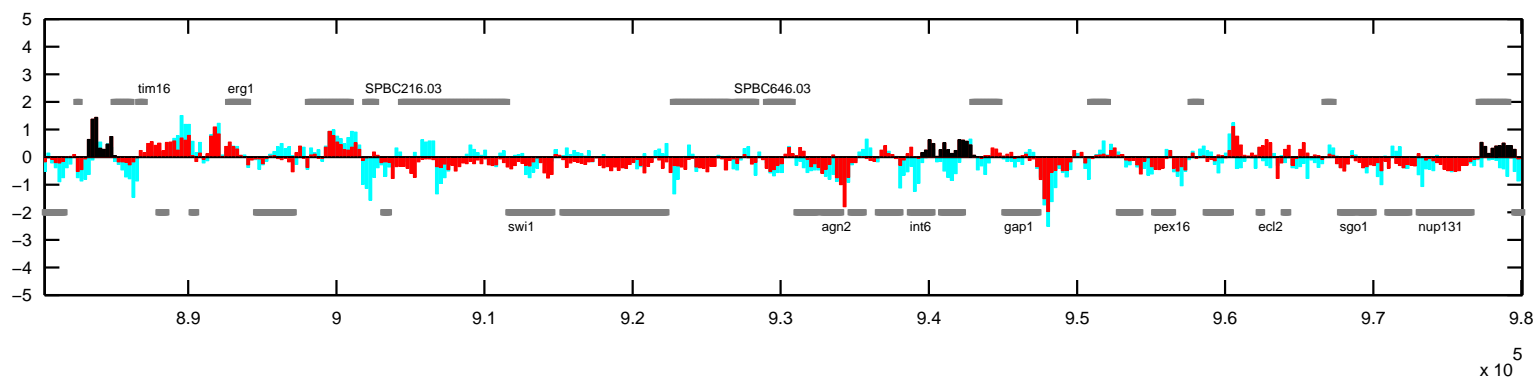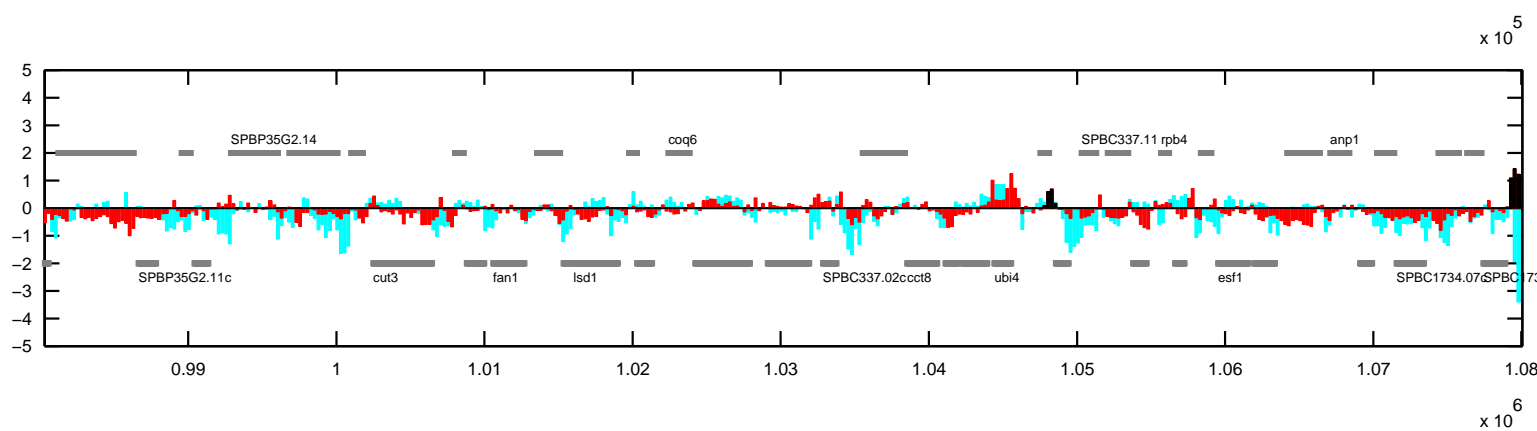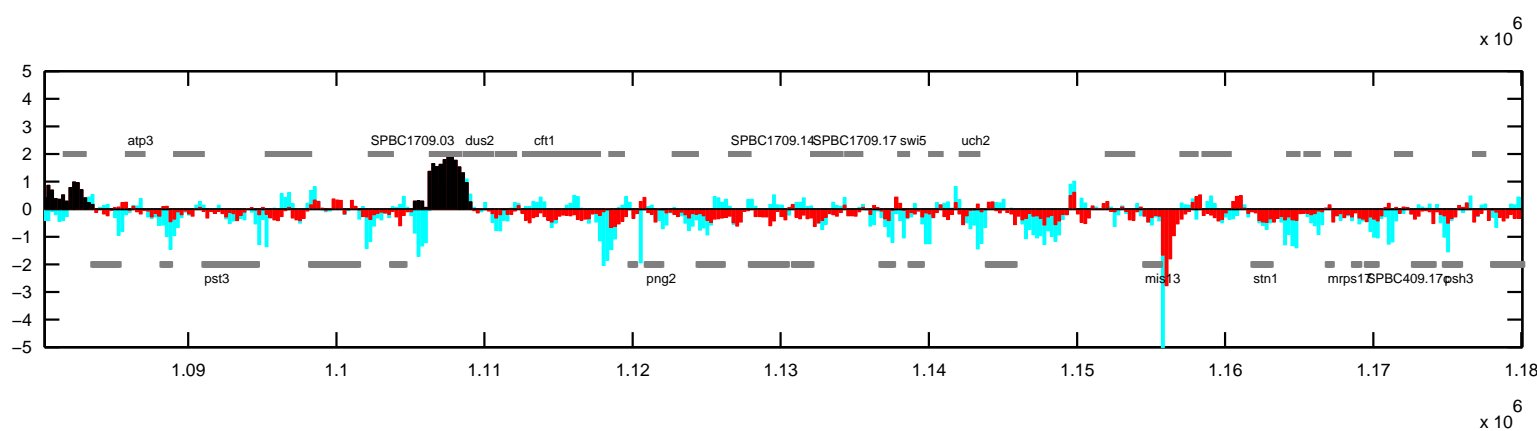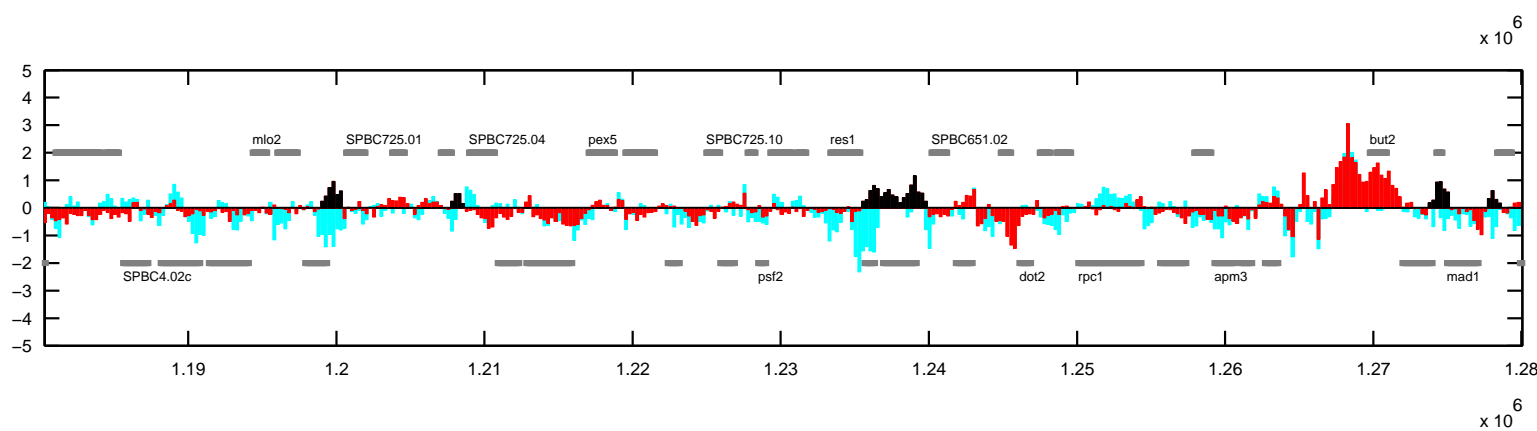

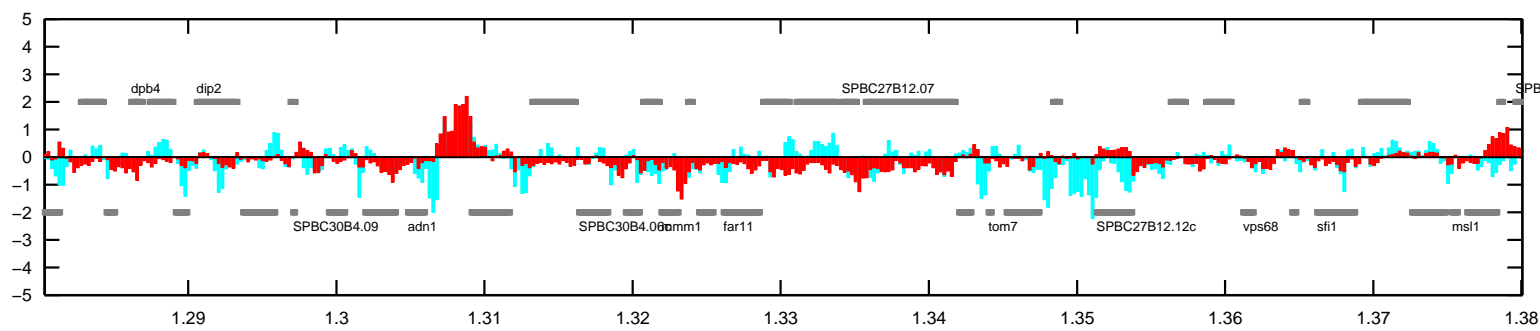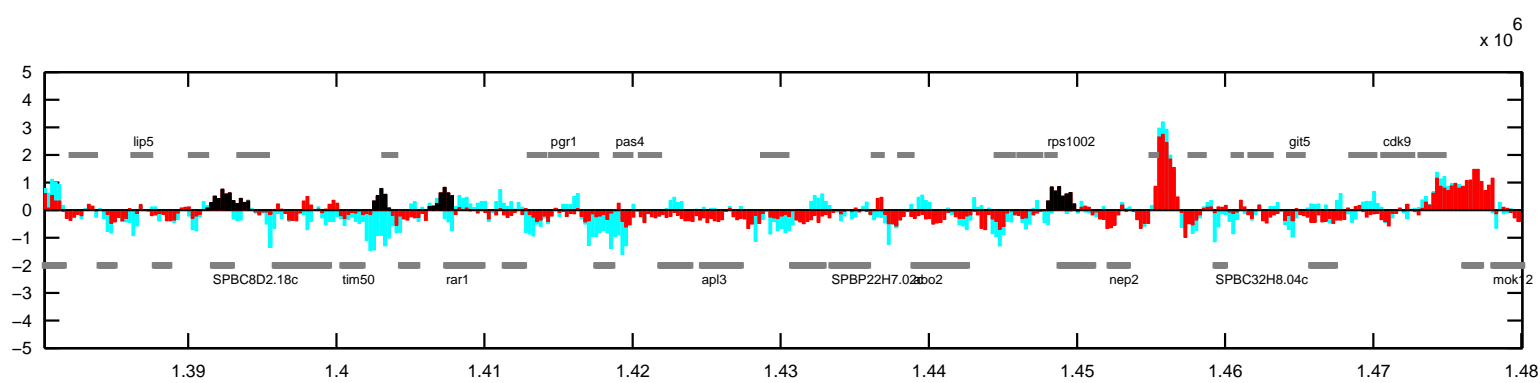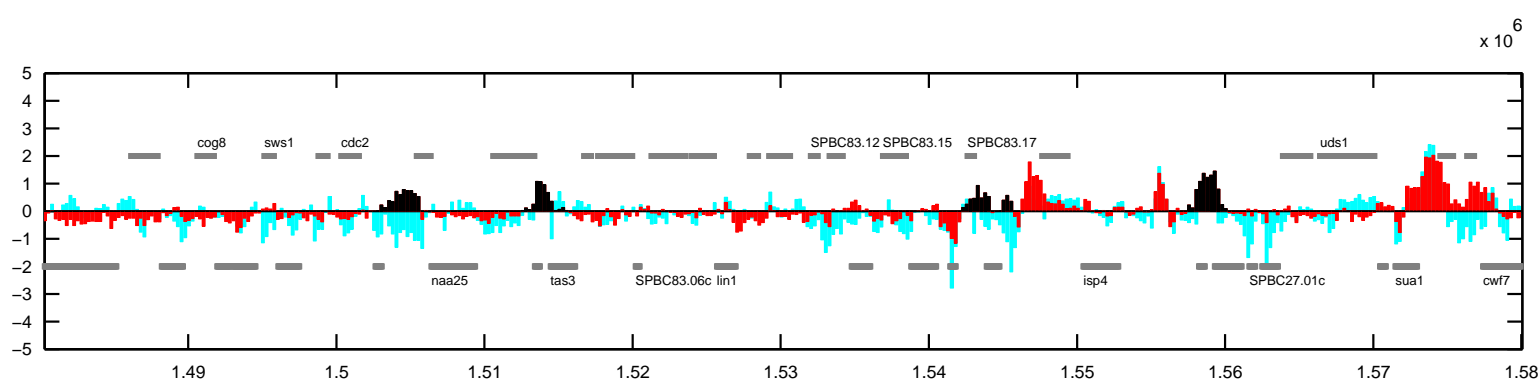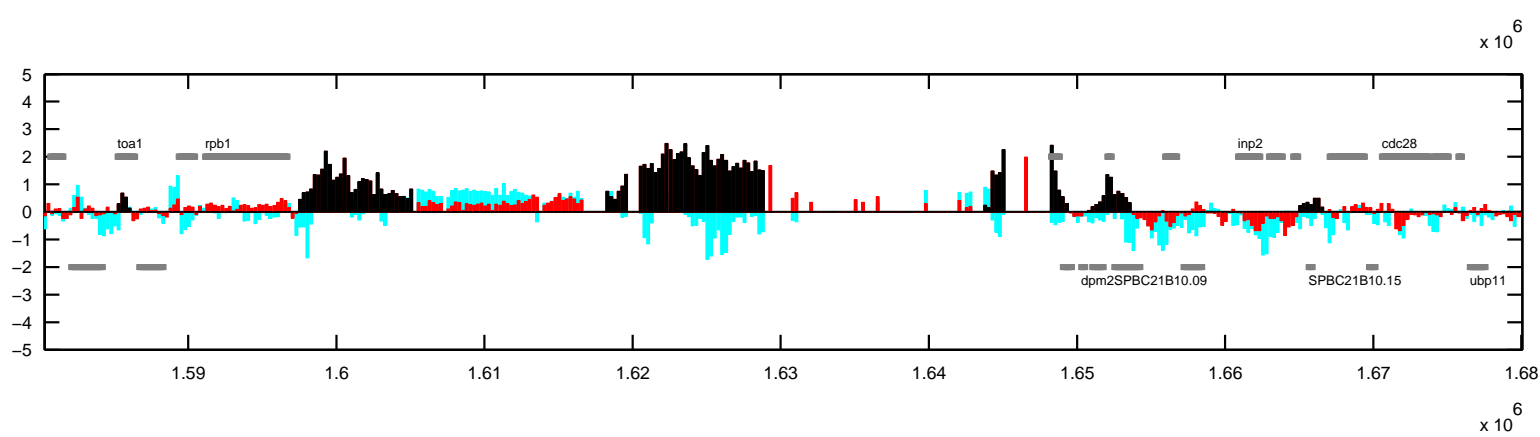

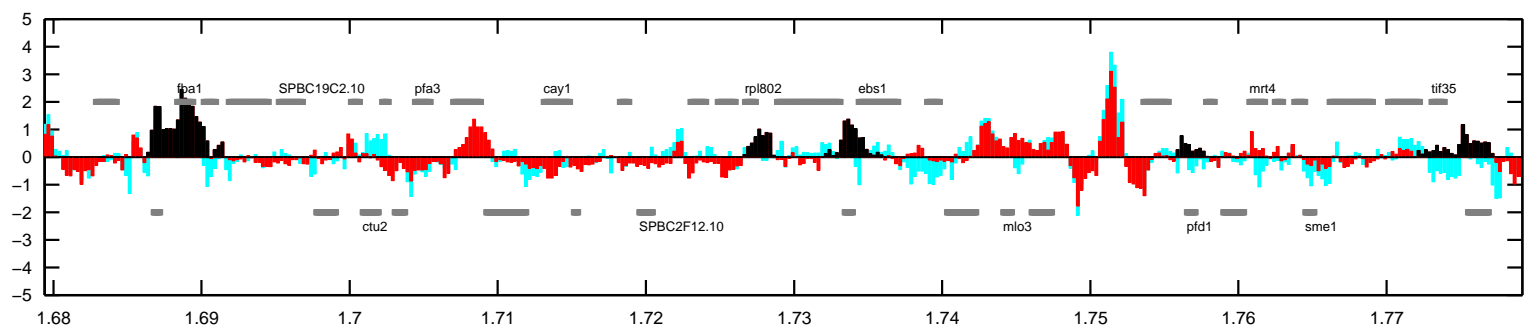

$\times 10^6$

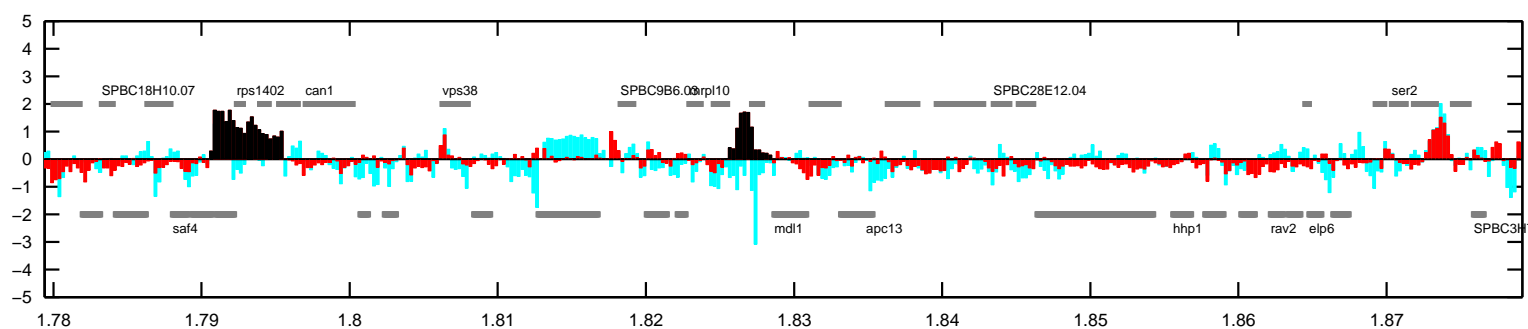

$\times 10^6$

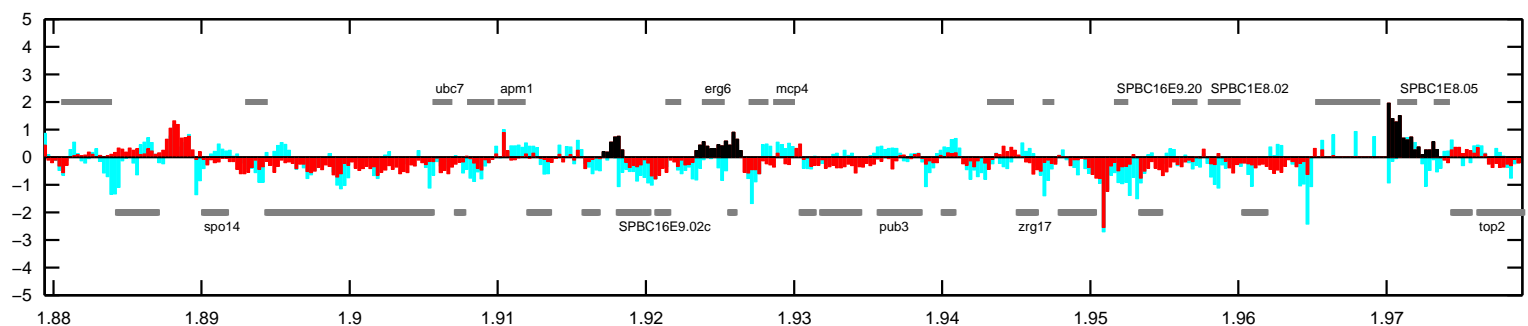

$\times 10^6$

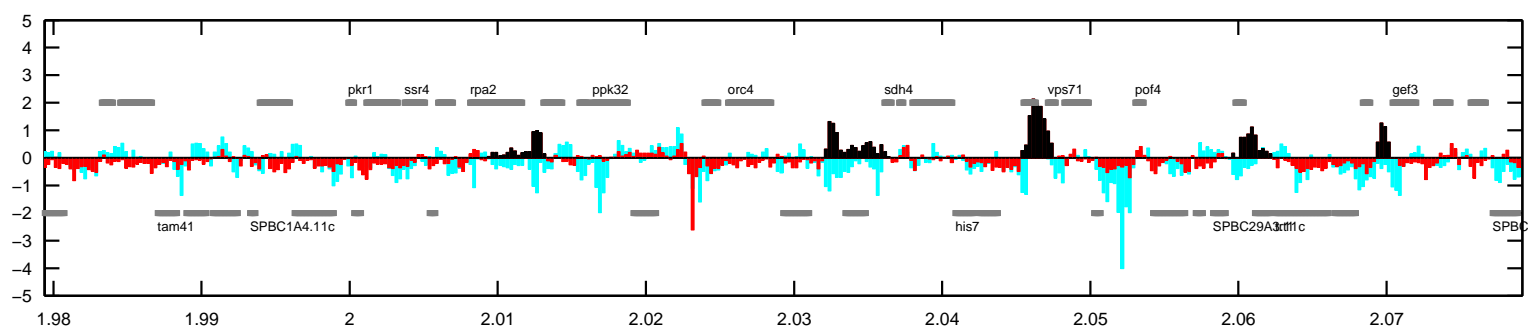

$\times 10^6$

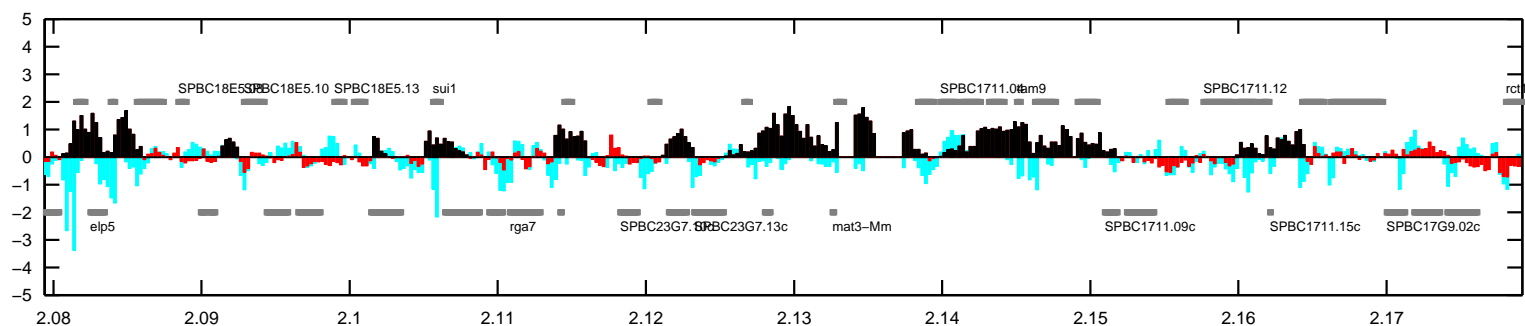

$\times 10^6$

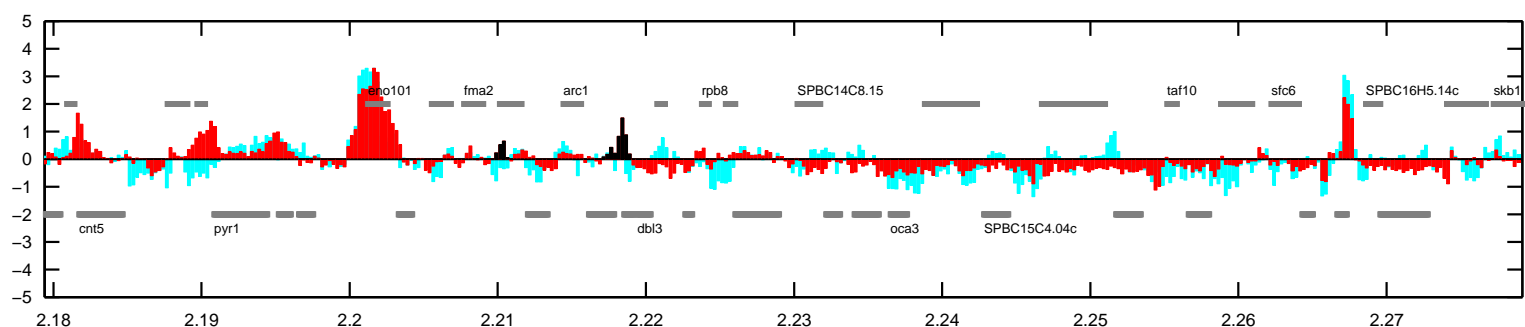

$\times 10^6$

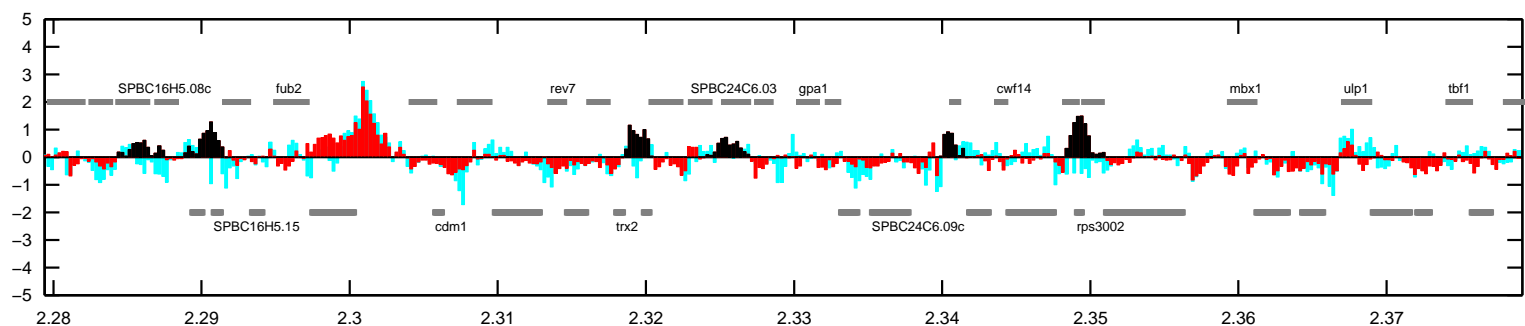

$\times 10^6$

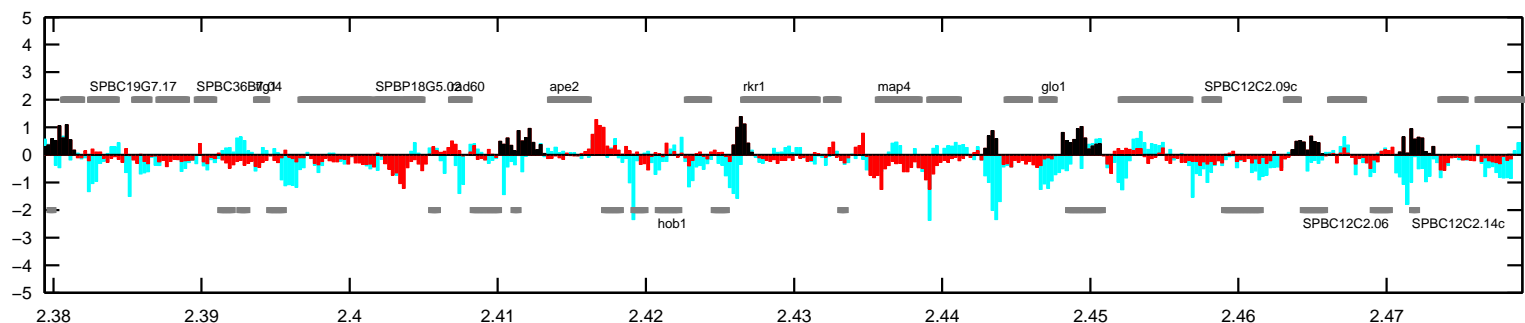

$\times 10^6$

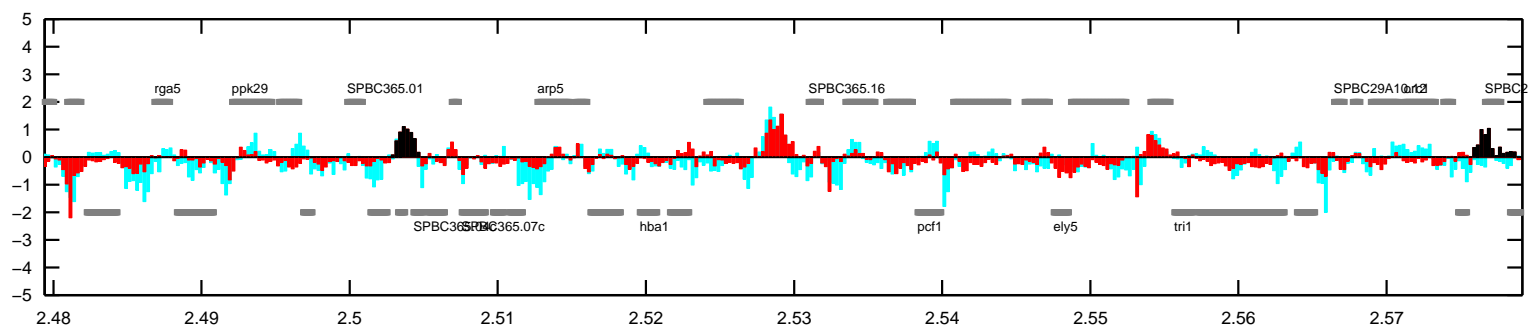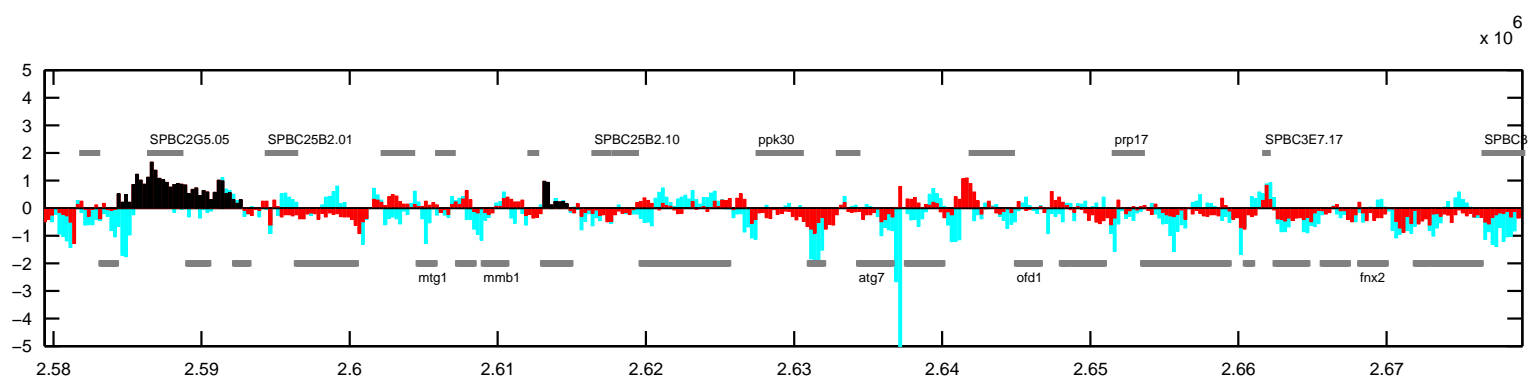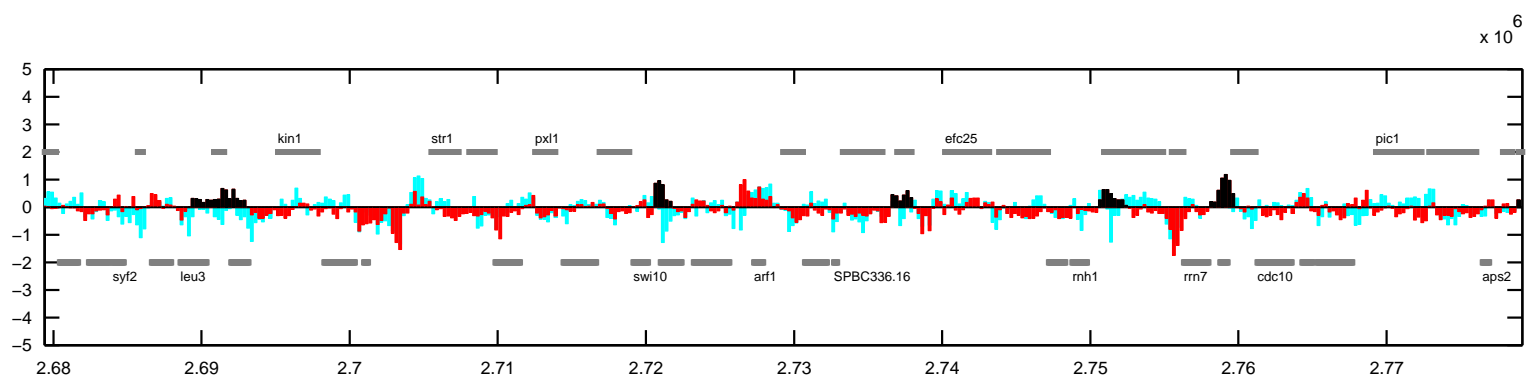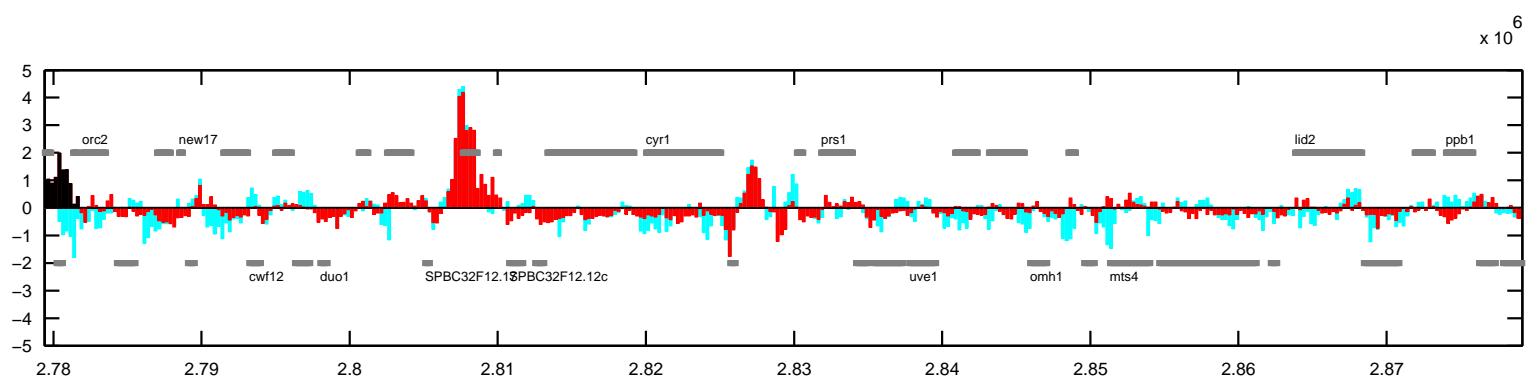

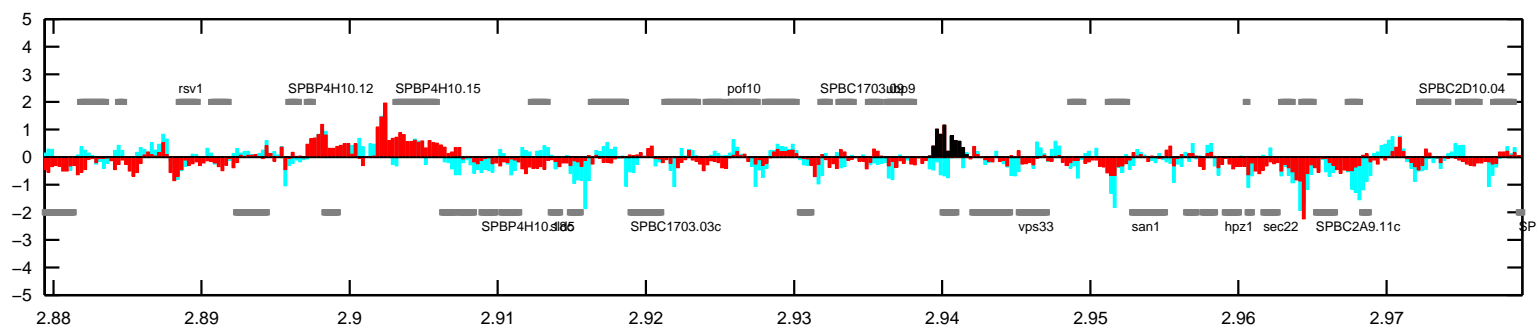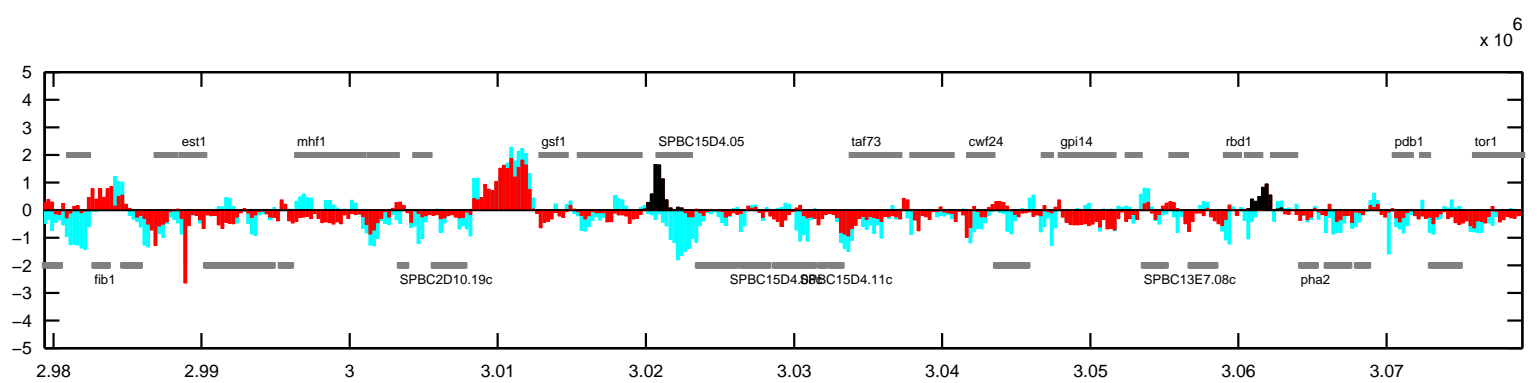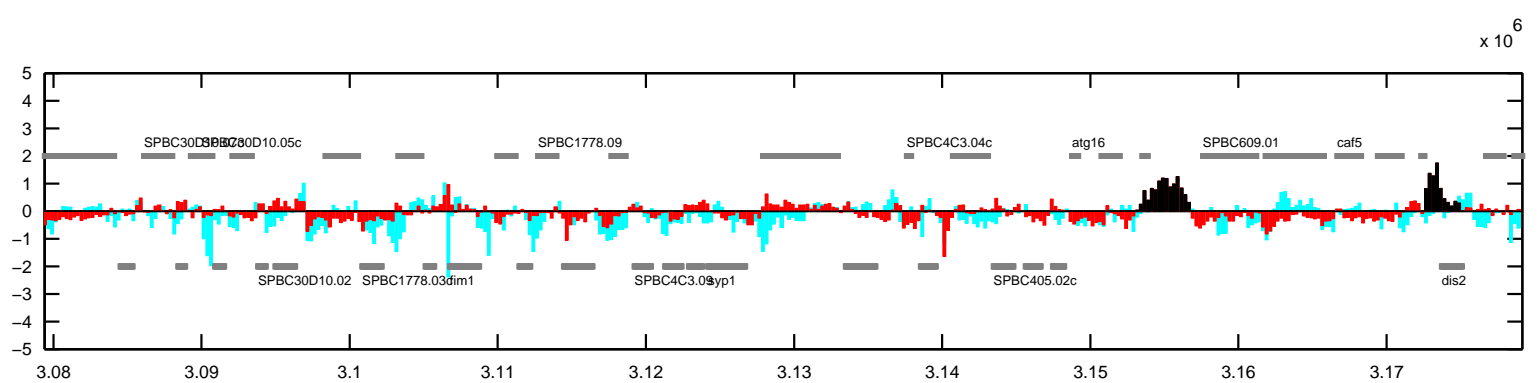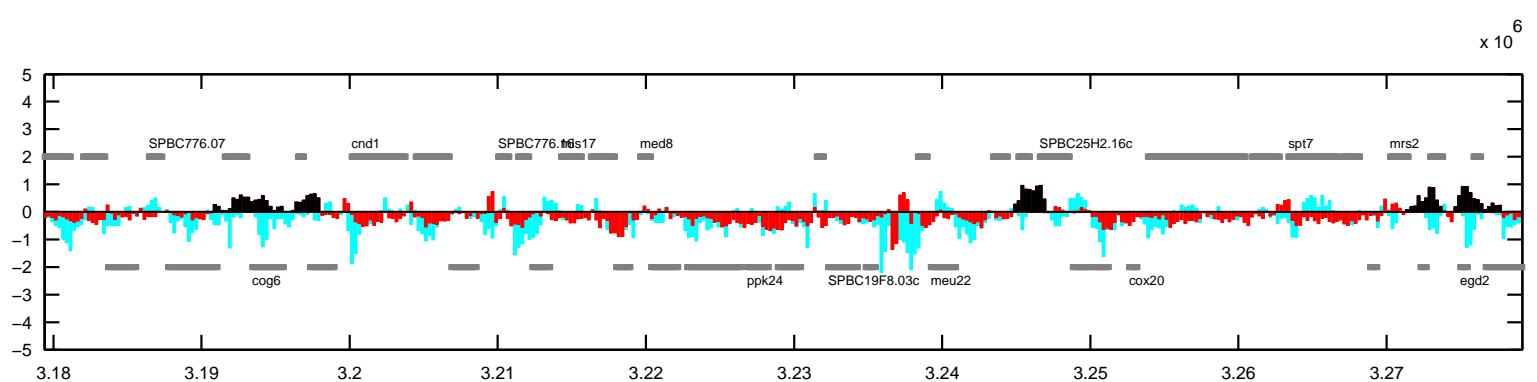

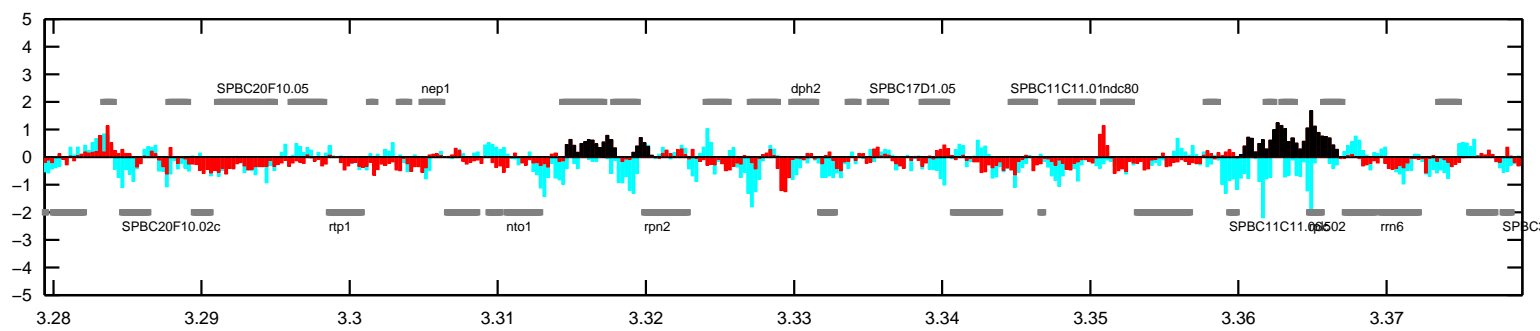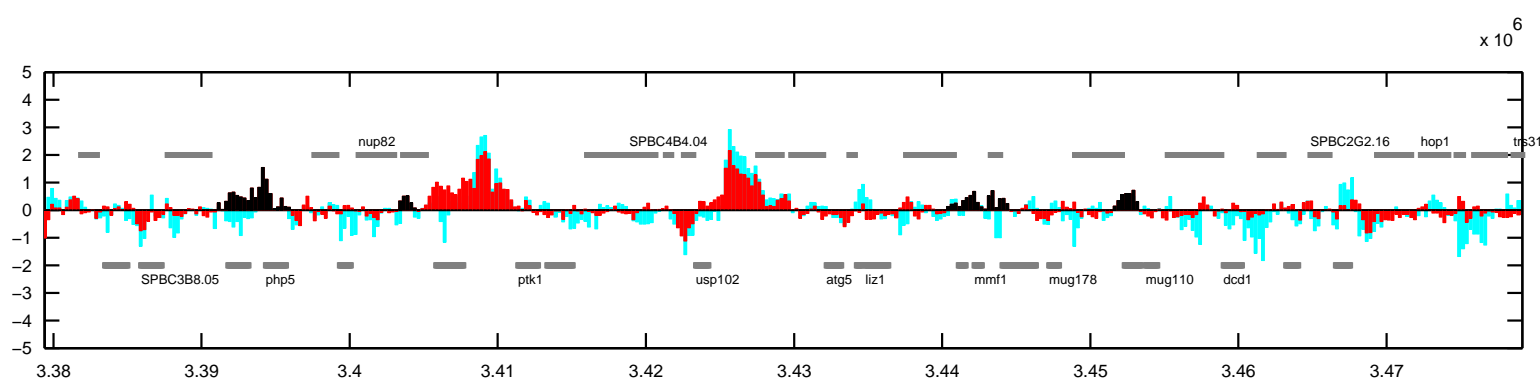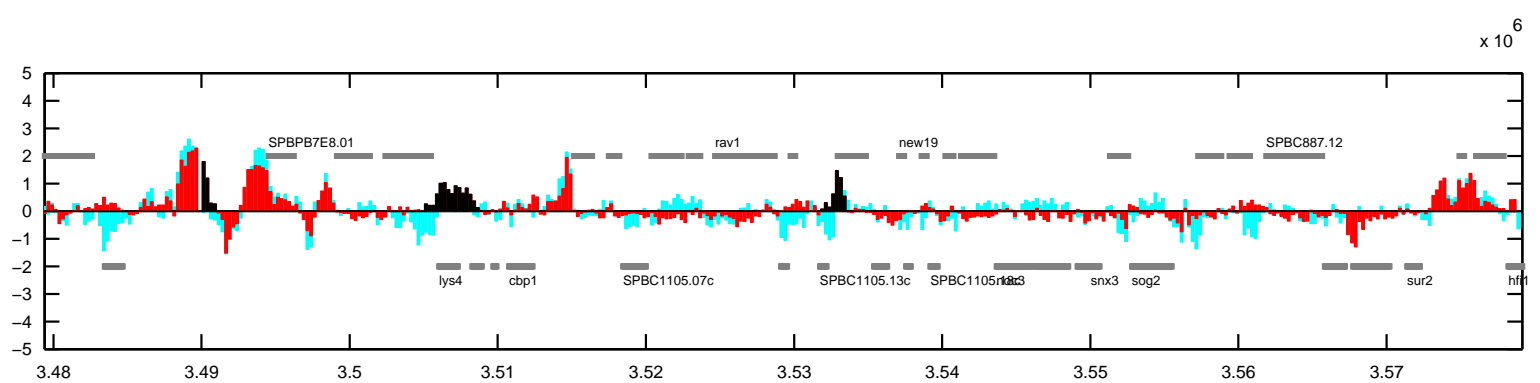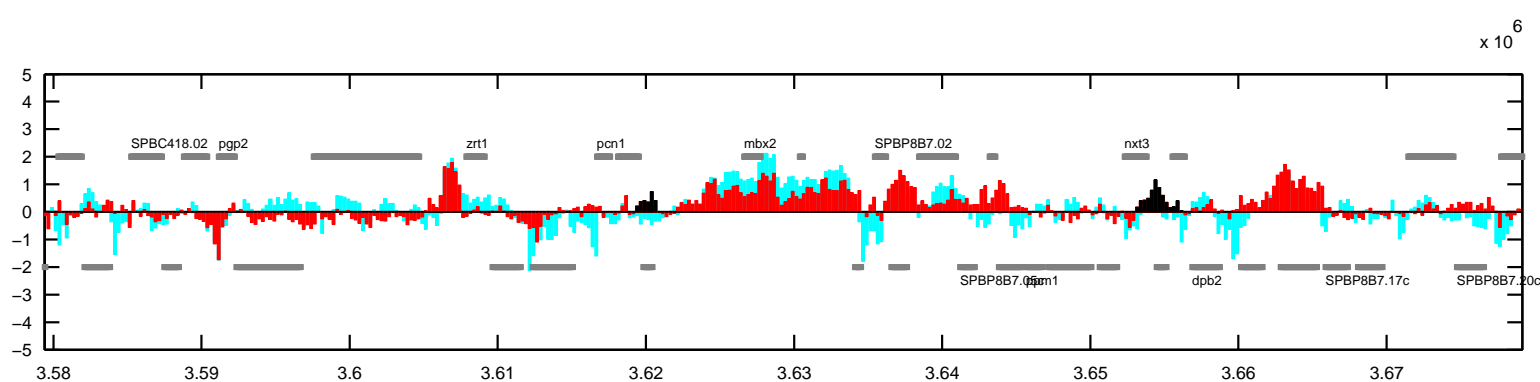

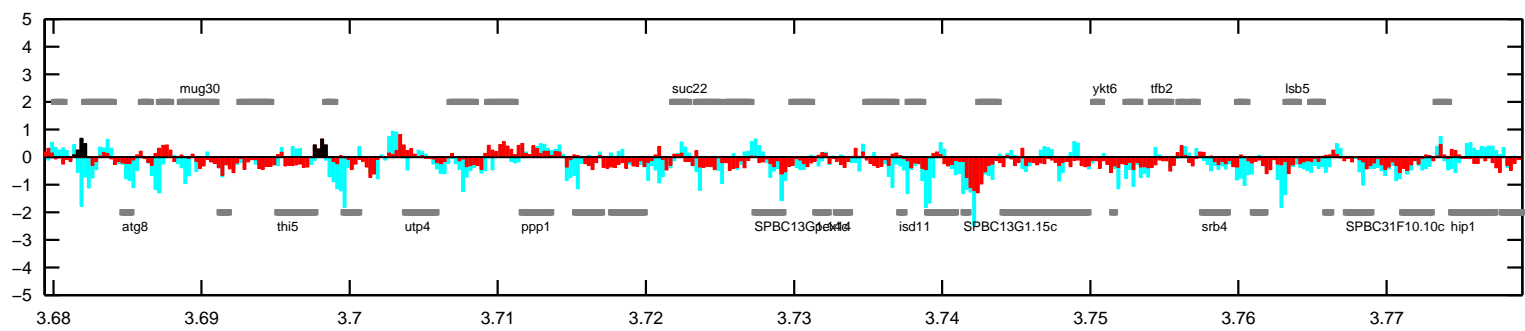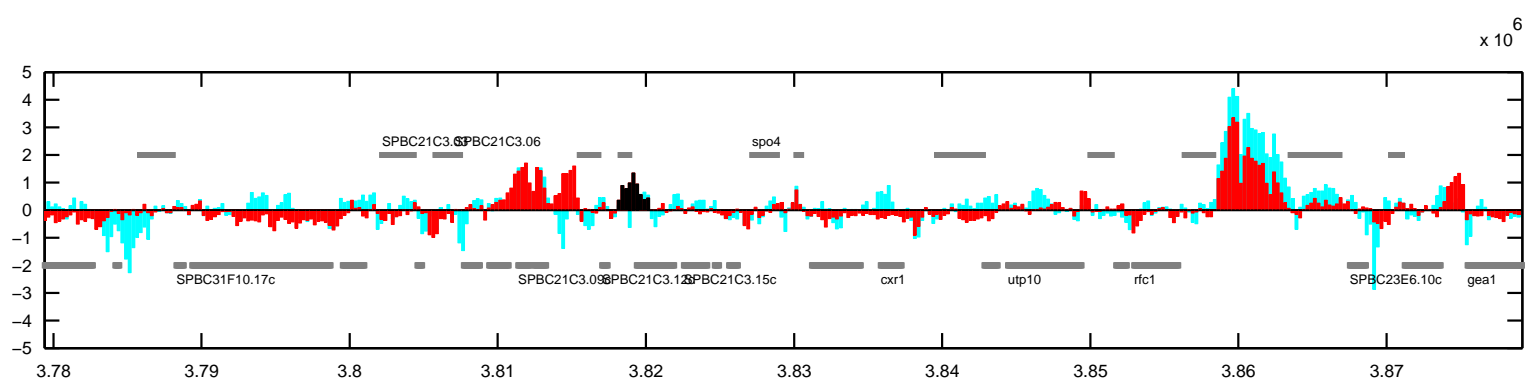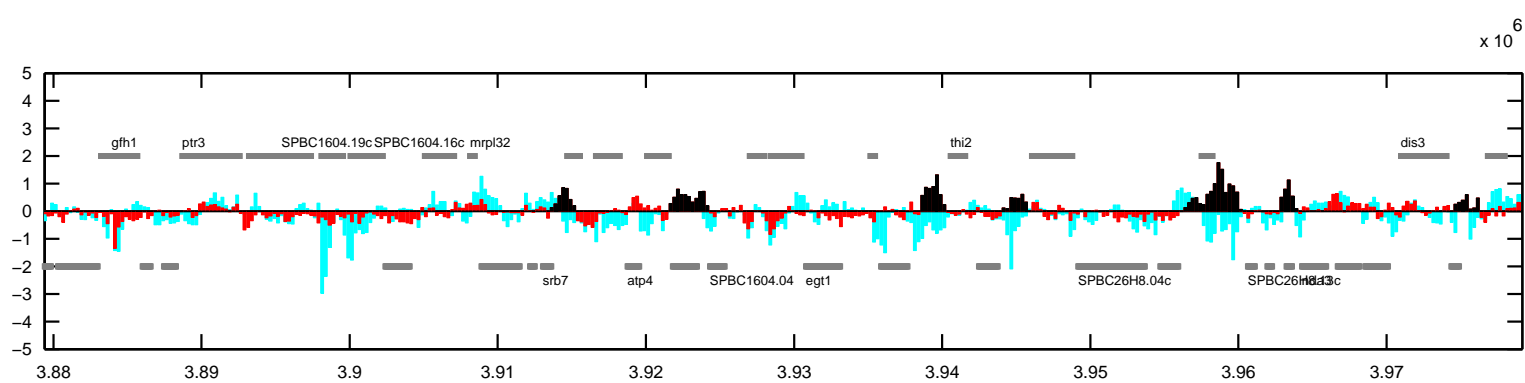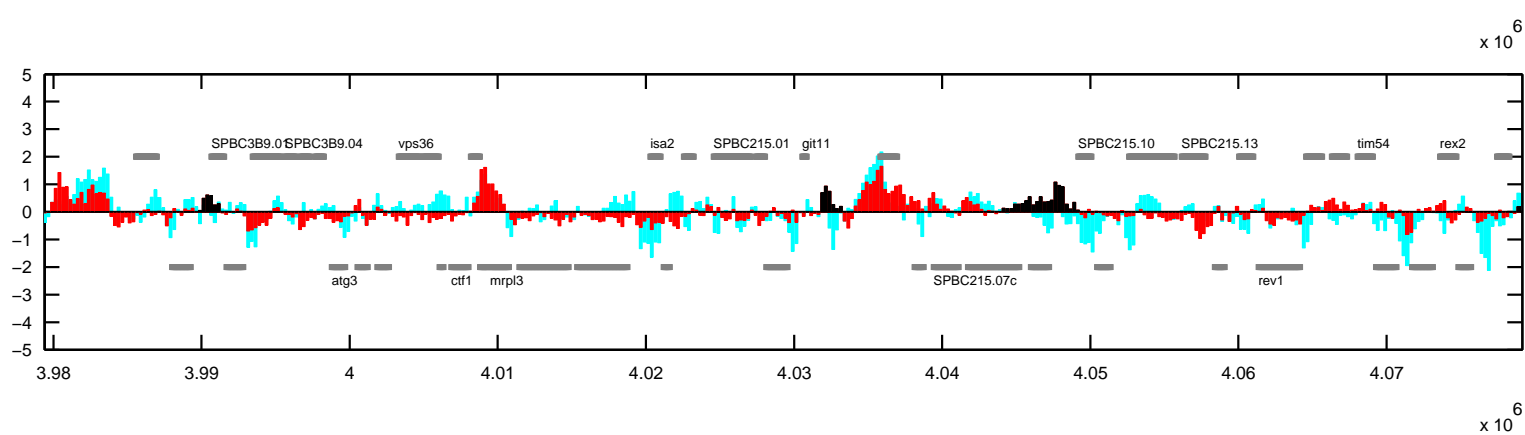

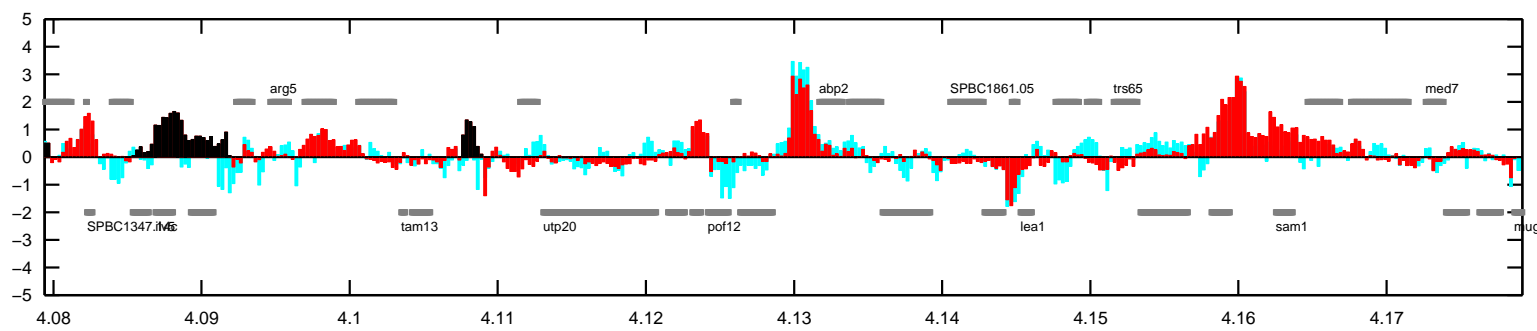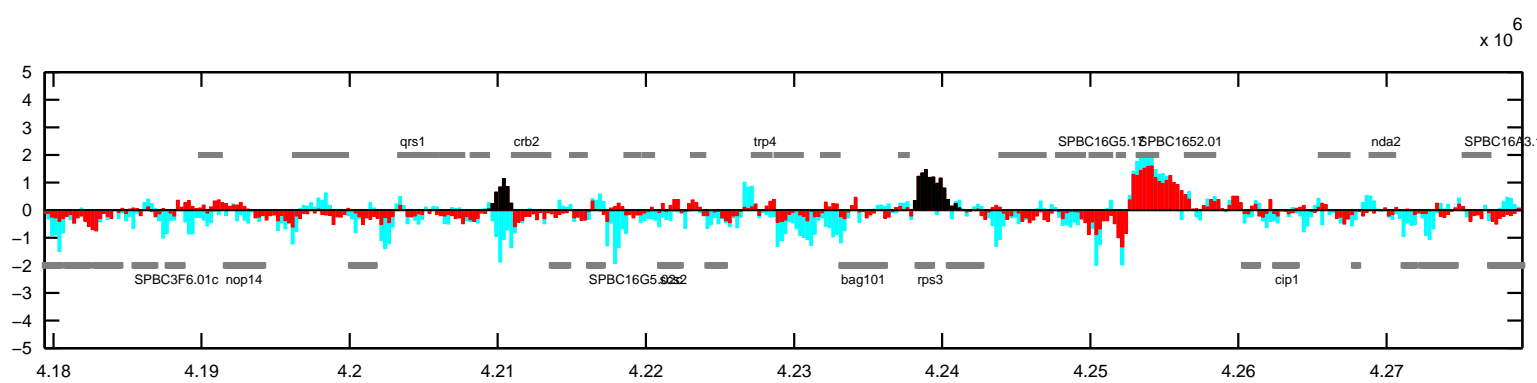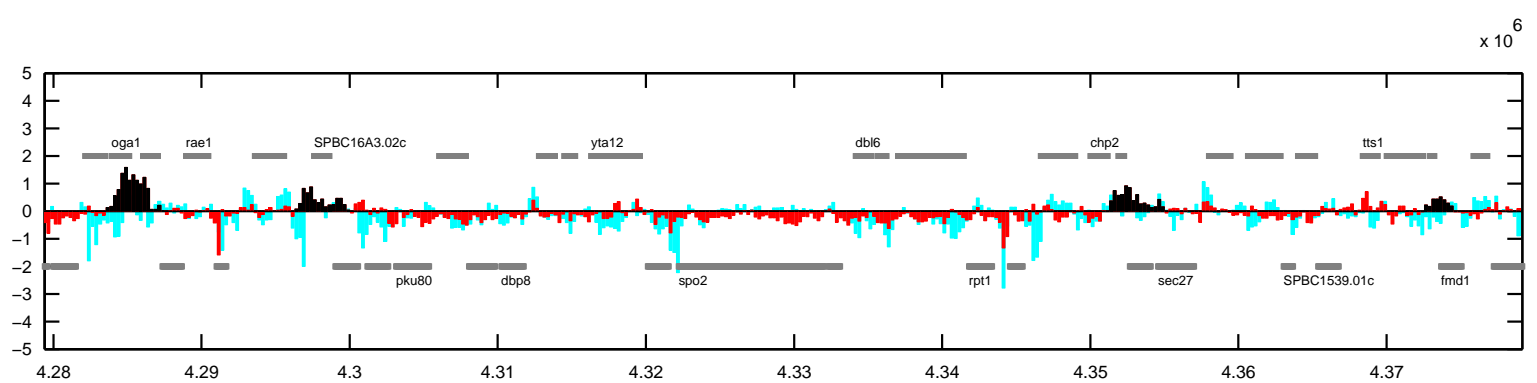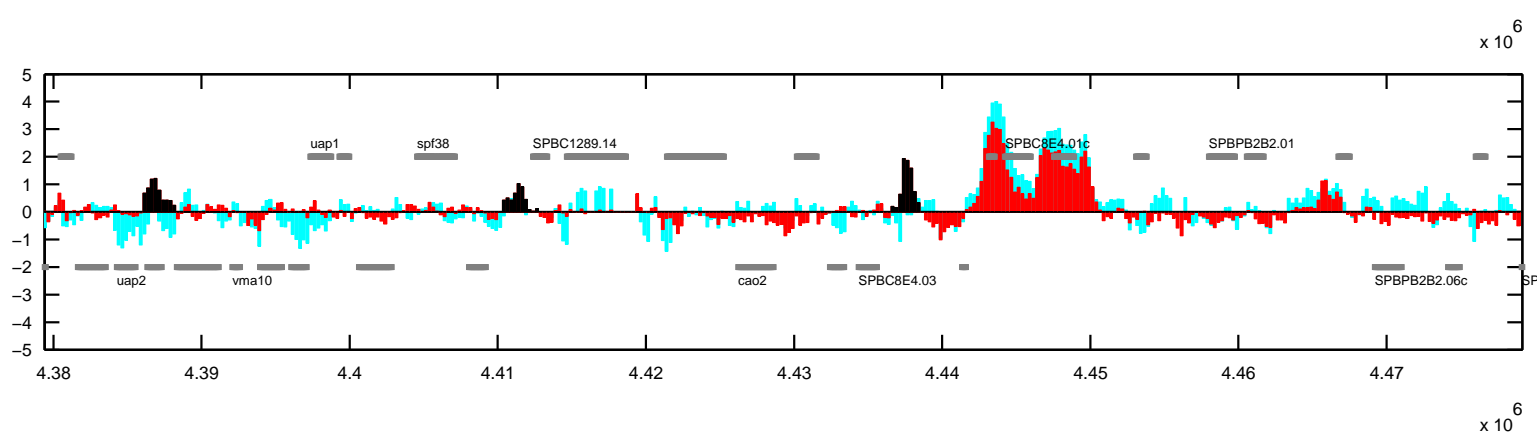

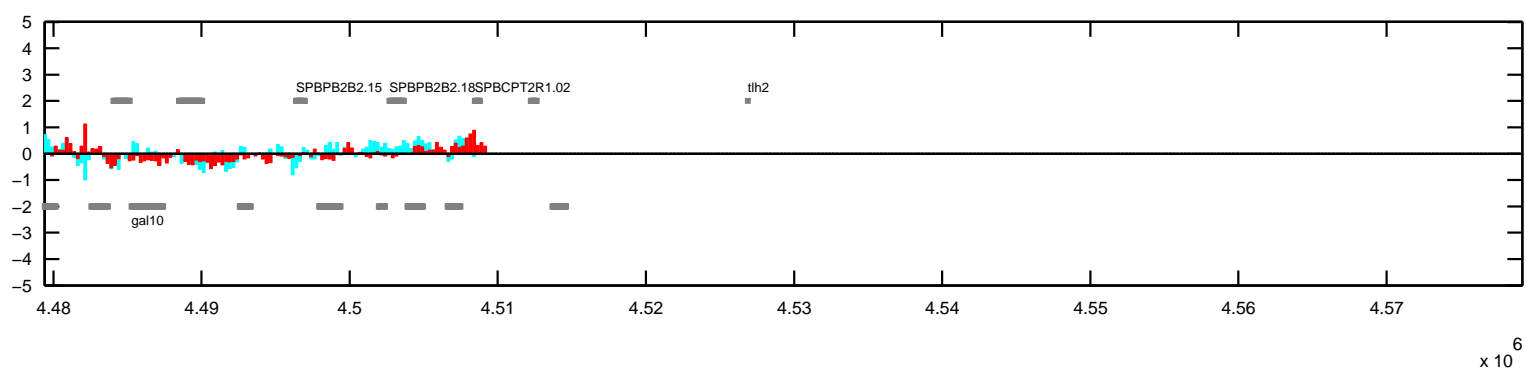

Supplement: SUPPLEMENTARY DATA [file supp_gkw252_nar-00155-v-2016-File006.pdf]

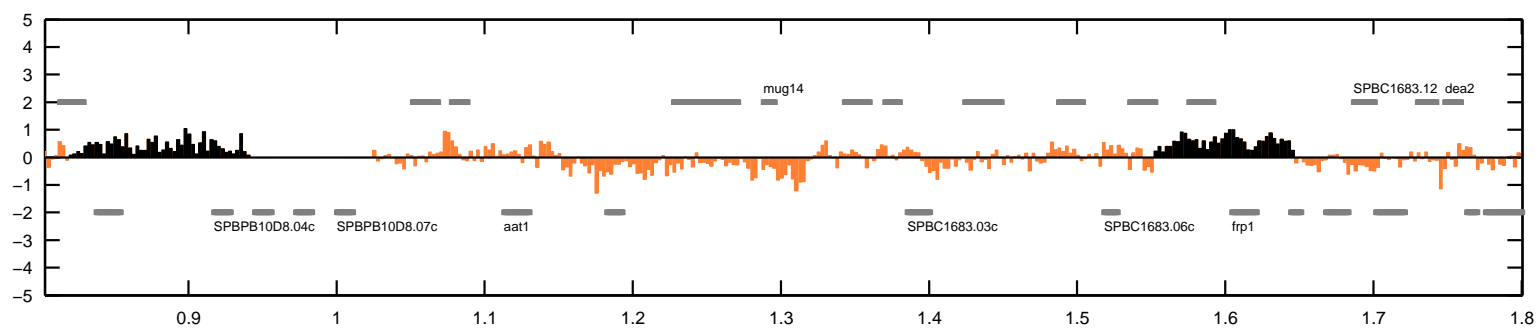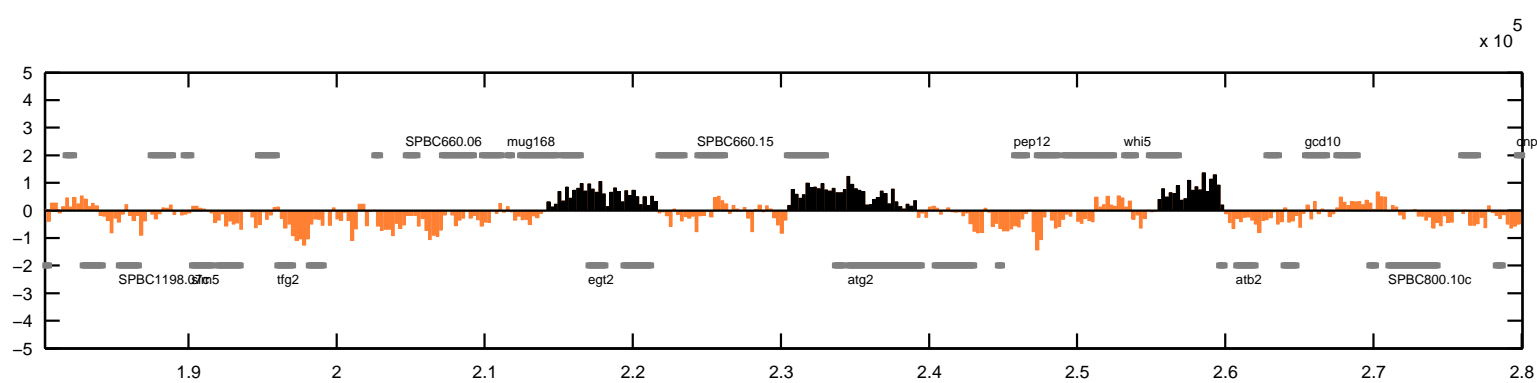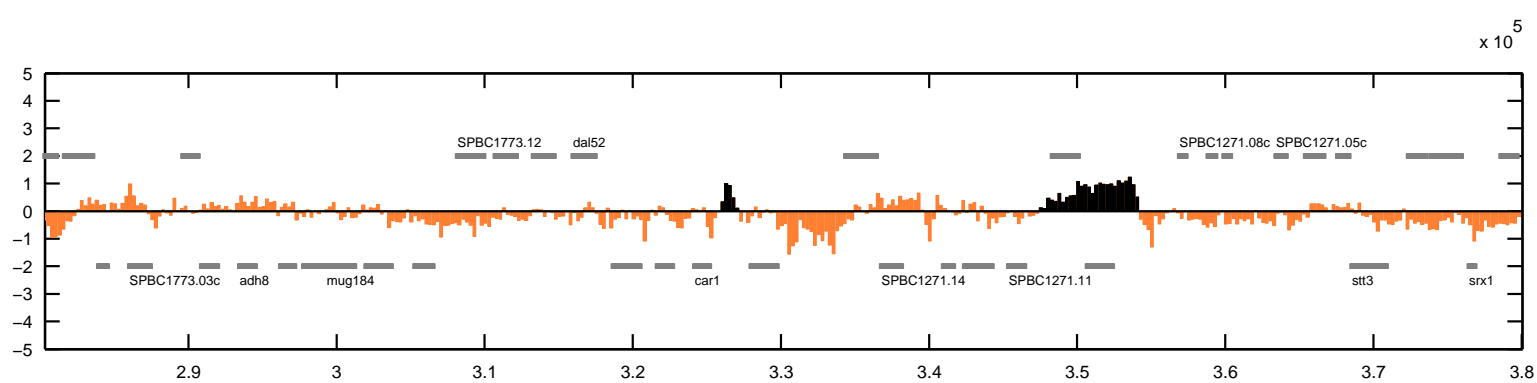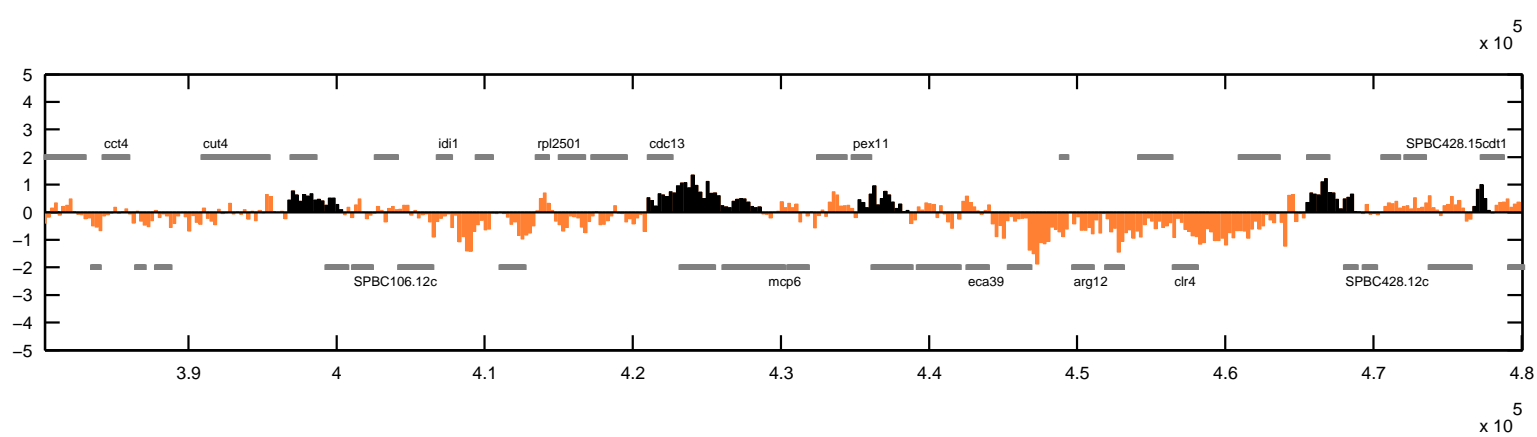

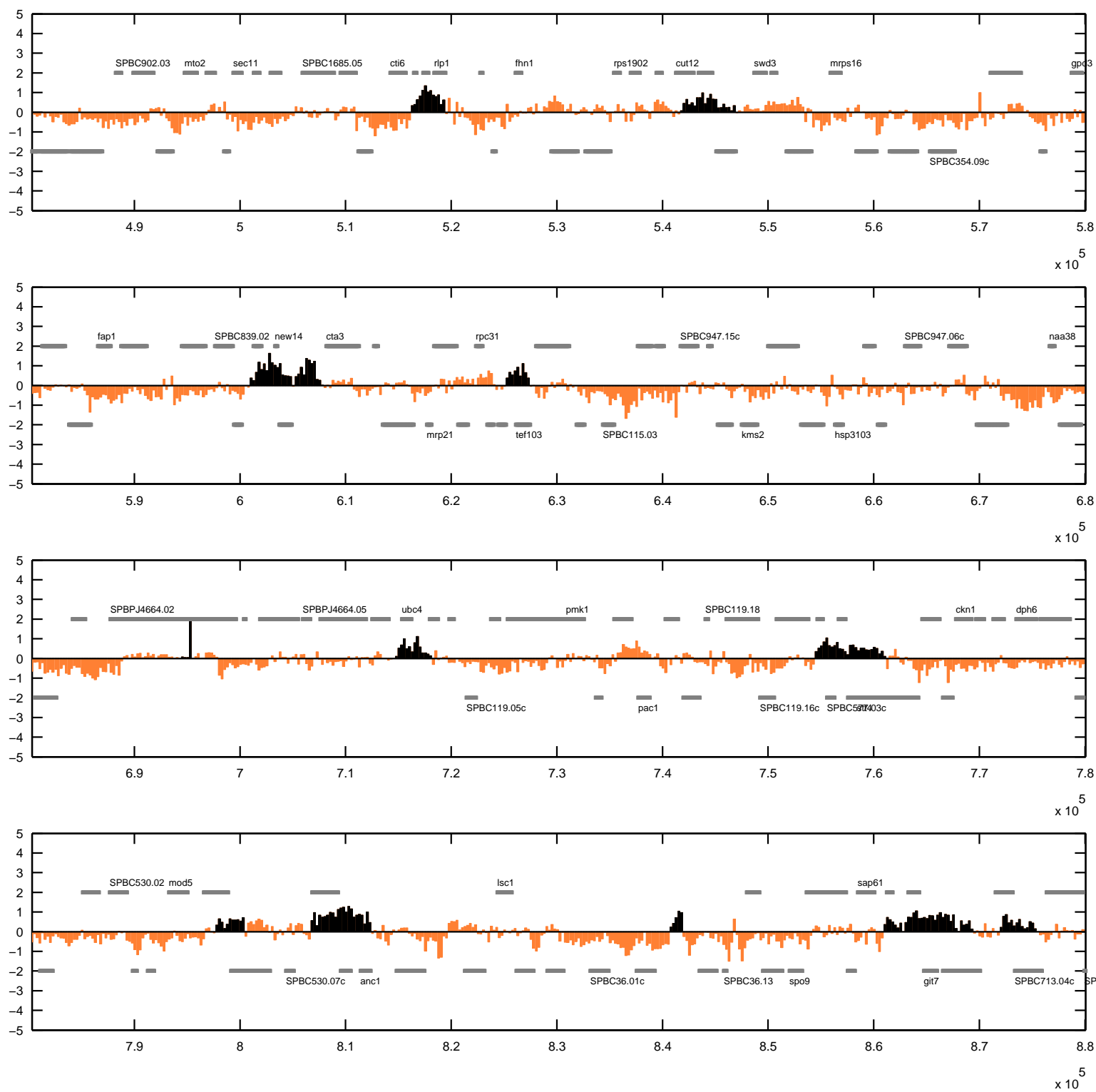

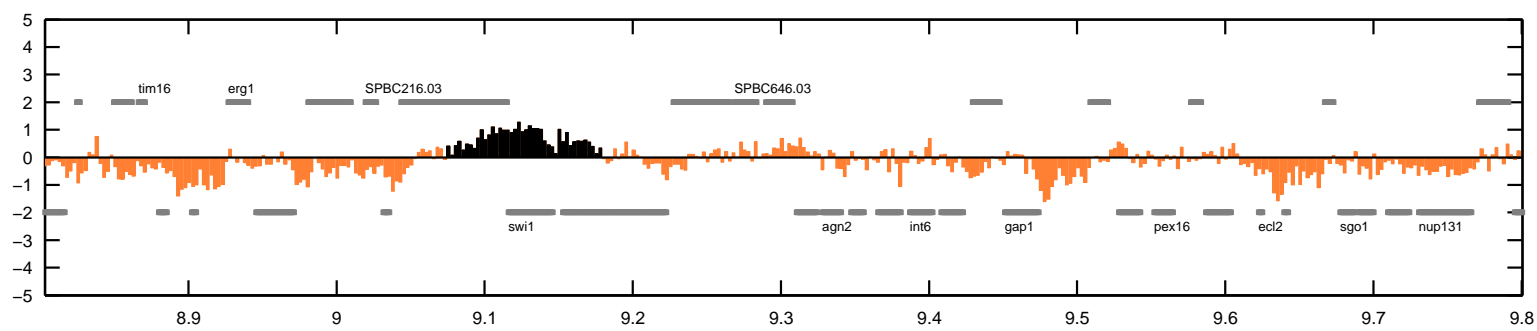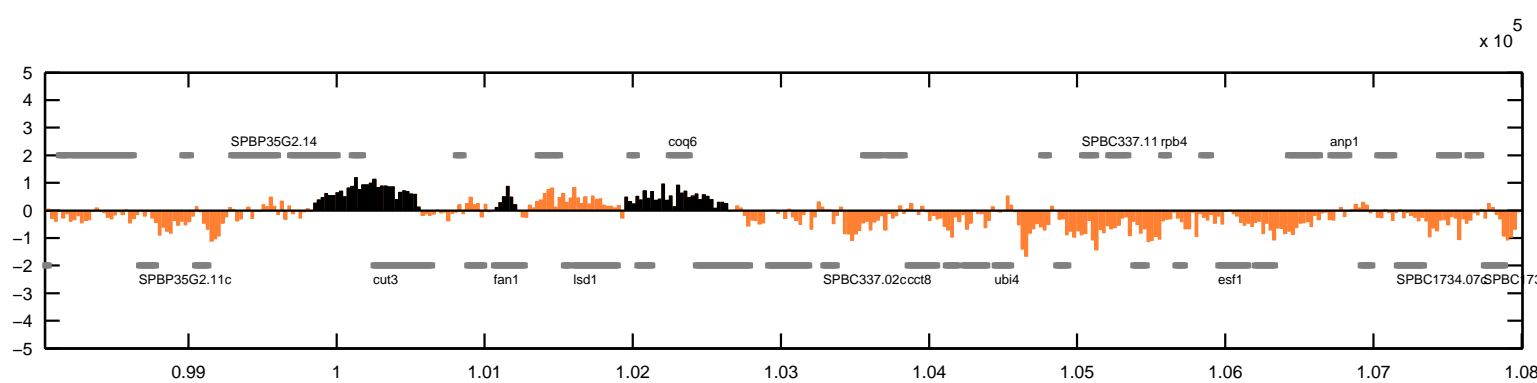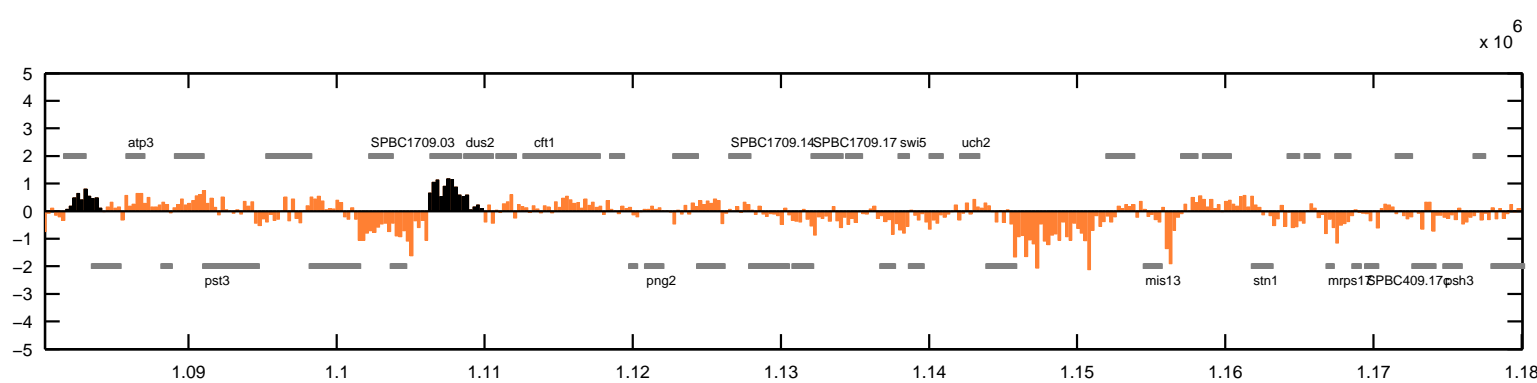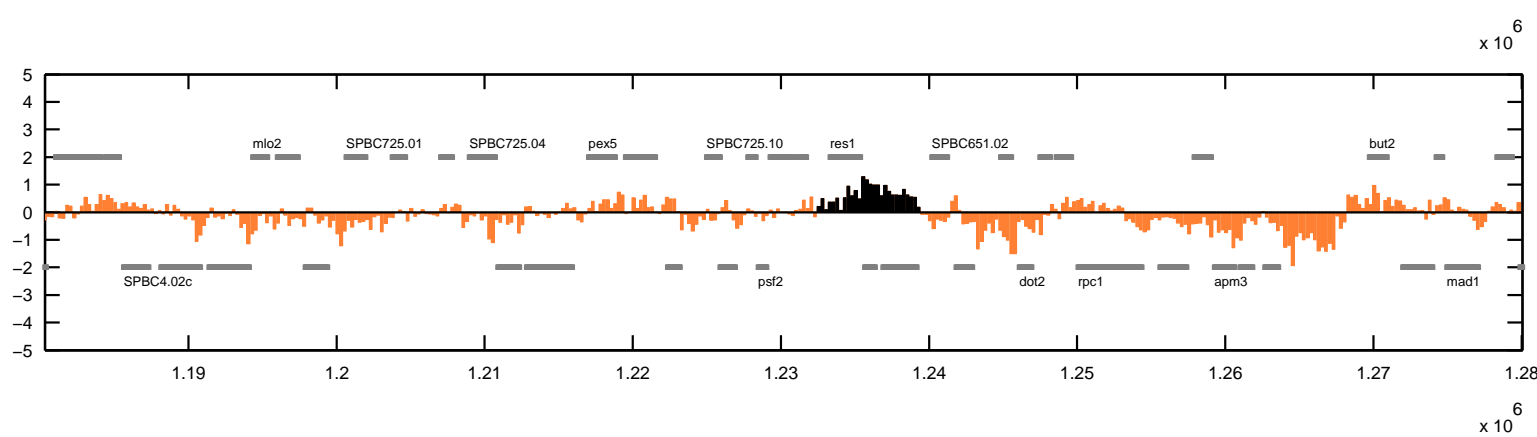

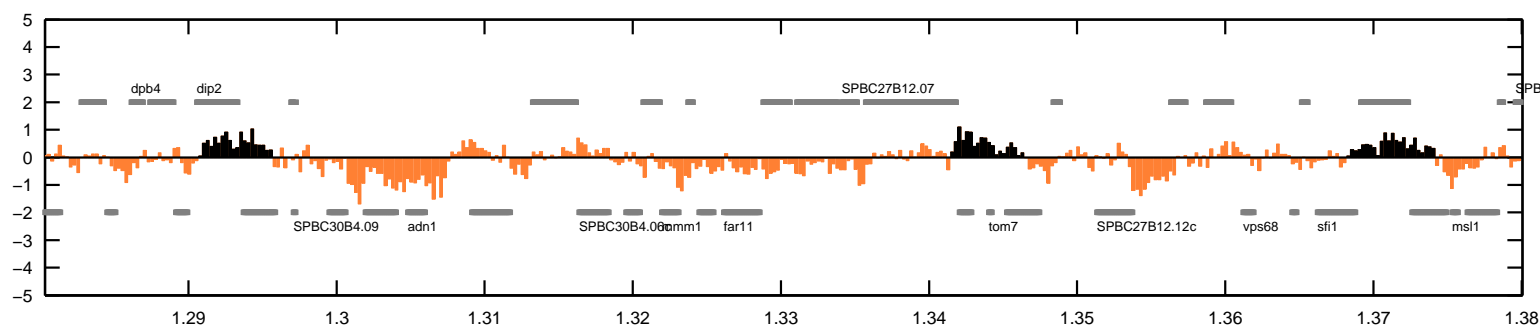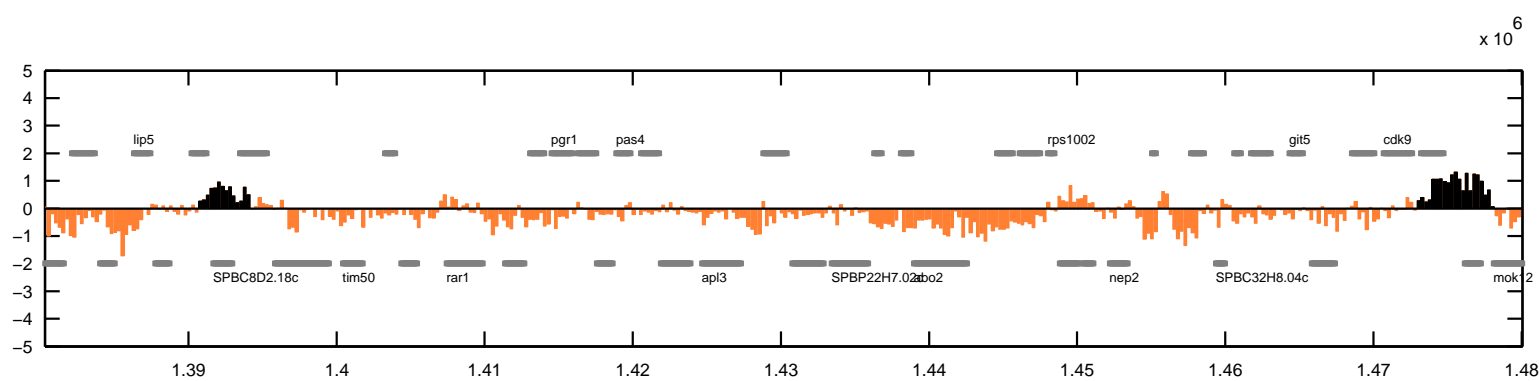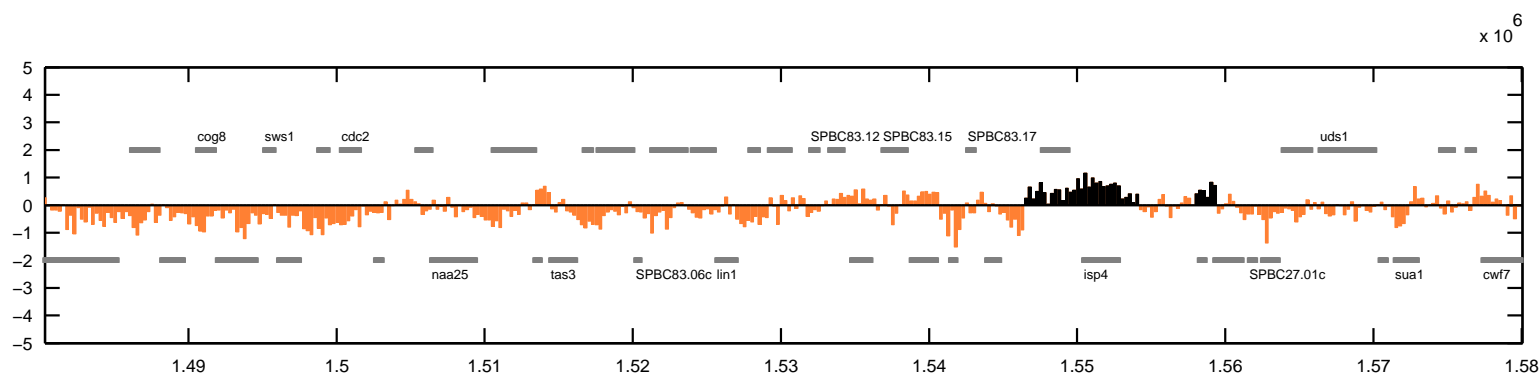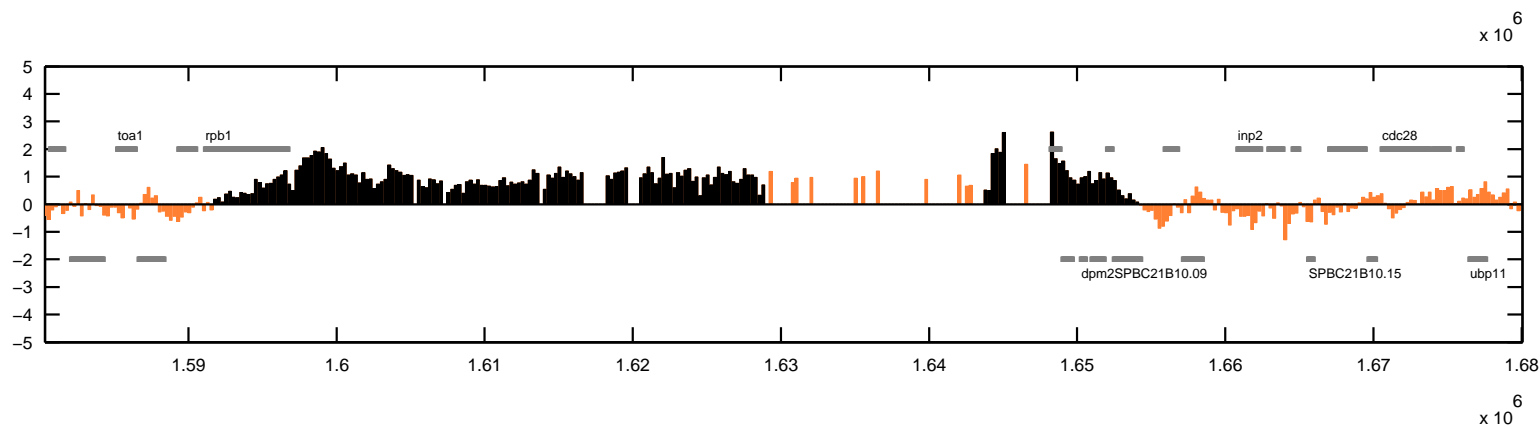

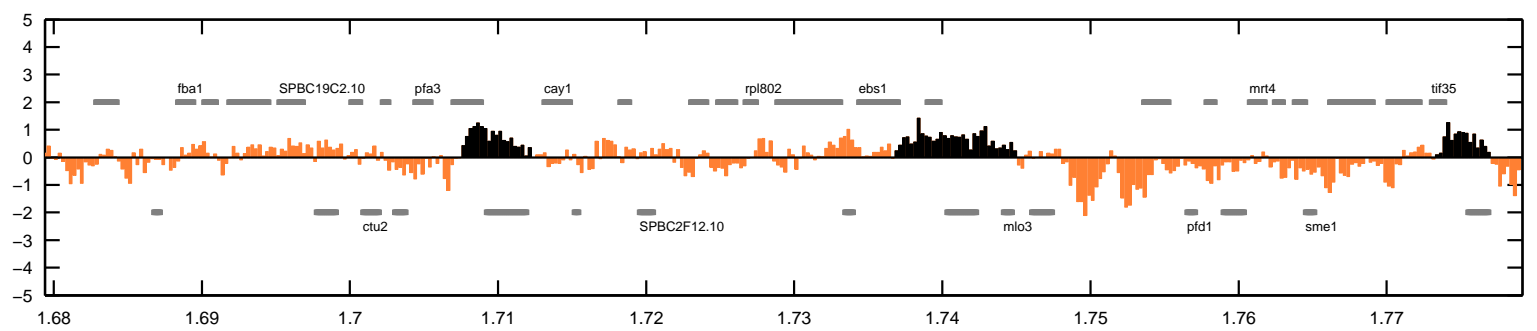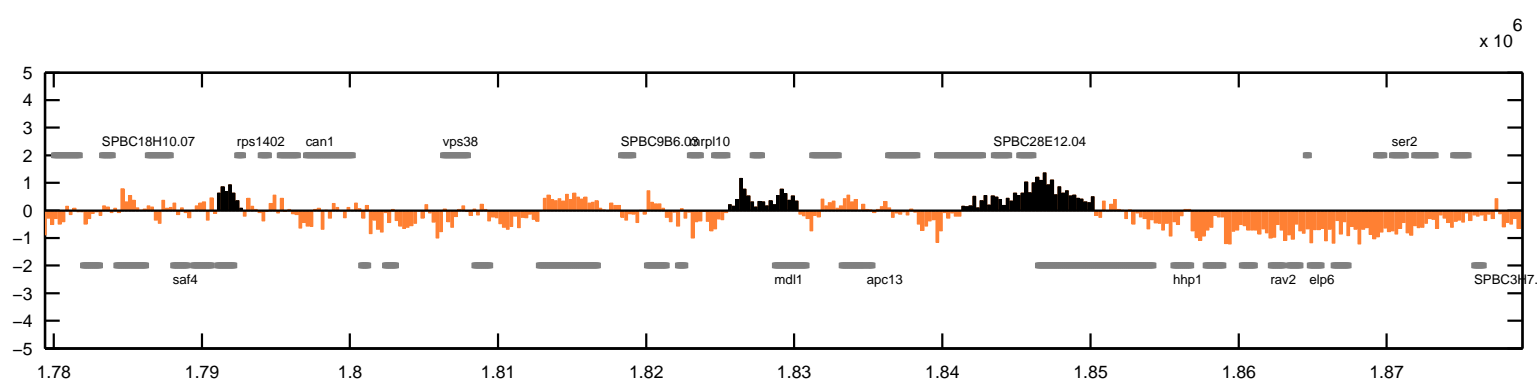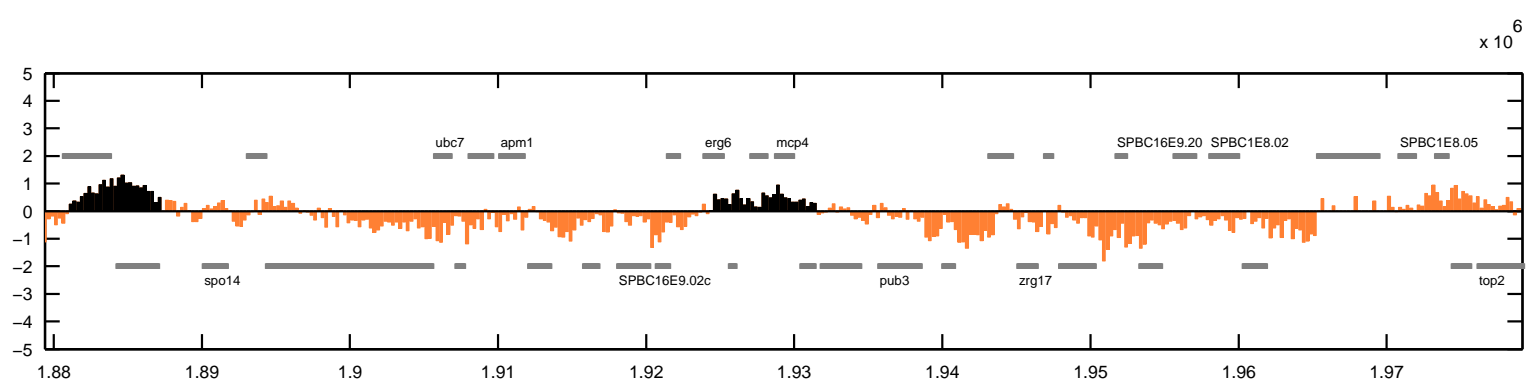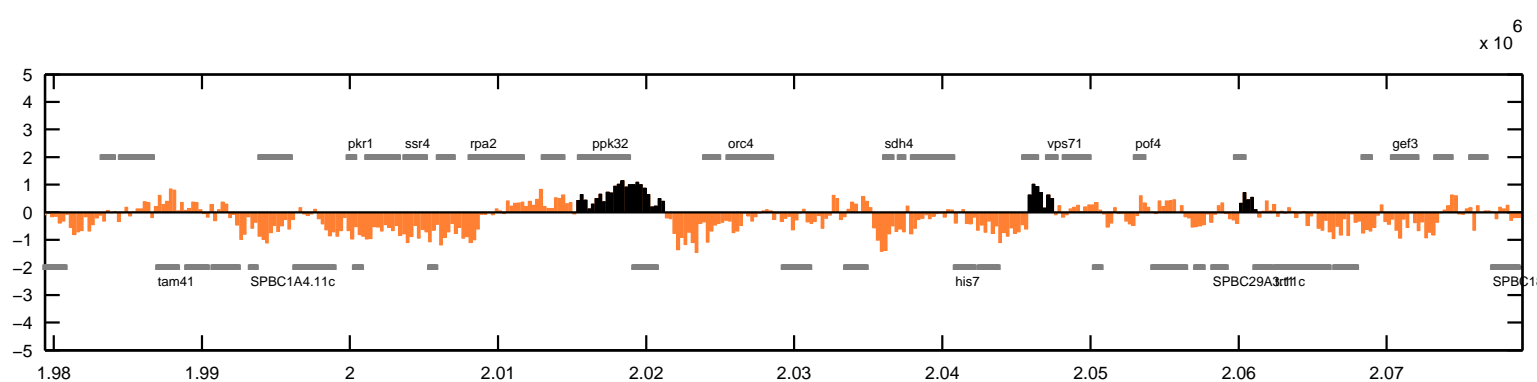

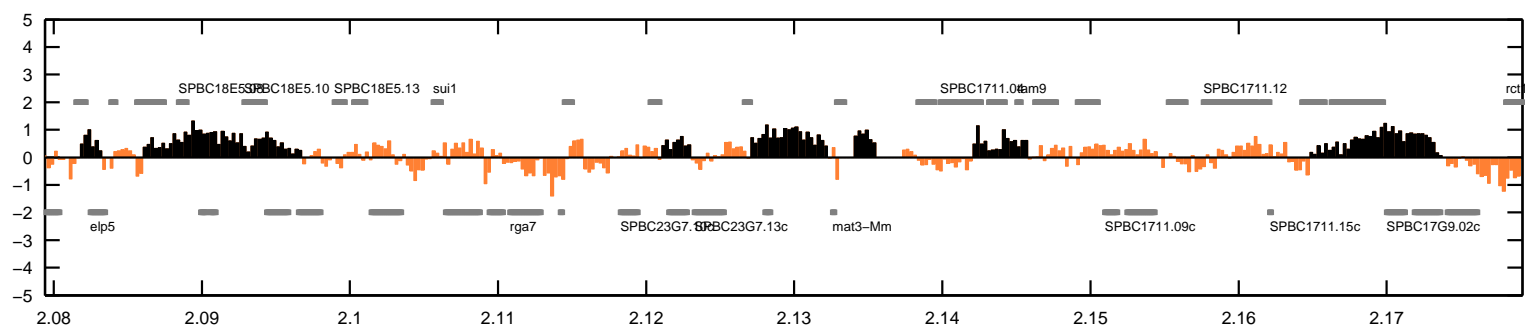

$\times 10^6$

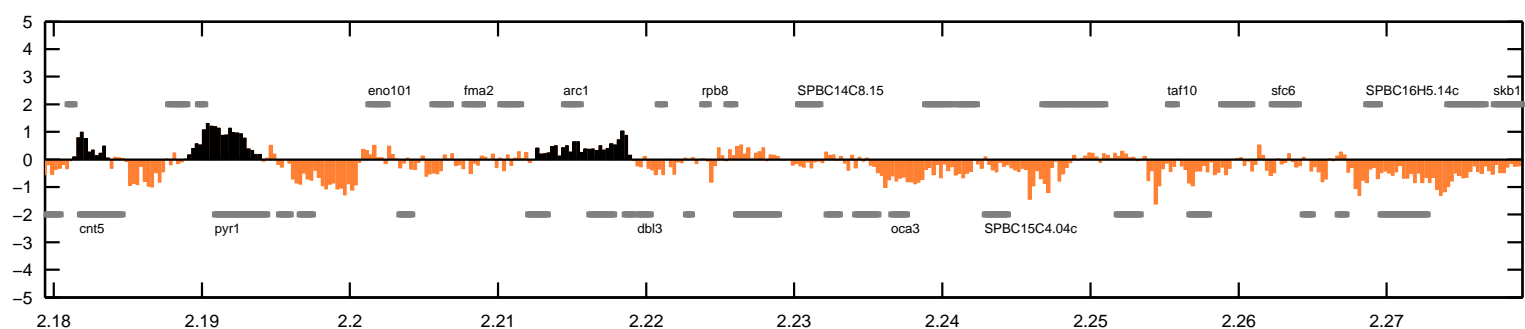

$\times 10^6$

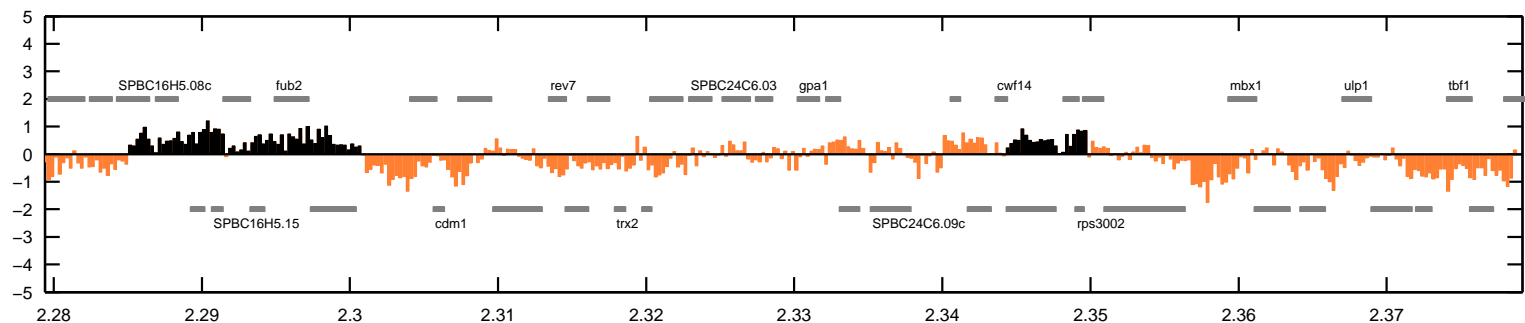

$\times 10^6$

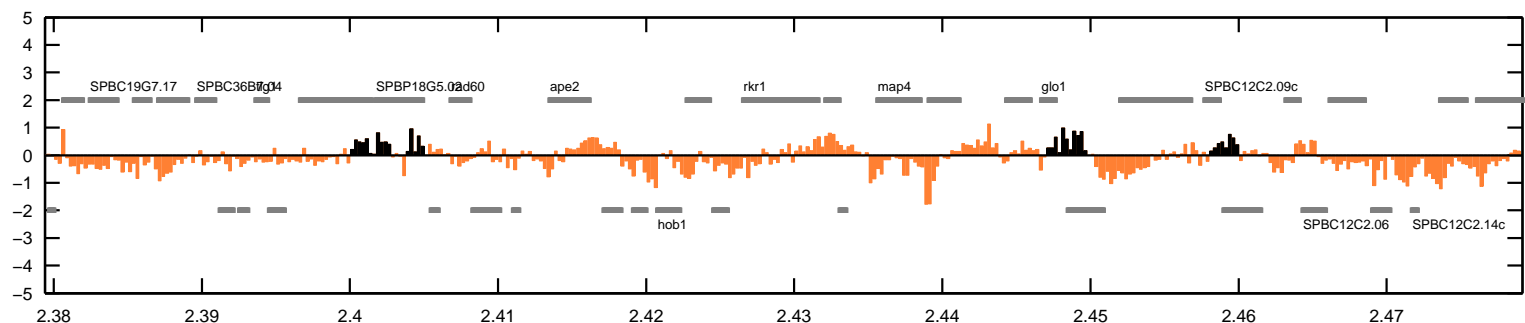

$\times 10^6$

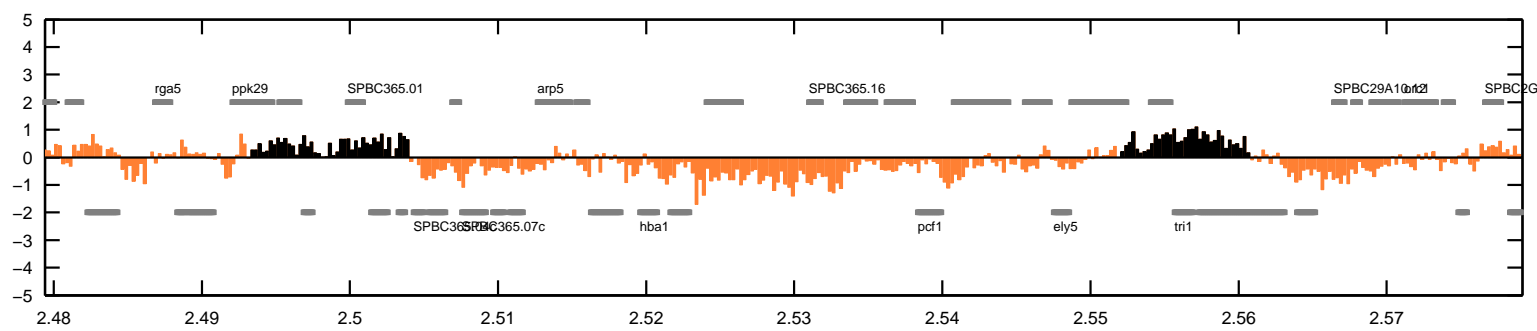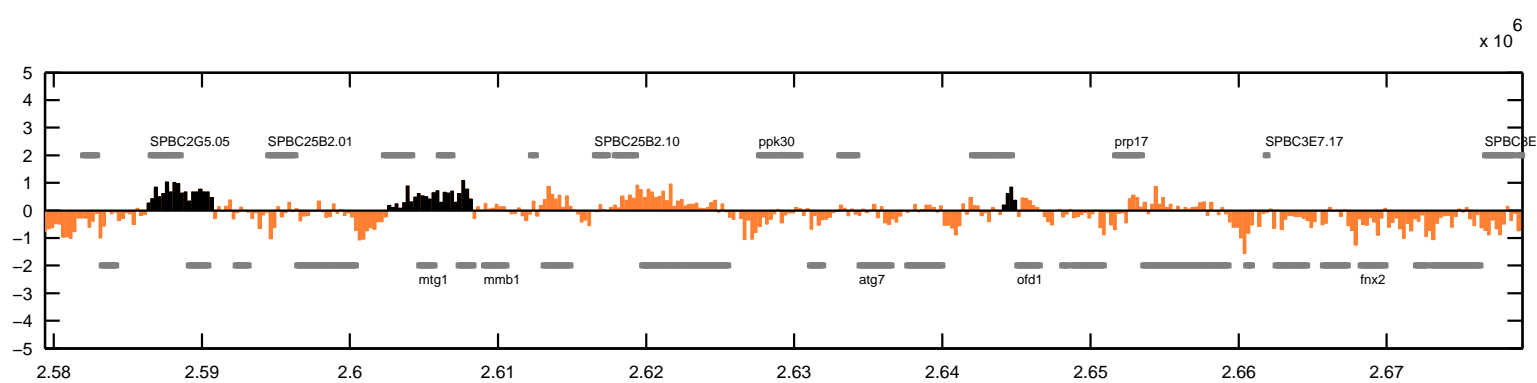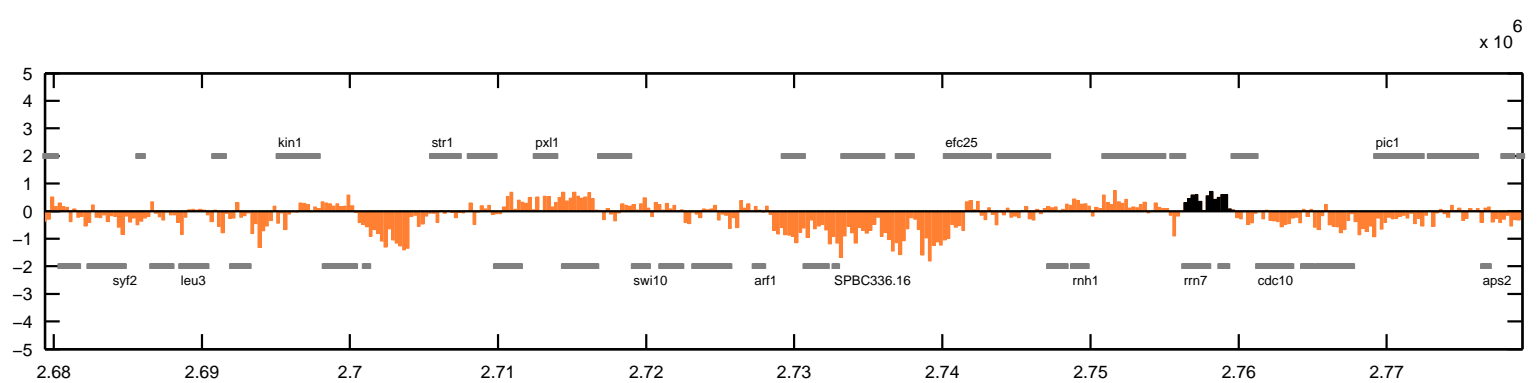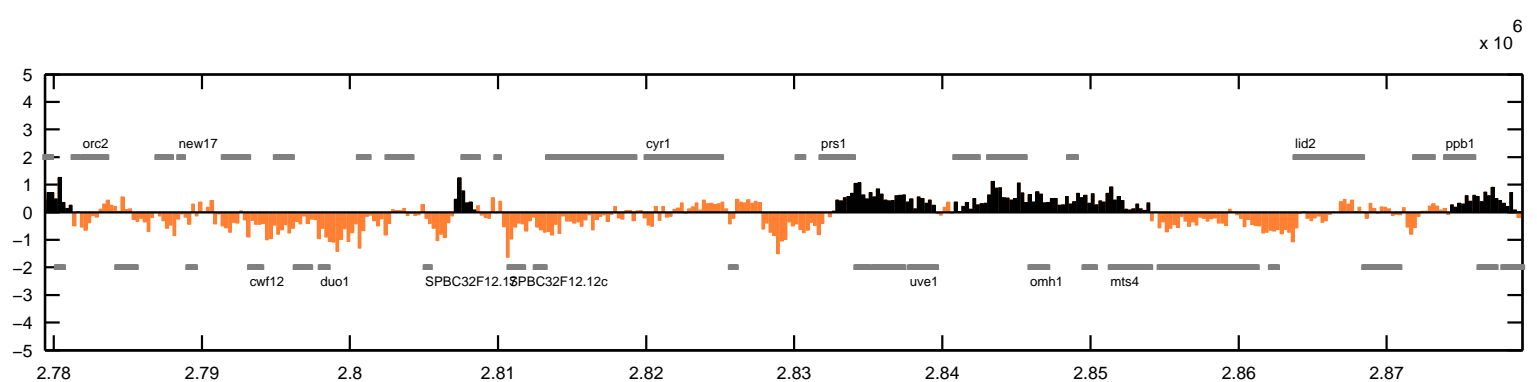

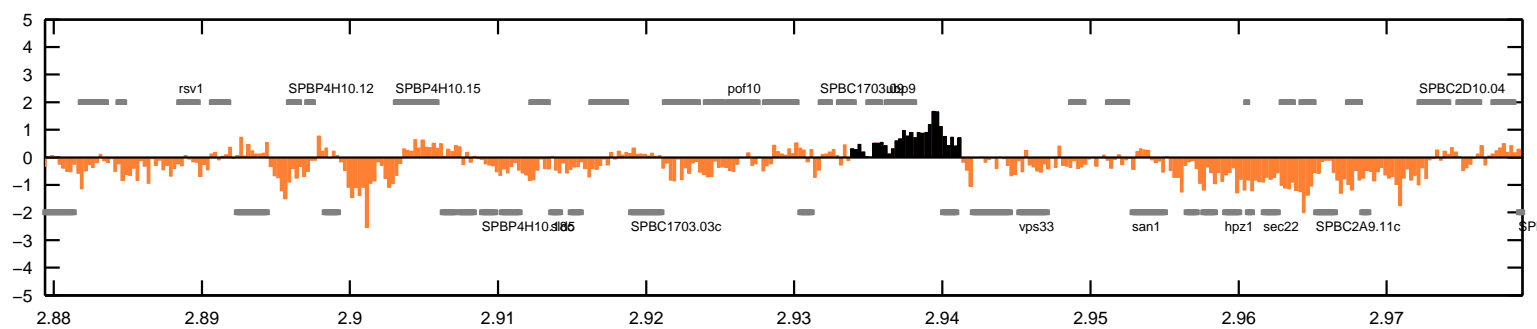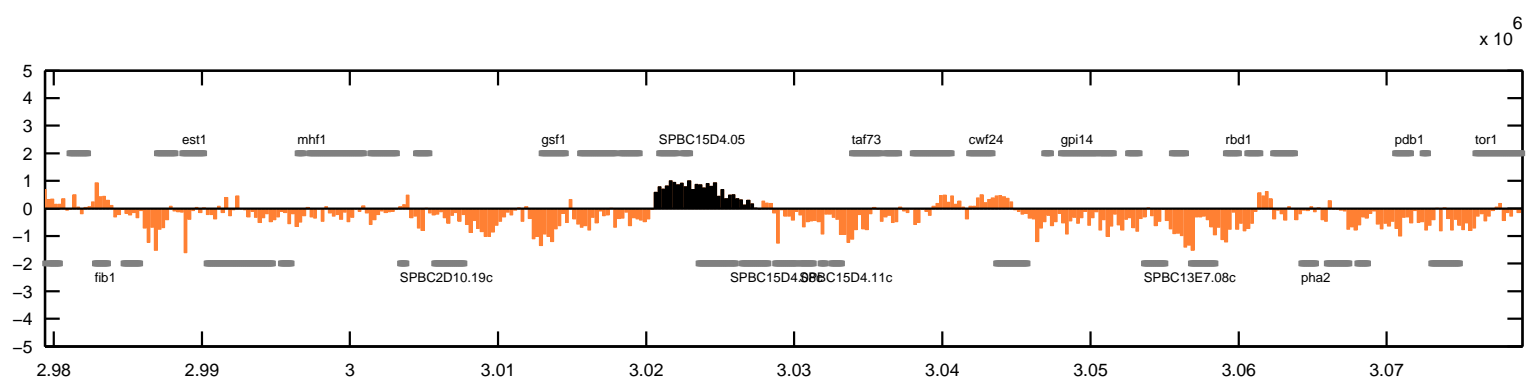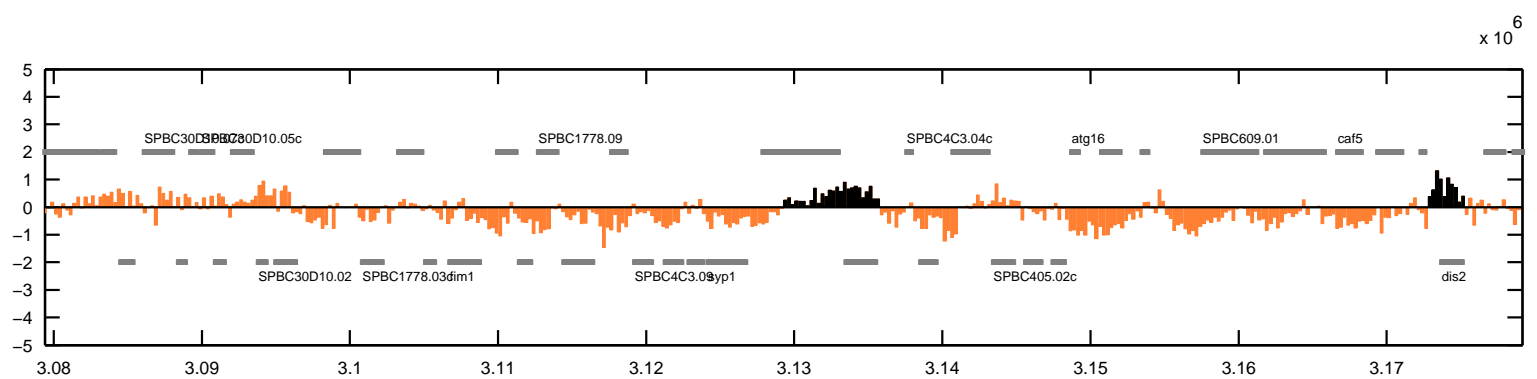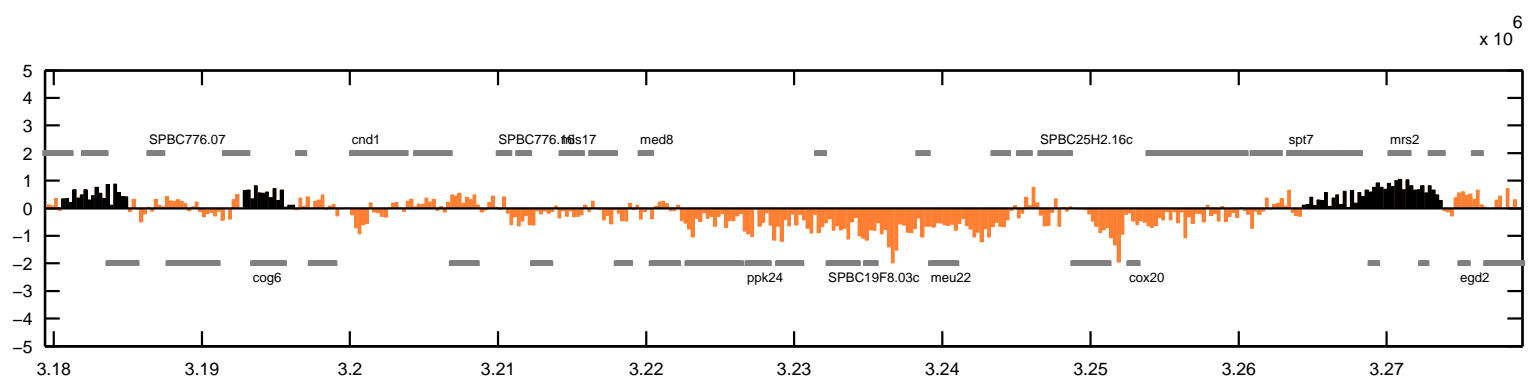

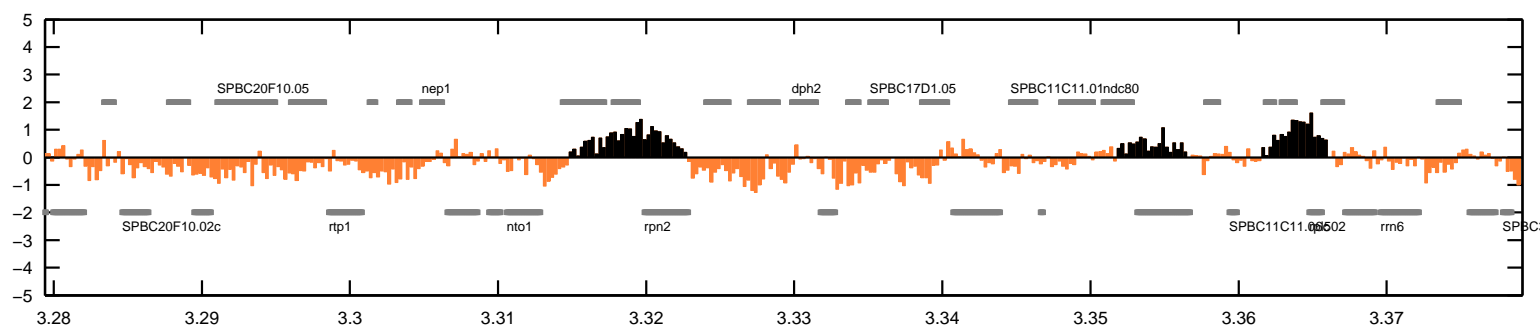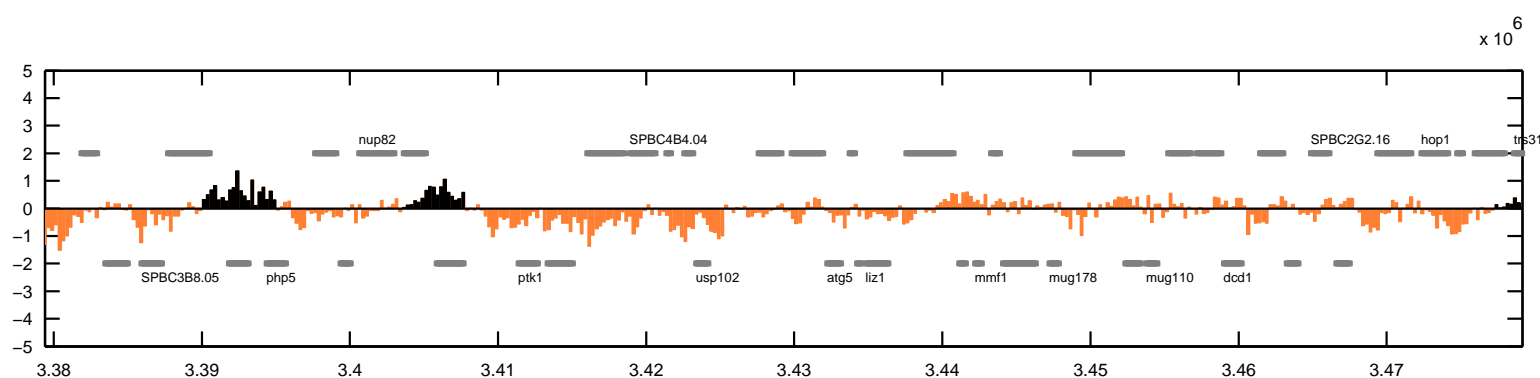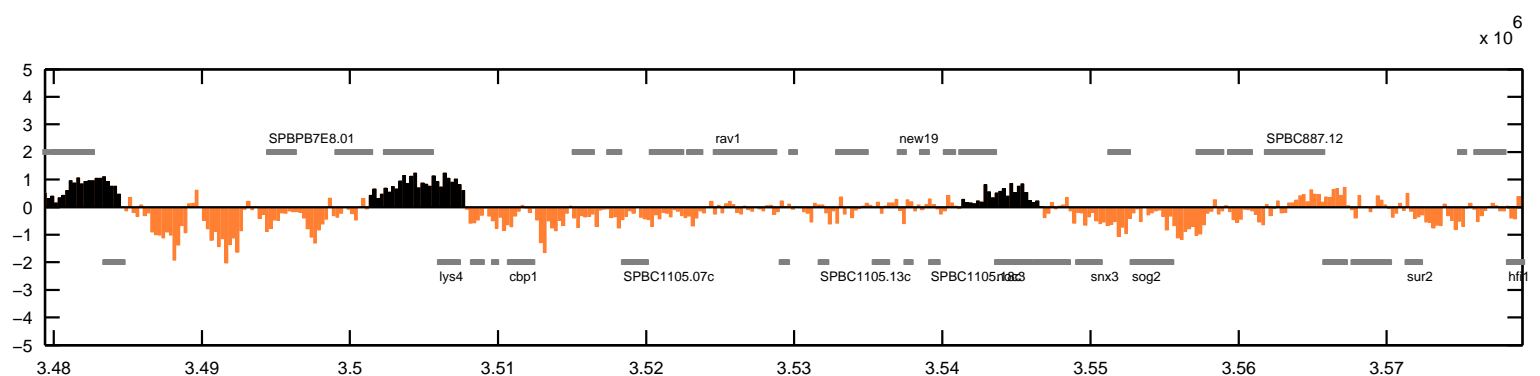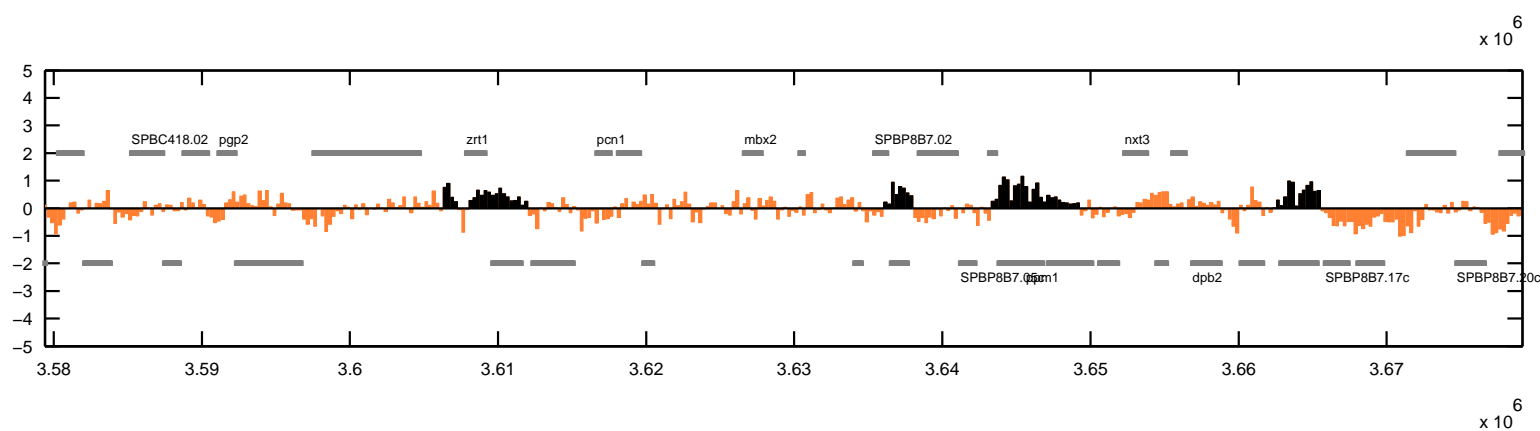

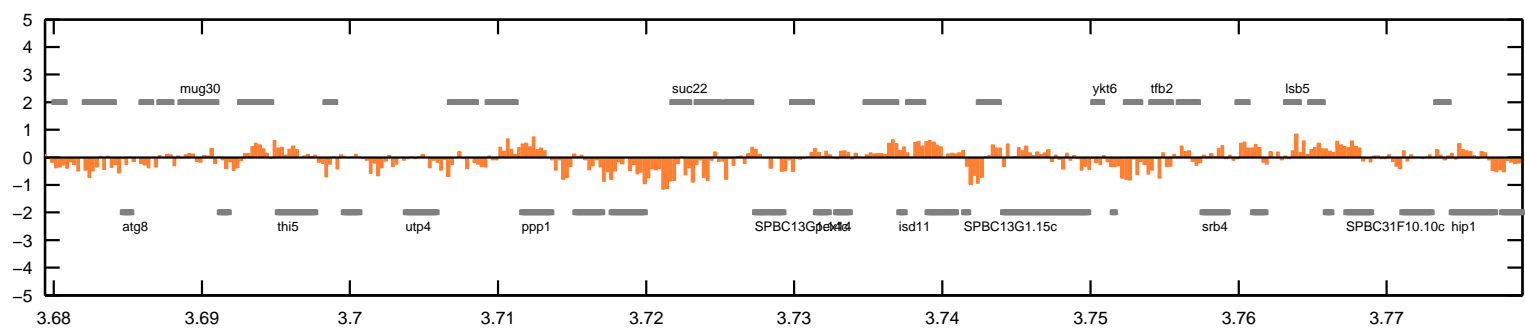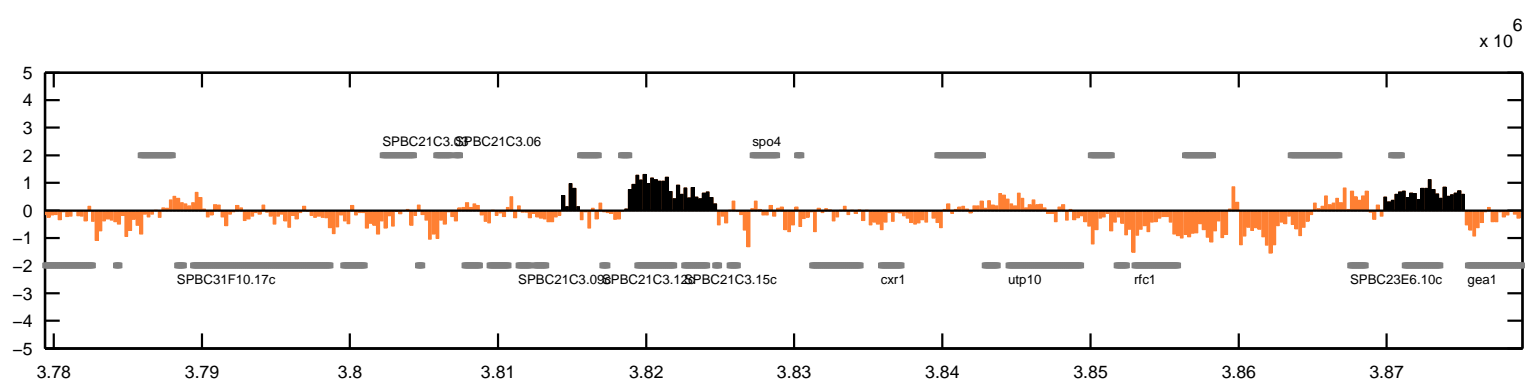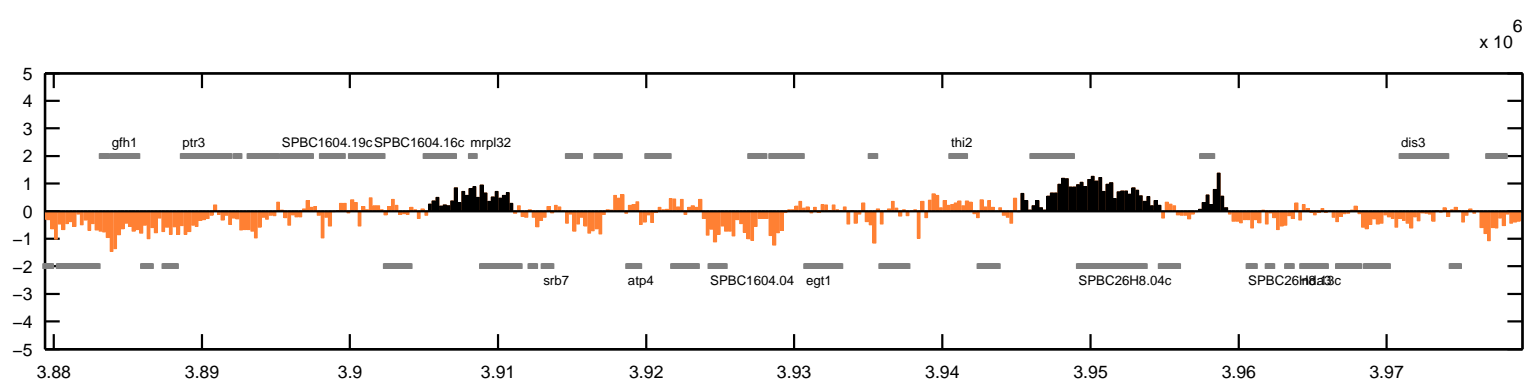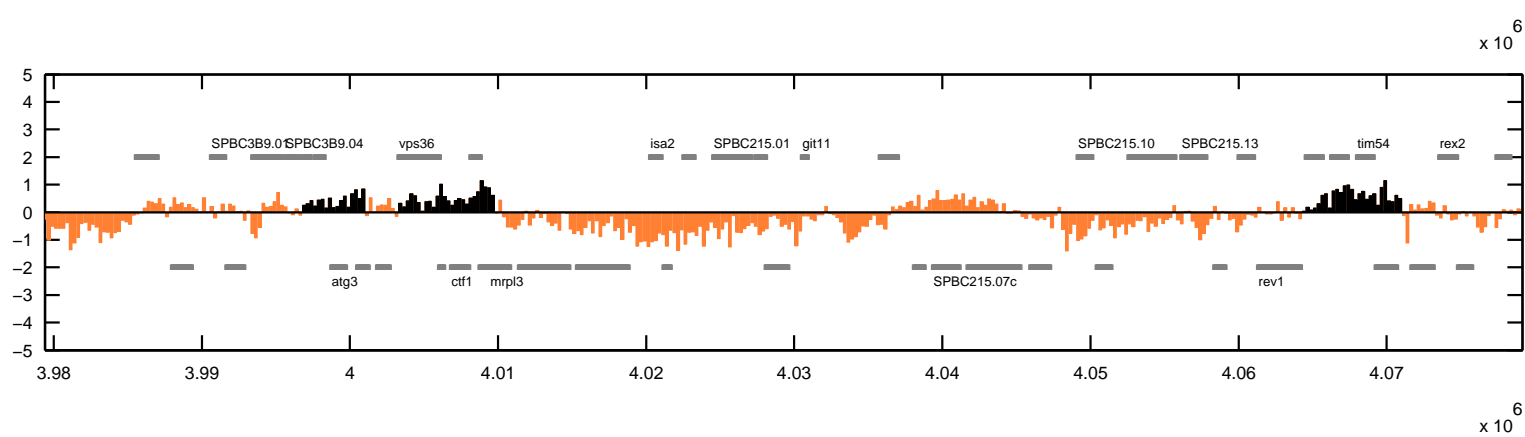

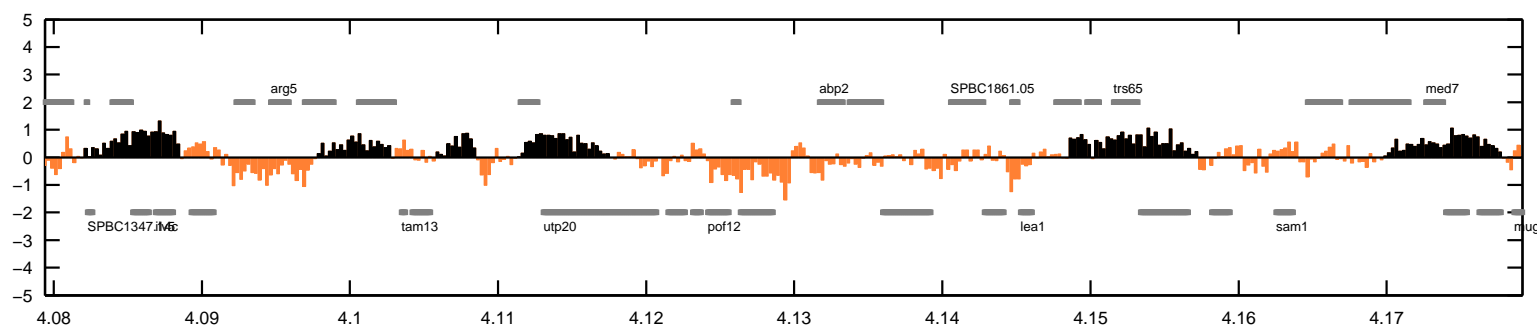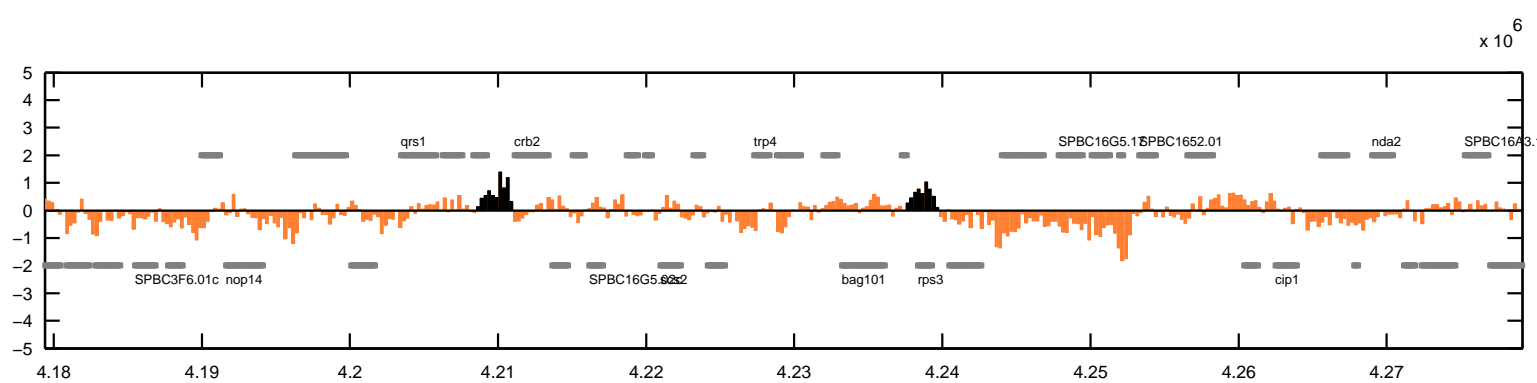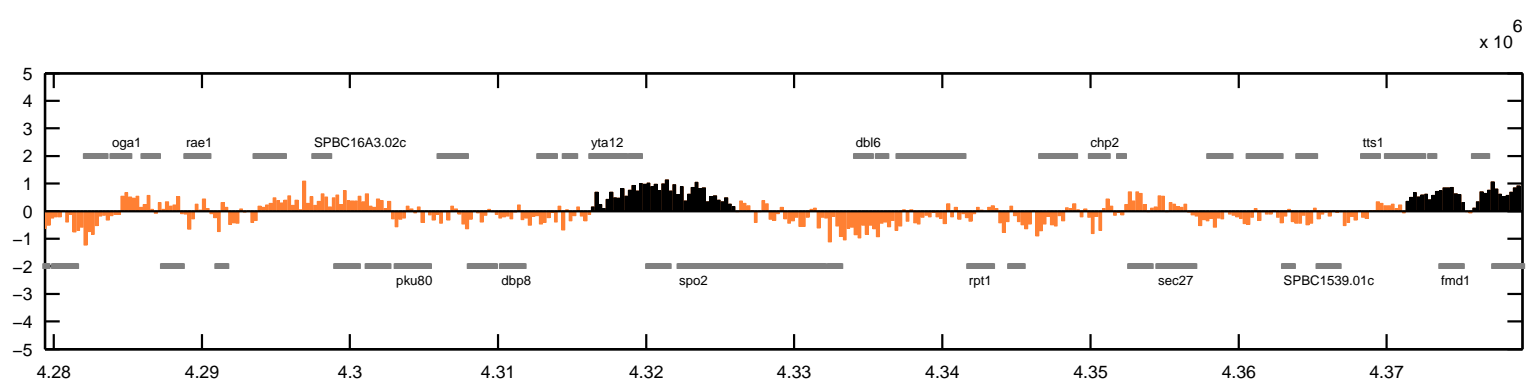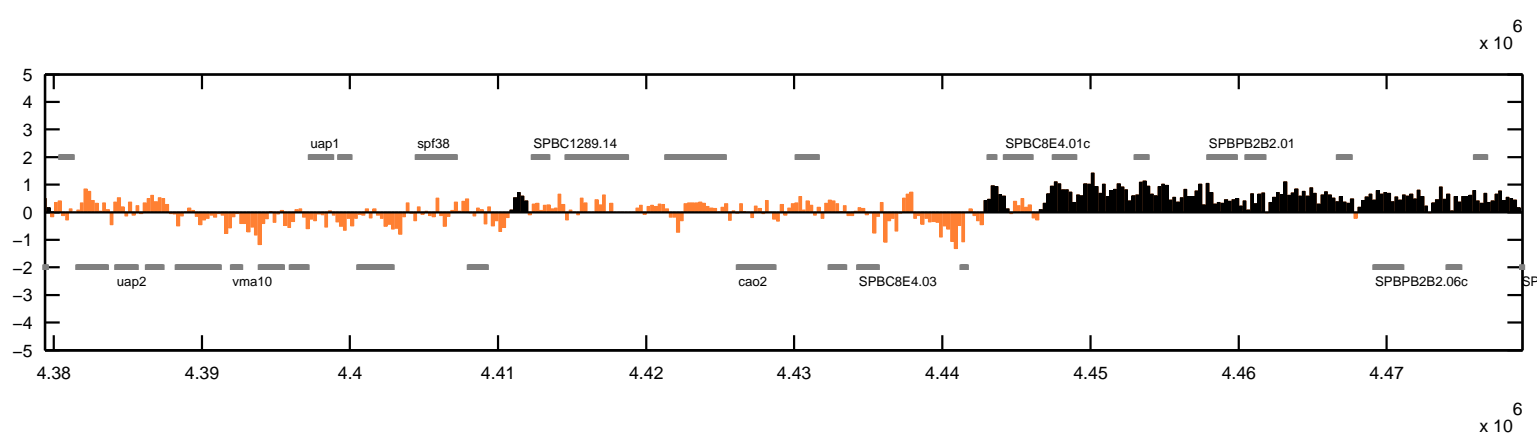

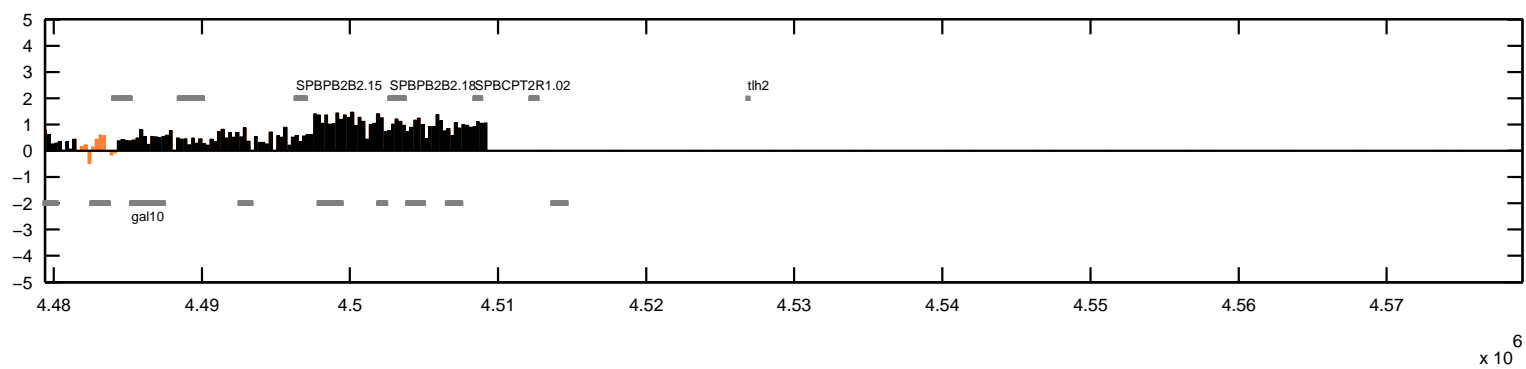

Supplement: SUPPLEMENTARY DATA [file supp_gkw252_nar-00155-v-2016-File007.pdf]

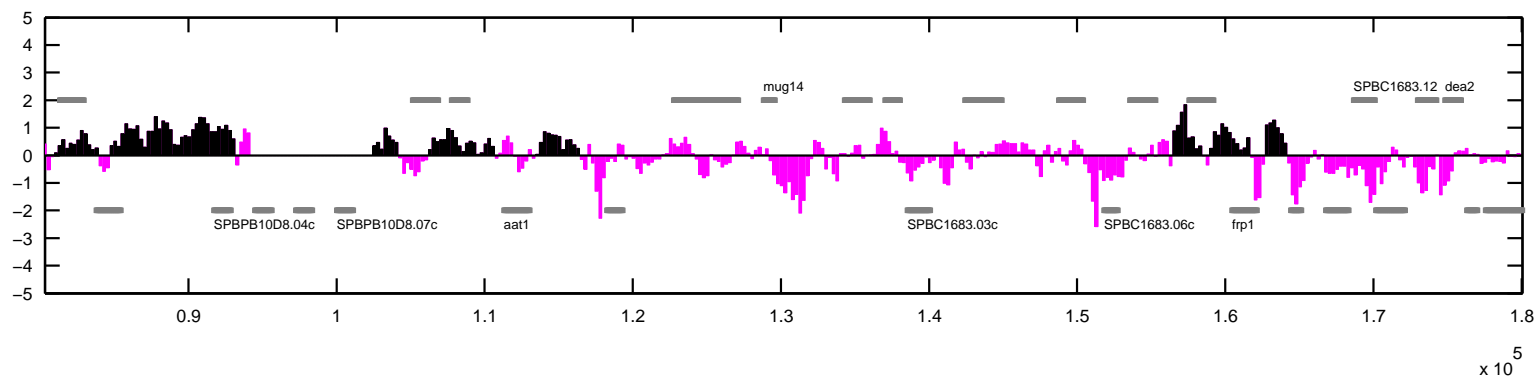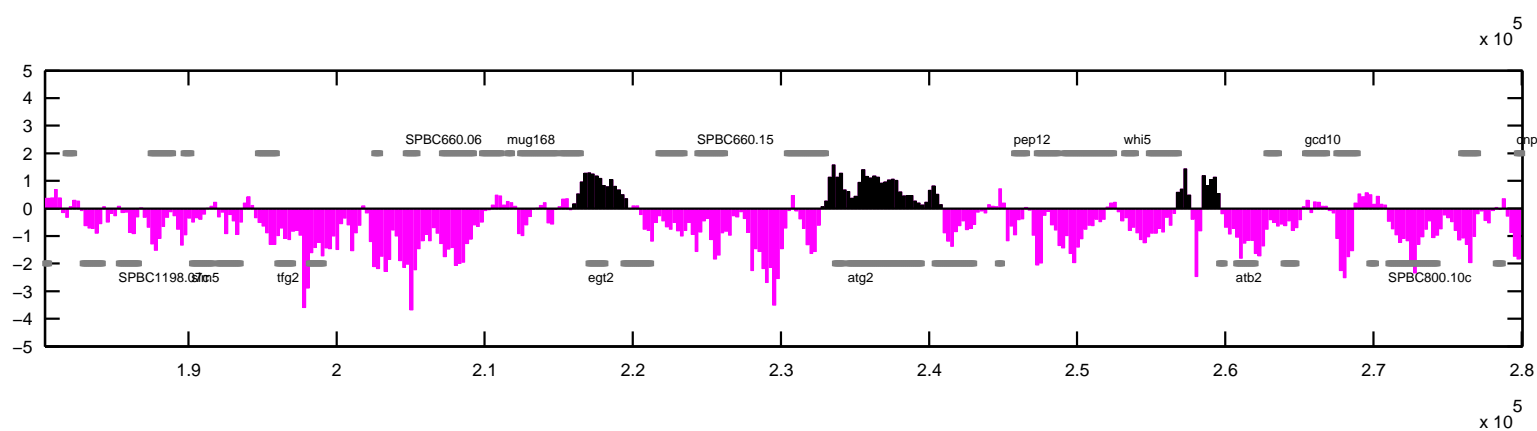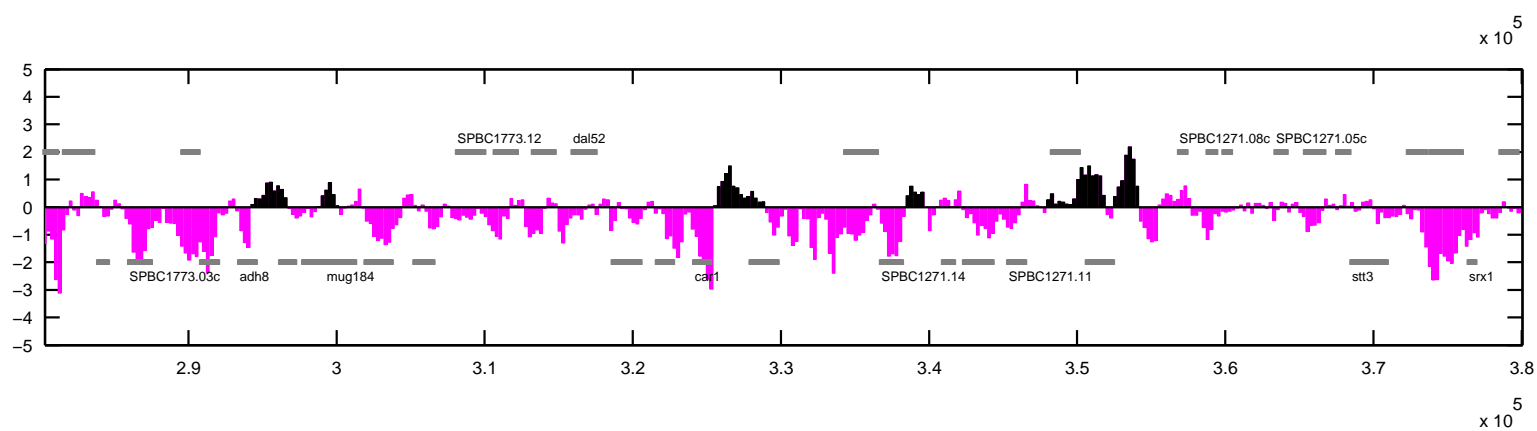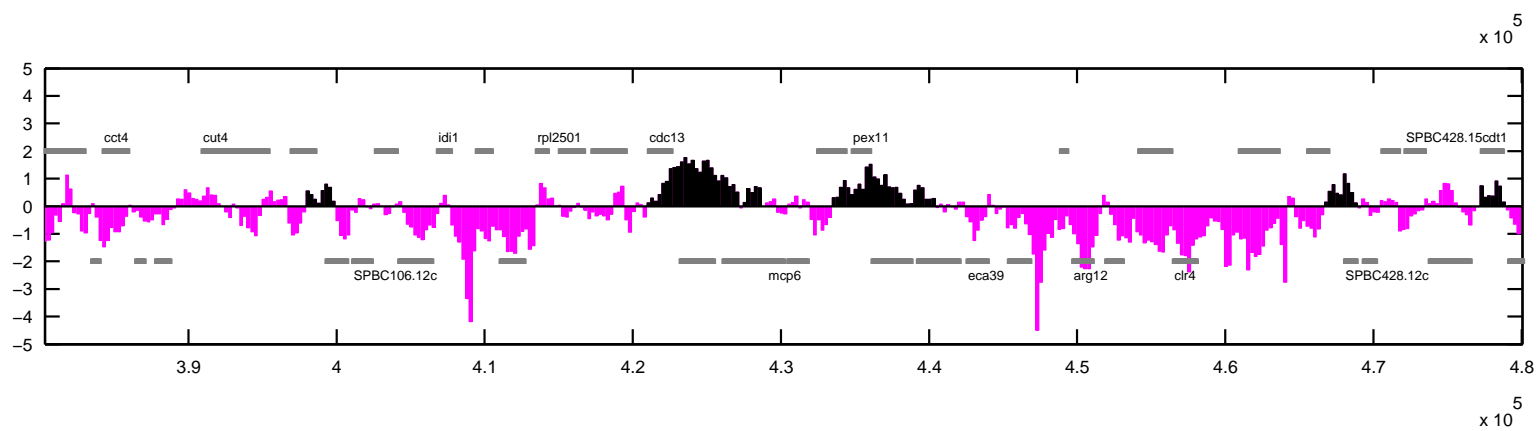

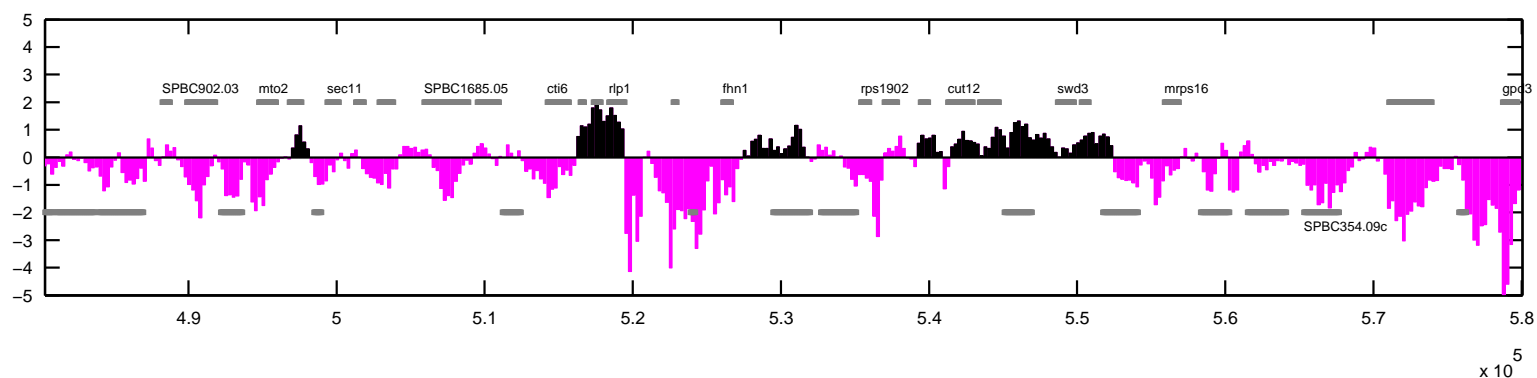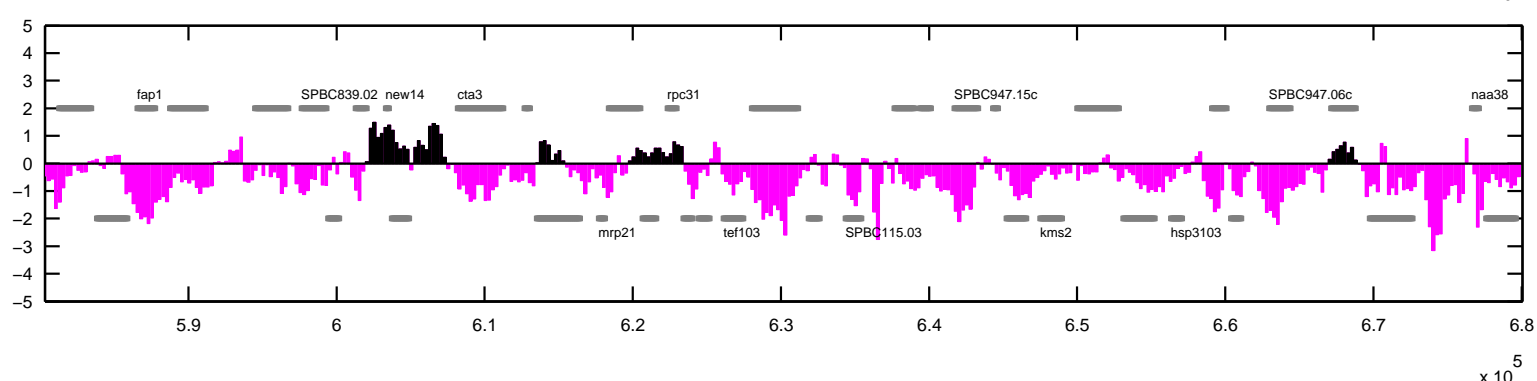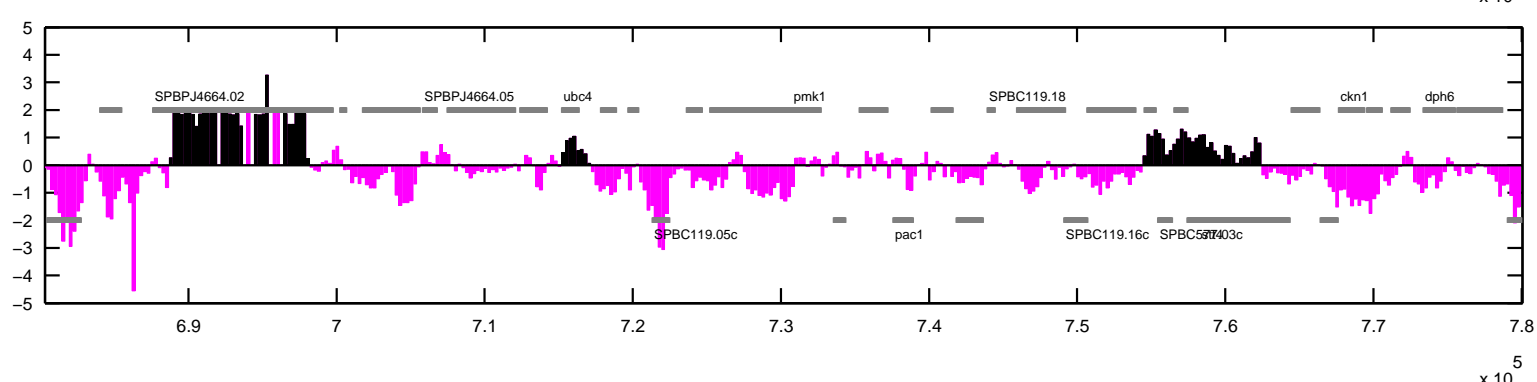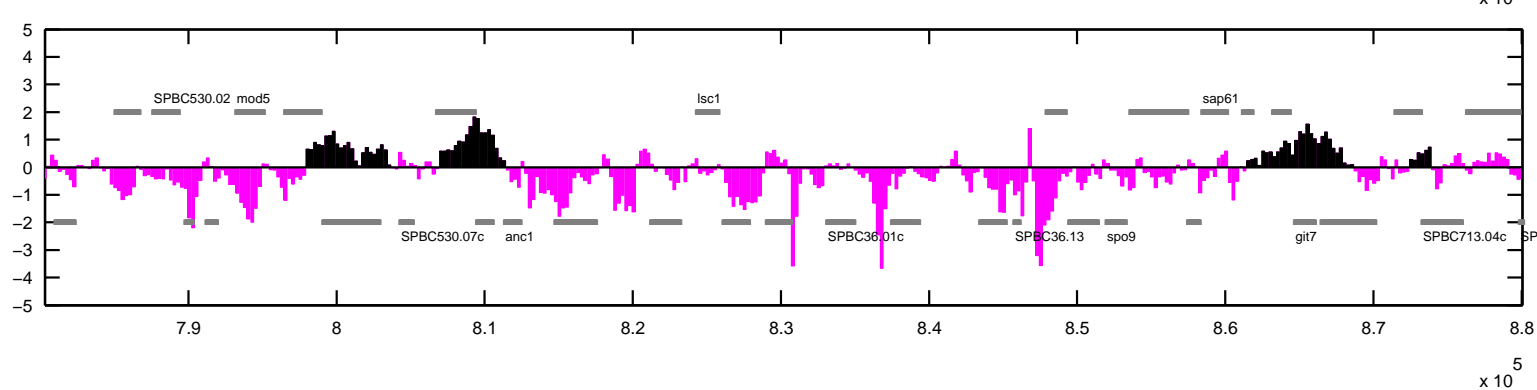

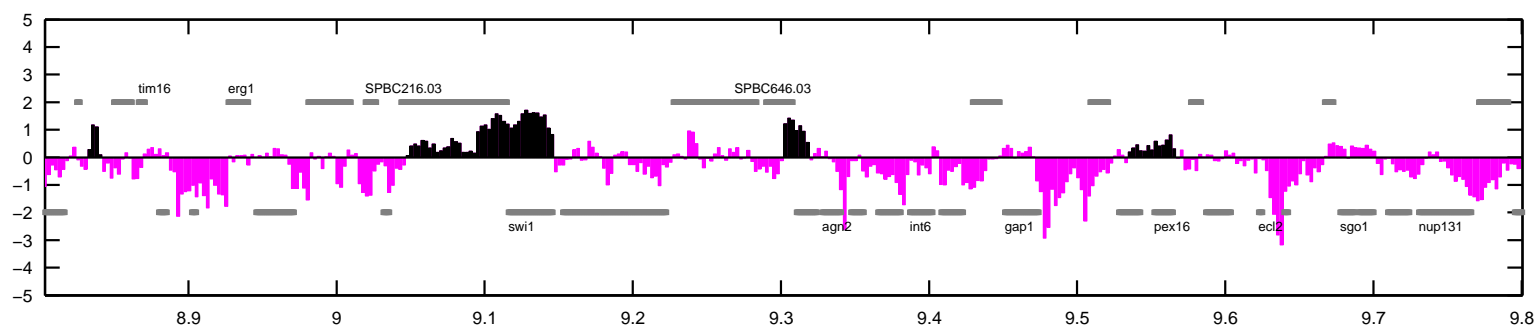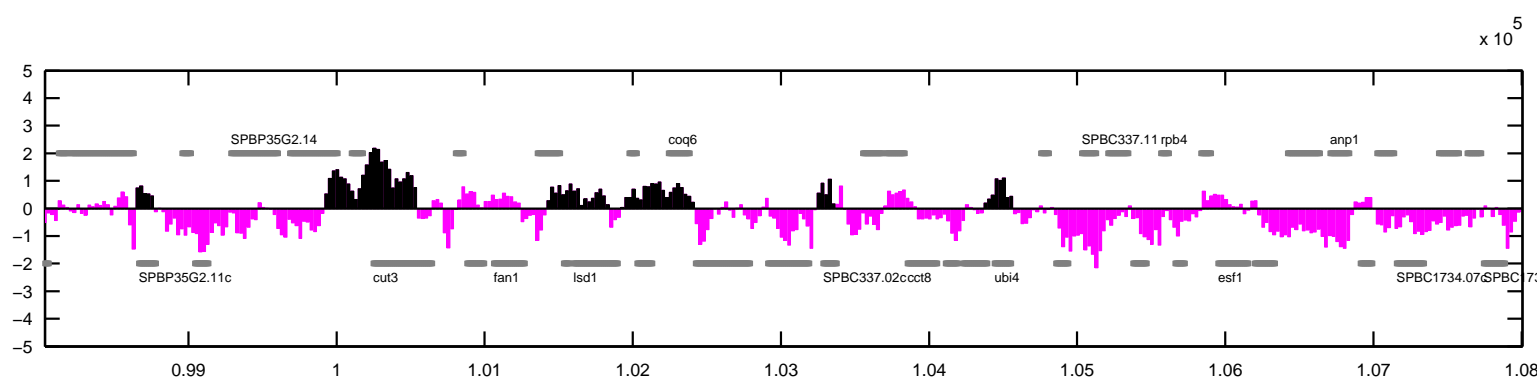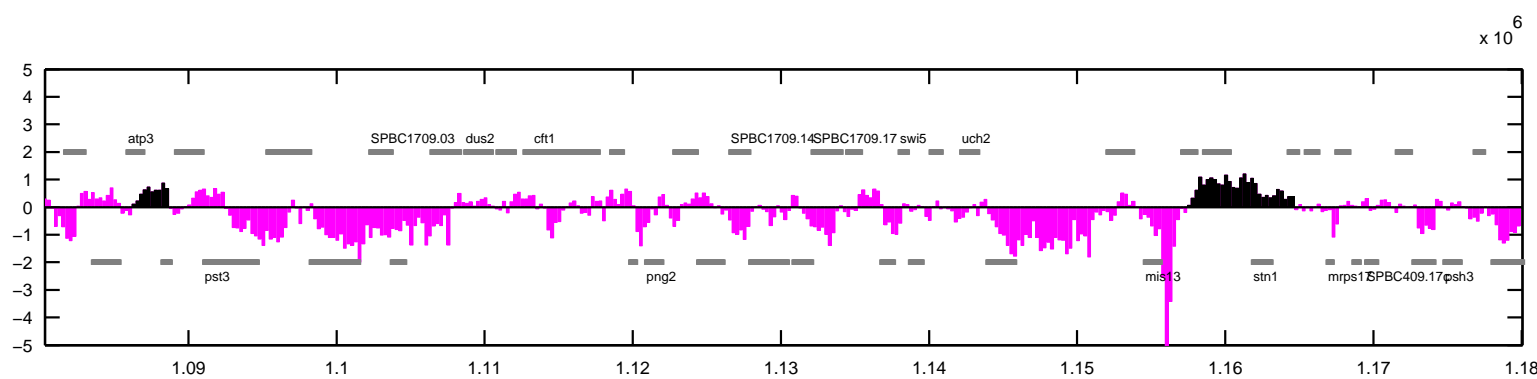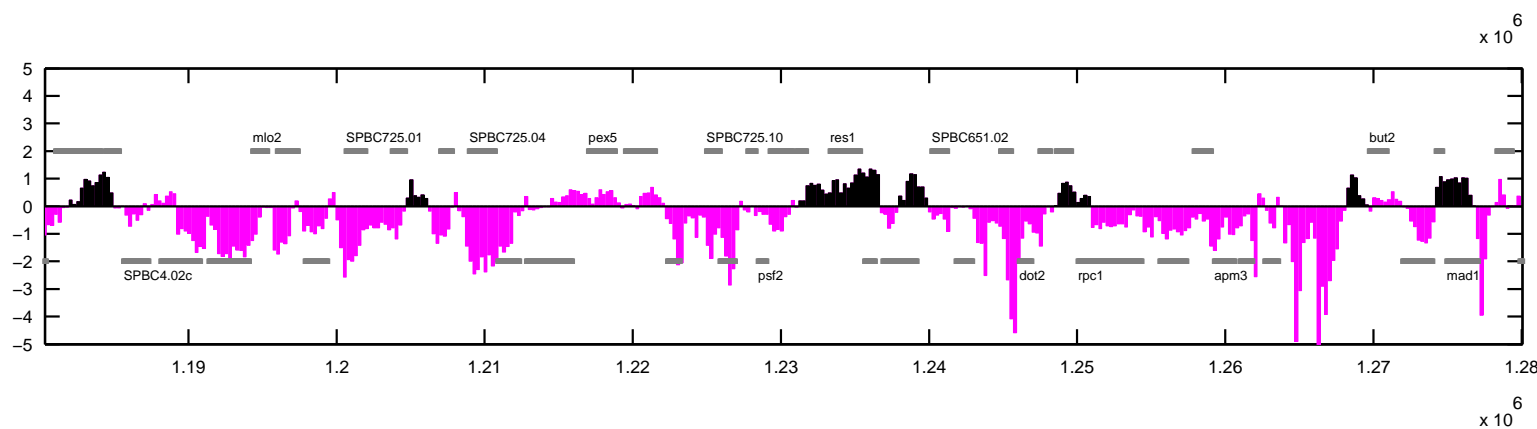

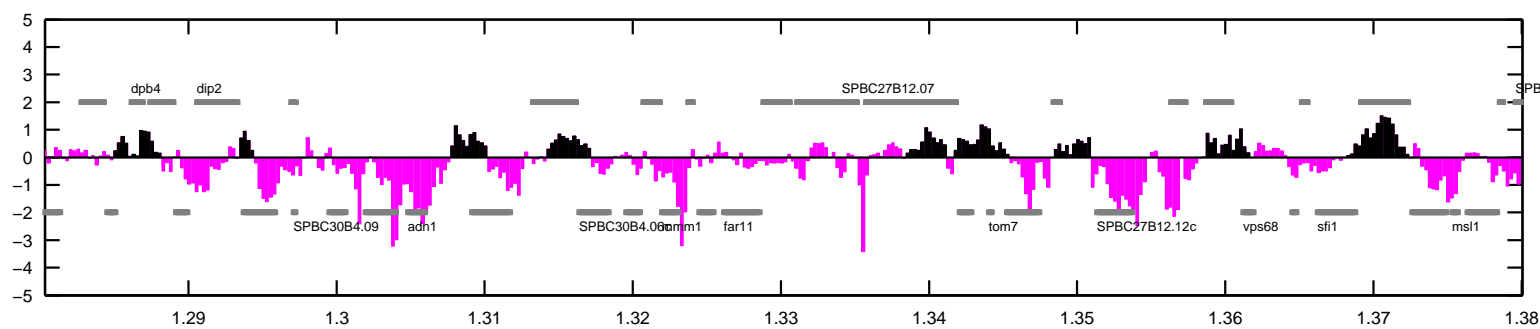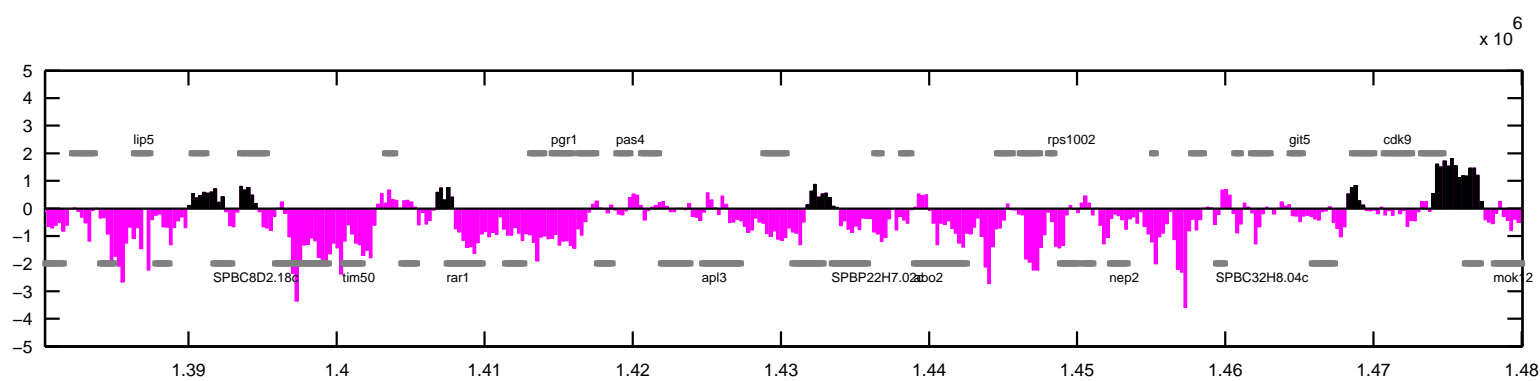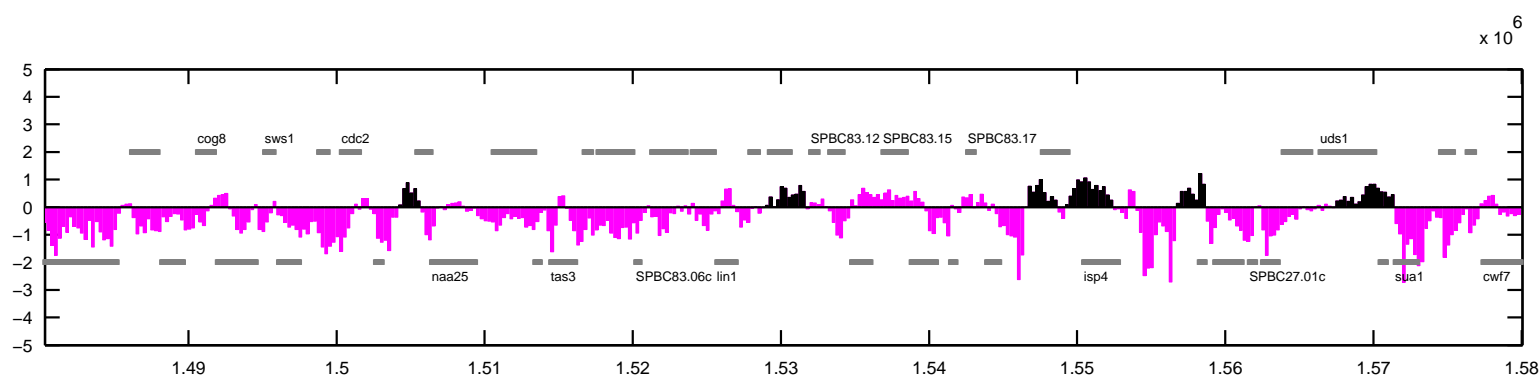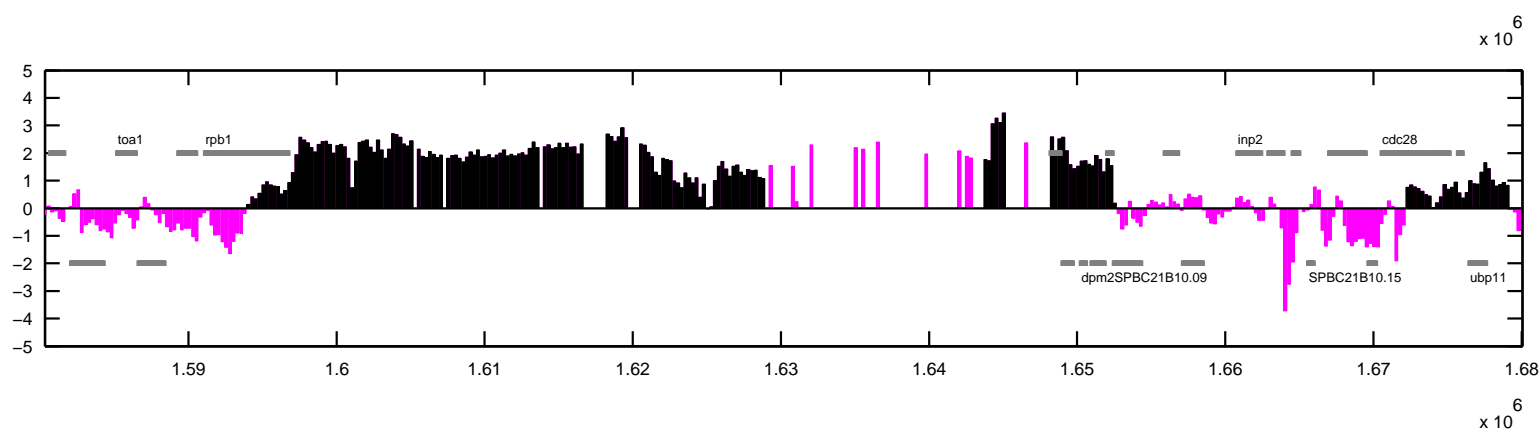

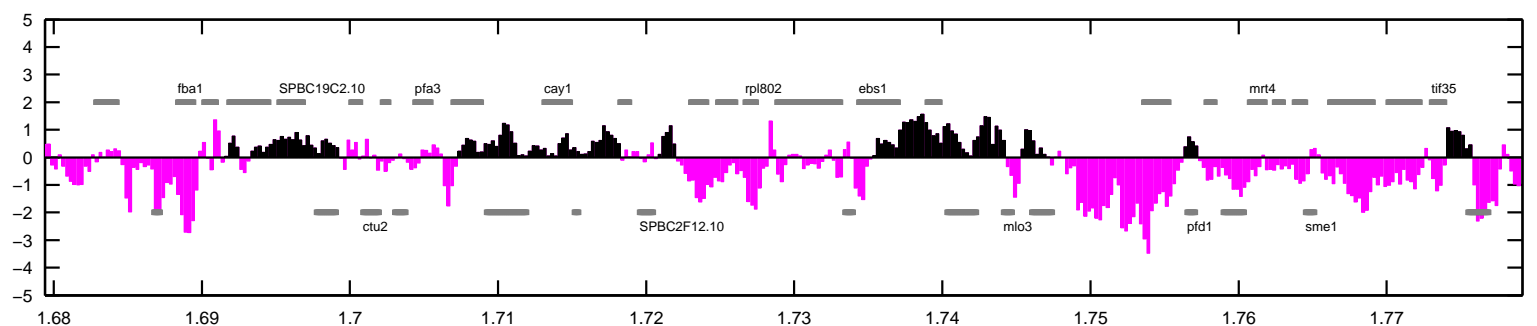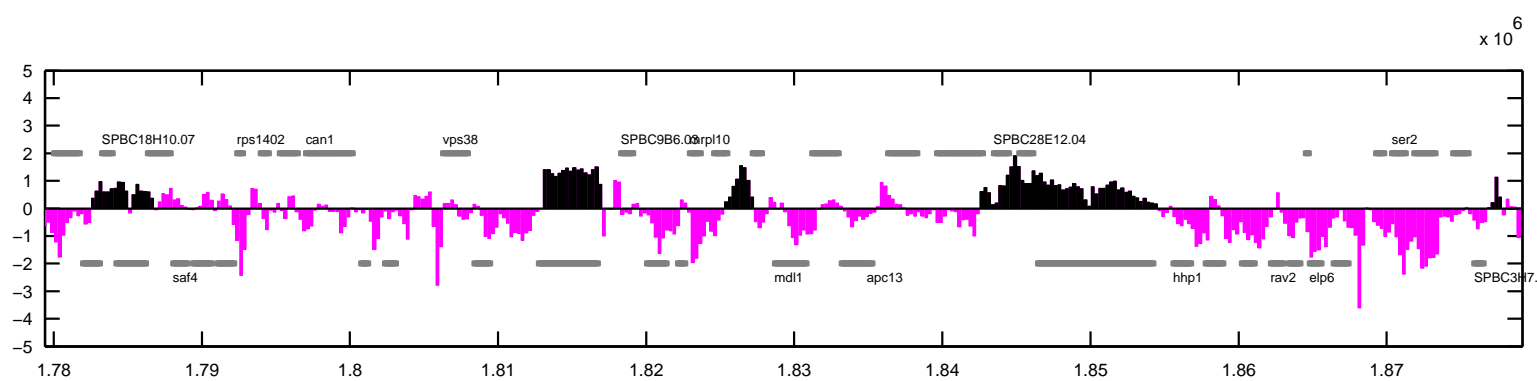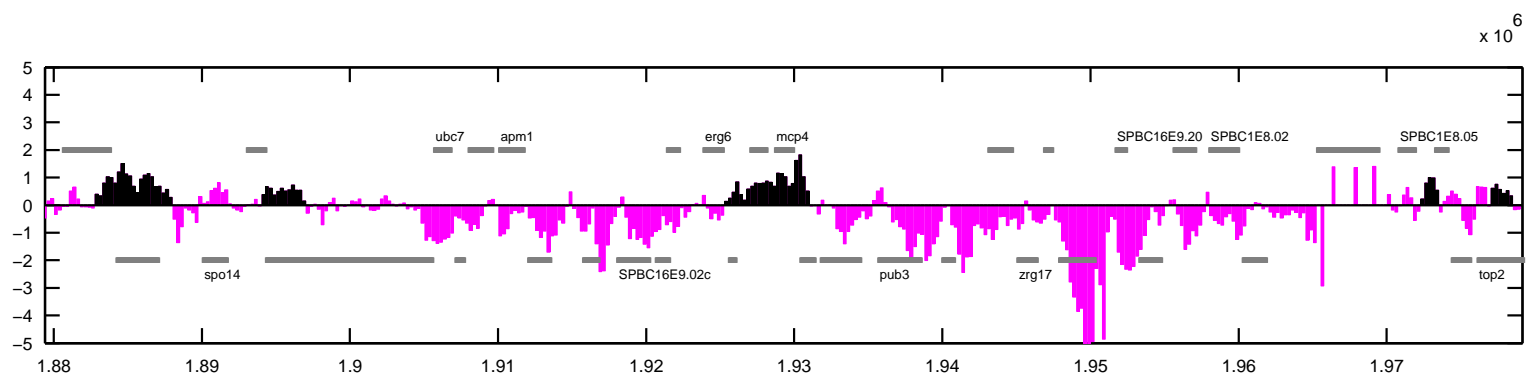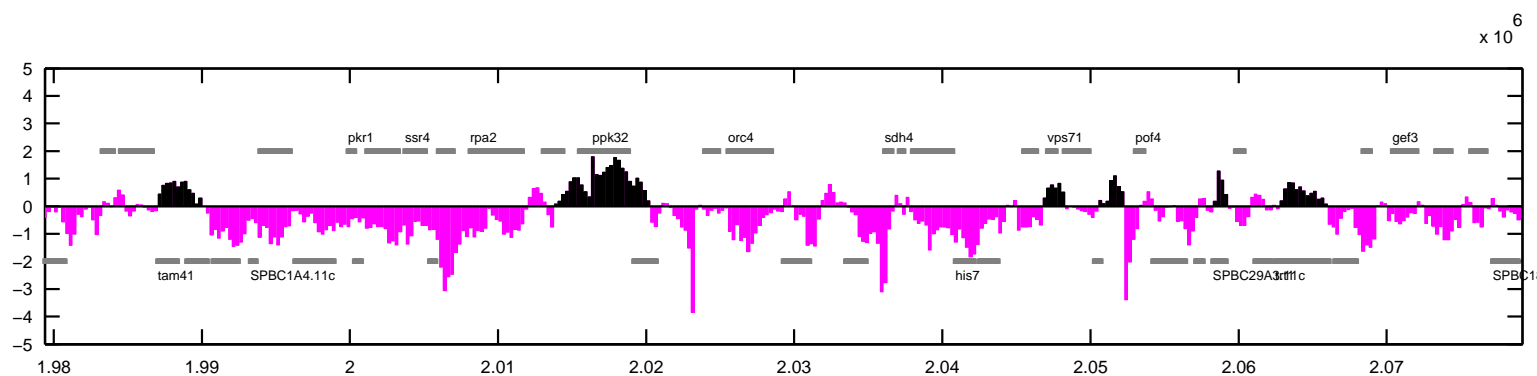

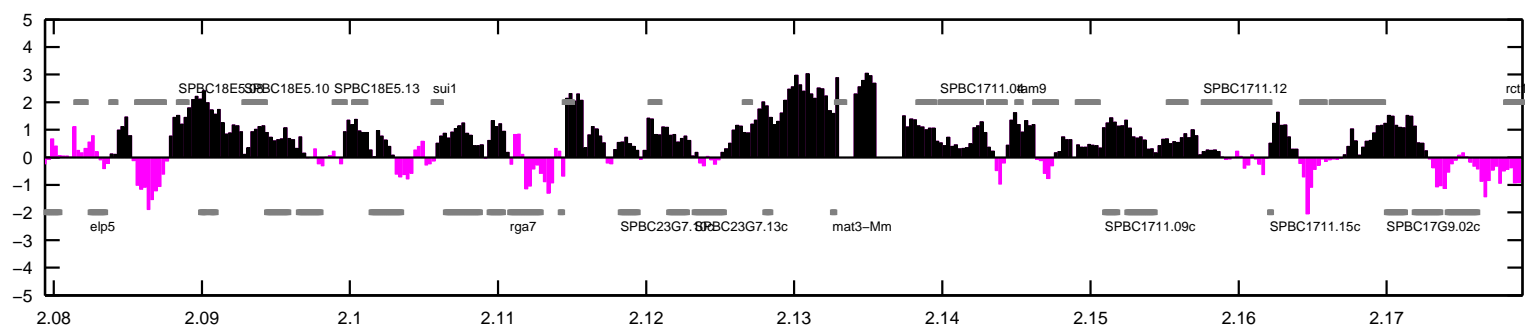

$\times 10^6$

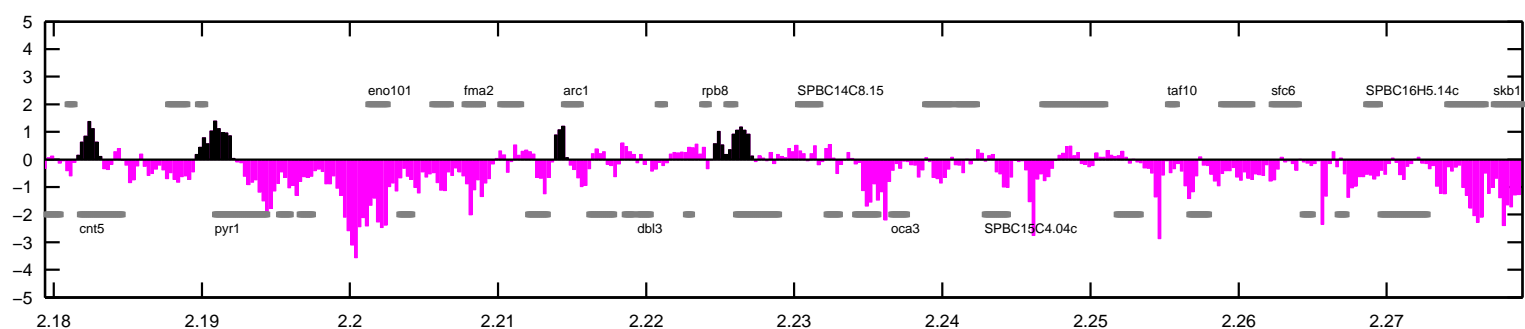

$\times 10^6$

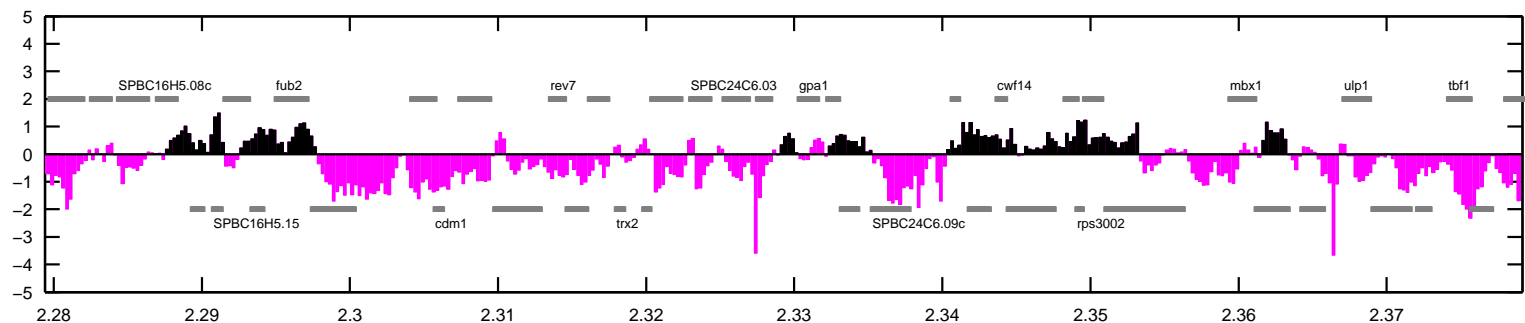

$\times 10^6$

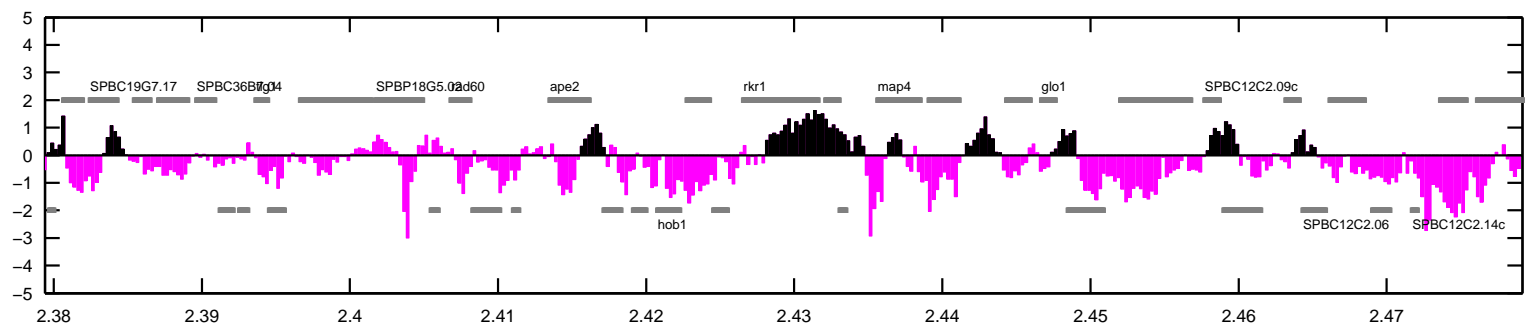

$\times 10^6$

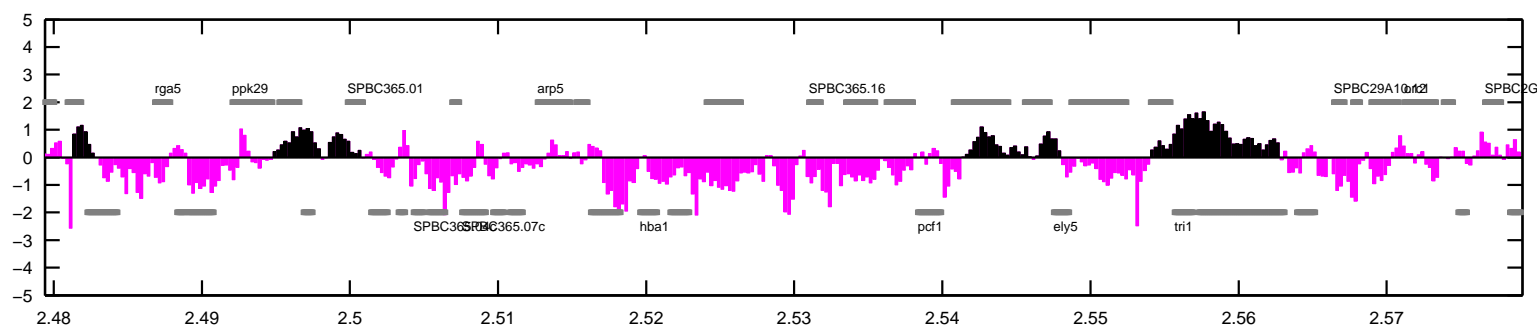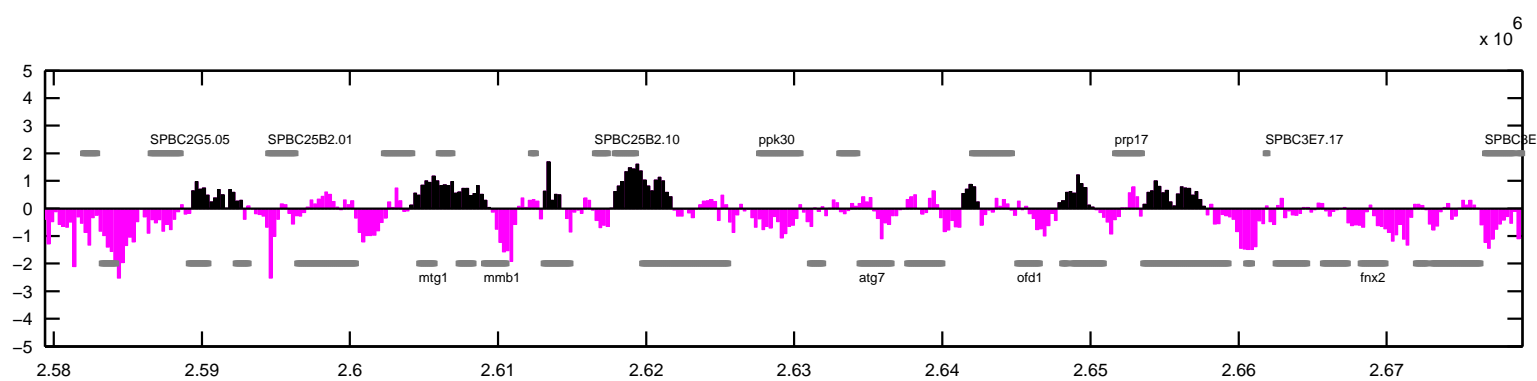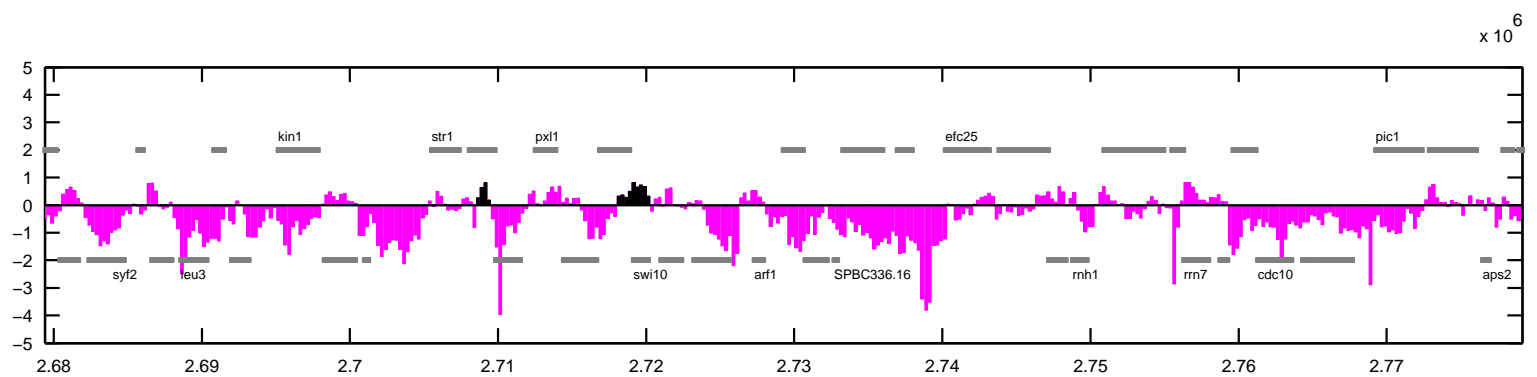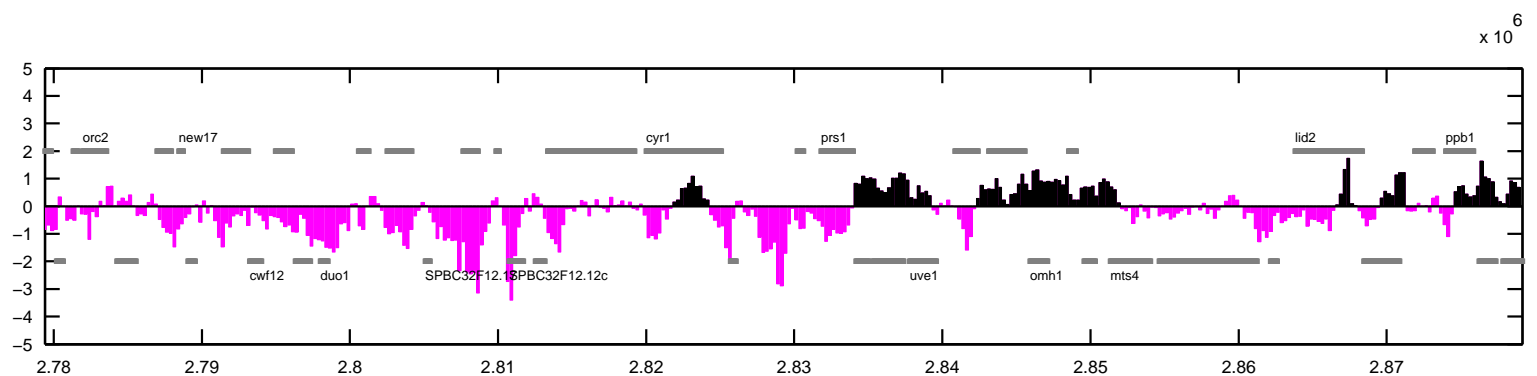

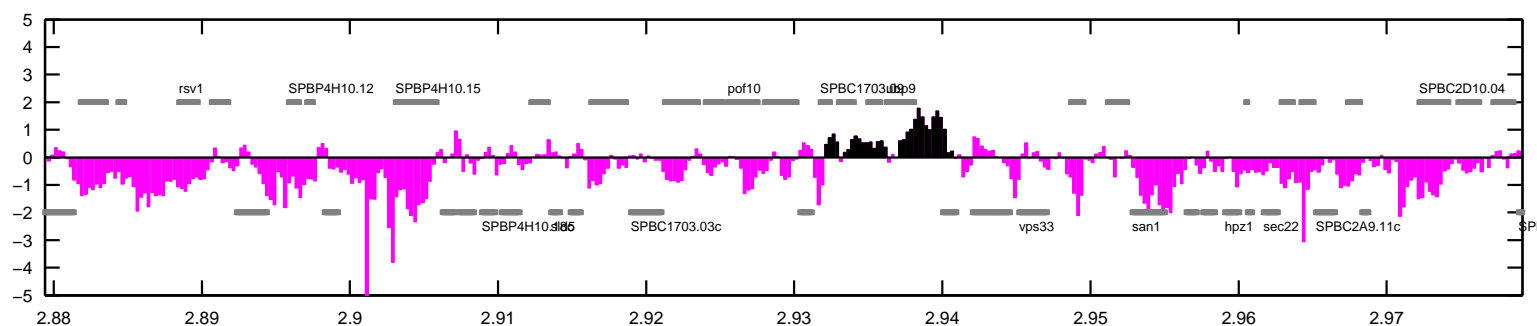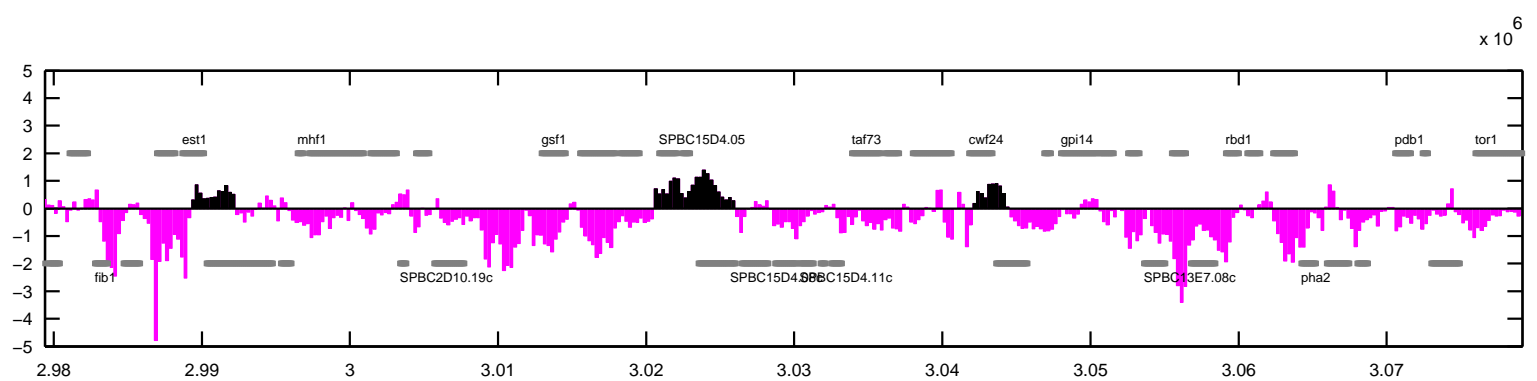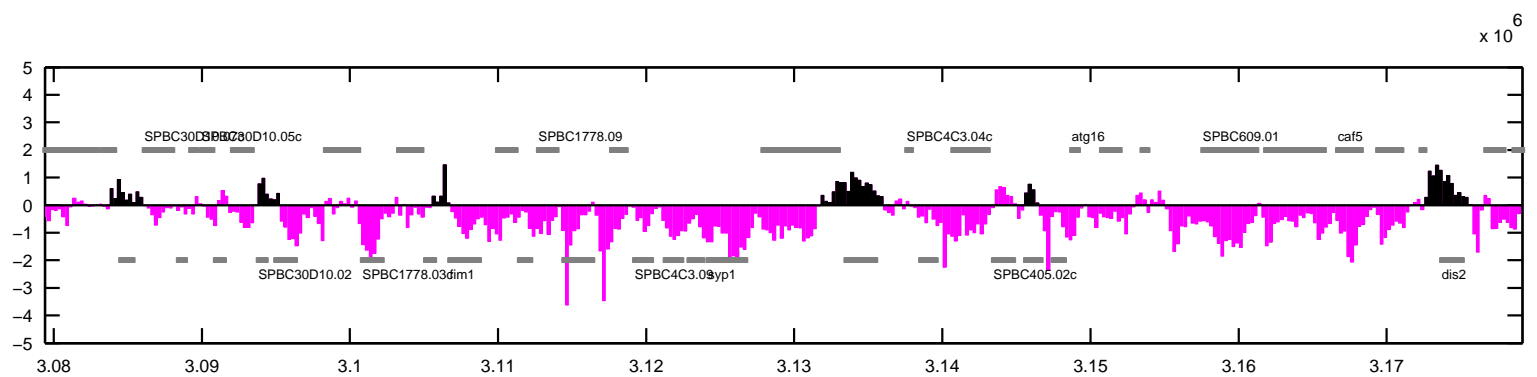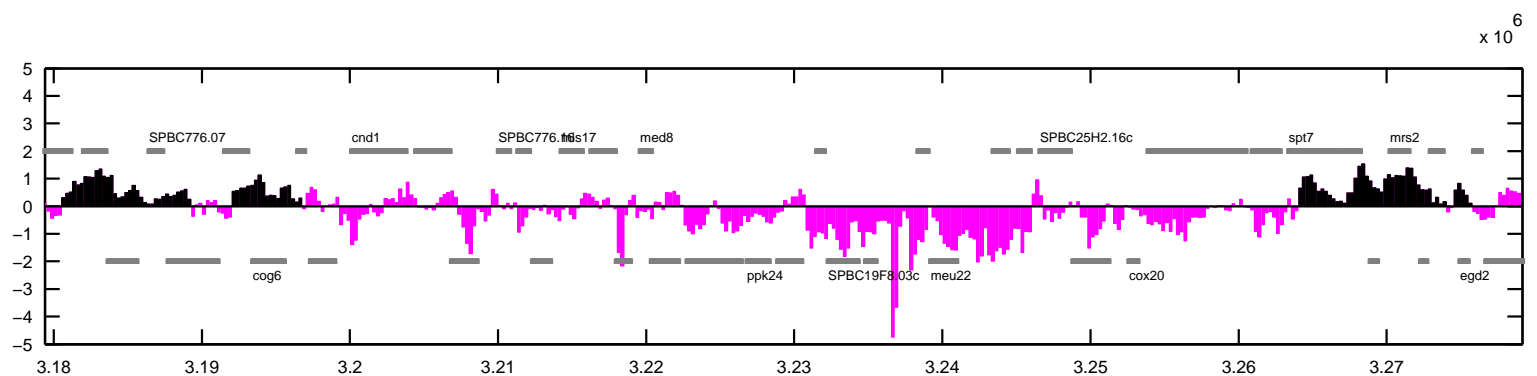

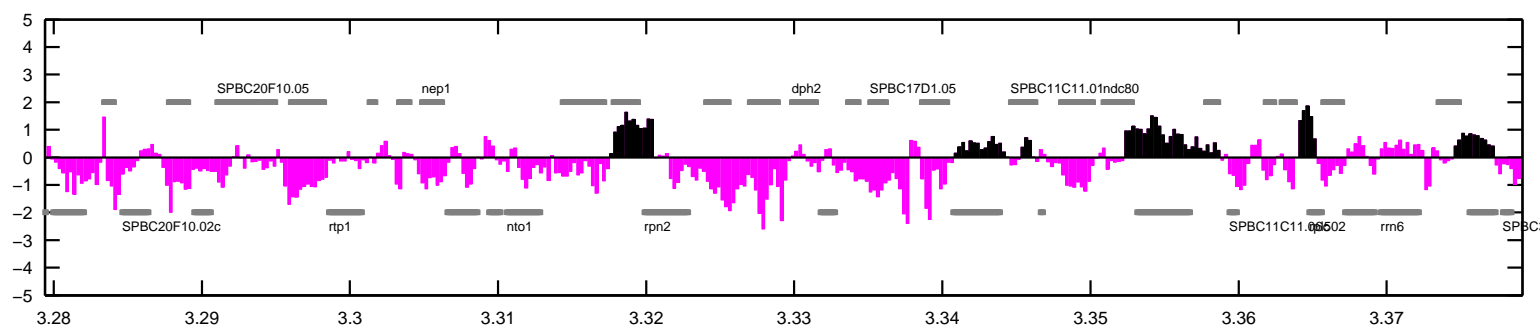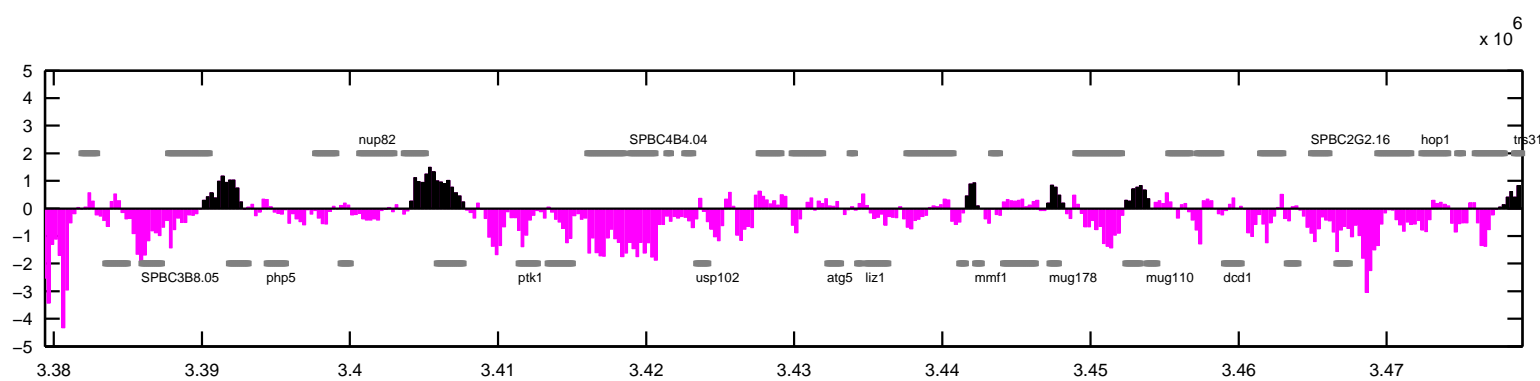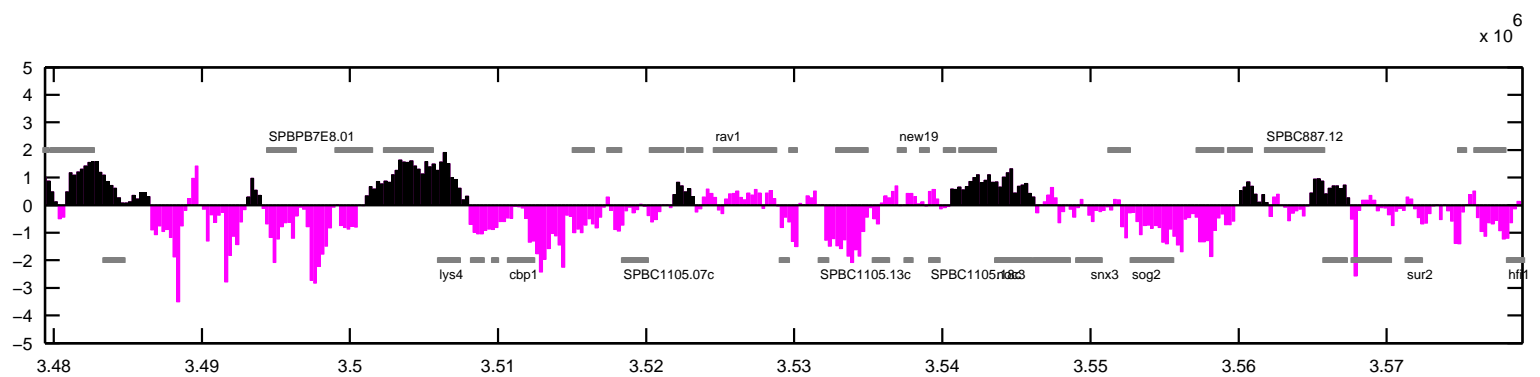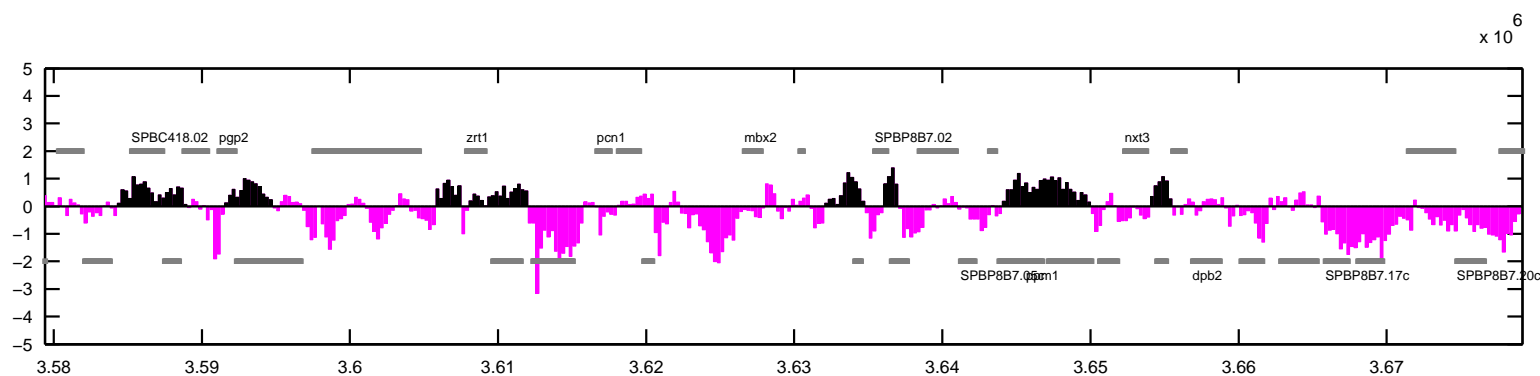

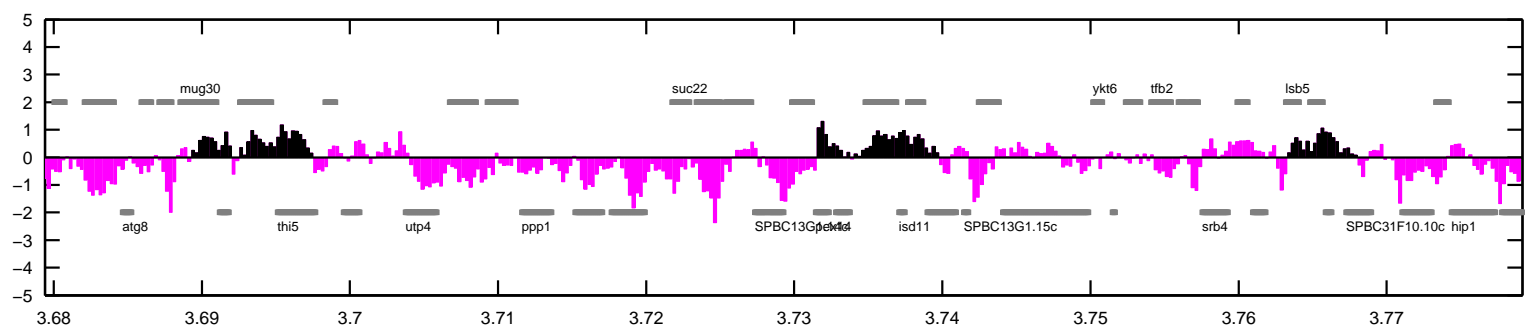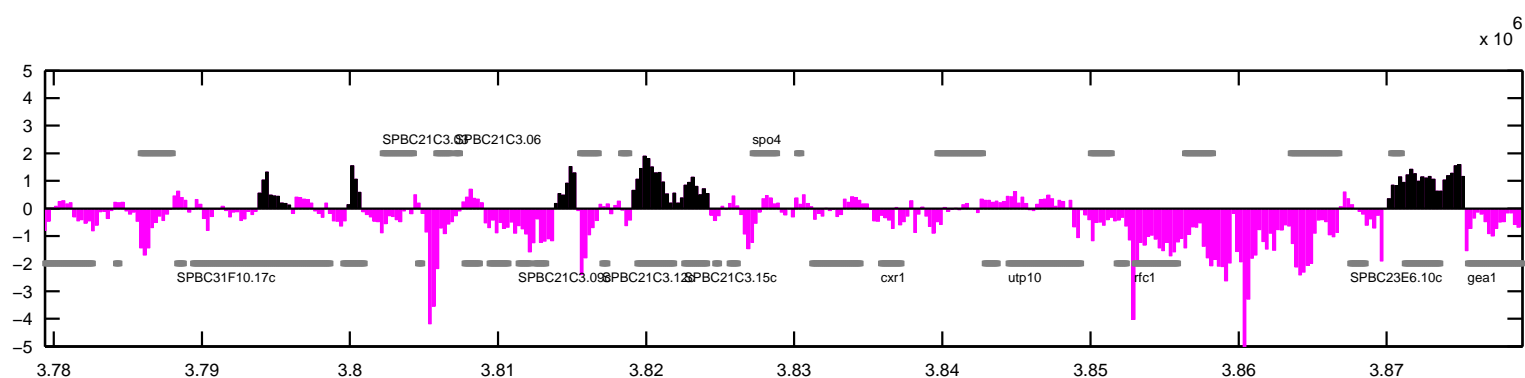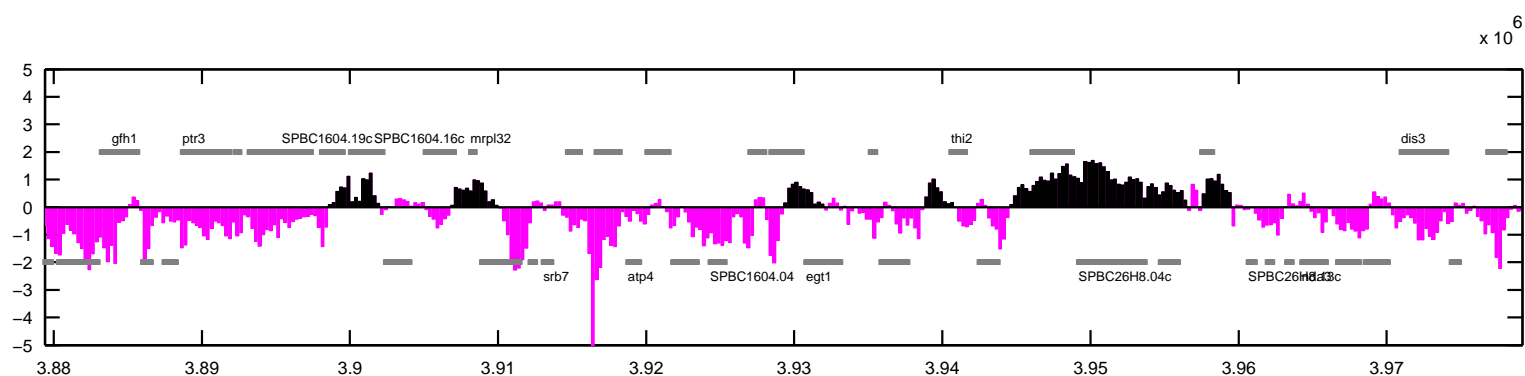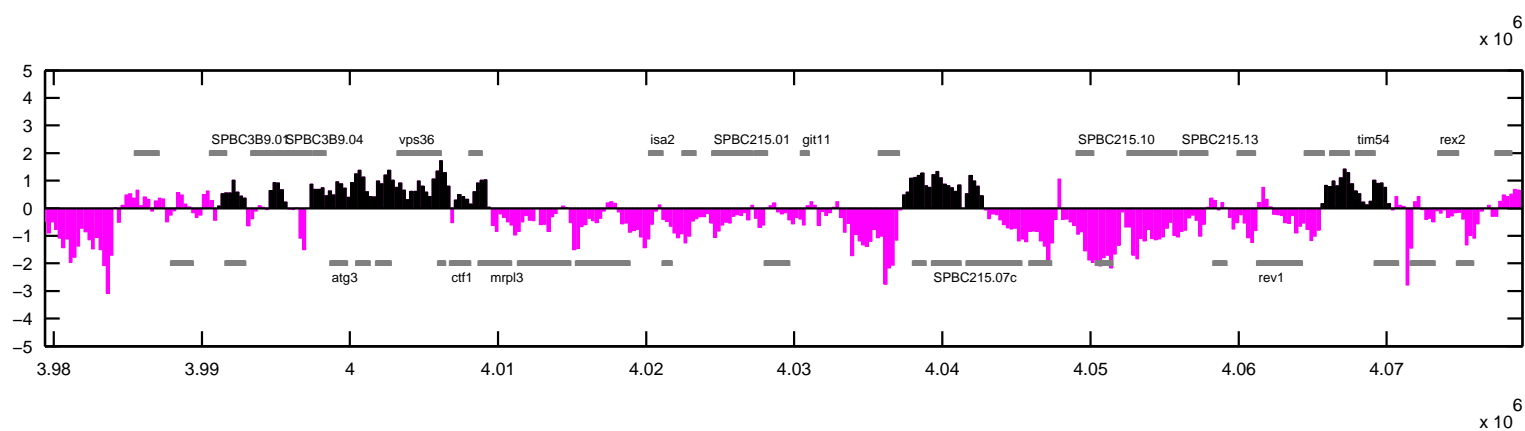

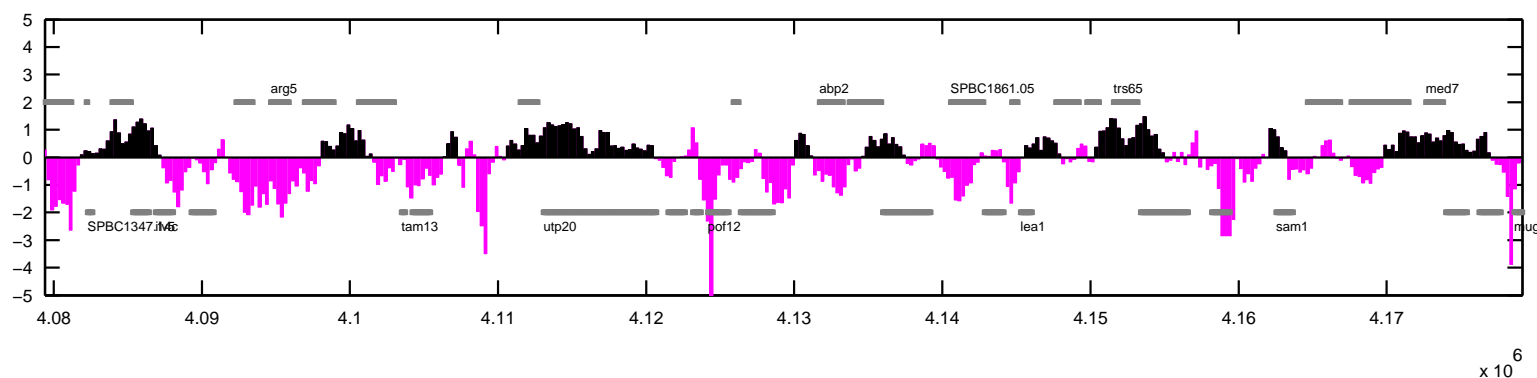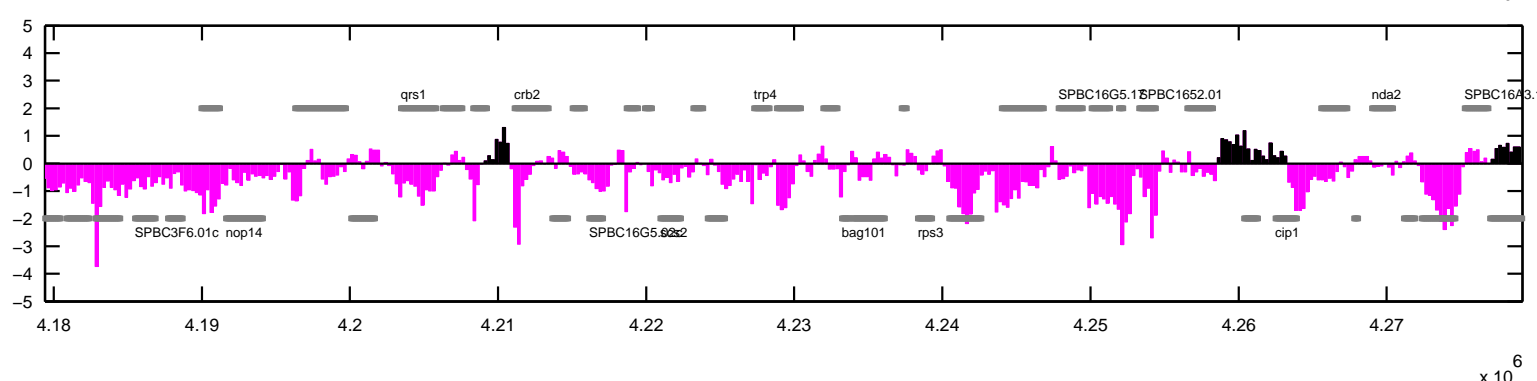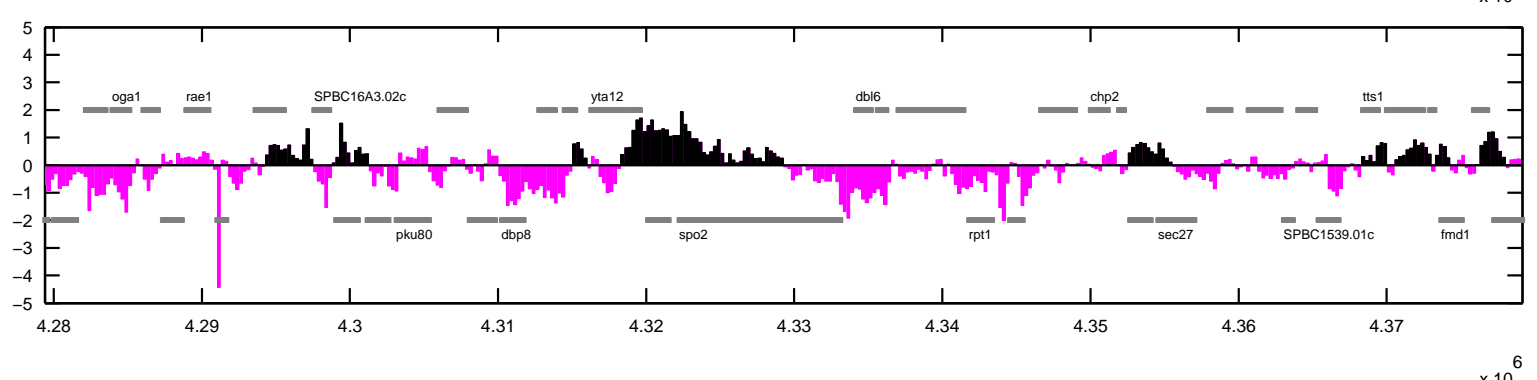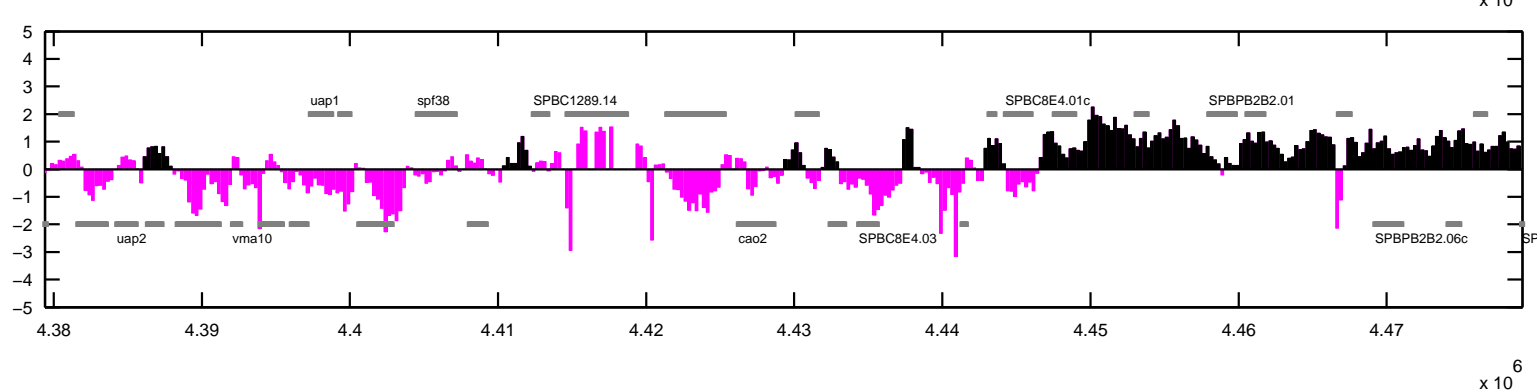

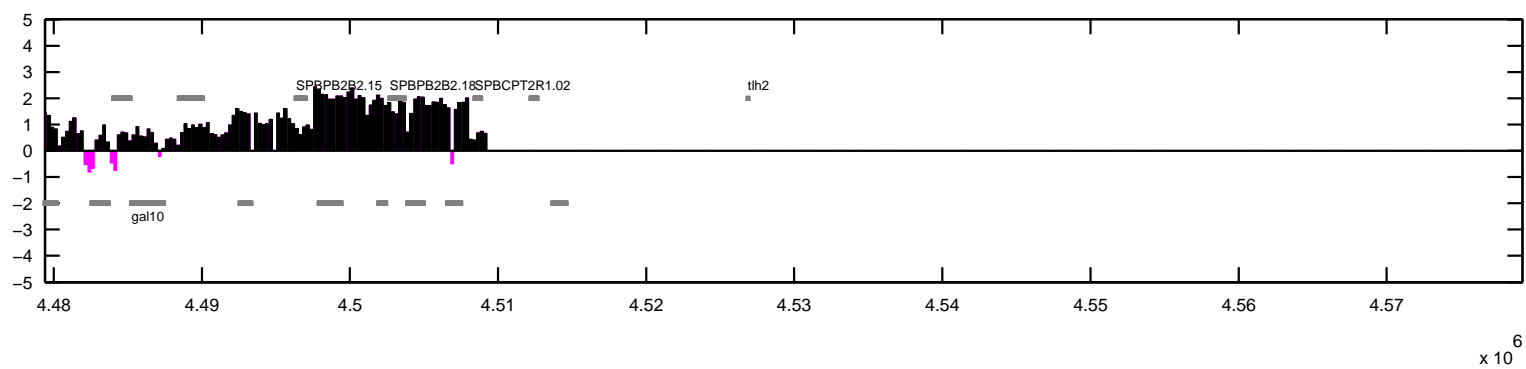

Supplement: SUPPLEMENTARY DATA [file supp_gkw252_nar-00155-v-2016-File008.pdf]

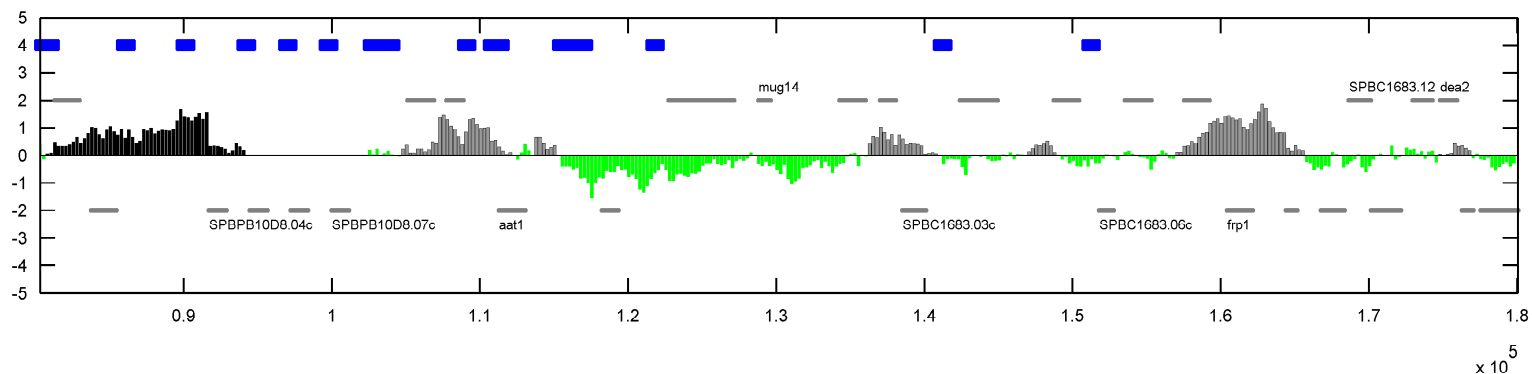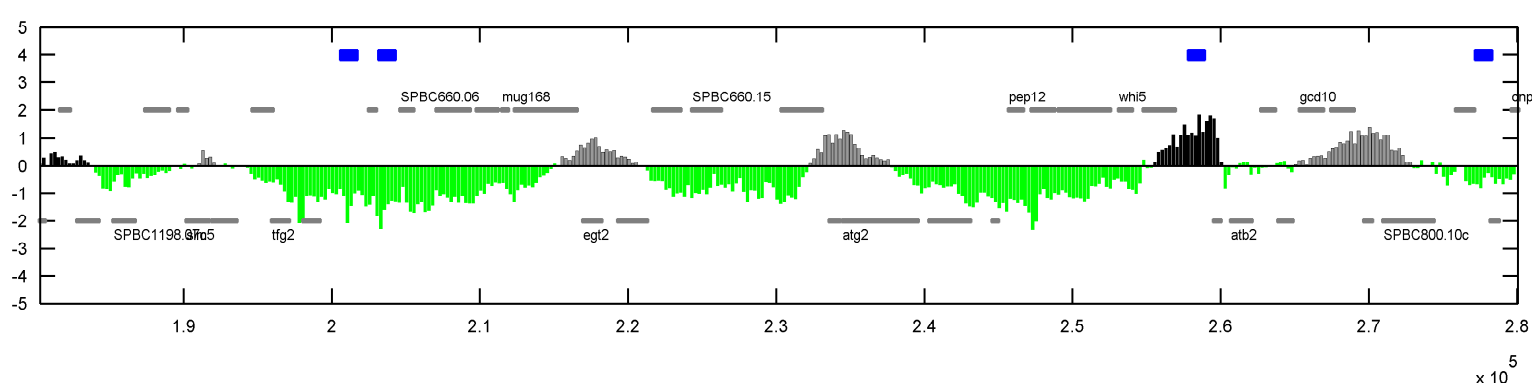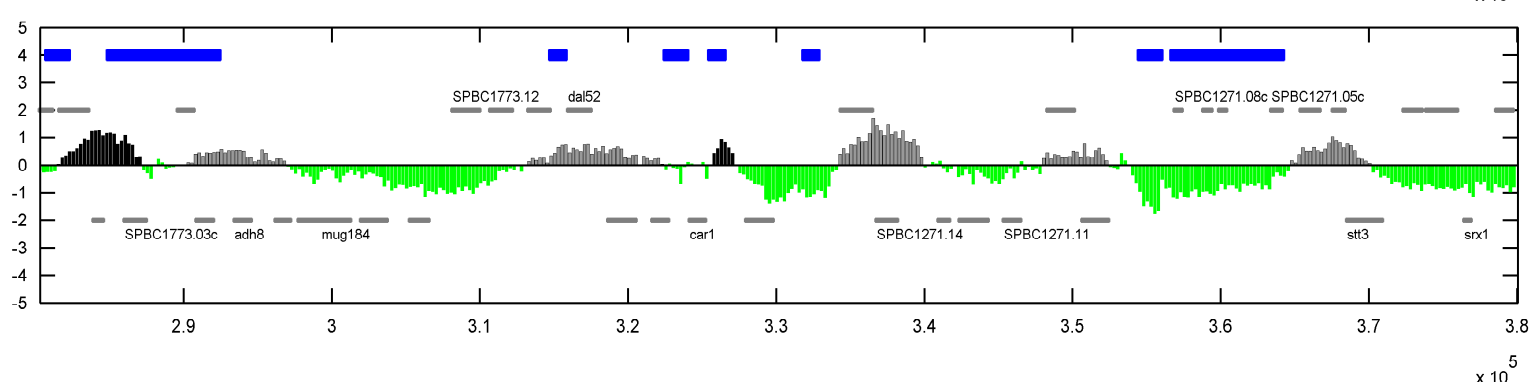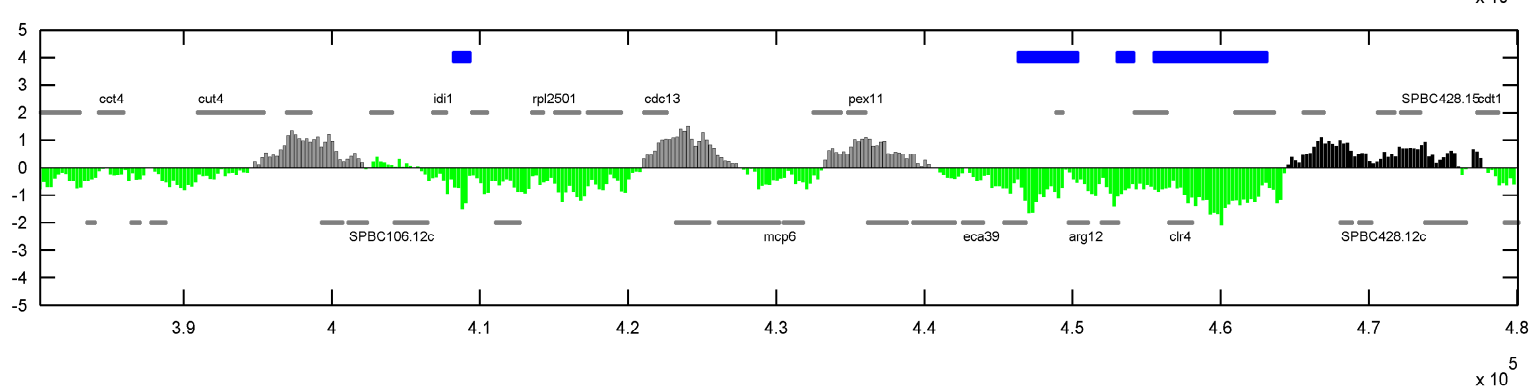

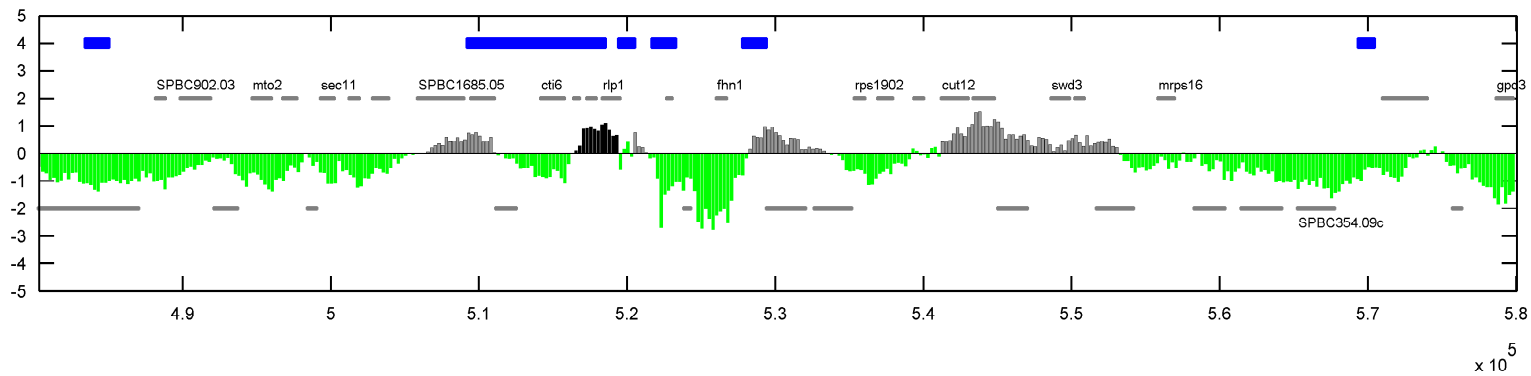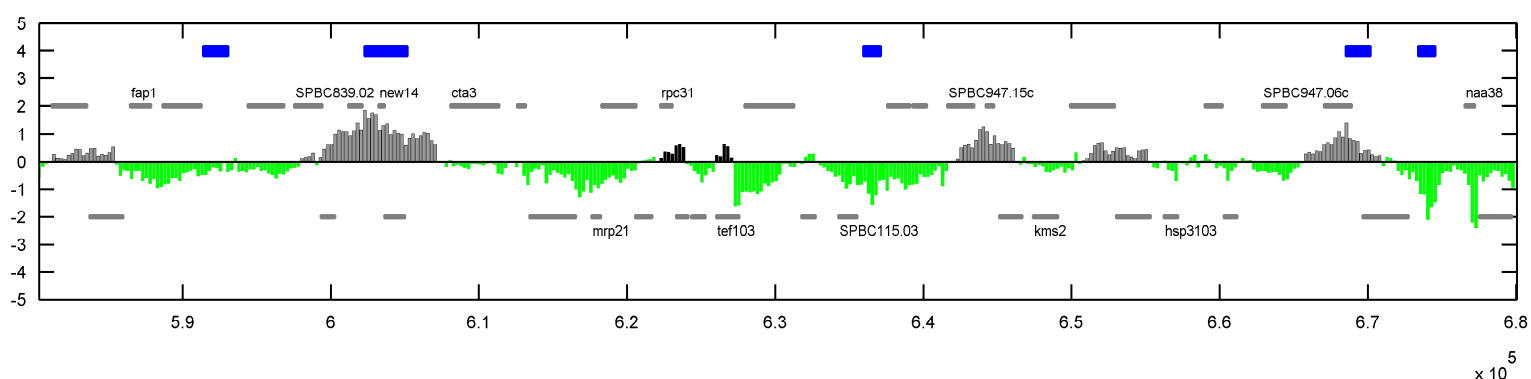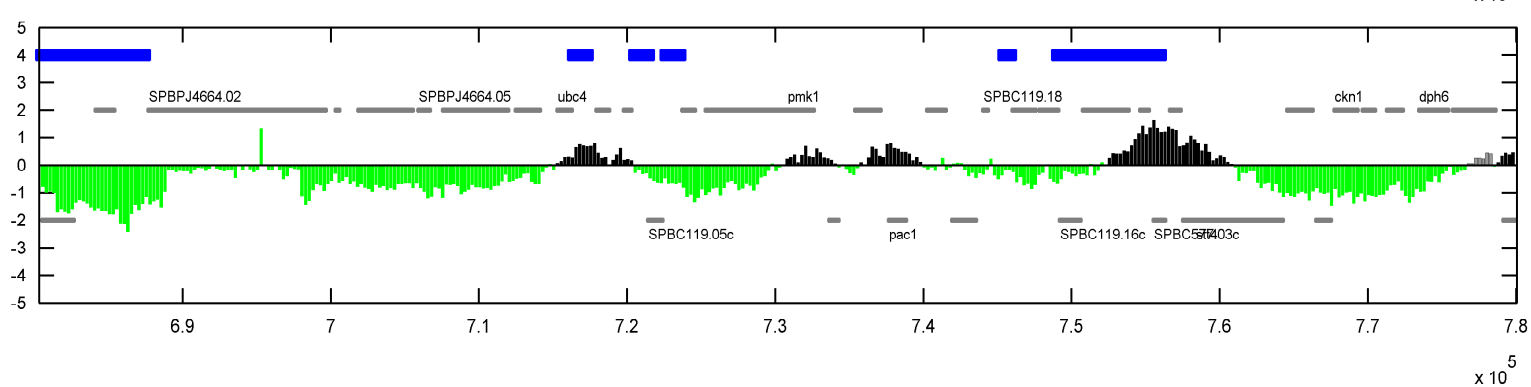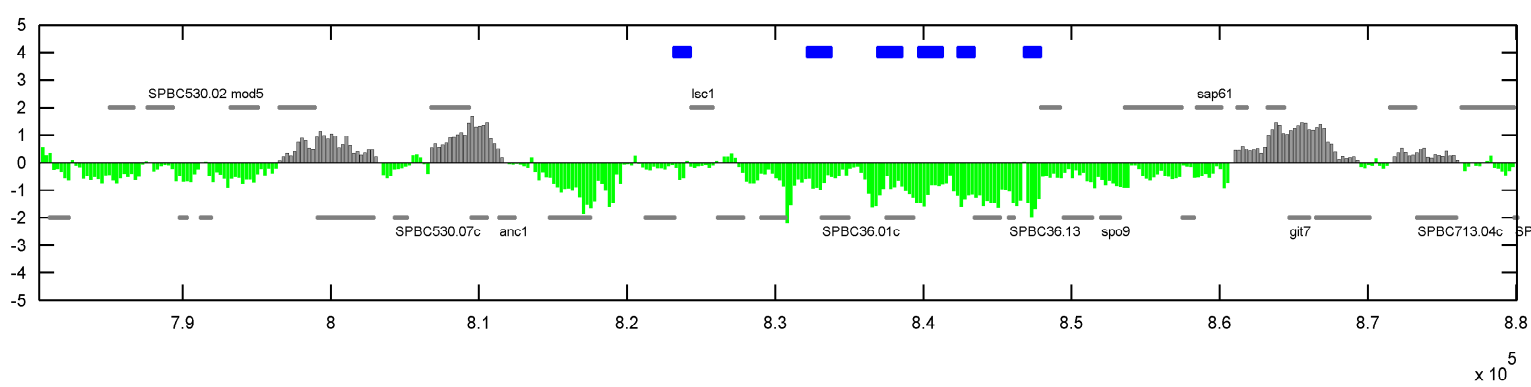

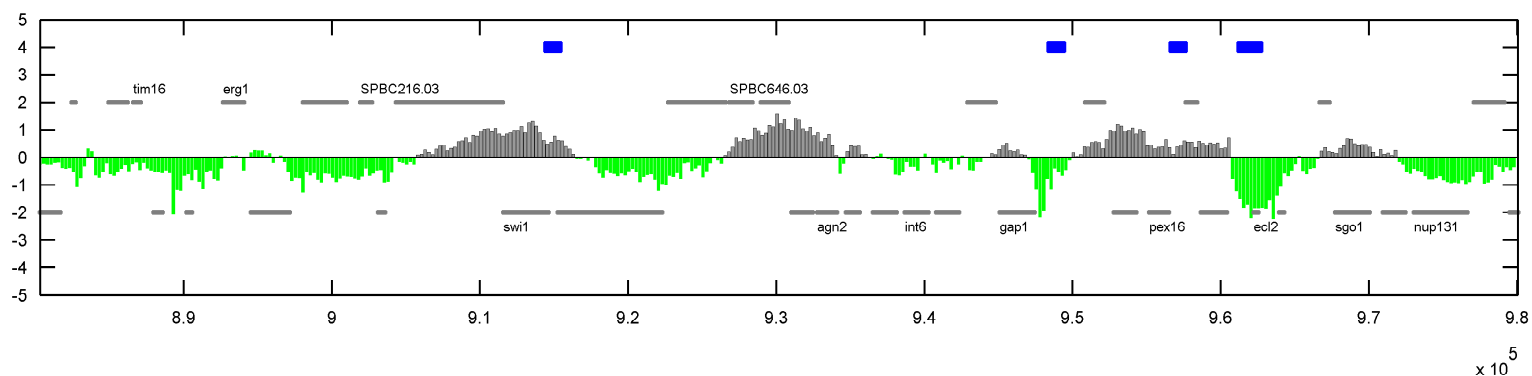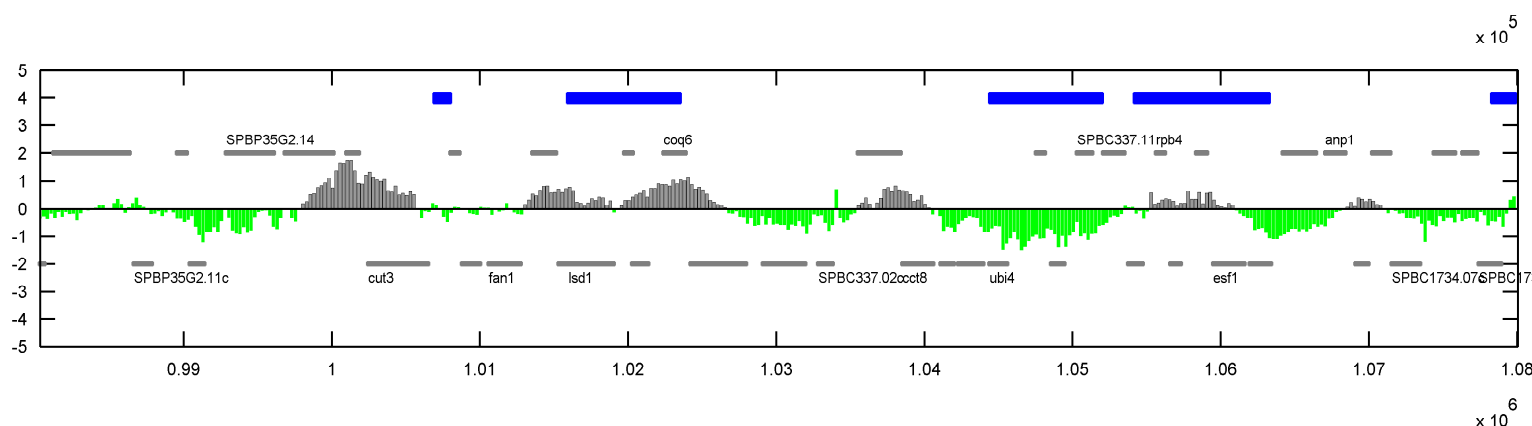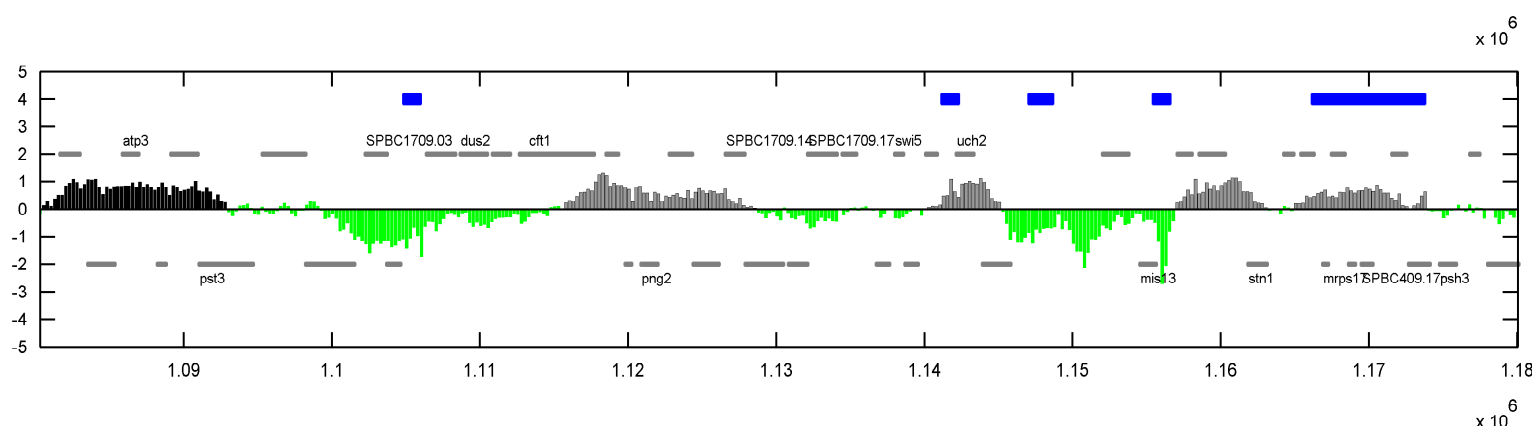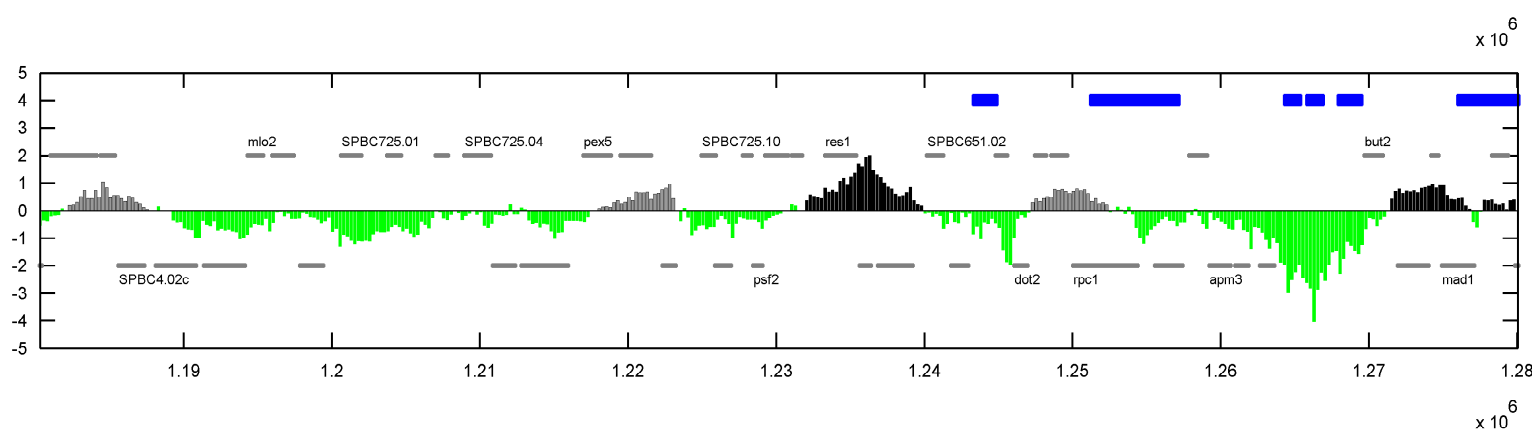

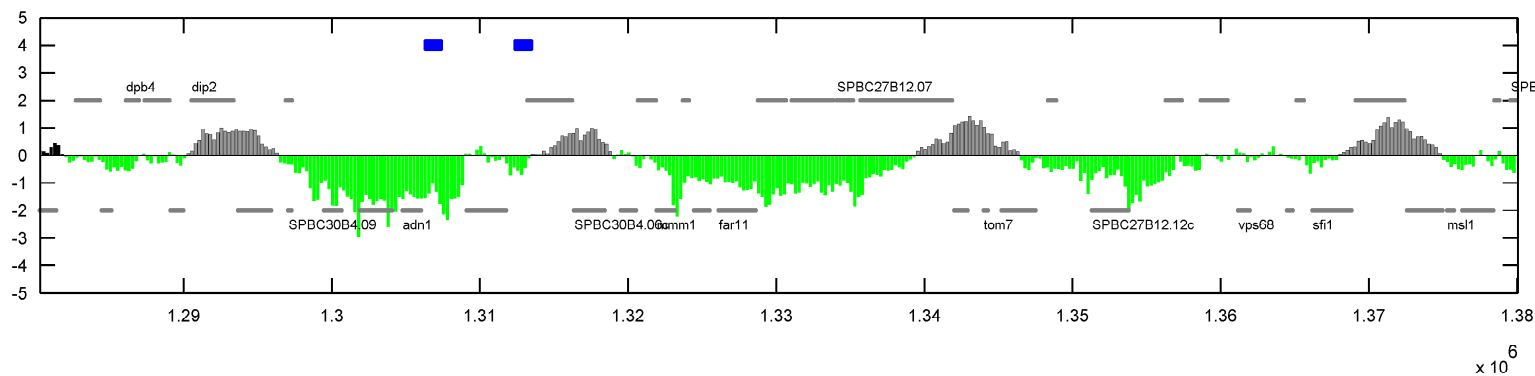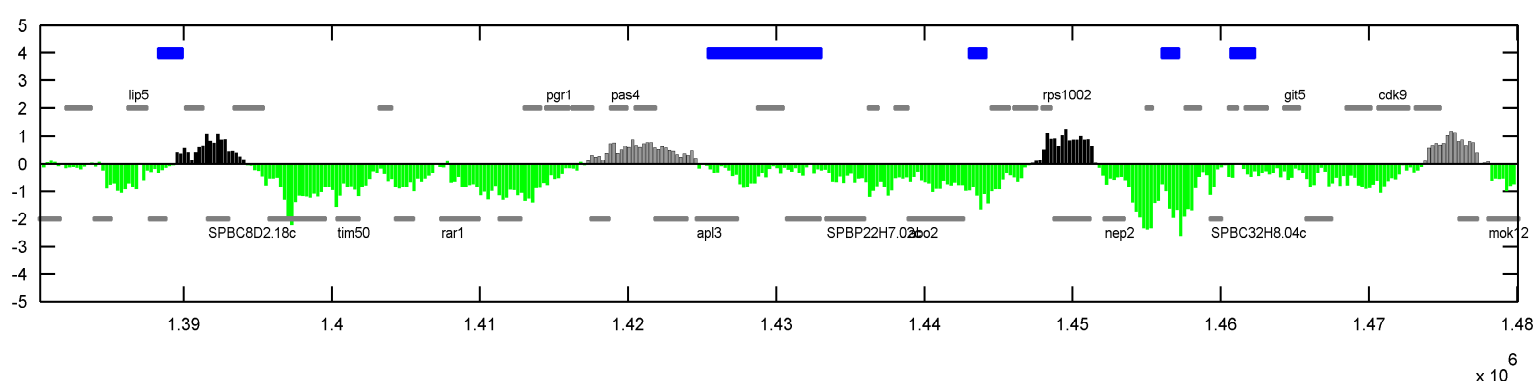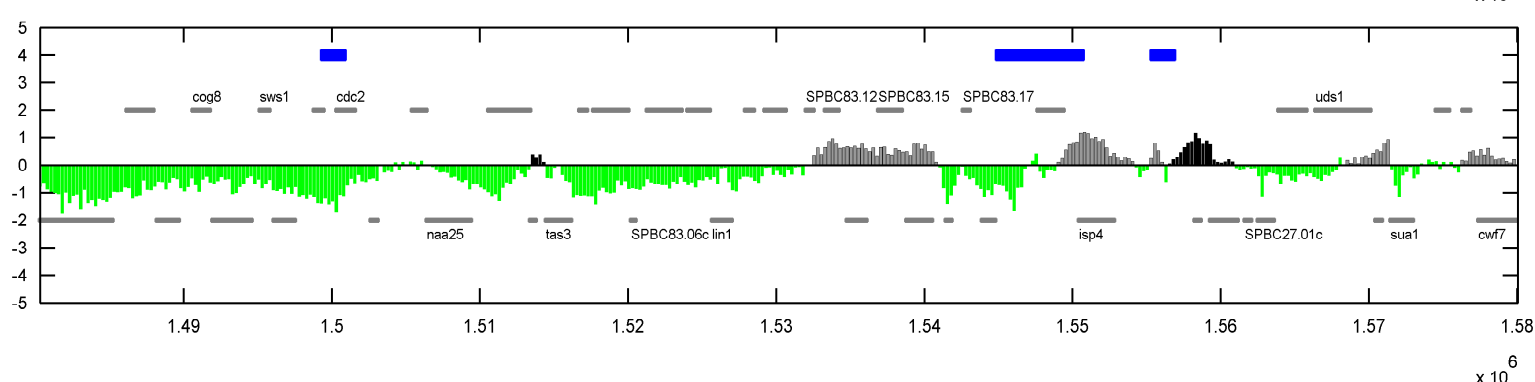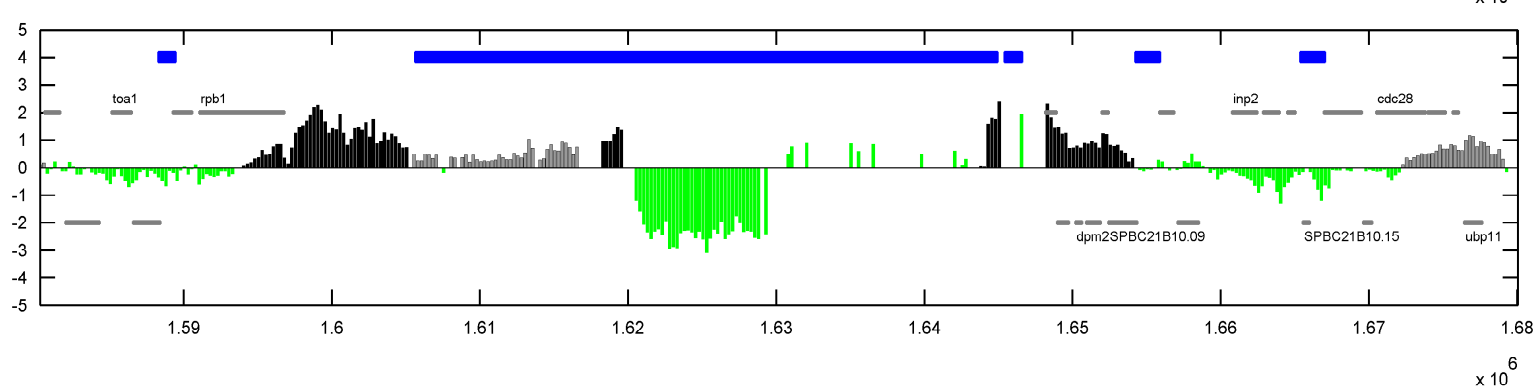

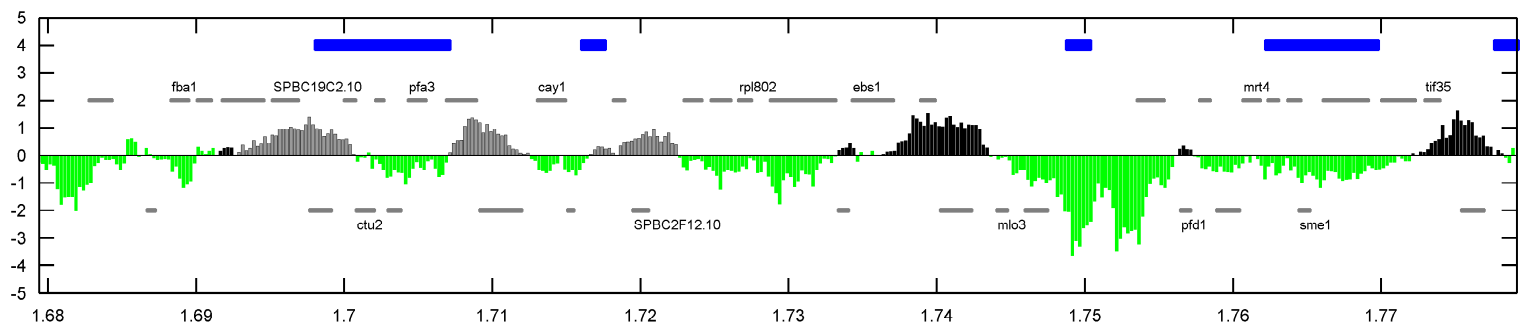

$\times 10^6$

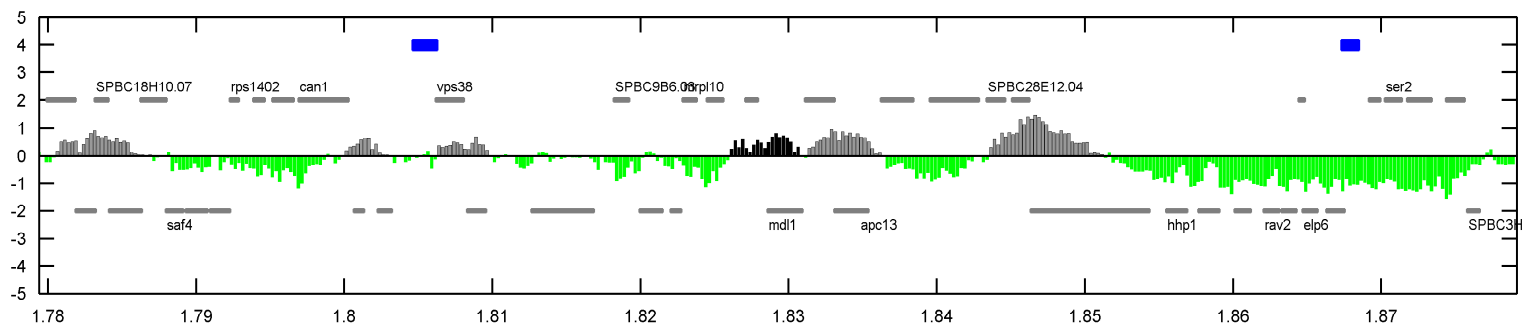

$\times 10^6$

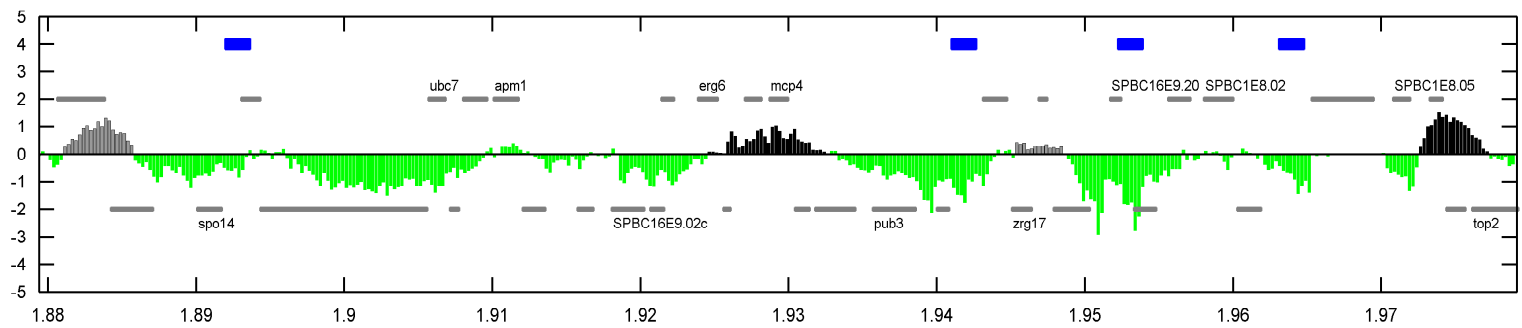

$\times 10^6$

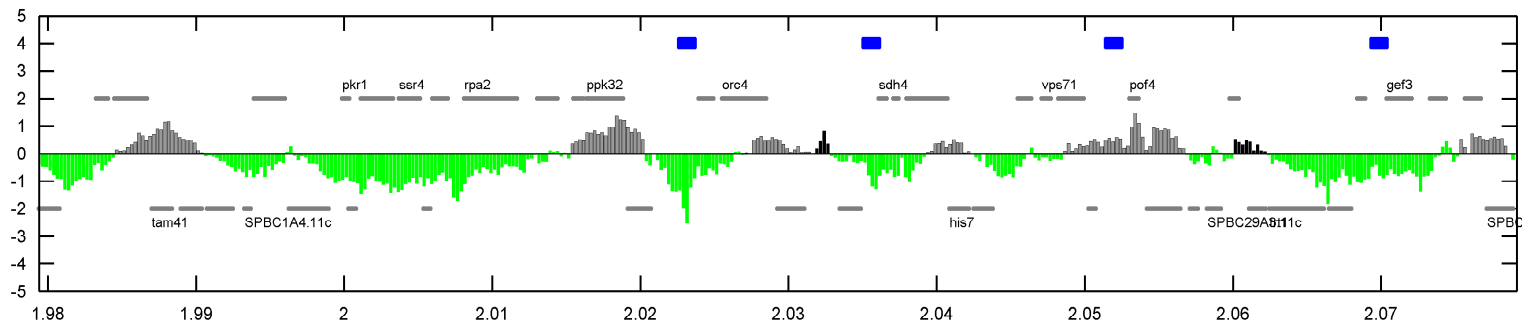

$\times 10^6$

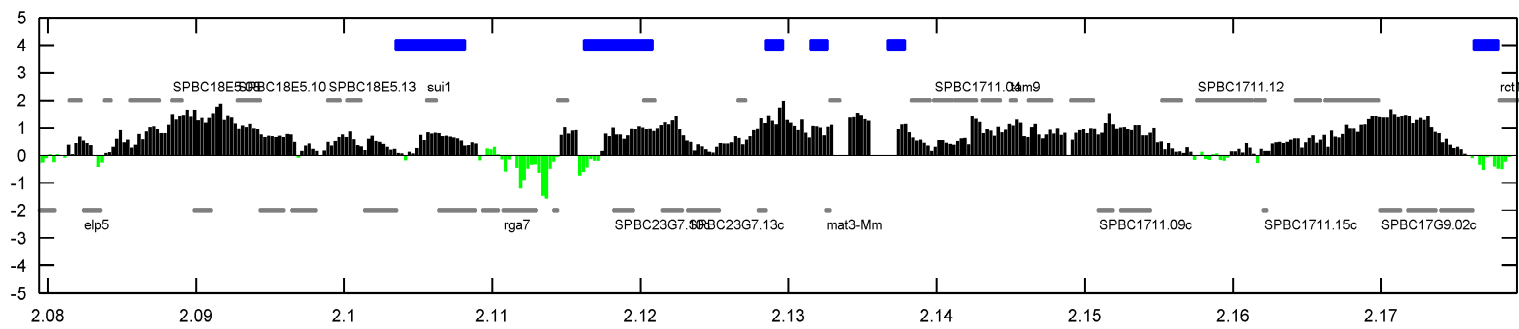

$\times 10^6$

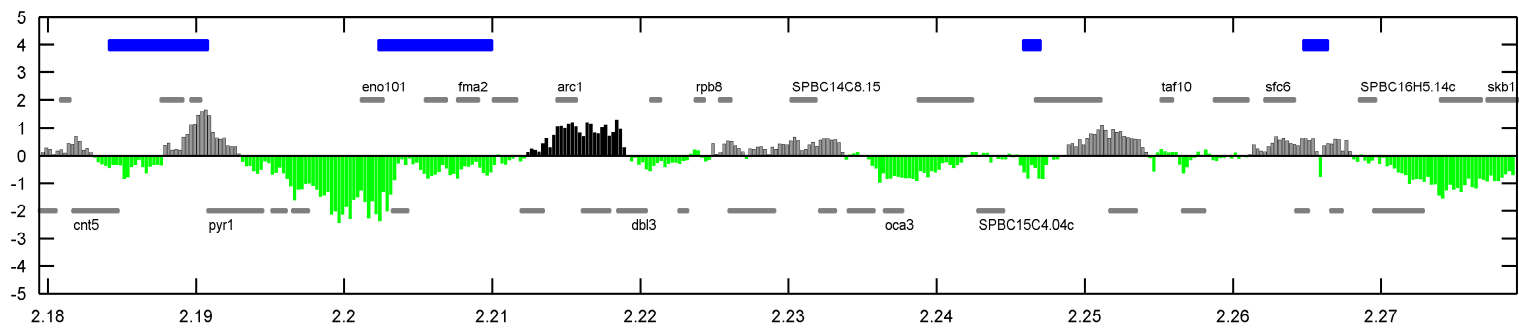

$\times 10^6$

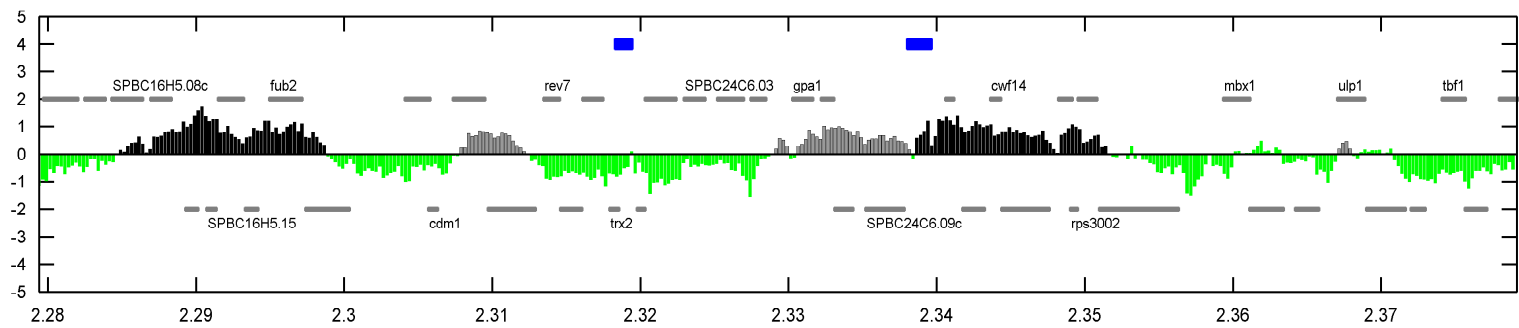

$\times 10^6$

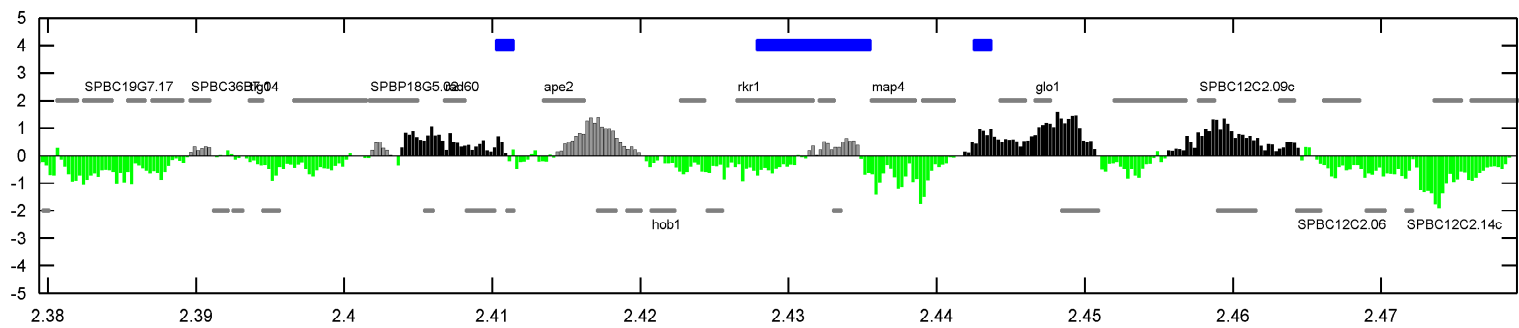

$\times 10^6$



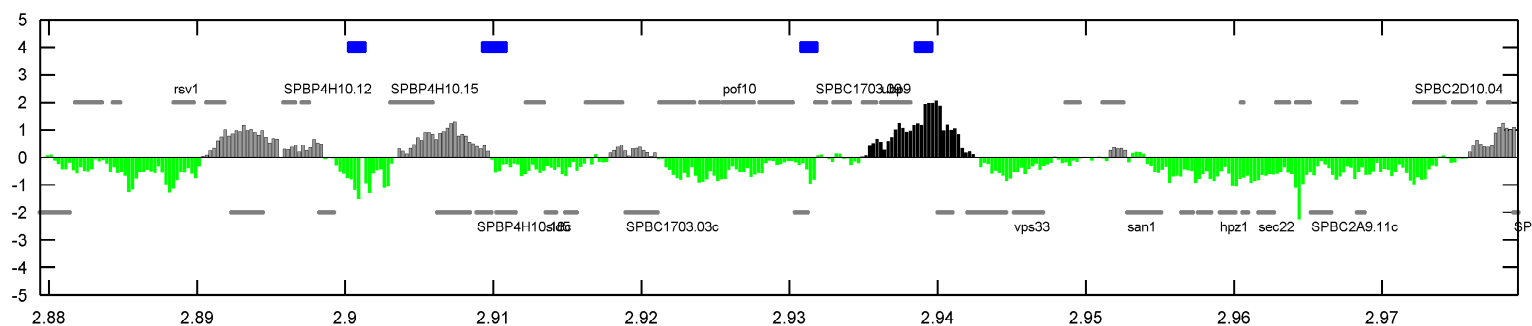

$\times 10^6$

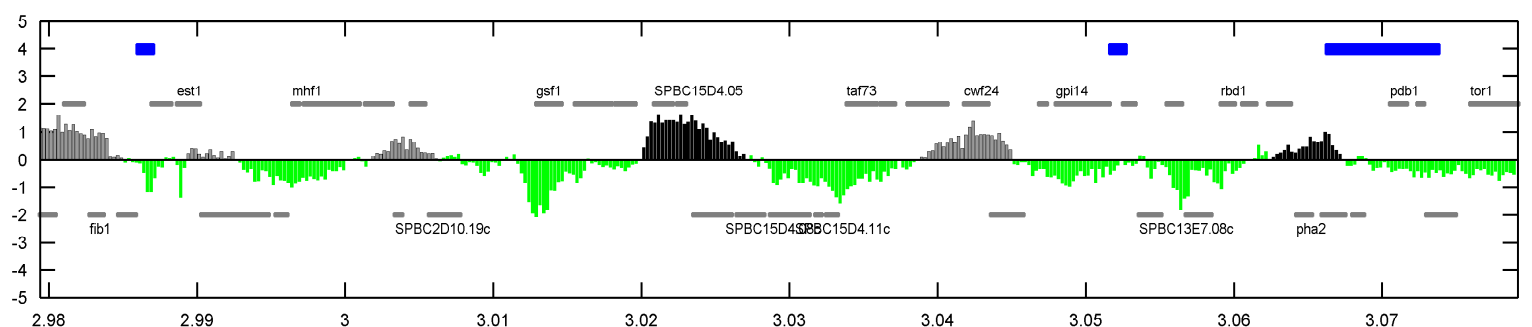

$\times 10^6$

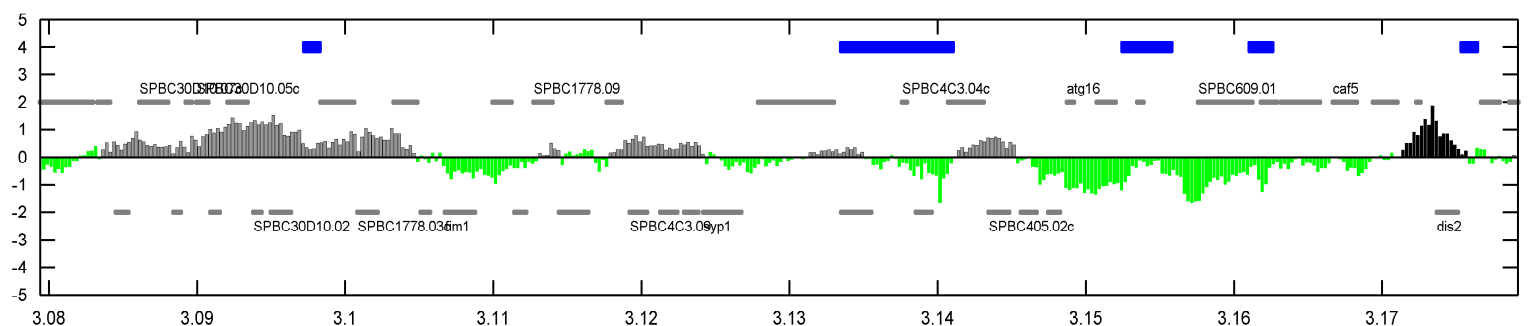

$\times 10^6$

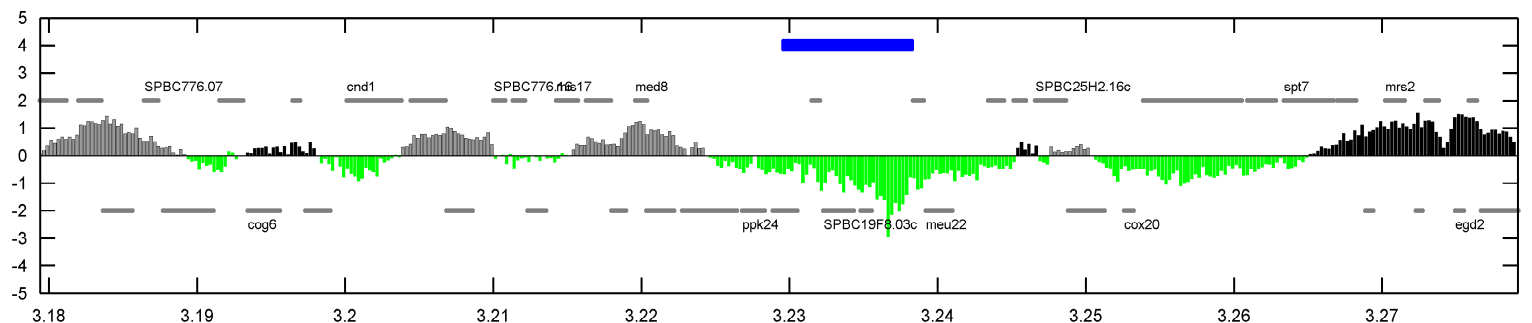

$\times 10^6$

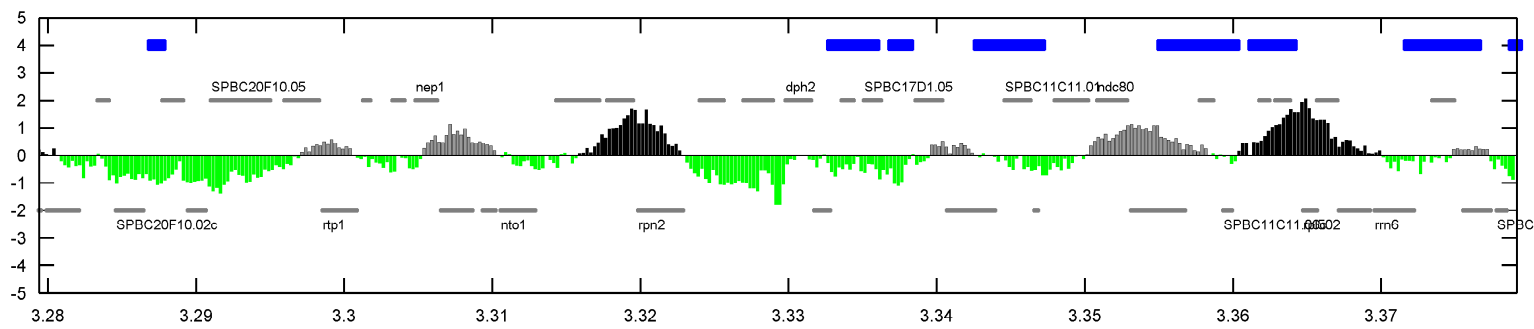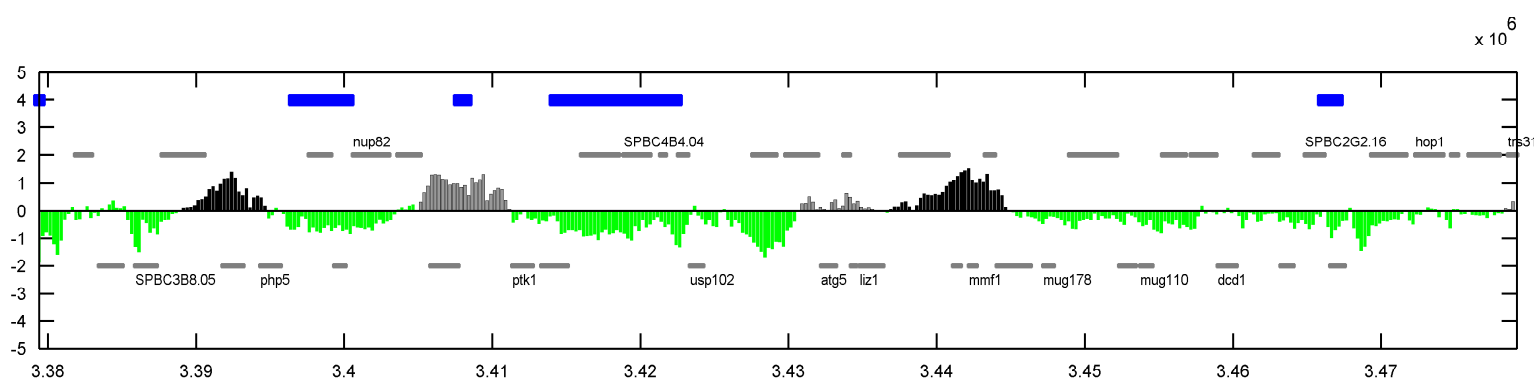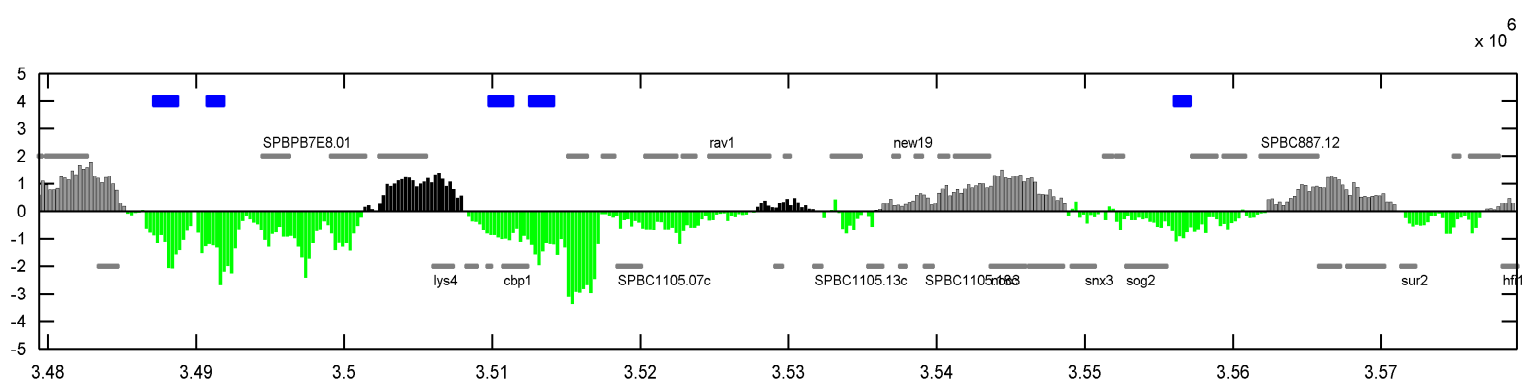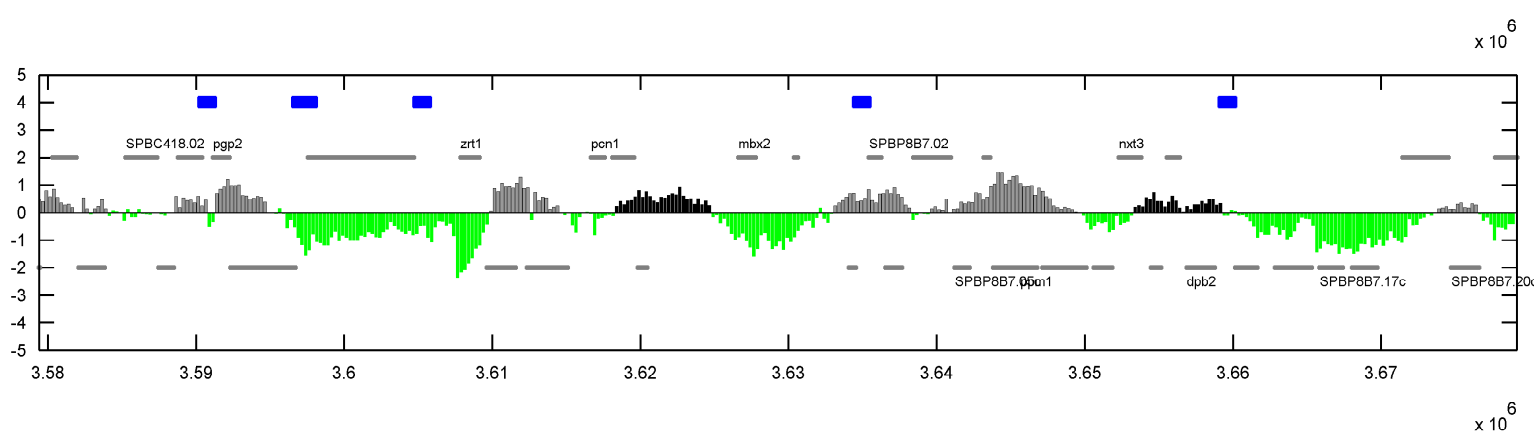

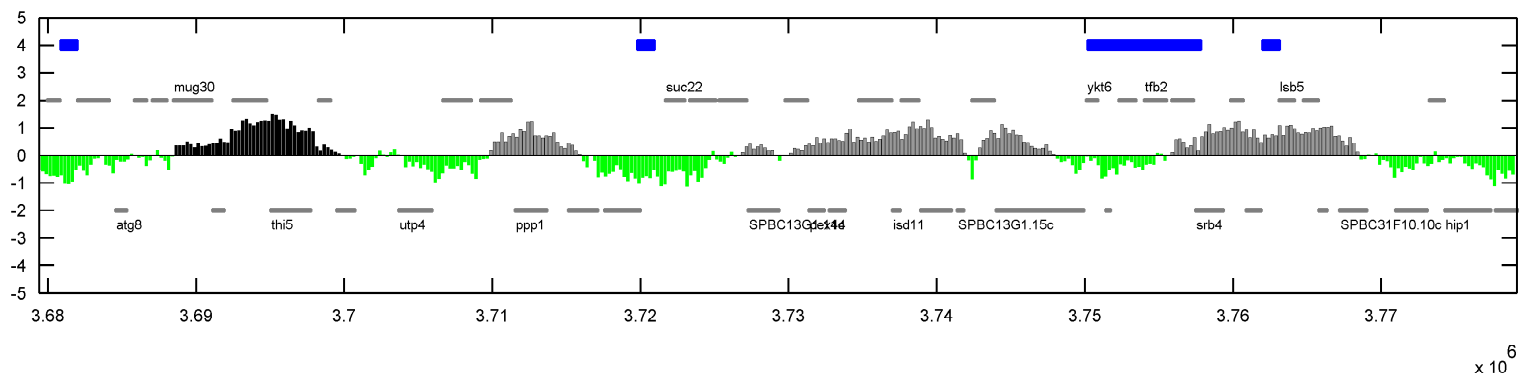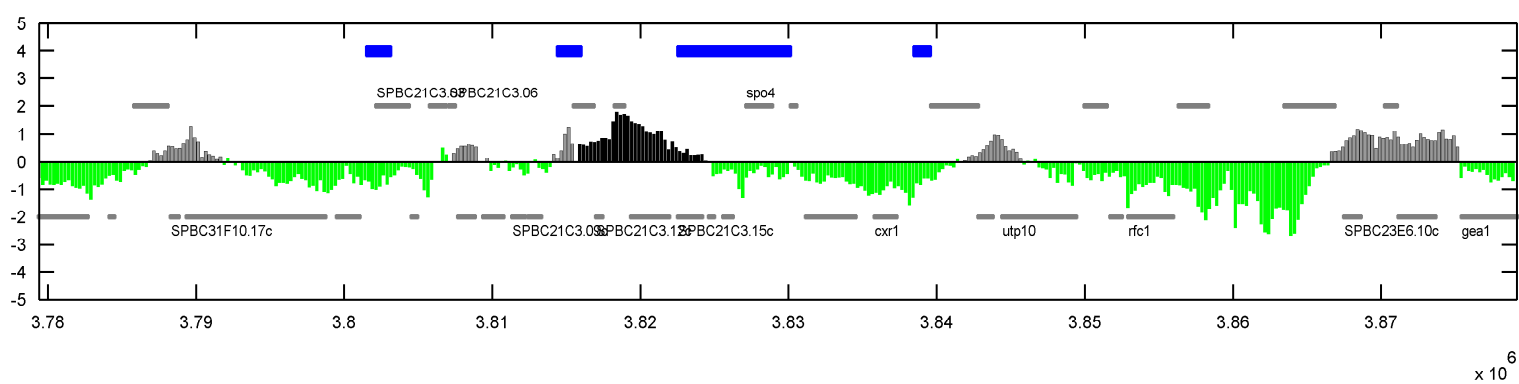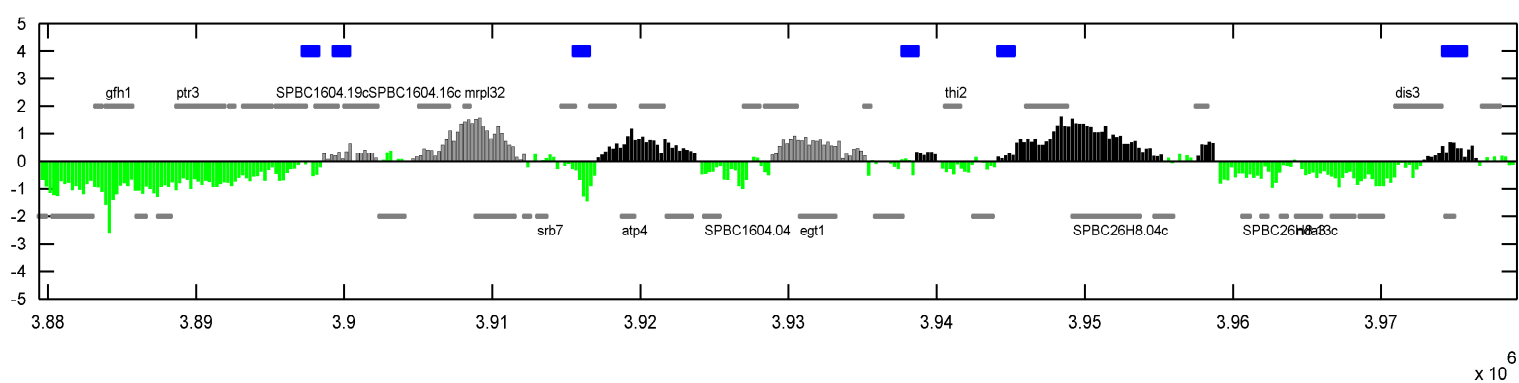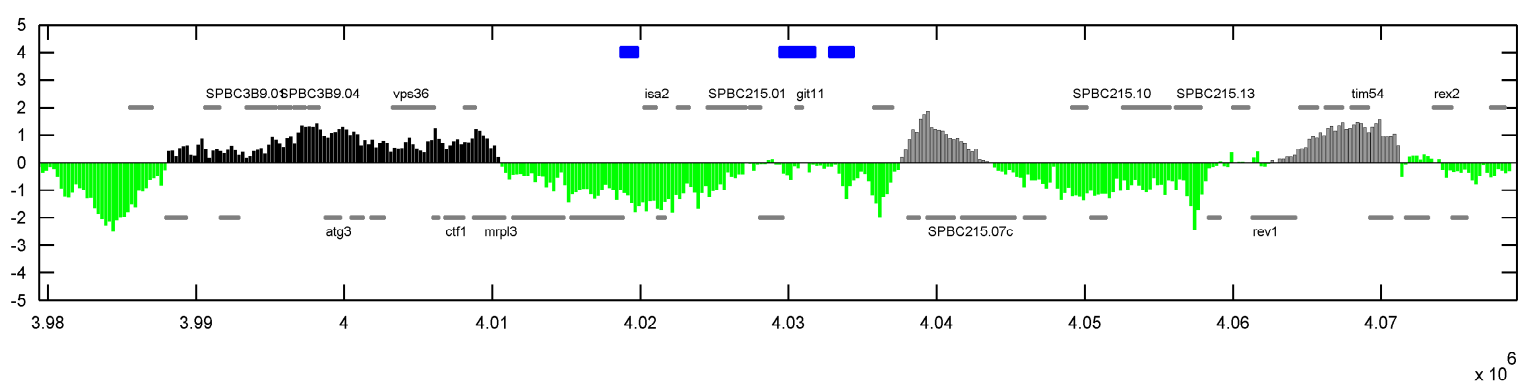

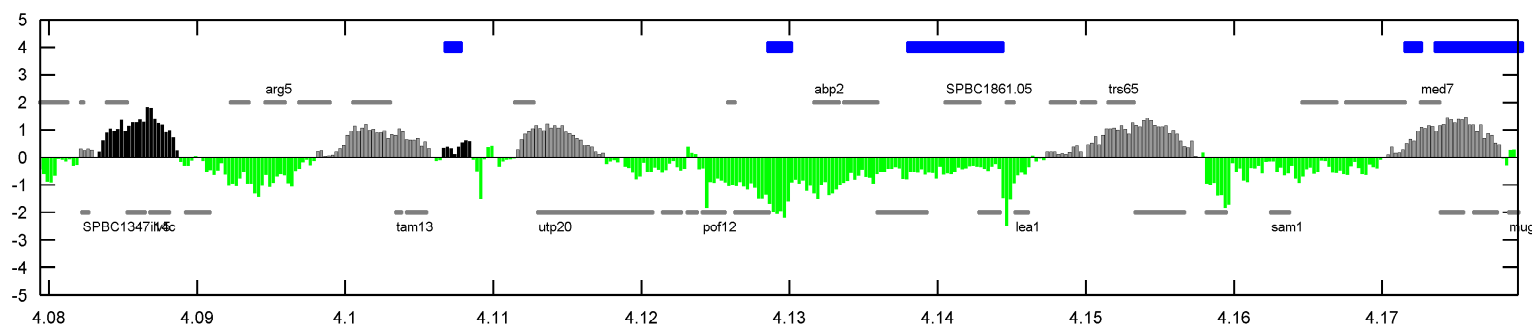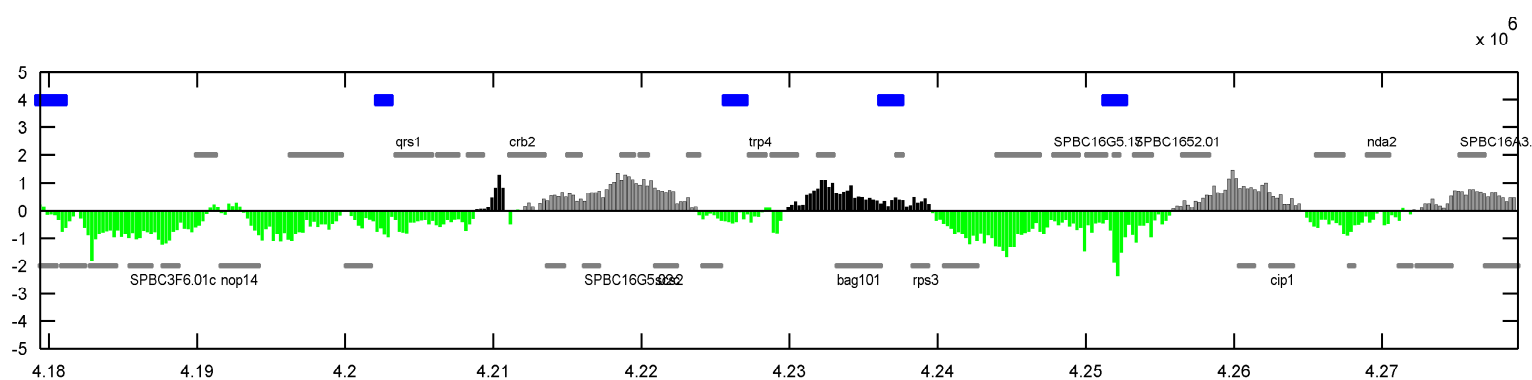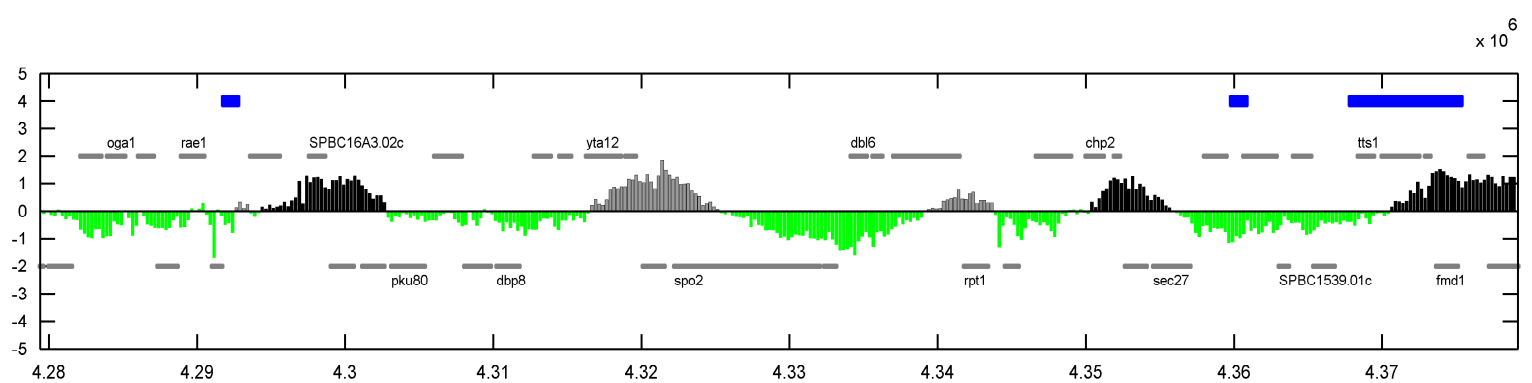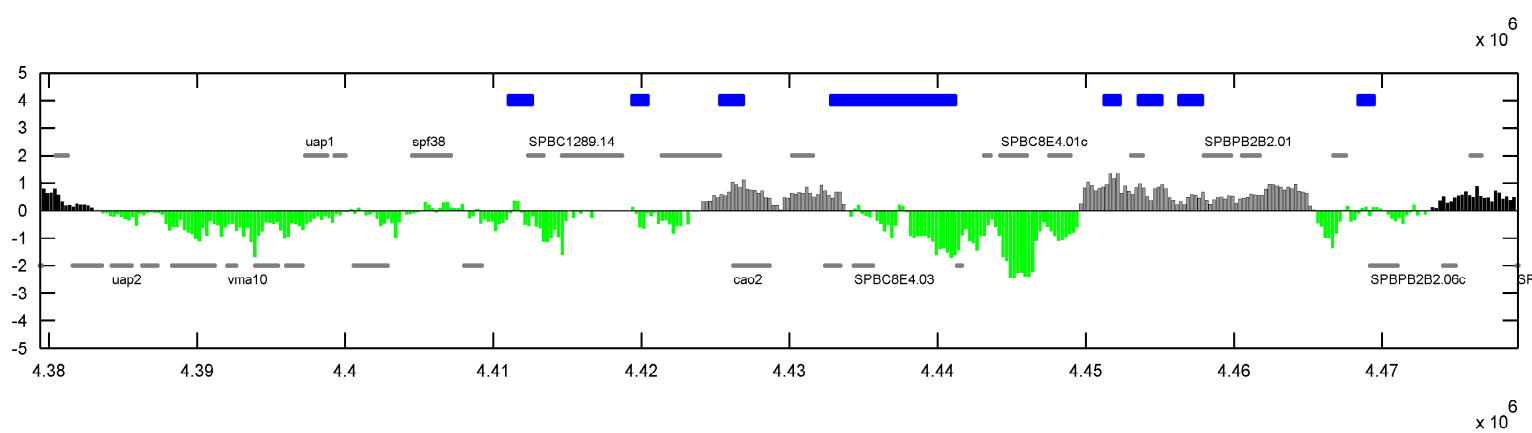

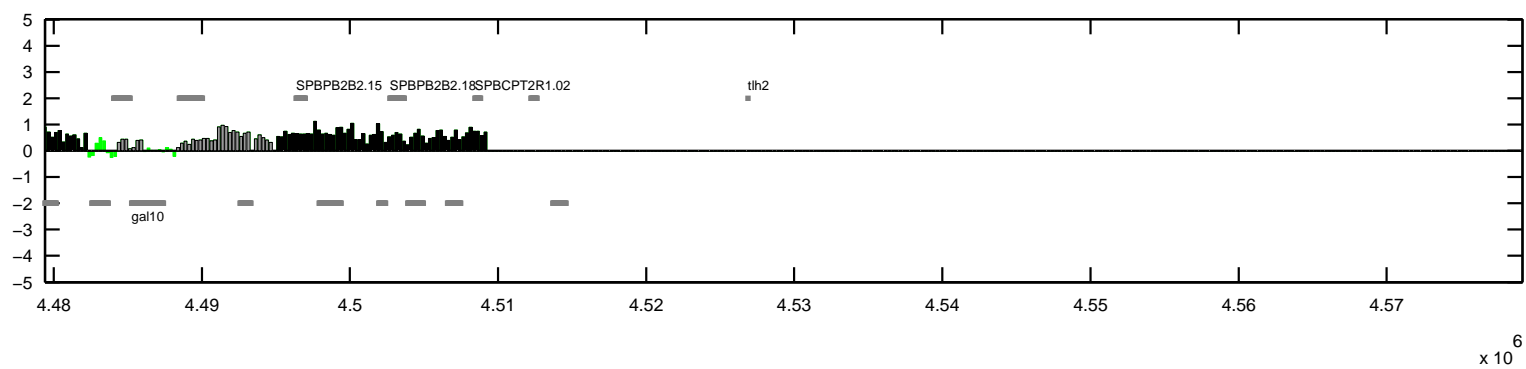

Supplement: SUPPLEMENTARY DATA [file supp_gkw252_nar-00155-v-2016-File009.pdf]
