# Supplementary material for: Transcription facilitates sister chromatid cohesion on chromosomal arms
Source: Nucleic Acids Res. 2016 Apr 15;44(14):6676–92. doi: 10.1093/nar/gkw252 (PMC5001582; doi:10.1093/nar/gkw252)
Supplement: SUPPLEMENTARY DATA [file supp_gkw252_nar-00155-v-2016-File010.pdf]

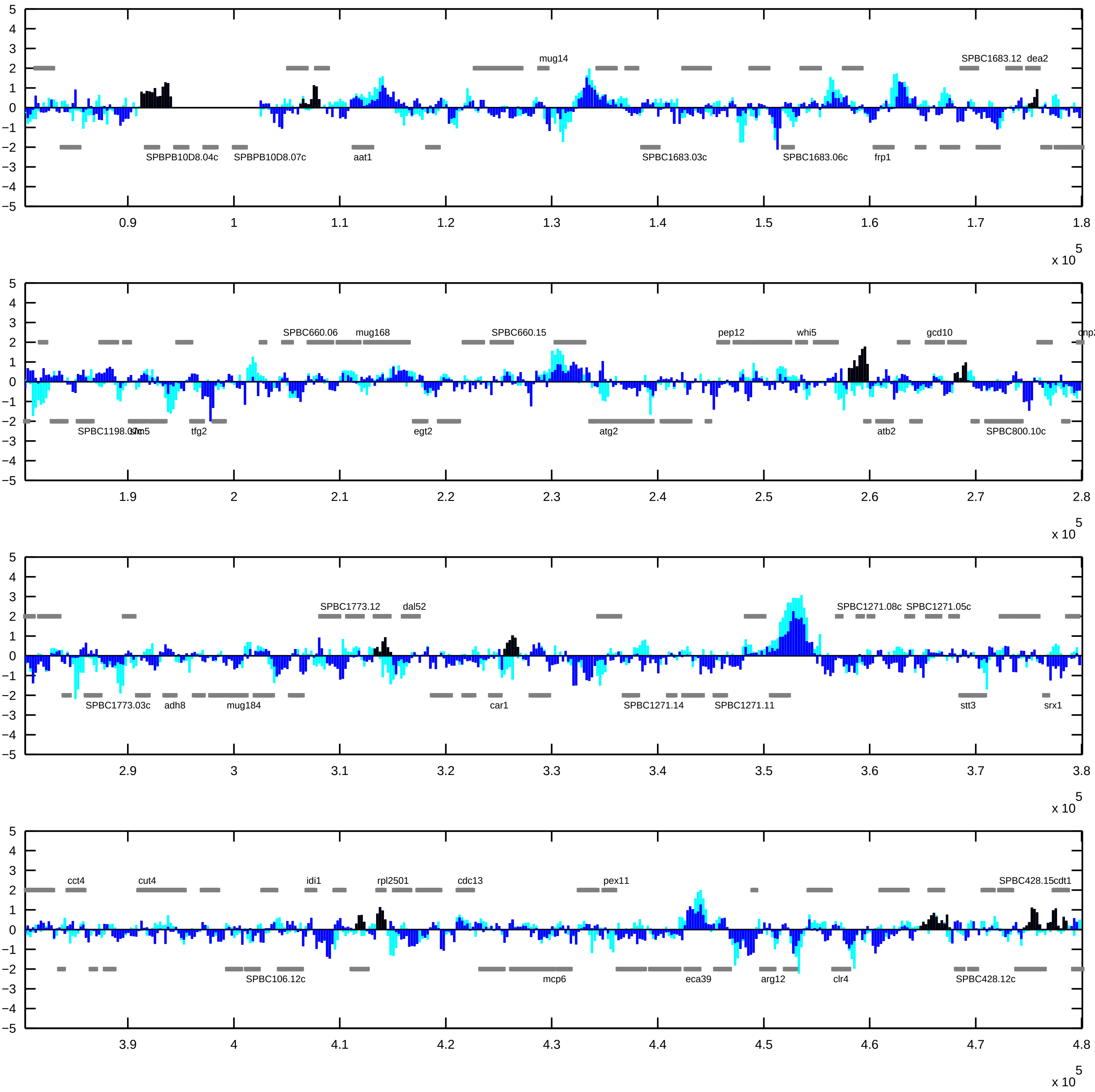
mug14
SPBC1683.12
dea2
SPBPB10D8.04c
SPBPB10D8.07c
aat1
SPBC1683.03c
SPBC1683.06c
frp1
SPBC660.06
mug168
SPBC660.15
pep12
whi5
gcd10
tfg2
egt2
atg2
atb2
SPBC800.10c
SPBC1773.12
dal52
SPBC1271.08c
SPBC1271.05c
SPBC1773.03c
adh8
mug184
car1
SPBC1271.14
SPBC1271.11
stt3
srx1
cct4
cut4
idi1
rpl2501
cdc13
pex11
SPBC428.15cdt1
SPBC106.12c
mcp6
eca39
arg12
clr4
SPBC428.12c
x 10^5

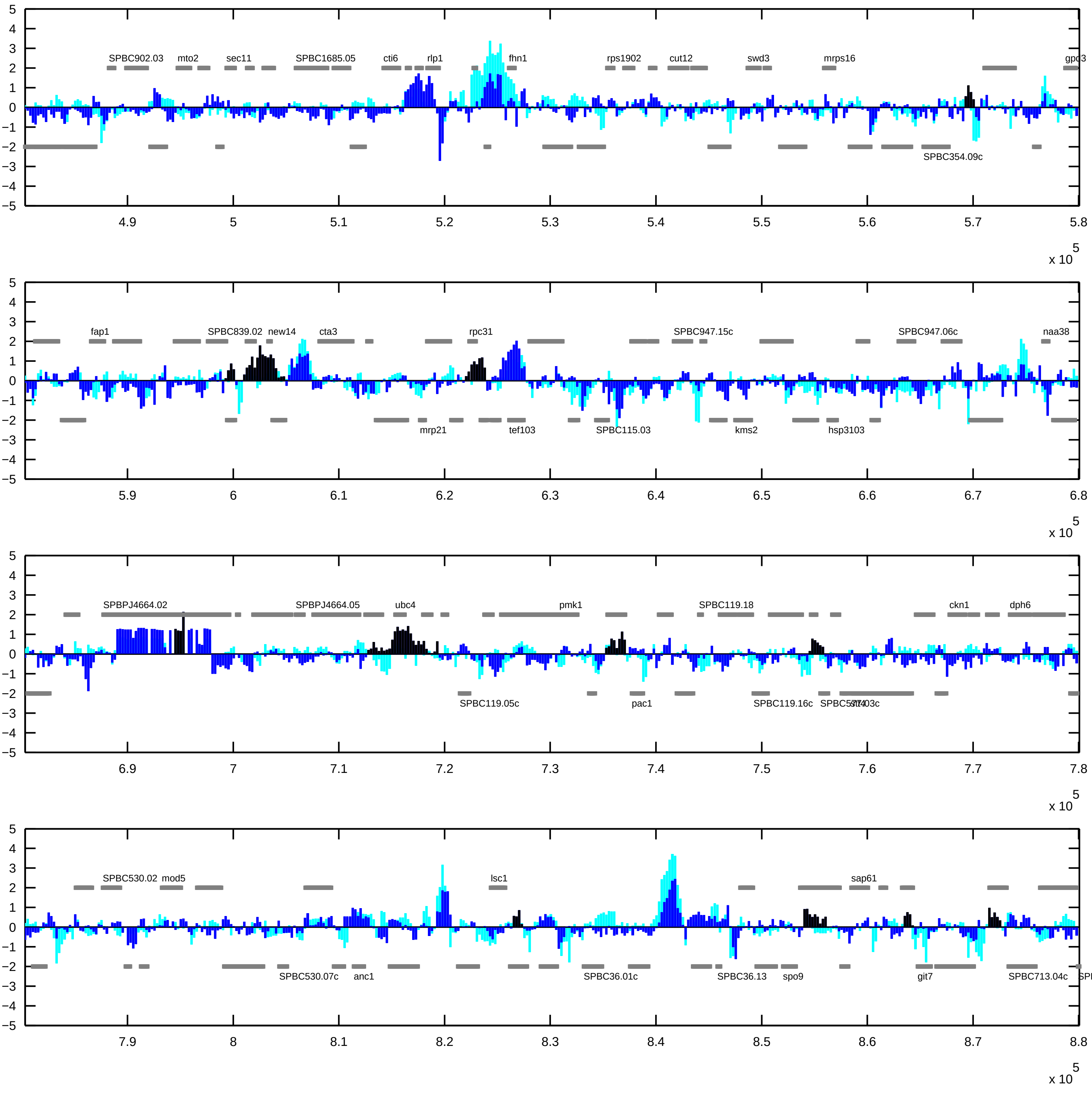
SPBC902.03
mto2
sec11
SPBC1685.05
cti6
rlp1
fhn1
rps1902
cut12
swd3
mrps16
SPBC354.09c
4.9
5
5.1
5.2
5.3
5.4
5.5
5.6
5.7
5.8
x 10^5
fap1
SPBC839.02
new14
cta3
rpc31
SPBC947.15c
SPBC947.06c
naa38
mrp21
tef103
SPBC115.03
kms2
hsp3103
5.9
6
6.1
6.2
6.3
6.4
6.5
6.6
6.7
6.8
x 10^5
SPBPJ4664.02
SPBPJ4664.05
ubc4
pmk1
SPBC119.18
ckn1
dph6
SPBC119.05c
pac1
SPBC119.16c
6.9
7
7.1
7.2
7.3
7.4
7.5
7.6
7.7
7.8
x 10^5
SPBC530.02
mod5
lsc1
sap61
SPBC530.07c
anc1
SPBC36.01c
SPBC36.13
spo9
git7
SPBC713.04c
7.9
8
8.1
8.2
8.3
8.4
8.5
8.6
8.7
8.8
x 10^5

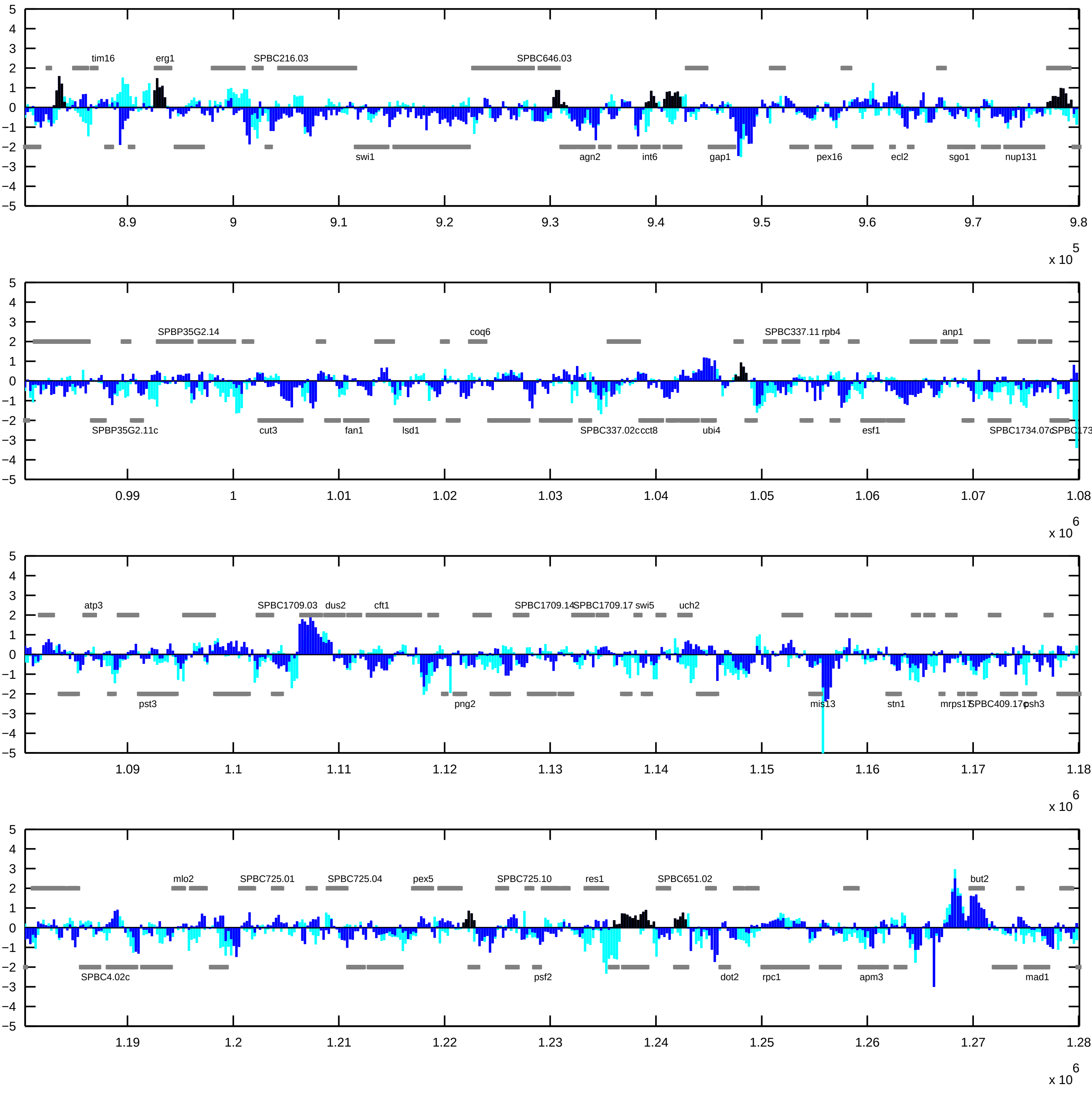
tim16
erg1
SPBC216.03
SPBC646.03
swi1
agn2
int6
gap1
pex16
ecl2
sgo1
nup131
8.9 9 9.1 9.2 9.3 9.4 9.5 9.6 9.7 9.8
x 10^5
SPBP35G2.14
coq6
SPBC337.11 rpb4
anp1
SPBP35G2.11c
cut3
fan1
lsd1
SPBC337.02c cct8
ubi4
esf1
SPBC1734.07c
0.99 1 1.01 1.02 1.03 1.04 1.05 1.06 1.07 1.08
x 10^6
atp3
SPBC1709.03
dus2
cft1
SPBC1709.14 SPBC1709.17 swi5
uch2
pst3
png2
mis13
stn1
mrps17 SPBC409.17c psh3
1.09 1.1 1.11 1.12 1.13 1.14 1.15 1.16 1.17 1.18
x 10^6
mlo2
SPBC725.01
SPBC725.04
pex5
SPBC725.10
res1
SPBC651.02
but2
SPBC4.02c
psf2
dot2
rpc1
apm3
mad1
1.19 1.2 1.21 1.22 1.23 1.24 1.25 1.26 1.27 1.28
x 10^6

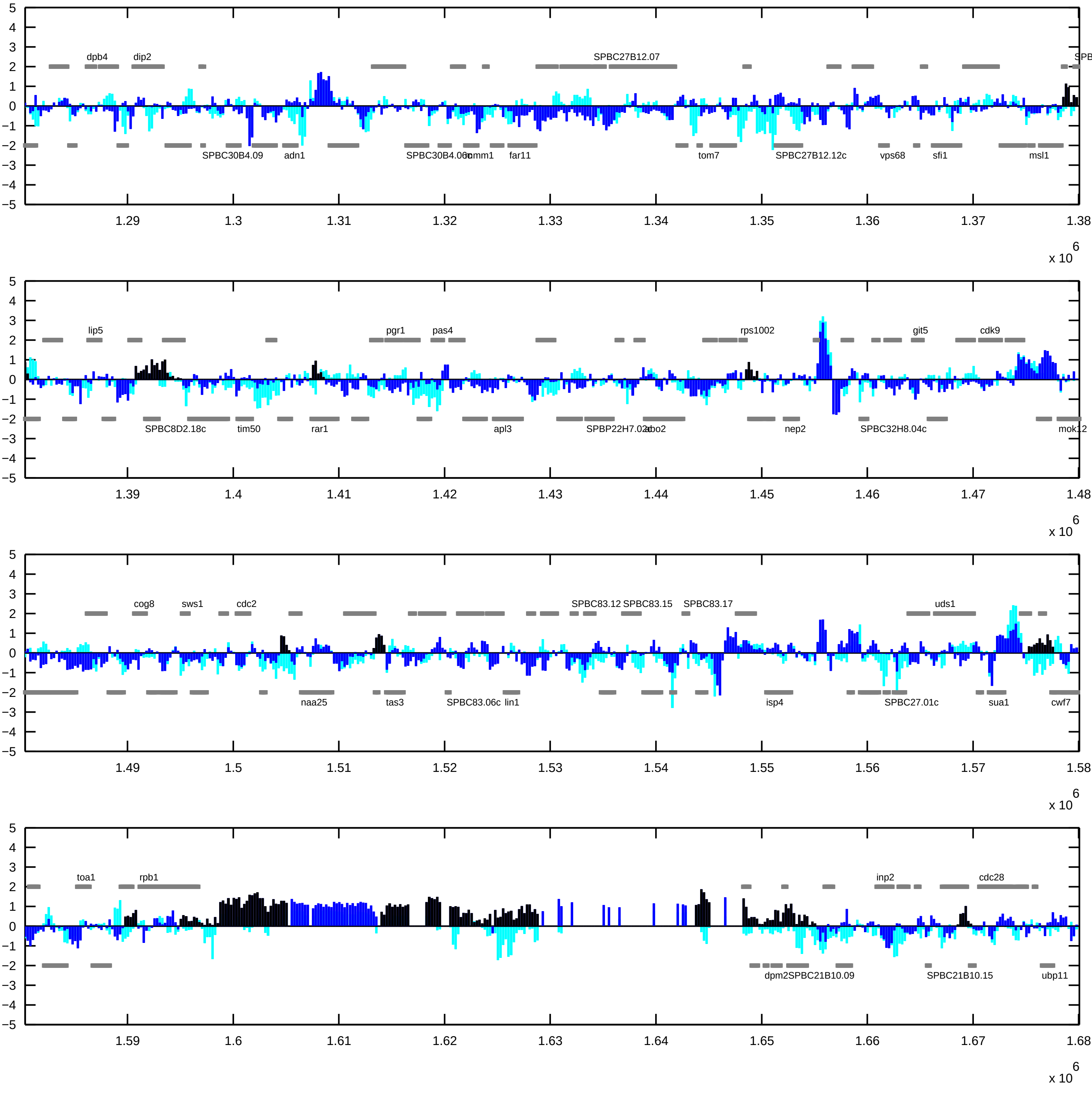
dpb4
dip2
SPBC27B12.07
SPBC30B4.09
adn1
SPBC30B4.06c
mmm1
far11
tom7
SPBC27B12.12c
vps68
sfi1
msl1
lip5
pgr1
pas4
rps1002
git5
cdk9
SPBC8D2.18c
tim50
rar1
apl3
SPBP22H7.02c
abo2
nep2
SPBC32H8.04c
mok12
cog8
sws1
cdc2
SPBC83.12
SPBC83.15
SPBC83.17
uds1
naa25
tas3
SPBC83.06c
lin1
isp4
SPBC27.01c
sua1
cwf7
toa1
rpb1
inp2
cdc28
dpm2
SPBC21B10.09
SPBC21B10.15
ubp11
x 10^6

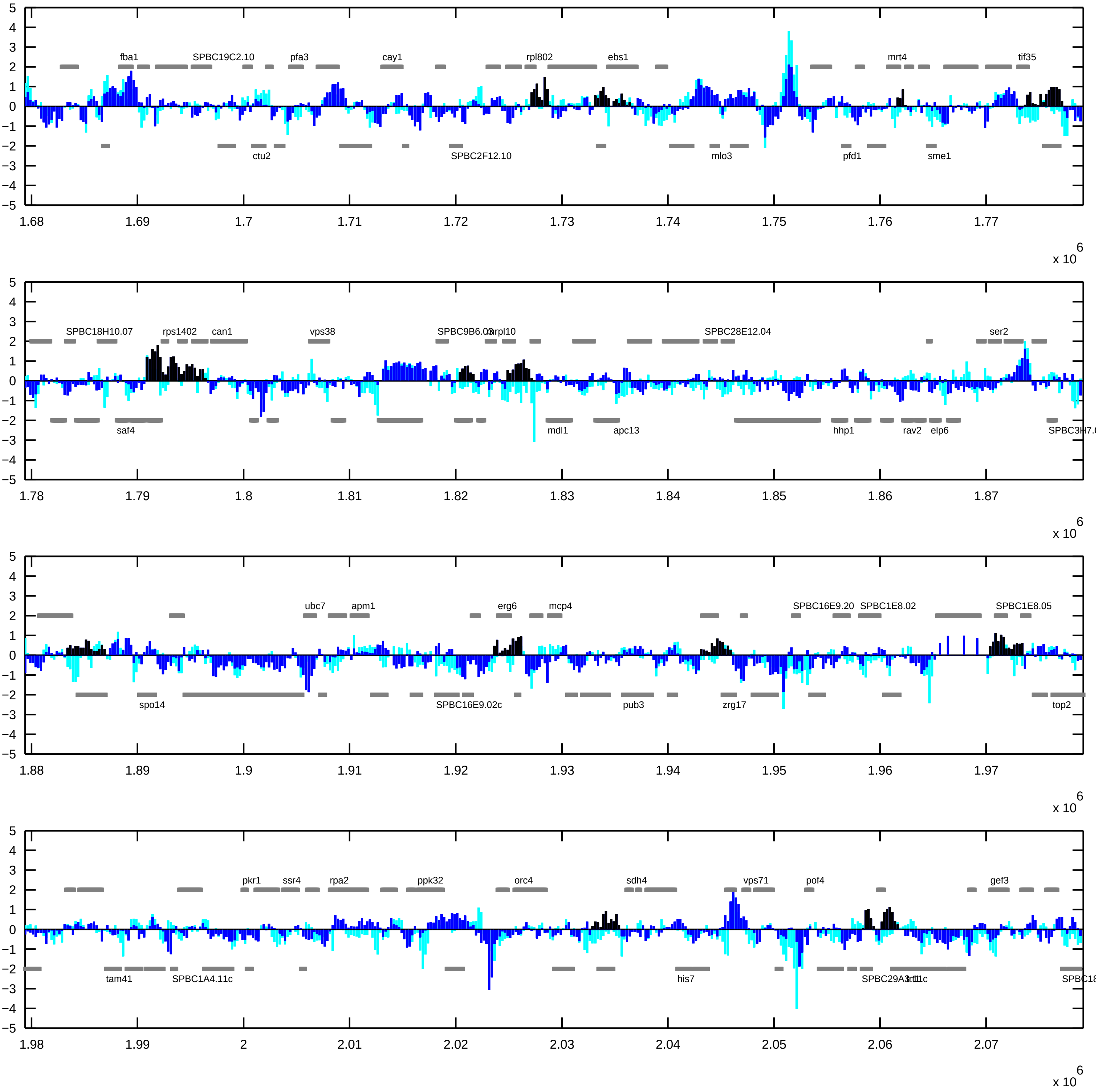
fba1
SPBC19C2.10
pfa3
cay1
rpl802
ebs1
mrt4
tif35
ctu2
SPBC2F12.10
mlo3
pfd1
sme1
x 10 6
SPBC18H10.07
rps1402
can1
vps38
SPBC28E12.04
ser2
saf4
mdl1
apc13
hhp1
rav2
elp6
ubc7
apm1
erg6
mcp4
SPBC16E9.20
SPBC1E8.02
SPBC1E8.05
spo14
SPBC16E9.02c
pub3
zrg17
top2
pkr1
ssr4
rpa2
ppk32
orc4
sdh4
vps71
pof4
gef3
tam41
SPBC1A4.11c
his7

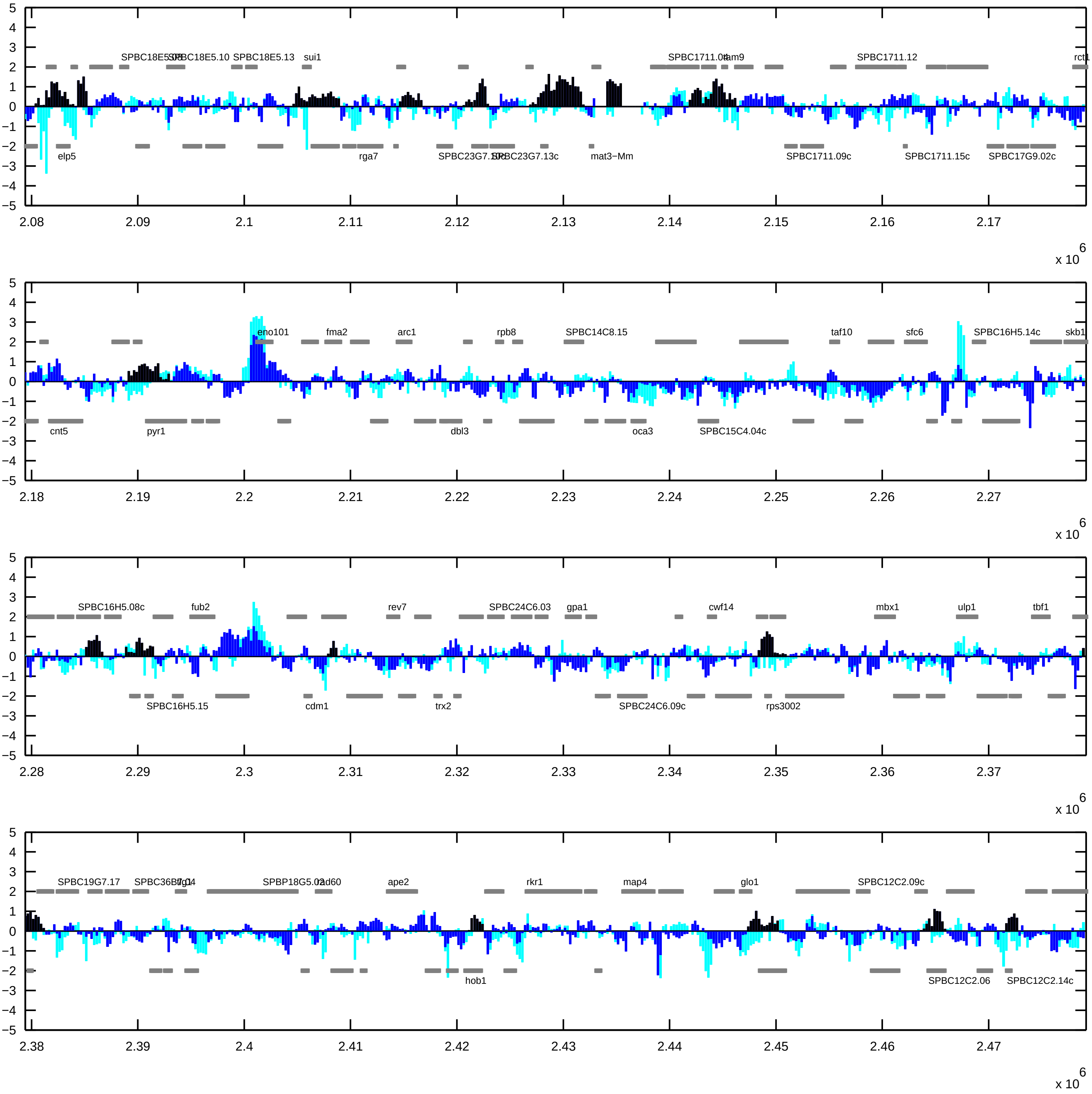
SPBC18E5.13
sui1
SPBC1711.12
elp5
rga7
SPBC23G7.13c
mat3−Mm
SPBC1711.09c
SPBC1711.15c
SPBC17G9.02c
2.08 2.09 2.1 2.11 2.12 2.13 2.14 2.15 2.16 2.17
eno101
fma2
arc1
rpb8
SPBC14C8.15
taf10
sfc6
SPBC16H5.14c
skb1
cnt5
pyr1
dbl3
oca3
SPBC15C4.04c
2.18 2.19 2.2 2.21 2.22 2.23 2.24 2.25 2.26 2.27
SPBC16H5.08c
fub2
rev7
SPBC24C6.03
gpa1
cwf14
mbx1
ulp1
tbf1
SPBC16H5.15
cdm1
trx2
SPBC24C6.09c
rps3002
2.28 2.29 2.3 2.31 2.32 2.33 2.34 2.35 2.36 2.37
SPBC19G7.17
ape2
rkr1
map4
glo1
SPBC12C2.09c
hob1
SPBC12C2.06
SPBC12C2.14c
2.38 2.39 2.4 2.41 2.42 2.43 2.44 2.45 2.46 2.47

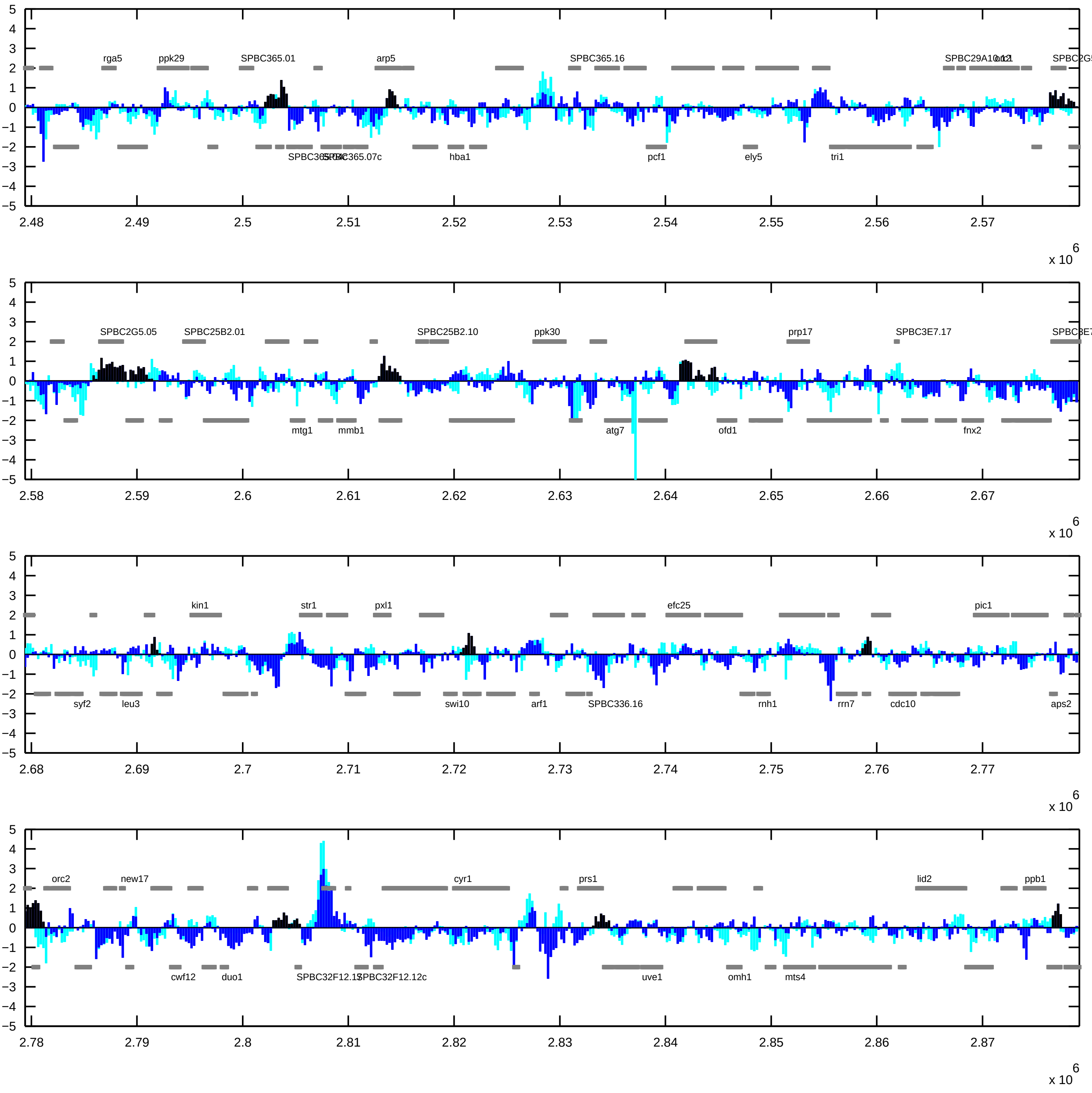
rga5
ppk29
SPBC365.01
arp5
SPBC365.16
SPBC29A10.12
SPBC365.07c
hba1
pcf1
ely5
tri1
x 10
6
SPBC2G5.05
SPBC25B2.01
SPBC25B2.10
ppk30
prp17
SPBC3E7.17
mtg1
mmb1
atg7
ofd1
fnx2
kin1
str1
pxl1
efc25
pic1
syf2
leu3
swi10
arf1
SPBC336.16
rnh1
rrn7
cdc10
aps2
orc2
new17
cyr1
prs1
lid2
ppb1
cwf12
duo1
SPBC32F12.12c
uve1
omh1
mts4

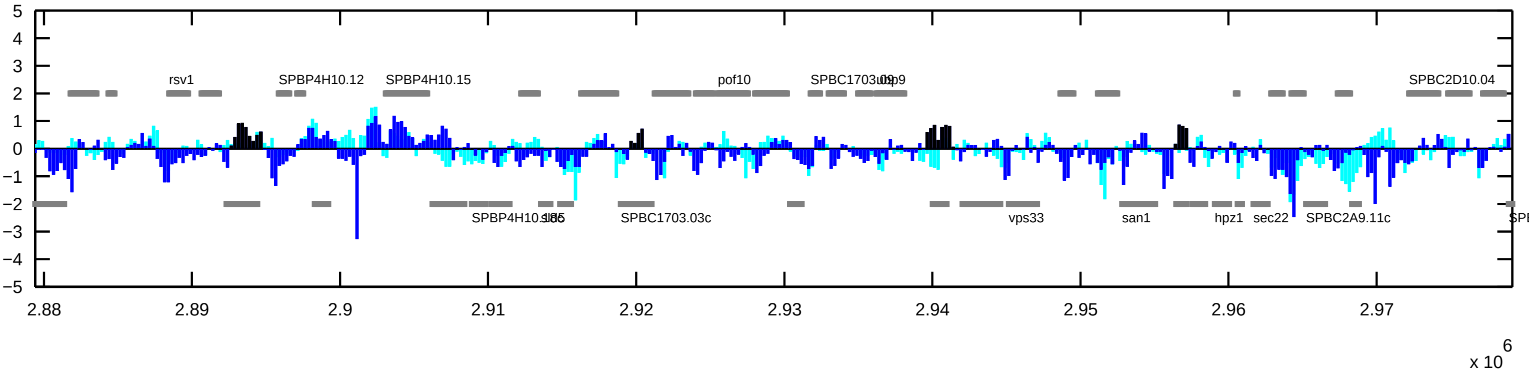

5
4
3
2
1
0
-1
-2
-3
-4
-5
rsv1
SPBP4H10.12
SPBP4H10.15
pof10
SPBC2D10.04
SPBC1703.03c
vps33
san1
hpz1
sec22
SPBC2A9.11c
2.88
2.89
2.9
2.91
2.92
2.93
2.94
2.95
2.96
2.97
x 10
6

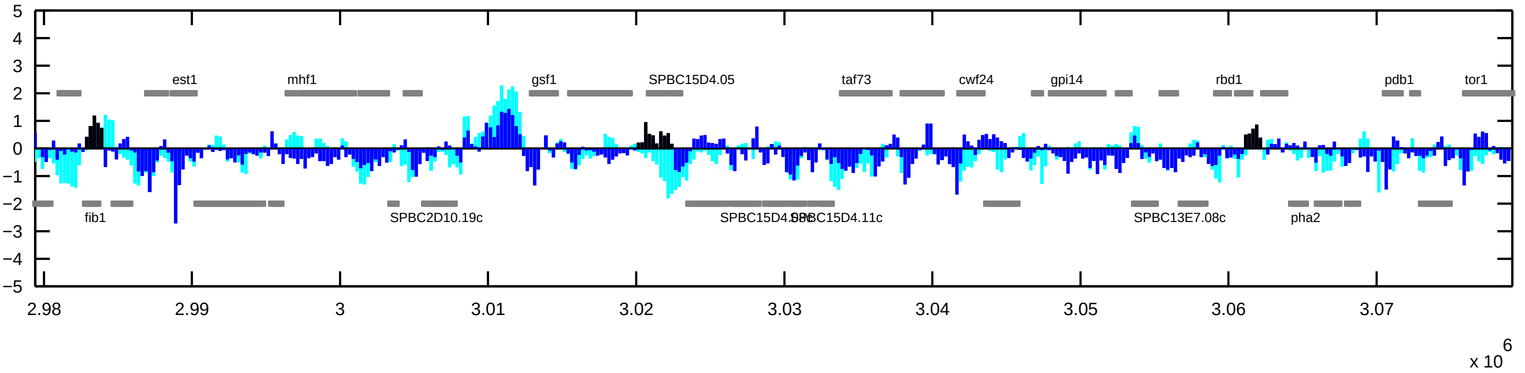

5
4
3
2
1
0
-1
-2
-3
-4
-5
est1
mhf1
gsf1
SPBC15D4.05
taf73
cwf24
gpi14
rbd1
pdb1
tor1
fib1
SPBC2D10.19c
SPBC13E7.08c
pha2
2.98
2.99
3
3.01
3.02
3.03
3.04
3.05
3.06
3.07
x 10
6

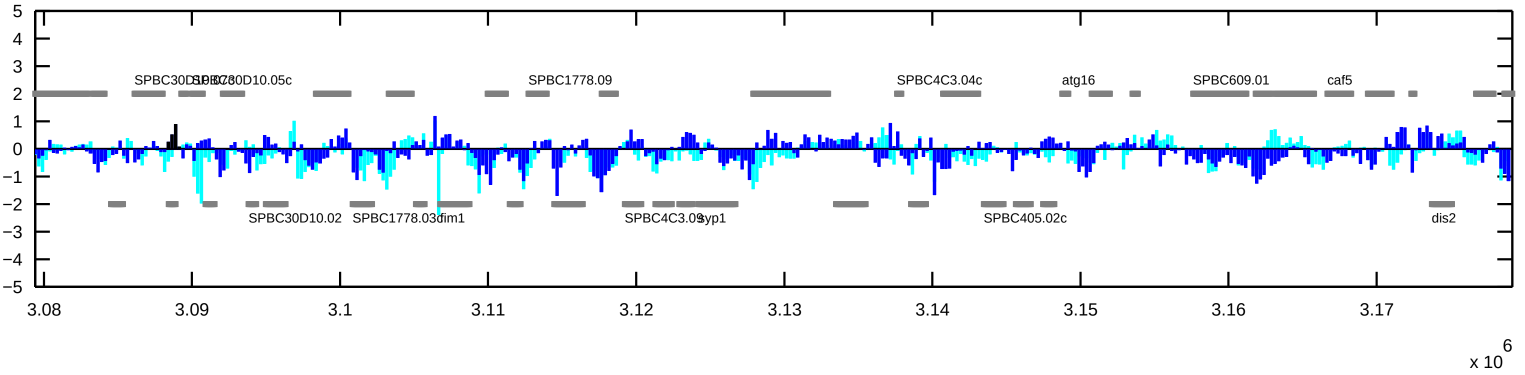

5
4
3
2
1
0
-1
-2
-3
-4
-5
SPBC1778.09
SPBC4C3.04c
atg16
SPBC609.01
caf5
SPBC30D10.02
SPBC405.02c
dis2
3.08
3.09
3.1
3.11
3.12
3.13
3.14
3.15
3.16
3.17
x 10
6

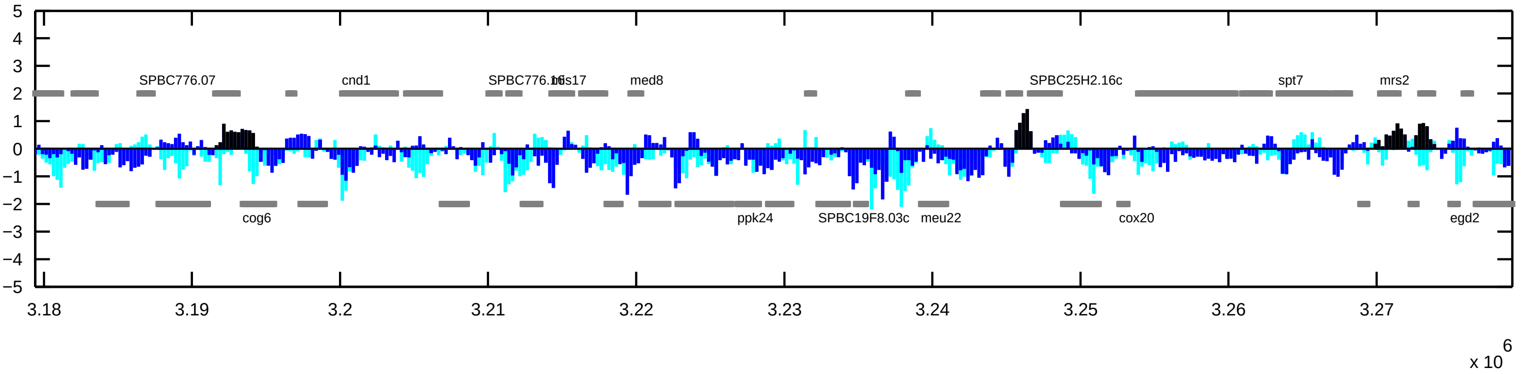

5
4
3
2
1
0
-1
-2
-3
-4
-5
SPBC776.07
cnd1
med8
SPBC25H2.16c
spt7
mrs2
cog6
ppk24
SPBC19F8.03c
meu22
cox20
egd2
3.18
3.19
3.2
3.21
3.22
3.23
3.24
3.25
3.26
3.27
x 10
6

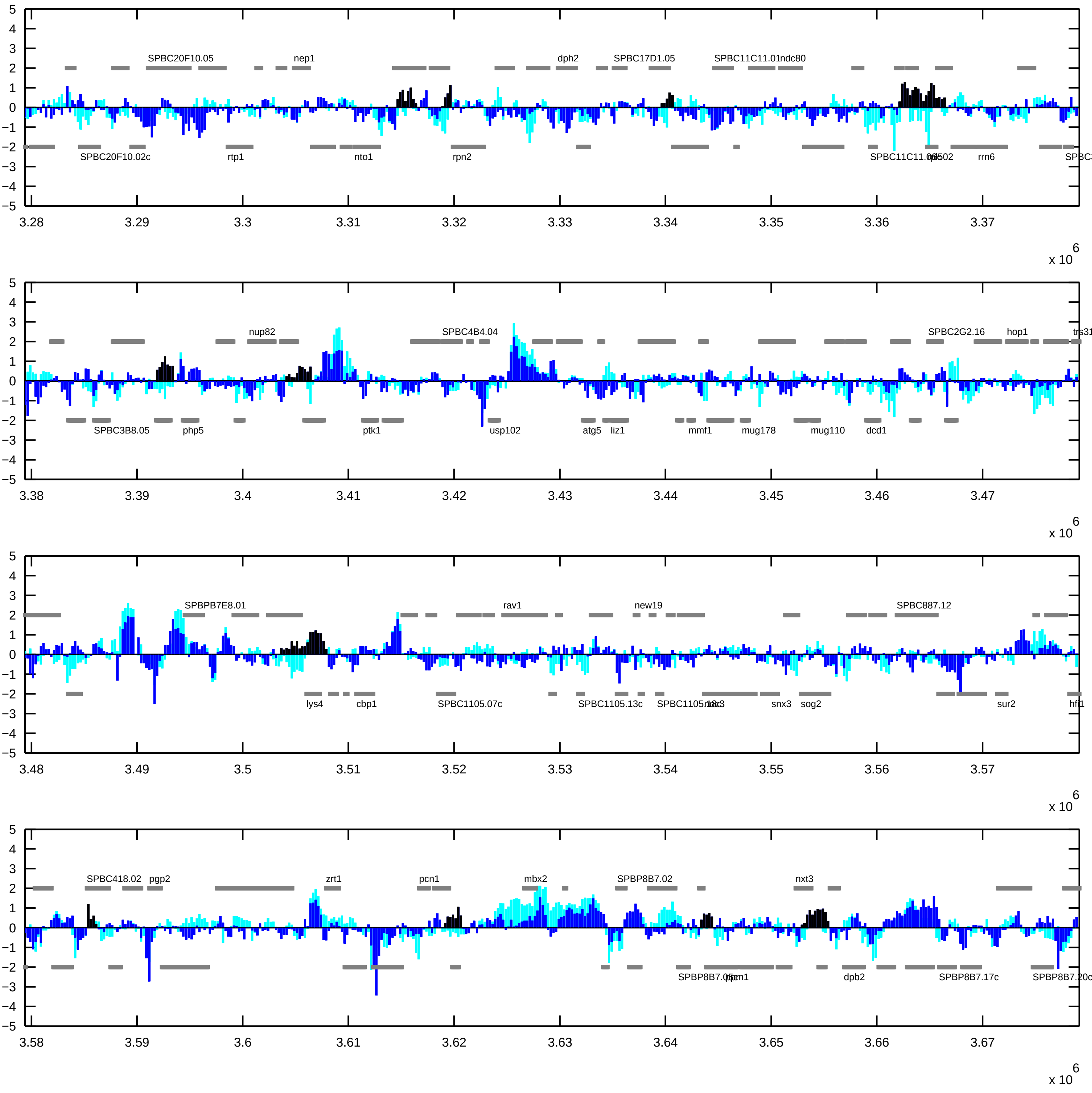
SPBC20F10.05
nep1
dph2
SPBC17D1.05
SPBC11C11.01
ndc80
SPBC20F10.02c
rtp1
nto1
rpn2
rrn6
nup82
SPBC4B4.04
SPBC2G2.16
hop1
SPBC3B8.05
php5
ptk1
usp102
atg5
liz1
mmf1
mug178
mug110
dcd1
SPBPB7E8.01
rav1
new19
SPBC887.12
lys4
cbp1
SPBC1105.07c
SPBC1105.13c
snx3
sog2
sur2
SPBC418.02
pgp2
zrt1
pcn1
mbx2
SPBP8B7.02
nxt3
dpb2
SPBP8B7.17c
x 10
6

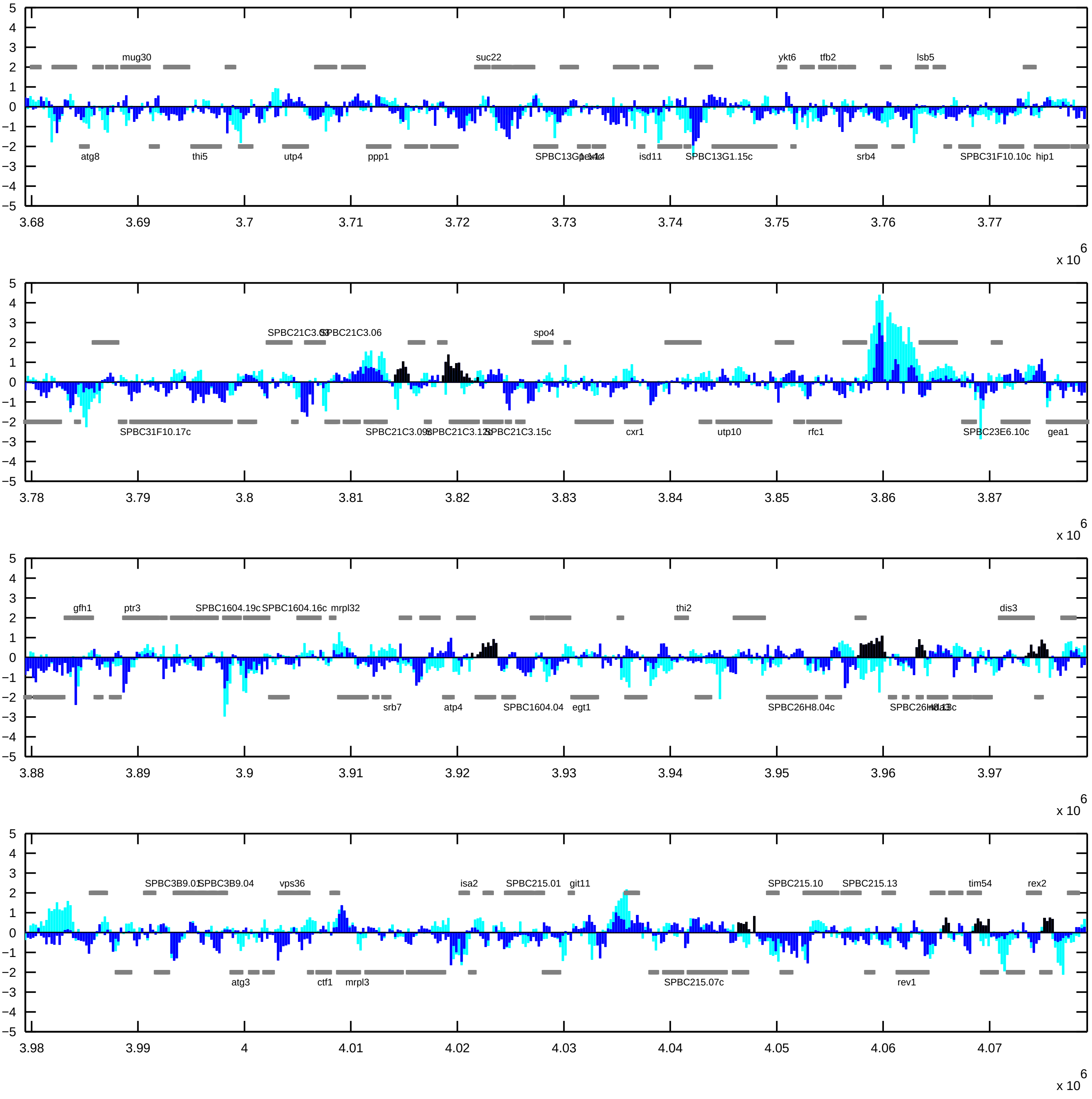
mug30
suc22
ykt6
tfb2
lsb5
atg8
thi5
utp4
ppp1
isd11
SPBC13G1.15c
srb4
SPBC31F10.10c
hip1
spo4
SPBC31F10.17c
cxr1
utp10
rfc1
SPBC23E6.10c
gea1
gfh1
ptr3
mrpl32
thi2
dis3
srb7
atp4
SPBC1604.04
egt1
SPBC26H8.04c
SPBC3B9.04
vps36
isa2
SPBC215.01
git11
SPBC215.10
SPBC215.13
tim54
rex2
atg3
ctf1
mrpl3
SPBC215.07c
rev1
x 10
6

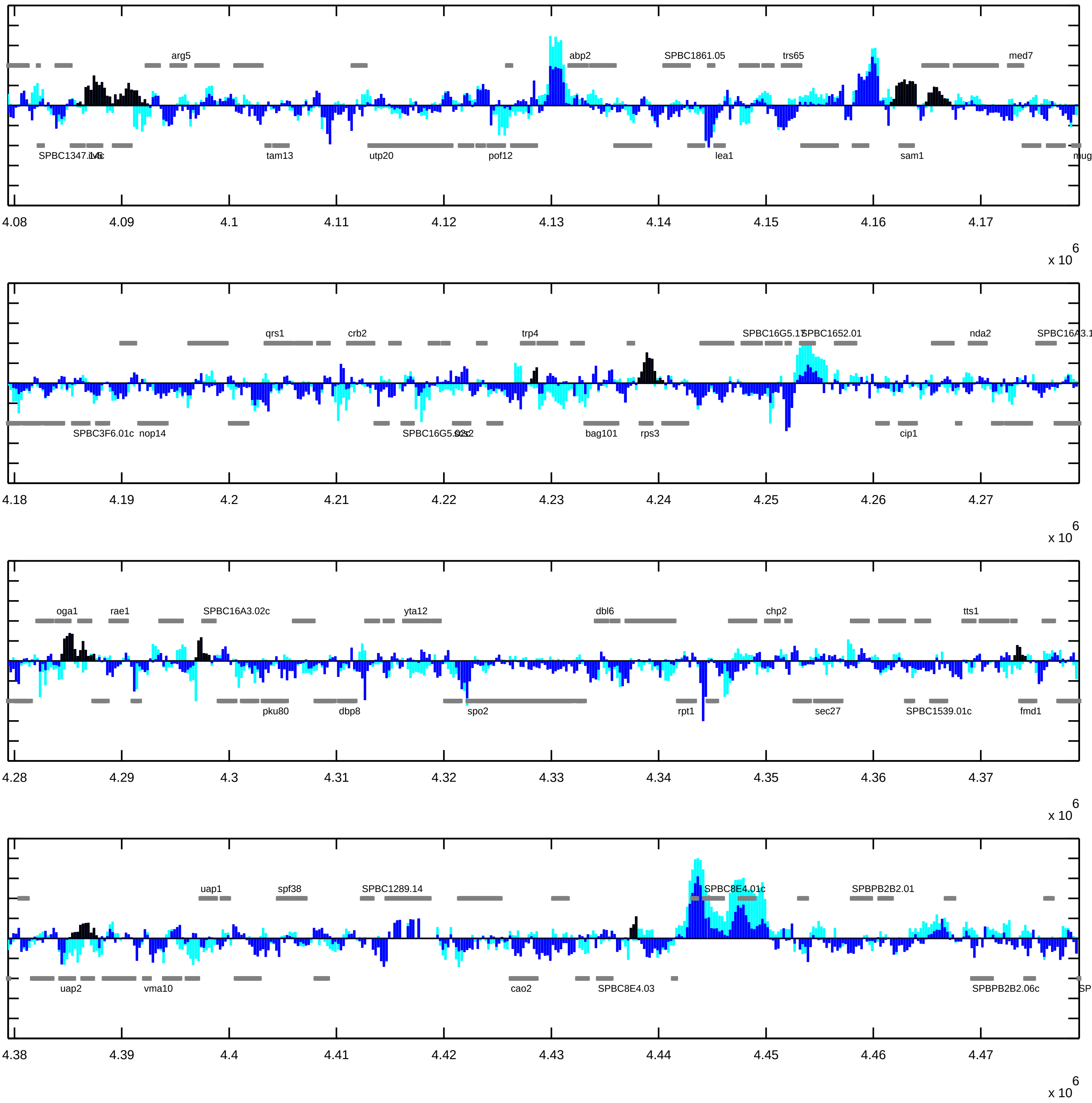
arg5
abp2
SPBC1861.05
trs65
med7
SPBC1347.14c
tam13
utp20
pof12
lea1
sam1
4.08 4.09 4.1 4.11 4.12 4.13 4.14 4.15 4.16 4.17
x 10^6
qrs1
crb2
trp4
SPBC1652.01
nda2
SPBC3F6.01c
nop14
bag101
rps3
cip1
4.18 4.19 4.2 4.21 4.22 4.23 4.24 4.25 4.26 4.27
x 10^6
oga1
rae1
SPBC16A3.02c
yta12
dbl6
chp2
tts1
pku80
dbp8
spo2
rpt1
sec27
SPBC1539.01c
fmd1
4.28 4.29 4.3 4.31 4.32 4.33 4.34 4.35 4.36 4.37
x 10^6
uap1
spf38
SPBC1289.14
SPBC8E4.01c
SPBPB2B2.01
uap2
vma10
cao2
SPBC8E4.03
SPBPB2B2.06c
4.38 4.39 4.4 4.41 4.42 4.43 4.44 4.45 4.46 4.47
x 10^6

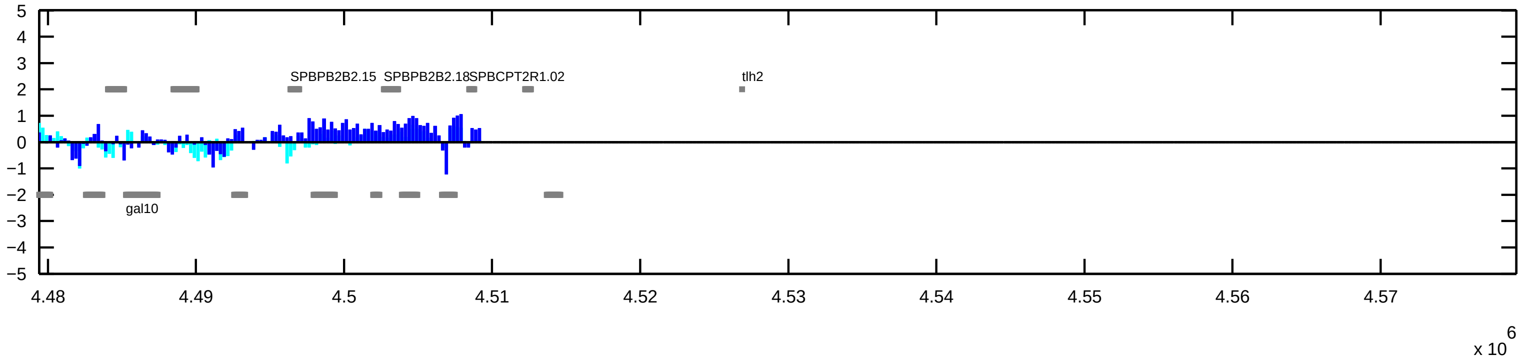
SPBPB2B2.15
SPBPB2B2.18
SPBCPT2R1.02
tlh2
gal10
5
4
3
2
1
0
−1
−2
−3
−4
−5
4.48
4.49
4.5
4.51
4.52
4.53
4.54
4.55
4.56
4.57
x 10^6
